# Evolution of organellar genes of chlorophyte algae: Relevance to phylogenetic inference

**DOI:** 10.1371/journal.pone.0216608

**Published:** 2019-05-06

**Authors:** Nuttapong Mekvipad, Anchittha Satjarak

**Affiliations:** Department of Botany, Chulalongkorn University, Bangkok, Thailand; National Cheng Kung University, TAIWAN

## Abstract

Protein-coding genes in organellar genomes have been widely used to resolve relationships of chlorophyte algae. The mode of evolution of these protein-coding genes affects relationship estimations, yet selection effects on genes commonly used as markers in phylogenetic analyses are insufficiently well understood. To gain more understanding about the effects of green algal organelle protein-coding genes on phylogenies, more information is needed about the mode of gene evolution. We used phylogenetic frameworks to examine evolutionary relationships of 58 protein-coding genes present in the organellar genomes of chlorophyte and streptophyte algae at multiple levels: organelle, biological function, and individual gene, and calculated pairwise dN/dS ratios of algal organellar protein-coding genes to investigate mode of evolution. Results indicate that mitochondrial genes have evolved at a higher rate than have chloroplast genes. Low dN/dS ratios indicating relatively high level of conservation indicate that *nad*2, *nad*5, *atp*A, *atp*E, *psb*C, and *psb*D might be particularly good candidates for use as markers in chlorophyte phylogenies. Chlorophycean *atp*6, *nad*2, *atp*F, *clp*P, *rps*2, *rps*3, *rps*4, and *rps*7 protein-coding sequences exhibited selective mutations, suggesting that changes in proteins encoded by these genes might have increased fitness in Chlorophyceae.

## Introduction

Land plants (embryophytes), closely-related streptophyte green algae, and chlorophyte green algae together form the Viridiplantae [1]. The modern Viridiplantae are (mostly) oxygenic photosynthetic eukaryotes hypothesized to have descended from a single common ancestor after the acquisition of primary plastids[2]. The chlorophyte algae, representing the majority of known green algal species, include three major algal classes (Chlorophyceae, Trebouxiophyceae, and Ulvophyceae), some smaller classes (e.g. Chlorodendrophyceae), and a paraphyletic assemblage of prasinophytes having traits considered to represent those of the last common Viridiplantae ancestor[3,4].

Although molecular data available to date indicate that the class Chlorophyceae is probably a monophyletic group, monophyly of Ulvophyceae and Trebouxiophyceae has been questioned[5–7], and chloroplast genome data have increasingly been used to explore this issue[3]. Studies based upon chloroplast protein-coding gene sequence comparisons have supported a concept of monophyly for both Ulvophyceae and Chlorophyceae and indicated their sister relationship[5–9]. However, an inference of monophyly for Ulvophyceae has depended on taxa and genes employed in phylogenetic analyses. For example, when species of Bryopsidiales, typically considered ulvophycean, were added to analyses, Bryopsidiales branched outside Ulvophyceae, sister either to a clade consisting of Trebouxiophyceae[6], or to Chlorodendrophyceae[7]. However, monophyletic Ulvophyceae (bootstrap value = 90) was resolved when a combined data set of chloroplast and mitochondrial protein-coding genes was employed in an analysis of selected taxa[4].

Likewise, phylogenetic relationships of Trebouxiophyceae have been analyzed using chloroplast genomic sequence data. Smith et al. (2011) and Yan et al. (2015) reported that chloroplast protein coding genes suggest the monophyly of a clade consisting of core Trebouxiophyceae plus Chlorellales, whereas Turmel et al. (2016) and Lemieux et al. (2014) suggested that Chlorellales is sister to a clade consisting of Chlorophyceae, Ulvophyceae, plus core Trebouxiophyceae [5,7–9]. These differences indicate the need for further investigation of the degree to which the use of both mitochondrial and chloroplast genomes may be useful and if heterogeneity in gene evolution may have influenced the utility of organellar genomes in resolving chlorophyte phylogeny.

Sets of concatenated sequences are commonly used to sidestep phylogenetic problems arising from incongruence of single gene trees and differing modes of gene evolution. However, the construction of phylogenetic trees from different sets of concatenated nucleic acid sequences or protein alignments can also result in incongruent tree topologies. For example, a study of the plant order Liliales suggested that concatenated nucleotide sequences of protein-coding genes grouped by their biological function, selection force, and substitution rates yield different tree topologies. These differences arise from heterogeneity in rates of nucleotide evolution[10,11]. Differences in organellar genome architecture are also potential sources of phylogenetic issues in green algae, as illustrated by effects of plastid *rpo*B expansion[12]. To gain more understanding about the effects of green algal organelle protein-coding genes on phylogenies, we need to understand evolutionary scenarios that might have taken place in chlorophyte histories. In this study, we used the power of phylogenetic analyses to investigate the evolutionary history of chlorophyte organellar protein-coding genes at multiple levels, and considered whether the information at those levels was sufficient to resolve monophyly of known clades. Our results suggested that chlorophyte organellar genes were subjected to variable levels of evolutionary force, indicating that particular organellar genes might be more informative for estimating relationships of different clades.

## Materials and methods

### Sequence dataset assembly

The dataset for this study included protein-coding sequences from complete mitochondrial and chloroplast genomes of 26 green algal species and 1 glaucophyte, *Cyanophora paradoxa* employed as an outgroup, which are publicly available in Genbank (accessed in December 2017). The algal organellar genomes in the dataset were selected based on the following criteria: 1) the genomes were complete, 2) protein coding regions were annotated, and 3) both mitochondrial and chloroplast genomes of the same algal species/strain were available. Only protein-coding sequences present in all selected taxa were employed in the analysis. Genes from mitochondrial and chloroplast genomes were categorized into the following biological processes: translation, electron transport (photosystem I, photosystem II, and cytochrome b6f complex), NADH synthesis, and ATP synthesis. Using these criteria, we constructed a data matrix of 27 algal species, each with 58 protein-coding sequences– 13 mitochondrial sequences and 45 plastid sequences (Table 1). The corresponding plastid and mitochondrial genes of *C*. *paradoxa* were used as outgroups.

**Table 1:** List of protein-coding sequences from 27 green algal species used in this study.

species name	Mitochondrial genome accession number	Plastid genome accession number	Mitochondrial genes	Plastid genes
*Auxenochlorella protothecoides*	KC843974.1	KC843975.1	*atp*6, *atp*9, *cob*, *cox*1, *cox*2, *cox*3, *nad*1, *nad*2, *nad*3, *nad*4, *nad*4L, *nad*5, *nad*6	*atp*A, *atp*B, *atp*E, *atp*F, *atp*H, *clp*P, *pet*A, *pet*B, *pet*G, *psa*A, *psa*B, *psa*C, *psb*A, *psb*B, *psb*C, *psb*D, *psb*E, *psb*F, *psb*H, *psb*I, *psb*J, *psb*K, *psb*L, *psb*N, *psb*T, *psb*Z, *rbc*L, *rpl*2, *rpl*5, *rpl*14, *rpl*16, *rpl*20, *rpl*36, *rps*2, *rps*3, *rps*4, *rps*7, *rps*8, *rps*11, *rps*12, *rps*14, *rps*18, *rps*19, *ycf*3, *ycf*12
*Bracteacoccus aerius*	KJ806265.1	KT199254.1		
*Chaetosphaeridium globosum*	NC_009630.1	AF494278.1		
*Chara vulgaris*	AF494279.1	NC_008097.1		
*Chlorella sorokiniana* UTEX1230	KF554428.1	KJ742376.1		
*Chlorella sp*. ArM0029B	NC_024757.1	KF554427.1		
*Chlorella variabilis* NC64A	HQ874522.1	KJ718922.1		
*Chlorokybus atmophyticus*	KJ742377.1	NC_008822.1		
*Chlorotetraedron incus* SAG43.81	KM252919.1	NC_029673.1		
*Chromochloris zofingiensis* UTEX56	NC_005255.1	NC_029672.1		
*Coccomyxa subellipsoidea* C-169	NC_024758.1	HQ693844.1		
*Entransia fimbriata* UTEX LB2353	NC_022861.1	NC_030313.1		
*Gloeotilopsis planctonica* SAG29.93	KX306823.1	KX306824.1		
*Gloeotilopsis sarcinoidea* UTEX1710	KX306822.1	KX306821.1		
*Mesostigma viride*	AF353999.1	AF166114.1		
*Neochloris aquatica* UTEX B138	NC_024761.1	NC_029670.1		
*Nephroselmis olivacea*	AF110138.1	AF137379.1		
*Oltmannsiellopsis viridis*	KC967306.1	DQ291132.1		
*Ostreococcus tauri* RCC1561	DQ365900.1	KF285533.1		
*Prasinoderma coloniale* CCMP1220	AY359242.1	KJ746598.1		
*Pseudendoclonium akinetum*	KF387569.1	AY835431.1		
*Pseudomuriella schumacherensis* SAG2137	KX013547.1	KT199256.1		
*Pyramimonas parkeae* NIES254	KJ806273.1	KX013546.1		
*Ulva fasciata*	KU182748.1	KT882614.1		
*Ulva flexuosa*	KX455878.1	KX579943.1		
*Ulva sp*. UNA00071828	KP720617.1	KP720616.1		

### Sequence alignment, concatenations, and phylogenetic analyses

#### Protein-coding sequences

A combined data set of mitochondrial and plastid protein-coding genes was aligned using codon-based alignment in MAFFT v 7.205[13]. We concatenated these sequences using Sequence Matrix (Vaidya et al., 2010); the concatenated sequences included 42,785 nucleotide positions. We computed nucleotide substitution models using jModelTest [14] and performed Maximum-likelihood (ML) analyses using RAxML v 8.2.8 [15] on the CIPRES XSEDE Portal [16], using a GTR+Γ+I nucleotide substitution model and rapid bootstrapping method with 1,000 replications for bootstrap analyses. We performed Bayesian analysis using MrBayes v 3.2.6 [17] using the GTR+Γ+I substitution model. Four independent chains were run for 1,100,000 cycles and the consensus topologies were calculated after the burn-in of 100,000 cycles.

We first asked whether concatenated sequences from mitochondrial and chloroplast genomes, considered separately, would resolve the same phylogenetic relationship. We used jModelTest [14] to compute nucleotide substitution models and performed Maximum-likelihood (ML) analyses using RAxML v 8.2.8 [15] on the CIPRES XSEDE Portal[16], using a GTR+Γ+I nucleotide substitution model and rapid bootstrapping method with 1,000 replications for bootstrap analyses. We performed Bayesian analysis using MrBayes v 3.2.6 [17] using a GTR+Γ+I substitution model. Four independent chains were run for 1,100,000 cycles and the consensus topologies were calculated after the burn-in of 100,000 cycles.

To investigate whether genes encoding proteins for particular biological function provide sufficient information to resolve the monophyly of the known clades, we then concatenated protein-coding sequences based on their biological functions–mitochondrial NADH dehydrogenase (complex I) subunits, cytochrome c oxidase (complex IV) subunits, ATP synthase (complex V) subunits, and chloroplast ribosomal protein subunits, photosystem I subunits, photosystem II subunits, and cytochrome b6f complex subunits. We aligned the nucleotide sequences using MAFFT v 7.205 [13] and calculated their substitution models using jModelTest[14]. The alignment lengths of protein coding sequences and their predicted substitution model were shown in S1 Table. Then, using a GTR+I+G substitution model, on the CIPRES XSEDE Portal[16], we performed Maximum-likelihood (ML) analyses using RAxML v 8.2.8 [15] with, rapid bootstrapping method with 1,000 replications for bootstrap analyses. Bayesian analysis was performed using MrBayes v 3.2.6 [17] using the substitution model present in S1 Table. Four independent chains were run for 1,100,000 cycles and the consensus topologies were calculated after the burn-in of 100,000 cycles.

To study the evolutionary pattern of each mitochondrial and plastid protein-coding gene and to determine if the protein-coding sequences of a single gene was sufficient to resolve the relationships of major chlorophyte clades, we performed single-gene phylogenetic analyses using selected genes from the organelle genomes of the selected green algal strains from Table 1. We aligned algal protein-coding sequences using MAFFT v 7.205 [13], trimmed the alignment using automated1 option implemented in trimAL v 1.2 [18], and computed their nucleotide substitution models using jModelTest[14]. The alignment lengths of protein-coding genes and their predicted substitution model are shown in S2 Table. Then, using a suitable substitution model, on the CIPRES XSEDE Portal[16], we performed Maximum-likelihood (ML) analyses using RAxML v 8.2.8 [15] with, rapid bootstrapping method with 1,000 replications for bootstrap analyses. Bayesian analysis was performed using MrBayes v 3.2.6 [17]. Four independent chains were run for 1,100,000 cycles and the consensus topologies were calculated after the burn-in of 100,000 cycles.

#### Ribosomal rDNA

To investigate the relationship of green algal taxa using ribosomal rDNA, we used plastid rrnL and rrs of the algal taxa from Table 1. We aligned the rDNA sequences using in MAFFT v 7.205[13], trimmed the alignment using automated1 option implemented in trimAL v 1.2 [18], and concatenated the alignments using Sequence Matrix (Vaidya et al., 2010); the concatenated sequences included 4,222 nucleotide positions. We computed nucleotide substitution models using jModelTest [14] and performed Maximum-likelihood (ML) analyses using RAxML v 8.2.8 [15] on the CIPRES XSEDE Portal [16], using a GTR+Γ+I nucleotide substitution model and rapid bootstrapping method with 1,000 replications for bootstrap analyses. We performed Bayesian analysis using MrBayes v 3.2.6 [17] using the GTR+Γ+I substitution model. Four independent chains were run for 1,100,000 cycles and the consensus topologies were calculated after the burn-in of 100,000 cycles.

To understand the mode of evolution of each protein coding gene, we looked for the presence of mutations–synonymous and nonsynonymous substitutions–occurring in protein coding genes commonly present in the selected chlorophyte species. We calculated synonymous substitution and nonsynonymous substitution using MEGA v 7.0.26 [19] and manually calculated the pairwise ratios of synonymous substitution and nonsynonymous substitutions. Then we translated the numerical values of synonymous substitution and nonsynonymous substitutions ratios into a heatmap using an in-house R script. The alignments and the in-house R script used in this study have been deposited at https://github.com/NMekvipad/dndsHM.

## Results

In this study, we employed the power of phylogenetic analyses to investigate the evolution of organellar protein-coding genes of chlorophyte algal species and investigated whether the information was sufficient to resolve monophyly of known clades.

### Chlorophyte relationships based on combined mitochondrial and plastid protein-coding sequence data

**Fig 1:**
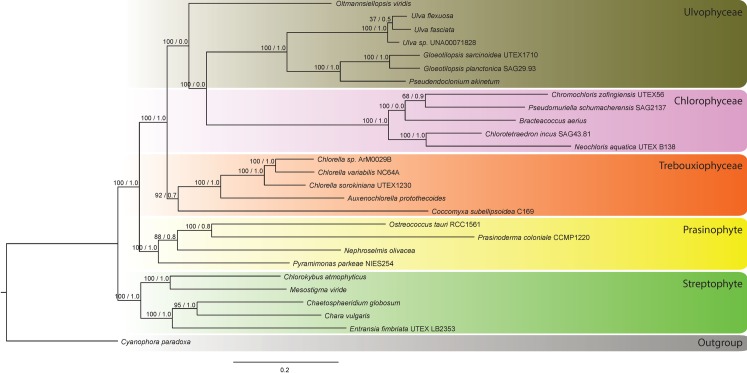
Maximum-Likelihood tree inferred from 58 protein-coding sequences– 13 mitochondrial and 45 plastid genes. Bootstrap value from ML and posterior probability from Bayesian inference are shown at the node. The scale bar represents the estimated number of nucleotide substitution per site.

Analyses of combined mitochondrial and chloroplast genomes resolved the monophyly of Chlorophyceae and the monophyly of Trebouxiophyceae. Trebouxiophyceae was sister to a clade consisting of Chlorophyceae plus Ulvophyceae. All streptophyte algae formed a clade diverged from chlorophyte algae (Fig 1).

### Chlorophyte relationships based on separate analysis of mitochondrial and plastid protein-coding sequences

The tree estimated from concatenated mitochondrial protein-coding genes resolved the a monophyletic Chlorophyceae and a clade of streptophyte algae. The ingroup taxa formed two clades: 1) a clade of Chlorophyceae plus Ulvophyceae and 2) a clade of Trebouxiophyceae plus Prasinophyceae plus streptophyte algae (Fig A in S1 File and S3 Table). The tree estimated from chloroplast protein-coding sequences similarly resolved Chlorophyceae monophyly and a clade of streptophyte algae. In this tree, ingroup taxa formed two clades: 1) a clade consisting of core chlorophytes–Chlorophyceae, Trebouxiophyceae, and Ulvophyceae and prasinophytes–and 2) a clade consisting of streptophyte algae (Fig B in S1 File and S3 Table).

### Chlorophyte relationships based on mitochondrial and plastid protein-coding genes categorized by biological function

We used concatenated protein-coding sequences relating to the same biological functions to estimate chlorophyte relationships. We found that protein-coding sequences of mitochondrial NADH dehydrogenase (complex I) subunits resolved monophyletic clades of Chlorophyceae and Ulvophyceae; that of mitochondrial cytochrome c oxidase (complex IV) subunits resolved 1) monophyletic clade of Chlorophyceae and 2) a clade consisting of streptophyte algae; that of mitochondrial ATP synthase (complex V) subunits resolved monophyletic Chlorophyceae (Figs A-C in S2 File and S3 Table).

Using chloroplast protein-coding genes, we found that plastid protein-coding sequences encoding plastid ATP synthase resolved monophyly of the algal clades Chlorophyceae, Trebouxiophyceae, Ulvophyceae, and a clade consisting of streptophyte algae. Protein-coding genes of large subunits of ribosomal proteins resolved the monophyly of Chlorophyceae and a clade of streptophyte algae; those of small subunits of ribosomal proteins resolved the monophyletic clades Chlorophyceae, Ulvophyceae, and a clade consisting of streptophyte algae; those of photosystem I subunits resolved monophyly of Chlorophyceae and a clade of streptophyte algae; those of photosystem II subunits resolved the monophyletic clades of Chlorophyceae, Ulvophyceae and a clade of streptophyte algae; and those of plastid cytochrome b6f complex subunits resolved the monophyly of Chlorophyceae and a clade of streptophyte algae (Figs D-I in S2 File and S3 Table).

### Chlorophyte relationships based on single protein-coding sequences from mitochondrial genomes

We used 13 mitochondrial protein-coding genes (Table 1) in our single-gene phylogenetic analyses. Our results showed that genes encoding NADH dehydrogenase (complex I) subunits *nad*1, *nad*2, *nad*3, *nad*4, *nad*4L, *nad*5, and *nad*6 resolved the monophyly of Chlorophyceae. *nad*2 resolved monophyly of Trebouxiophyceae. *nad*5 resolved the monophyly of Ulvophyceae. None of the mitochondrial single-gene estimations resolved monophyly of the clade consisting of streptophyte algae. Genes encoding Cytochrome c oxidase (complex IV) subunits, *cox*2 and *cox*3 resolved the monophyly of Chlorophyceae, but none resolved monophyly of Trebouxiophyceae or monophyly of Ulvophyceae. Protein-coding sequences from ATP synthase (complex V) subunits *atp*6 and *atp*9 resolved the monophyly of Chlorophyceae but none resolved the monophyly of Trebouxiophyceae or Ulvophyceae (Figs A-K in S3 File and S3 Table).

### Chlorophyte relationships based on single protein-coding sequences from chloroplast genomes

We performed similar analyses using 45 plastid protein-coding sequences (Table 1). Our results showed that genes involved in plastid ATP synthase–*atp*A, *atp*B, *atp*E, and *atp*F–resolved the monophyly of Chlorophyceae, but none resolved the monophyly of Trebouxiophyceae. *atp*A, *atp*E, and atp*H* resolved the monophyly of Ulvophyceae. *atp*A and *atp*B resolved a clade consisting of streptophyte algae. Genes involved in the photosynthetic apparatus–*pet*A, *psa*A, *psa*B, *psa*C, *psb*B, *psb*C, *psb*D, *psb*E, *psb*H, *psb*N, and *psb*Z–resolved the monophyly of Chlorophyceae. None resolved the monophyly of Trebouxiophyceae. *psb*C, and *psb*D resolved the monophyly of Ulvophyceae. *pet*A, *psa*A, *psa*B, *psb*B, *psb*C, *psb*D, and *psb*E resolved the clade consisting of streptophyte algae (Figs L-AA in S3 File and S3 Table).

Plastid protein-coding genes that were involved in translation processes included *rpl* and *rps* genes. Of these, single-gene phylogenetic estimations of *rpl*2, *rpl*5, *rpl*14, *rpl*16, *rpl*20, *rps*2, *rps*3, *rps*4, *rps*7, *rps*8, *rps*11, *rps*12, *rps*14, *rps*18, and *rps*19 resolved the monophyly of Chlorophyceae. None of the genes encoding for rpl and rps proteins resolved the monophyly of Trebouxiophyceae or Ulvophyceae. *rpl*2, *rpl*5, *rps*2, and *rps*12 resolved the clade consisting of streptophyte algae. The conserved sequence *ycf*3 resolved the monophyletic clades of Chlorophyceae and Ulvophyceae. *ycf*12 did not resolved the monophyly of any known clade (Figs AB-AQ in S3 File and S3 Table). *clp*P resolved the monophyly of Chlorophyceae. Among genes functioning in carbon fixation, only *rbc*L was used in this study, but a single-gene phylogenetic tree constructed from *rbc*L did not resolve monophyly of any of the known clades in chlorophyte lineage–Chlorophyceae, Trebouxiophyceae, and Ulvophyceae (Figs AR and AS in S3 File and S3 Table). Some mitochondrial or plastid protein-coding sequences did not resolve any known class-level clades; these included *cob*, *cox*1, *pet*B, *pet*G, *psb*A, *psb*F, *psb*I, *psb*J, *psb*K, *psb*L, *psb*T, *rpl*36, and *ycf*12 (Figs AT-BF in S3 File).

### Chlorophyte relationships based on plastid ribosomal rRNA genes

Analyses of combined plastid rrnL and rrs resolved the monophyly of Chlorophyceae, Trebouxiophyceae, and Ulvophyceae. Chlorophyceae was sister to a clade consisting of Trebouxiophyceae plus Ulvophyceae. Prasinophytes appeared as a paraphyletic assemblage. All streptophyte algae formed a clade diverged from core chlorophyte algae (Figs BG in S3 File).

### Evolutionary rates and mode of selection

To understand mode of evolution of each mitochondrial and plastid protein-coding gene, we performed single-gene pairwise comparative analyses of non-synonymous and synonymous substitution (dN/dS) ratios of all selected algal taxa (Table 1). Then, we generated heatmaps from the resulting pairwise dN/dS ratios. The cladograms of algal taxa in these heatmaps corresponded to their phylogenetic trees estimated using single-gene phylogenetic analyses in the previous steps (Figs A-BF in S3 File).

Results showed that 8 mitochondrial genes and 35 plastid genes exhibited pairwise dN/dS ratios that ranged from 0 to < 1.0. These included mitochondrial *atp*9 (0.0–0.8), *cox*3 (0.0–0.8), *nad*1 (0.0–0.7) and *nad4*L (0.0–0.9), and plastid *atp*A (0.0–0.4), *atp*B (0.0–0.3), *atp*H (0.0–0.3), *pet*A (0.0–0.7), *pet*B (0.0–0.2), *pet*G (0.0–0.5), *psa*A (0.0–0.2), *psa*B (0.0–0.2), *psa*C (0.0–0.4), *psb*A (0.0–0.6), *psb*B (0.0–0.3), *psb*C (0.0–0.3), *psb*D (0.0–0.2), *psb*E (0.0–0.4), *psb*H (0.0–0.3), *psb*I (0.0–0.5), *psb*K (0.0–0.6), *psb*L (0.0–0.4), *psb*N (0.0–0.9), *psb*T (0.0–0.4), *rbc*L (0.0–0.3), *rpl*2 (0.0–0.8), *rpl*5 (0.0–0.9), *rpl*14 (0.0–0.4), *rpl*16 (0.0–0.5), *rpl*36 (0.0–0.9), *rps*11 (0.0–0.8), *rps*12 (0.0–0.3), *rps*19 (0.0–0.6), and *yc*f3 (0.0–0.5) (Figs A-AG in S4 File).

In contrast, some genes exhibited pairwise ratios that were equal or greater than 1.0. These included mitochondrial *atp*6 (0.0–1.5), *cob* (0.0–2.6), *cox*1 (0.0–3.4), *cox*2 (0.0–1.1), *nad*2 (0.0–1.8), *nad*3 (0.0–1.0), *nad*4 (0.0–1.2), *nad*5 (0.0–1.1), *nad*6 (0.0–1.5), and plastid *atp*E (0.0–1.4), *atp*F (0.0–2.2), *clp*P (0.0–1.9), *psb*F (0.0–2.2), *psb*J (0.0–19.4), *psb*Z (0.0–1.2), *rpl*20 (0.0–1.1), *rps*2 (0.0–2.5), *rps*3 (0.0–1.8), *rps*4 (0.0–1.4), *rps*7 (0.0–1.6), *rps*8 (0.0–1.9), *rps*14 (0.0–1.1), *rps*18 (0.0–1.6), and *ycf*12 (0.0–2.1) (Figs AH-BF in S4 File).

## Discussion

In this study, we examined whether individual organellar genes or concatenations of such genes provided sufficient information to resolve monophyletic relationships of known green algal class-level clades and investigated gene mode of evolution.

### Organellar trees

To establish a reference for downstream analyses, we first performed phylogenetic analyses of combined protein-coding sequences from mitochondrial and chloroplast genomes. Our results suggested monophyly of Chlorophyceae and monophyly of Trebouxiophyceae. Trebouxiophyceae was sister to a clade consisting of Chlorophyceae plus Ulvophyceae. Prasinophytes appeared as sister to a clade consisting of Chlorophyceae, Ulvophyceae, plus Trebouxiophyceae. When topologies of the organellar trees are compared to that of the combined tree we observed that the topology of the combined tree was more similar to that of the plastid tree. This higher degree of similarity might reflect the greater number of plastid sequences as they contributed as much as 71.99 percent of the combined data matrix (30,803 from 42,785 nucleotide positions).

One notable difference between the mitochondrial tree and the chloroplast tree was the position of the prasinophytes. In the chloroplast tree and in the tree based on combined chloroplast and mitochondrial sequence data, the prasinophytes formed a clade sister to a clade consisting of chlorophyte algae. By contrast, prasinophytes were paraphyletic in the mitochondrial tree. This difference has been noted in previous studies (e.g. Satjarak et al., 2017) [20], suggesting the possibility that prasinophycean chloroplast genes might have experienced different selective forces than those of mitochondrial genomes. However, the relationships present in concatenated trees were not congruent with the relationship resolved from the single-protein analyses. Instead, we observed mixed patterns of relationship between prasinophycean protein-coding sequences and those of non-prasinophycean chlorophytes, as prasinophycean sequences appeared to be closely related to most of the known algal groups (Figs A-BF in S3 File). These observations might help to explain why it is still challenging to resolve the identity of the modern lineage that most closely resembled the last common ancestor of Viridiplantae.

Interestingly, Trebouxiophyceae also exhibited differences in phylogenetic position in organellar trees. In combined and plastid trees, Trebouxiophyceae was placed in a clade together with Chlorophyceae and Ulvophyceae. However, in mitochondrial trees, Trebouxiophyceae was placed in a clade with streptophyte algae and prasinophytes. This incongruency was similarly revealed by previously studies [4,21,22].

Another interesting observation arising from the comparison between the combined tree and individual organellar trees was the inconsistency of the presence of monophyly of Ulvophyceae. The monophyly of Ulvophyceae was not present in the combined tree, plastid tree, nor mitochondrial tree. This result was unexpected, as when single-gene trees were considered, the monophyly of Ulvophyceae was present in mitochondrial *nad*5 data from plastid *atp*A, *atp*H, *atp*E, *psb*C, *psb*D, and *ycf*3, which contributed as much as 16.60 percent of the combined data matrix (7,104 from 42,785 nucleotide positions).

Trees resolved using concatenated data of plastid ribosomal rDNA–rrnL and rrs–similarly exhibited monophyly of Chlorophyceae, Trebouxiophyceae, and Ulvophyceae (Figs BG in S3 File). However, Chlorophyceae was sister to the clade consisted of Trebouxiophyceae and Ulvophyceae suggesting that the evolutionary patterns of the core chlorophytes protein-coding genes differed from that of their plastid rDNAs.

### Trees from genes encoding proteins for known biological functions

We found that all gene groups–mitochondrial NADH dehydrogenase, cytochrome c oxidase, ATP synthase, plastid ATP synthase, ribosomal proteins, PSI subunits, PSII subunits, and cytochrome b6f complex subunits–resolved the monophyly of Chlorophyceae. The monophyly of Chlorophyceae in these trees was congruent with the results of single-gene estimations, where most of the genes resolved the monophyly of Chlorophyceae. Therefore, for the selected taxa belonging to Chlorophyceae, not only genes encoding for proteins involved in specific biological functions, but potentially a number of organellar genes provided sufficient information to resolve the monophyly of Chlorophyceae.

Despite evidence for monophyly of Chlorophyceae, trees resolved from protein-coding genes exhibited variations in relationships among chlorophyte algae. Mitochondrial NADH dehydrogenase (complex I) subunits, cytochrome c oxidase, mitochondrial ATP synthase (complex V) subunits, and plastid genes encoding for photosystem II subunits, and cytochrome b6f complex subunits resolved the clade consisting of Trebouxiophyceae that was sister to the clade consisting of Chlorophyceae and Ulvophyceae, while plastid genes encoding for ATP synthase subunits, small ribosomal proteins subunits, and photosystem I subunits resolved the tree in which the clade of Chlorophyceae was sister to the clade consisting of Trebouxiophyceae and Ulvophyceae. This incongruency of tree topology implied that these organellar genes had not been subjected to the same evolutionary direction or selection pressure.

### Single-gene analyses and dN/dS ratio estimates

Differences in the average values of dN/dS ratios (ranging from 0.00–3.41 for mitochondrial genes and 0.00–2.54 for plastid genes) suggested that in general chlorophyte mitochondrial genes have evolved at a higher rate than have chloroplast genes. It might be possible that mitochondrial genes accumulated higher ratios of non-synonymous mutations because this organelle was acquired prior to the acquisition of plastids in Viridiplantae. However, more study is still need in order to fully understand the presence of this scenario.

Species known to be closely related showed lower dN/dS ratios and species more distantly related showed higher dN/dS ratios. Among the chlorophyte taxa studied *Coccomyxa subellipsoidea* showed particularly low pairwise dN/dS ratios. These comparatively low dN/dS ratios were exhibited in the heatmap of all genes except mitochondrial *atp*6, *cob*, *cox1* and *nad*5 and plastid *atp*H, *pet*G, *psb*A, *psb*E, *psb*H, *psb*I, *psb*J, *psbL*, *rps*7, *rps*8 and *ycf*12. The presence of these “lower” ratios might have resulted from increase in synonymous substitution rate in *C*. *subellipsoidea* by GC biased gene conversion[8] or codon usage bias.

### Genes resolving the monophyly of Chlorophyceae

Among known algal clades, the monophyly of Chlorophyceae appeared in most of the single-gene trees–mitochondrial *atp*6, *atp*9, *cox*2, *cox*3, *nad*1, *nad*2, *nad*3, *nad*4, *nad*4L, *nad*5, and *nad*6 and plastid *atp*A, *atp*B, *atp*E, *atp*F, *clp*P, *pet*A, *psa*A, *psa*B, *psa*C, *psb*B, *psb*C, *psb*D, *psb*E, *psb*H, *psb*N, *rpl*2, *rpl*5, *rpl*14, *rpl*16, *rpl*20, *rps*2, *rps*3, *rps*4, *rps*7, *rps*8, *rps*11, *rps*12, *rps*14, *rps*18, and *rps*19. When their pairwise dN/dS ratios were mapped to the data matrices using their phylogenetic position resolved in the corresponding single-gene tree, we observed a correlation between gene power to infer monophyly for Chlorophyceae and low dN/dS ratio. In the heatmap, the dN/dS ratios for chlorophycean algae were comparatively low when compared to that of between chlorophycean and non-chlorophycean alga or that of between non-chlorophycean algae. This pattern was present in the heatmaps of mitochondrial *atp*6, *atp*9, *cox*2, *cox*3, *nad*1, *nad*2, *nad*3, *nad*4, *nad*4L, *nad*5, and *nad*6 and plastid *atp*A, *atp*B, *atp*E, *atp*F, *clp*P, *psa*A, *psa*B, *psa*C, *psb*B, *psb*C, *psb*D, *psb*E, *psb*N, *rpl*2, *rpl*5, *rpl*14, *rpl*16, *rpl*20, *rps*2, *rps*3, *rps*4, *rps*7, *rps*8, *rps*11, *rps*12, *rps*14, *rps*18, and *rps*19. The presence of these lower dN/dS ratios suggested that a higher degree of purifying selection had acted on these Chlorophyceae genes, indicating that the power of these these genes to resolve Chlorophyceae monophyly is based upon their sequence conservation.

However, a similar pattern of relationship between conservation (indicated by low dN/dS ratio) and resolution power was not observed for some other genes that likewise resolved Chlorophyceae monophyly, namely *pet*A, *psb*D, *psb*H, *psb*N, and *rpl*5. Similar dN/dS ratios suggested that *pet*A, *psb*D, *psb*H, *psb*N, and *rpl*5 of species studied had been subjected to a similar degree of purifying selection, which might reflect gene function. For instance, *pet*A encodes for cytochrome f precursor which plays an important role in electron transport from PSII to PSI[23], and *psb*D and *psb*H are PSII subunits that function as P680 binding protein or PSII reaction center binding protein and in PSII assembly[24,25].

### Genes resolving the monophyly of Trebouxiophyceae

The monophyly of Trebouxiophyceae was resolved in trees estimated using mitochondrial *nad*2. This inference of monophyly is congruent with the presence of a cluster of low dN/dS ratios for trebouxiophycean *nad*2, indicating that purifying selection had acted on this trebouxiophycean gene.

### Genes resolving the monophyly of Ulvophyceae

The monophyly of Ulvophyceae was resolved in single-gene analyses of mitochondrial *nad*5 and plastid *atp*A, *atp*H, *atp*E, *psb*C, *psb*D, and *ycf*3. Strong purifying selection at the intra-genus level was indicated by low dN/dS ratios for these genes from *Ulva* and *Gloeotilopsis*.

Interestingly, we observed that some genes that have widely been used for phylogenetic estimations might not be as informative as expected. For example, mitochondrial *cob*, *cox*1, and plastid *rbc*L, which have been extensively used in phylogenetic studies did not resolve monophyly of known clades [26,27]. The absence of evidence for monophyly provided by such genes explored in this study suggests that these particular sequences might not be sufficiently informative for single-gene phylogenetic analyses. In contrast to the absence of known clades in the trees resolved from the classical DNA markers, our single-gene analyses suggested that some other organellar genes might be more beneficial in green algal phylogeny. For example, *nad*2, *nad*5, *atp*A, *atp*E, *psb*C, and *psb*D, single-gene analyses resolved the monophyly of more than one known class-level algal group. We propose that these genes might be good candidates to use as DNA markers for algal taxonomic analyses.

### Fast-evolving genes in Chlorophyceae

**Fig 2:**
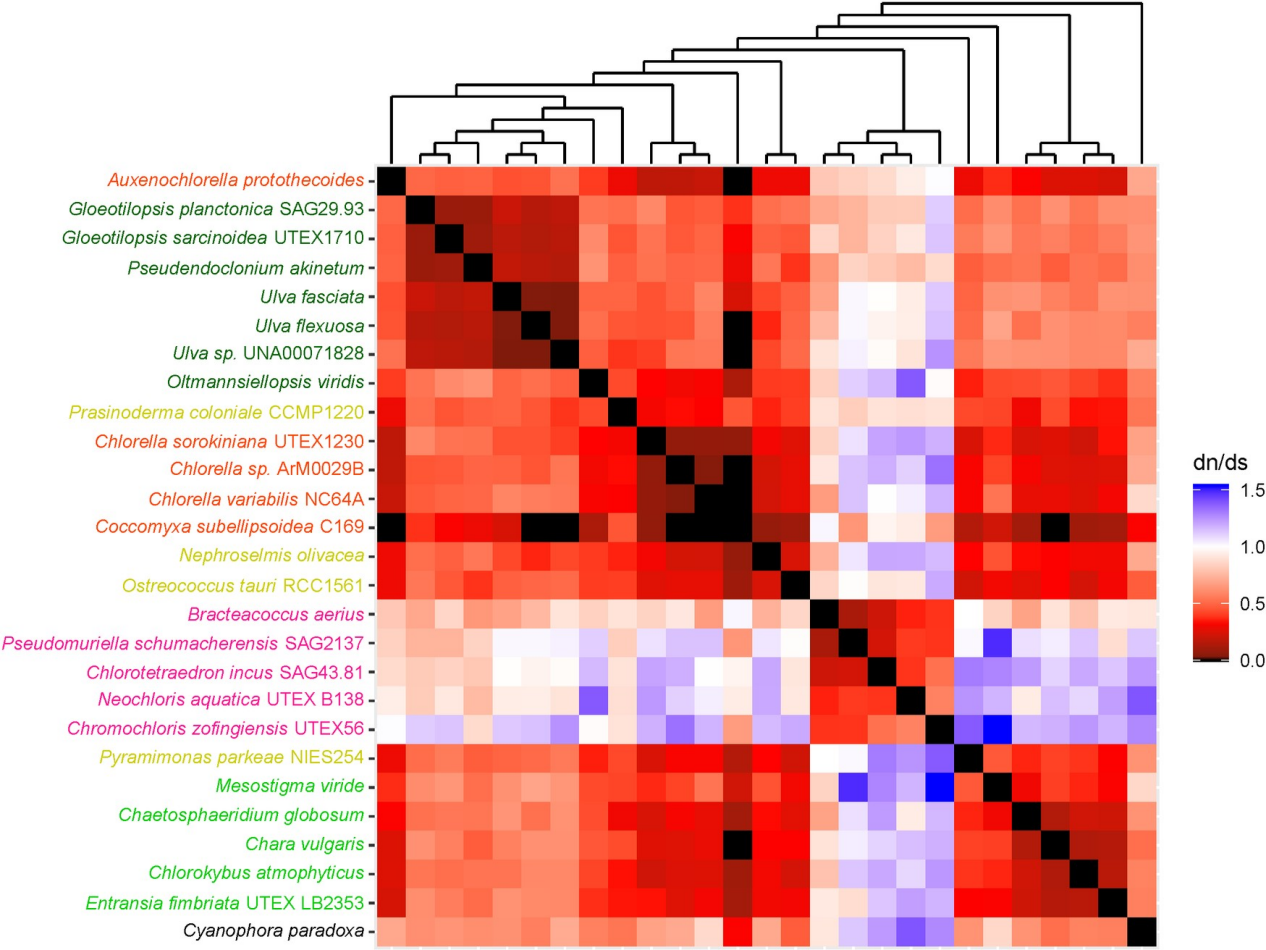
Heatmap of pairwise dN/dSdn/ds ratios calculated from alignment of mitochondrial *atp*6. The scale bar showed the color corresponded to dN/dS ratios. The cladogram above the heatmap corresponds to its single-gene phylogenetic tree.

**Fig 3:**
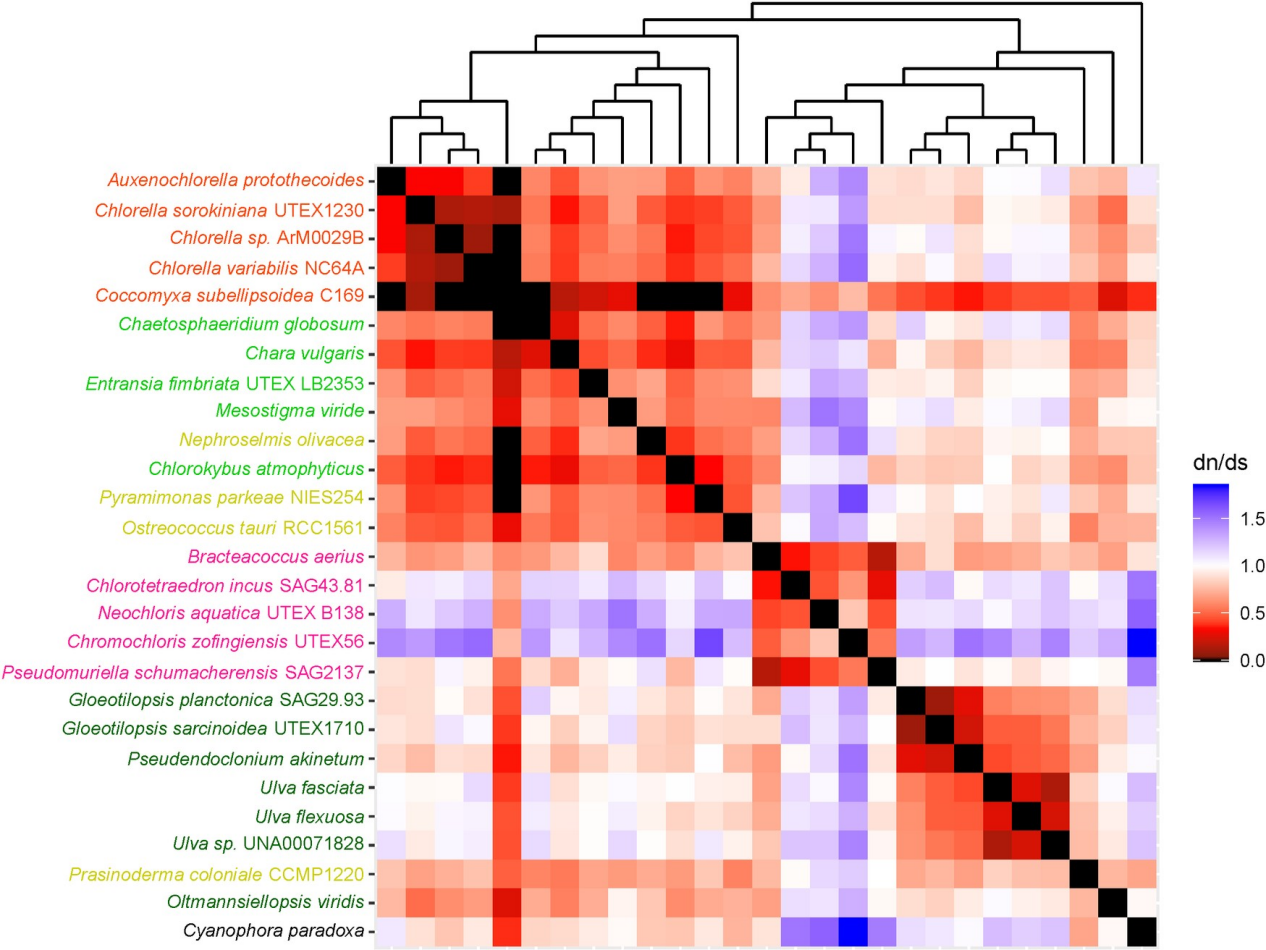
Heatmap of pairwise dN/dSdn/ds ratios calculated from alignment of mitochondrial *nad*2. The scale bar represents color corresponded to pairwise dN/dS ratios. The cladogram above the heatmap corresponds to phylogenetic relationship estimated using algal *nad*2.

**Fig 4:**
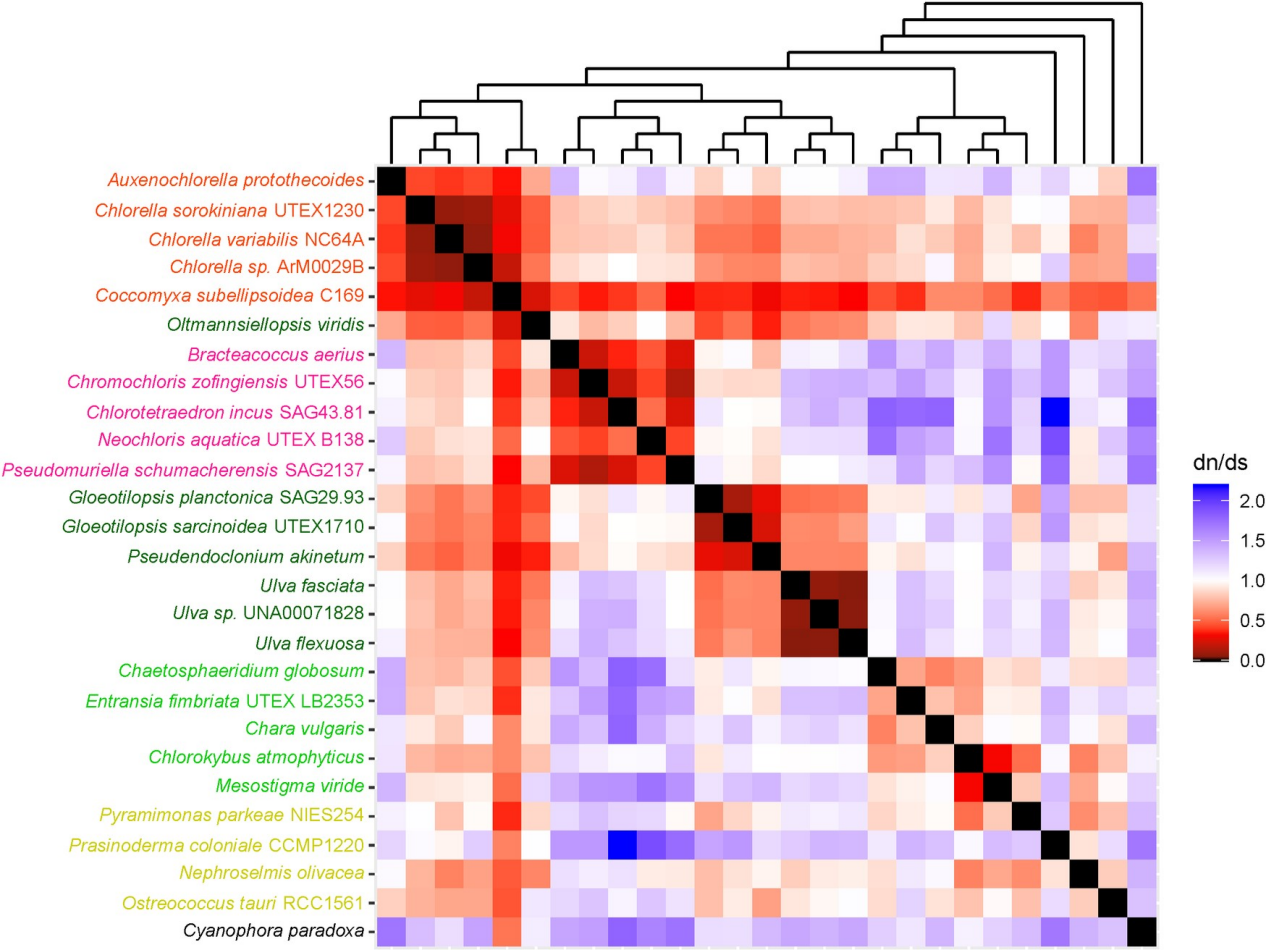
Heatmap of pairwise dN/dSdn/ds ratios calculated from alignment of plastid *atp*F. The scale bar represents color corresponded to pairwise dN/dS ratios. The cladogram above the heatmap corresponds to phylogenetic relationship estimated using algal *atp*F.

**Fig 5:**
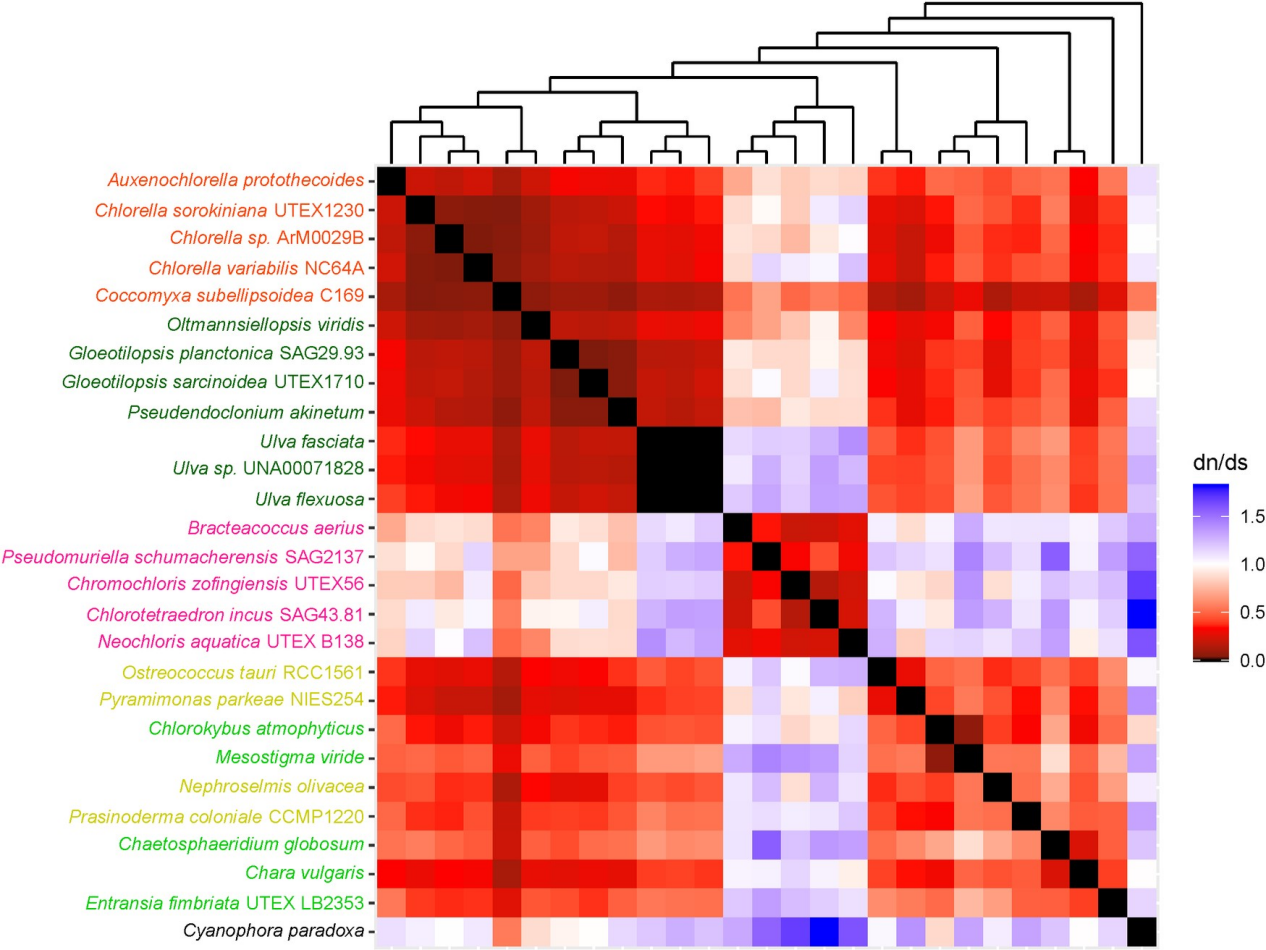
Heatmap of pairwise dN/dSdn/ds ratios calculated from alignment of plastid. The scale bar represents color corresponded to pairwise dN/dS ratios. The cladogram above the heatmap corresponds to phylogenetic relationship estimated using algal *clp*P.

**Fig 6:**
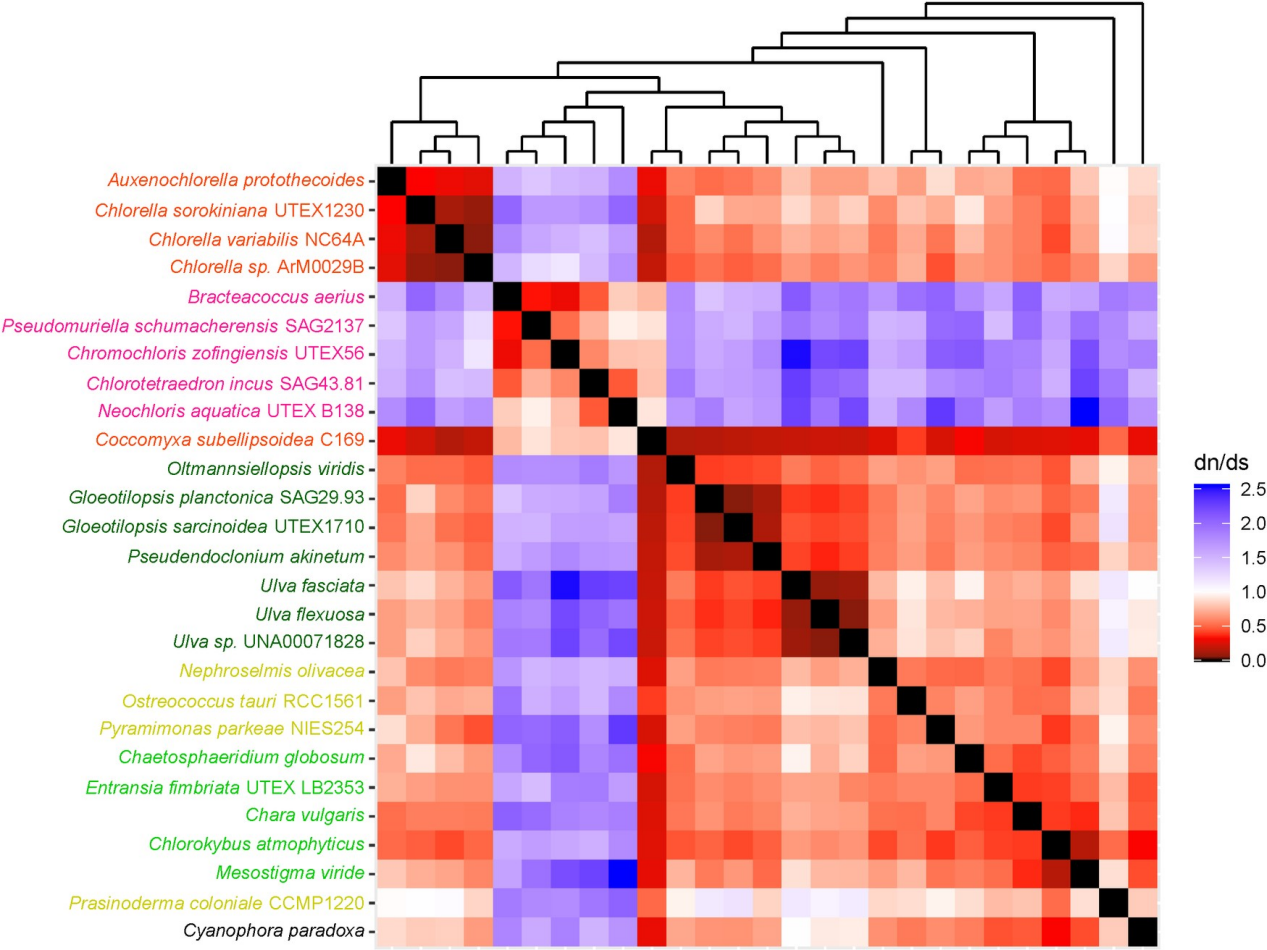
Heatmap of pairwise dN/dSdn/ds ratios calculated from alignment of plastid. The scale bar represents color corresponding to pairwise dN/dS ratios. The cladogram above the heatmap corresponds to phylogenetic relationship estimated using algal *rps*2.

**Fig 7:**
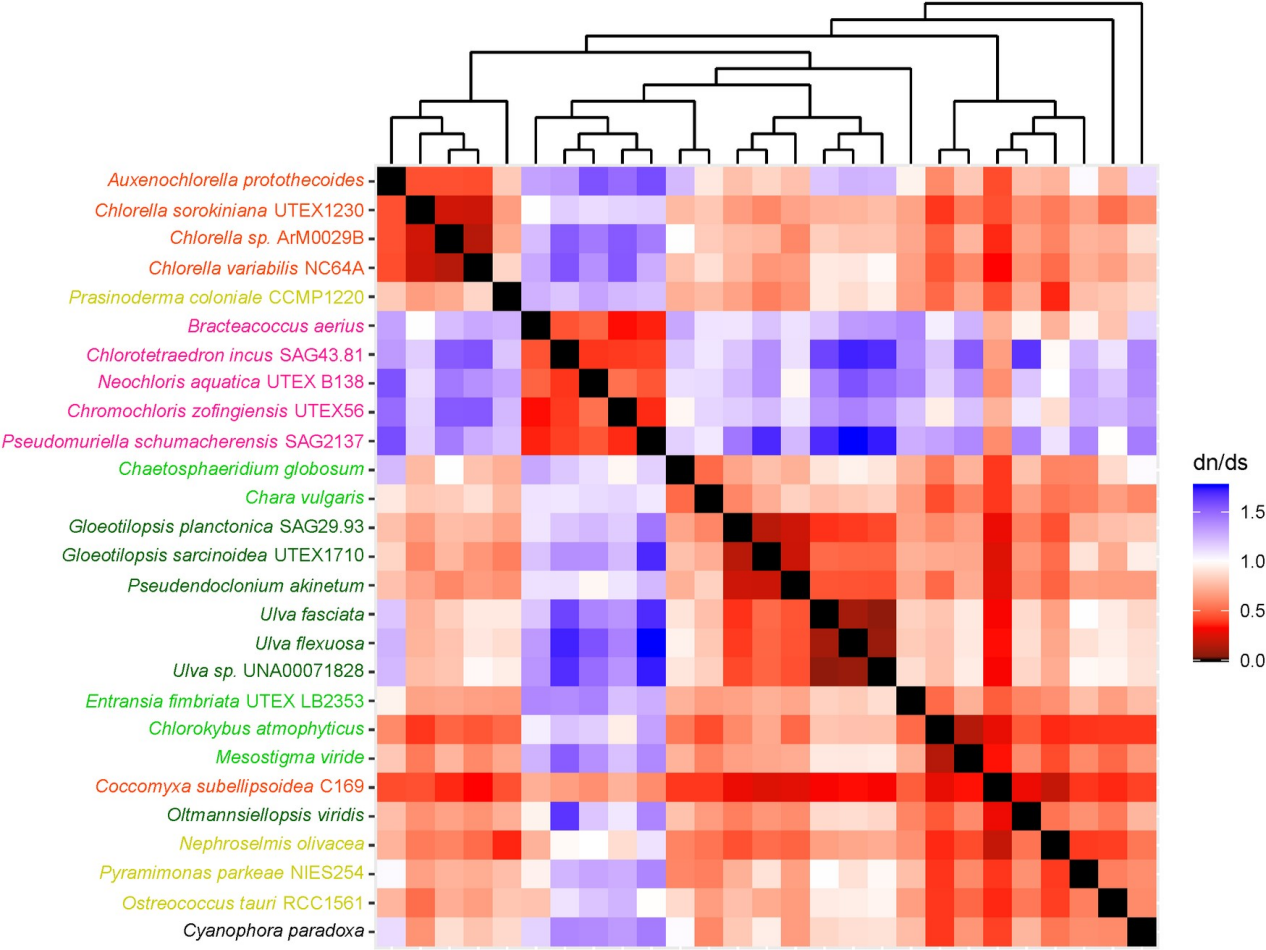
Heatmap of pairwise dN/dSdn/ds ratios calculated from alignment of plastid *rps*3. The scale bar represents color corresponding to pairwise dN/dS ratios. The cladogram above the heatmap corresponds to phylogenetic relationship estimated using algal *rps*3.

**Fig 8:**
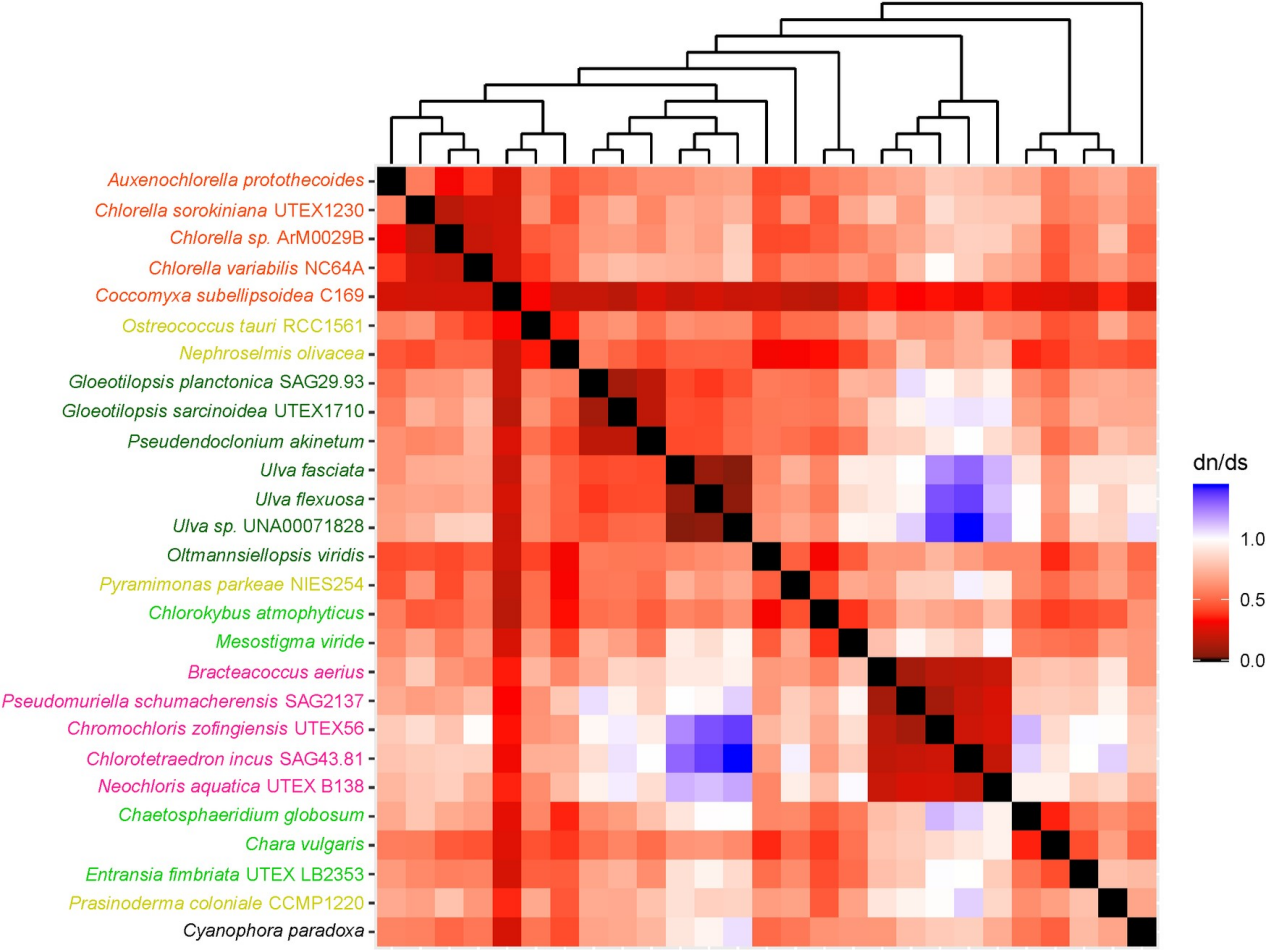
Heatmap of pairwise dN/dSdn/ds ratios calculated from alignment of plastid *rps*4. The scale bar represents color corresponding to pairwise dN/dS ratios. The cladogram above the heatmap corresponds to phylogenetic relationship estimated using algal *rps*4.

**Fig 9:**
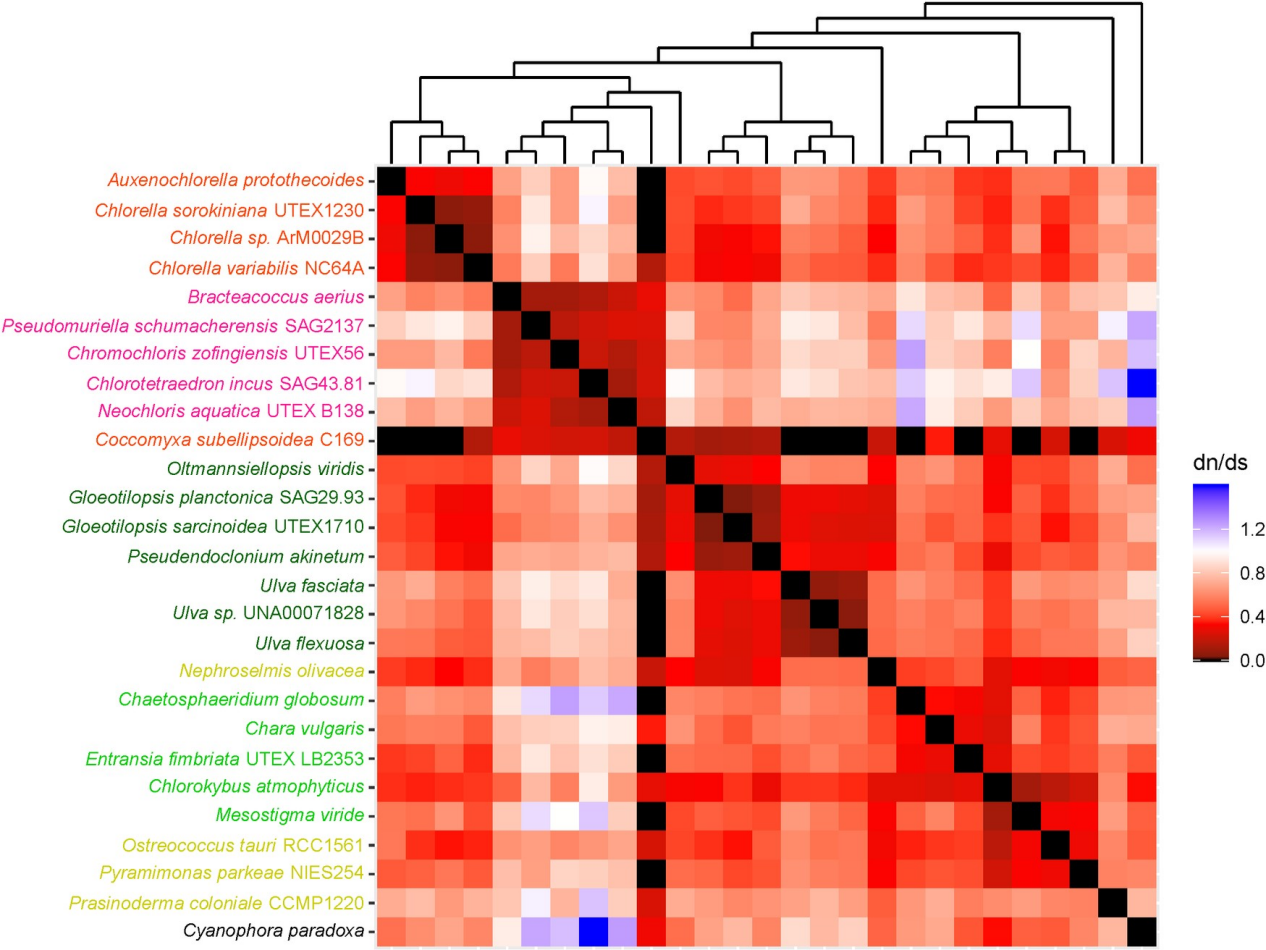
Heatmap of pairwise dN/dSdn/ds ratios calculated from alignment of plastid *rps*7. The scale bar represents color corresponding to pairwise dN/dS ratios. The cladogram above the heatmap corresponds to phylogenetic relationship estimated using algal *rps*7.

The heatmaps used in this study helped us to understand long branches occurring in Chlorophyceae trees. These long branches were observed in single-gene phylogenetic estimations using mitochondrial *atp*6, *nad*2, *atp*F, *clp*P, *rps*2, *rps*3, *rps*4, and *rps*7 (Figs 2–9). Mapping dN/dS ratios to phylogenetic relationship indicated types of mutations that had occurred in these genes. We found that the presence of these long branches correlated with contrasting values of dN/dS ratios between taxa belonging to Chlorophyceae (dN/dS <1) and between Chlorophyceae and non-Chlorophyceae (dN/dS >1 or ~ 1 in most pairs). The co-occurrence of long branches and contrasting values of dN/dS ratios suggested that these genes might have been subjected to selective mutation that increased fitness in Chlorophyceae.

The divergence of mitochondrial *atp*6 in Chlorophyceae from that of other algal taxa correlated with change in structure of chlorophycean ATP synthase complex (Fig 2). A previous study had showed that original subunits d, e, and f of chlorophycean mitochondrial ATP synthase complex might have been substituted by proteins of unknown origin, mitochondrial F1F0 ATP synthase associated proteins (Asa) because oligomycin, a chemical that would normally inhibit ATP synthase activity by blocking proton flow through F0 complex, did not inhibit the activity of ATP synthase in *Chlamydomonas reinharditii* where the subunit was substituted by Asa7 protein[28]. The selective mutation in *atp*6 reported in this study might correlate with oligomycin resistance conferred by Asa7. To test whether the Asa7 forms a complex with subunit a of F0 complex to confer oligomycin resistance, and whether structural changes in chlorophycean atp6 protein accommodate or interact with Asa proteins, further structural analysis will be needed.

Chlorophyceae plastid *atp*F also exhibited divergence from other *atp*F we studied (Fig 3). This divergence might correlate with change in structure of chlorophycean chloroplast ATP synthase complex, since previous analysis showed that *C*. *reinhardtii* chloroplast ATP synthase occurred as dimers formed by ATP synthase subunit b, encoded by the *atp*F gene[29]. It would be interesting to know if this dimerization also occurs in other chlorophycean species used in this study.

Another protein-coding gene exhibiting selective mutation in this study was *nad*2 (Fig 4), a gene encoding a central subunit of the NADH synthase membrane arm that functions in the Na^+^/H^+^ antiporter of NADH synthase complex[30]. The selective mutation observed in these three genes might alter the activity of the Na+/H+ antiporter.

The divergence of *clp*P might be a result of difference in sequence length (Fig 5). The length of chlorophycean *clp*P was approximately 1.5 kb while that of non-chlorophycean *clp*P ranged from 0.5 to 0.6 kb. The increase of chlorophycean *clp*P might have resulted from the insertion of self-splicing large sequence having no known function, as reported for *clp*P sequences of *Chlamydomonas reinhardtii* [31] and *Chlamydomonas eugametos*[32]. However, additional sequence data for chlorophycean *clp*P are needed to test whether the presence of a large self-splicing sequence is a clade-specific character of chlorophycean *clp*P.

Other chlorophycean genes that also exhibited selective mutation included *rps*2, *rps*3, *rps*4 and *rps*7 (Fig 6–9). These genes encode proteins for 30S ribosomal subunits functioning in mRNA binding during transcription initiation, 16S rRNA stabilization, and assembly of 30S ribosomal subunit[33,34]. It would be interesting to determine if the presence of this selective mutation alters activity of these processes and plays an important role in diversification of Chlorophyceae.

## Conclusion

We report the effect of using protein-coding regions in organelle, biological function, and single-gene analyses on phylogenetic inference for chlorophyte algae. By comparing the results of phylogenetic estimation using these different data sets, we observed diverse patterns of evolution. Mitochondrial genomes seem to have evolved at a higher rate than do chlorophyte chloroplast genomes. Interestingly, mitochondrial and chloroplast genes of Trebouxiophyceae exhibit different patterns of evolution direction, indicated by our observation that trebouxiophycean mitochondrial and chloroplast genes appeared to be more closely related to those of streptophyte algae than to chlorophyte algae. We examined the informative level of organelle genes by comparing their ability to resolve known chlorophyte clades, observing that single-gene phylogenetic inferences using *nad*2, *nad*5, *atp*A, *atp*E, *psb*C, and *psb*D resolved the monophyly of at least two known chlorophyte clades. These results suggest that these particular genes might form a recommended gene set for broad taxonomic sampling or for preliminary analyses. By comparing pairwise ratios of non-synonymous and synonymous substitution rates, we observed a level of contrasting value (>1) between chlorophycean and non-chlorophycean *atp*6, *nad*2, *atp*F, *clp*P, *rps*2, *rps*3, *rps*4, and *rps*7, which represents the presence of selective mutations that have accumulated in these protein-coding genes.

## Supporting information

S1 Table Alignment length and nucleotide substitution model used for phylogenetic analysis of concatenated sequences of each gene family. (XLSX)

S2 Table Alignment length and nucleotide substitution model used for phylogenetic analysis of sequence of each gene. (XLSX)

S3 Table Monophyly of known clades resolved from phylogenetic estimations. Bootstrap value cut-off is ≥ 80 in Maximum Likelihood framework or ≥ 0.8 in Bayesian Inference.
(XLSX)

S1 File Phylogenetic trees constructed from concatenated mitochondrial gene sequence and concatenated plastid gene sequence. (PDF)

S2 File Phylogenetic trees constructed from concatenated sequences of each gene family. (PDF)

S3 File Single gene phylogenetic trees of genes in this study. (PDF)

S4 File Heatmaps of pairwise dN/dS ratios of genes in this study. (PDF)
