# Supplementary material for: Evolution of organellar genes of chlorophyte algae: Relevance to phylogenetic inference
Source: PLoS One. 2019 May 6;14(5):e0216608. doi: 10.1371/journal.pone.0216608 (PMC6502327; doi:10.1371/journal.pone.0216608)
Supplement: S1 File — (PDF) [file pone.0216608.s004.pdf]

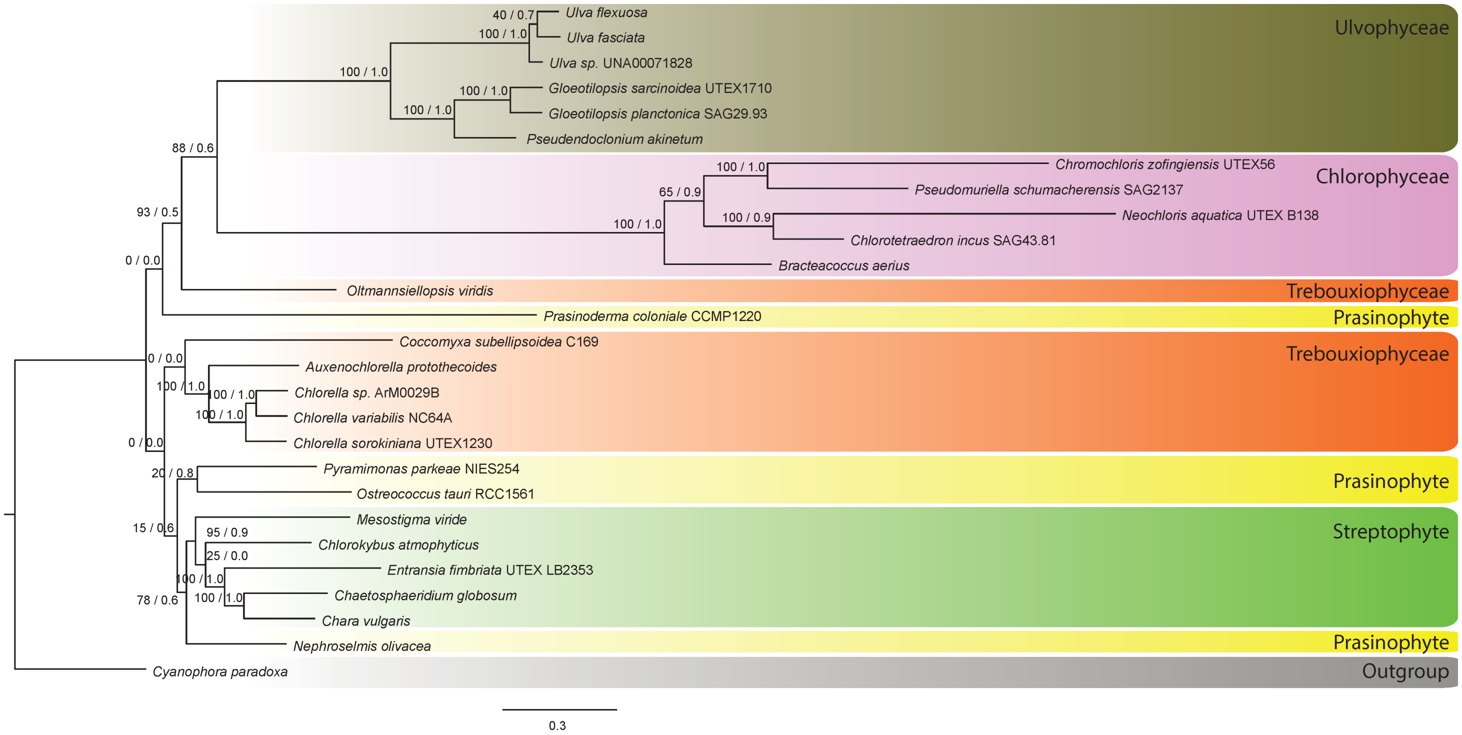

S1 Fig. A. Maximum-Likelihood tree inferred from 13 mitochondrial protein-coding sequences of algal taxa in Table 1 using a GTR+Γ+I nucleotide substitution model. Bootstrap value from ML and posterior probability from Bayesian inference are shown at the node. The scale bar represents the estimated number of nucleotide substitution per site.

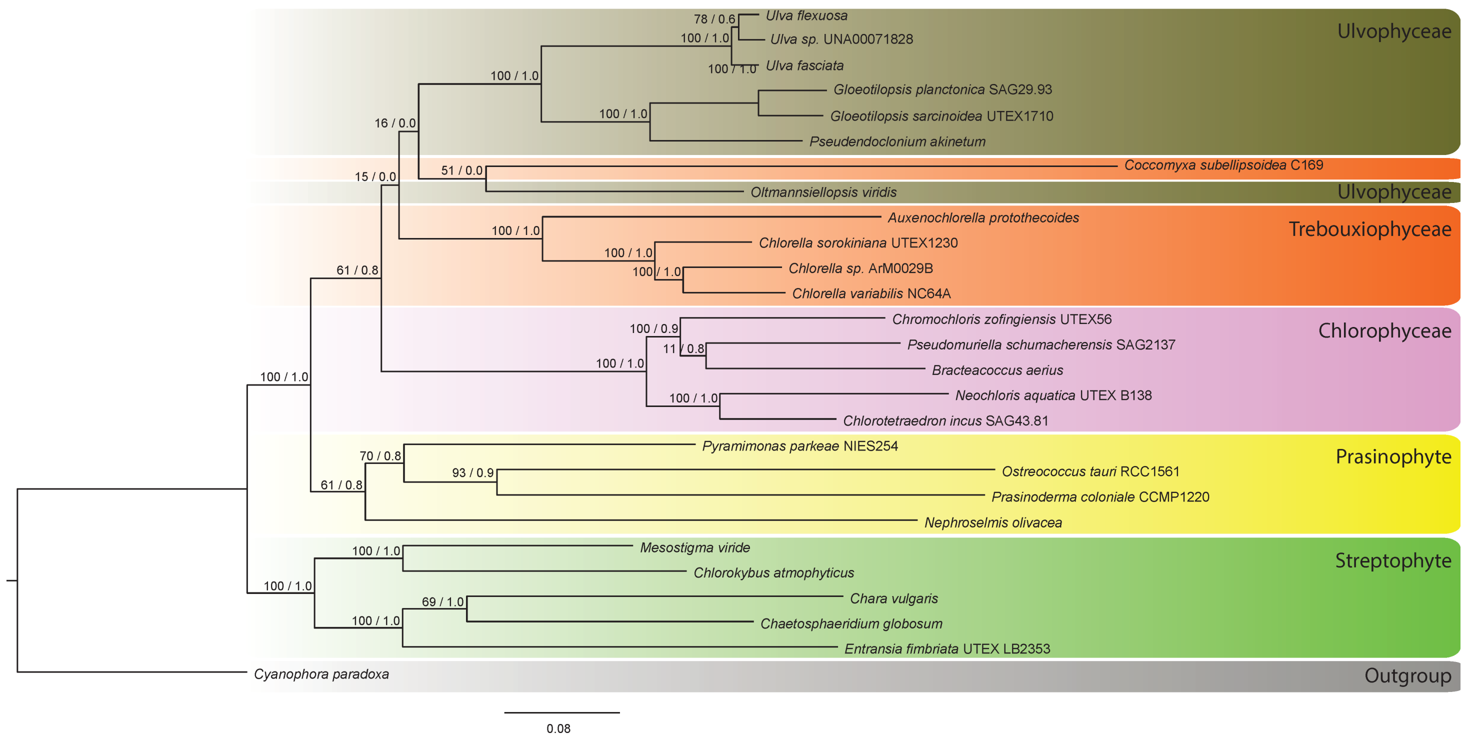

S1 Fig. B. Maximum-Likelihood tree inferred from 45 plastid protein-coding sequences of algal taxa in Table 1 using a GTR+Γ+I nucleotide substitution model. Bootstrap value from ML and posterior probability from Bayesian inference are shown at the node. The scale bar represents the estimated number of nucleotide substitution per site.
