# Supplementary material for: Evolution of organellar genes of chlorophyte algae: Relevance to phylogenetic inference
Source: PLoS One. 2019 May 6;14(5):e0216608. doi: 10.1371/journal.pone.0216608 (PMC6502327; doi:10.1371/journal.pone.0216608)
Supplement: S2 File — (PDF) [file pone.0216608.s005.pdf]

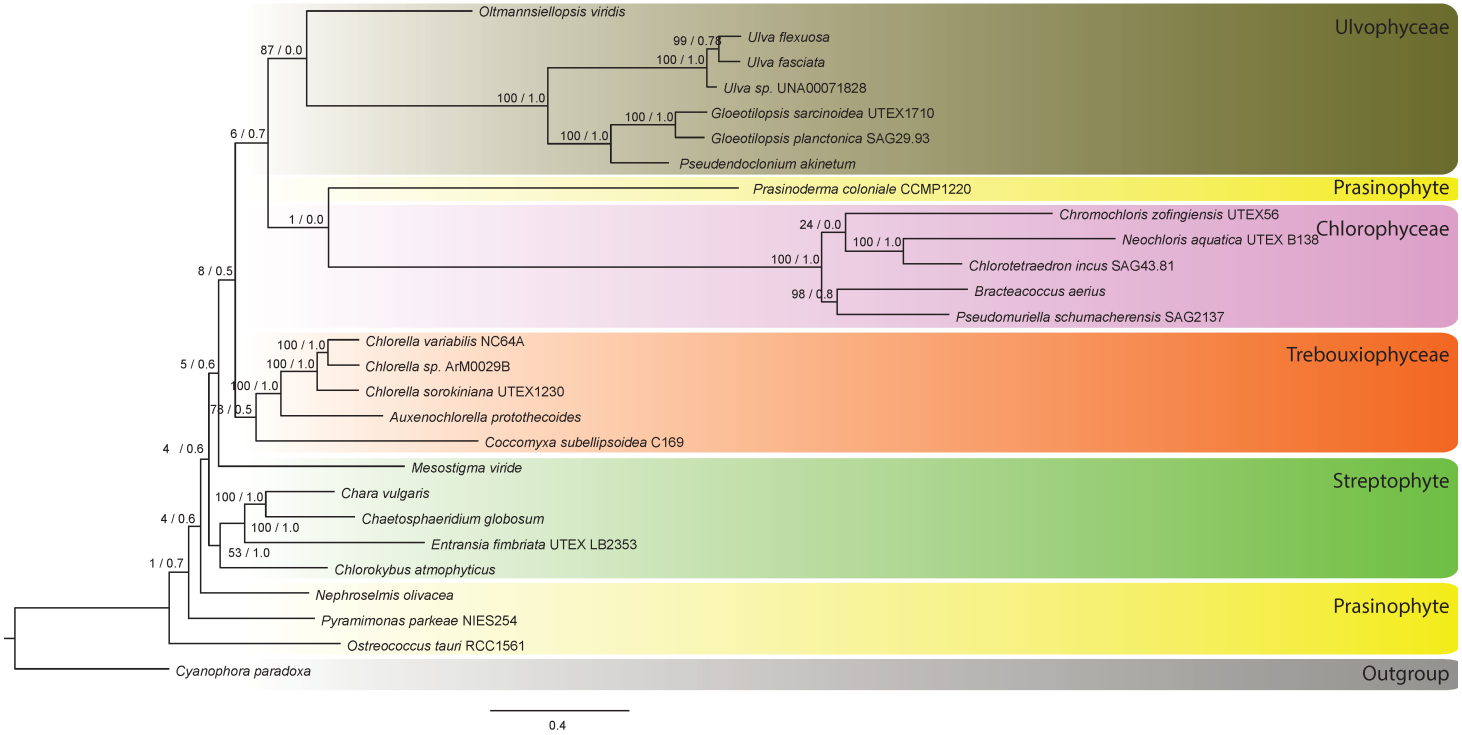

S2 Fig. A. Maximum-Likelihood tree inferred from mitochondrial protein-coding sequences encode for NADH dehydrogenase subunits of algal taxa in Table 1 using a GTR+Γ+I nucleotide substitution model. Bootstrap value from ML and posterior probability from Bayesian inference are shown at the node. The scale bar represents the estimated number of nucleotide substitution per site.

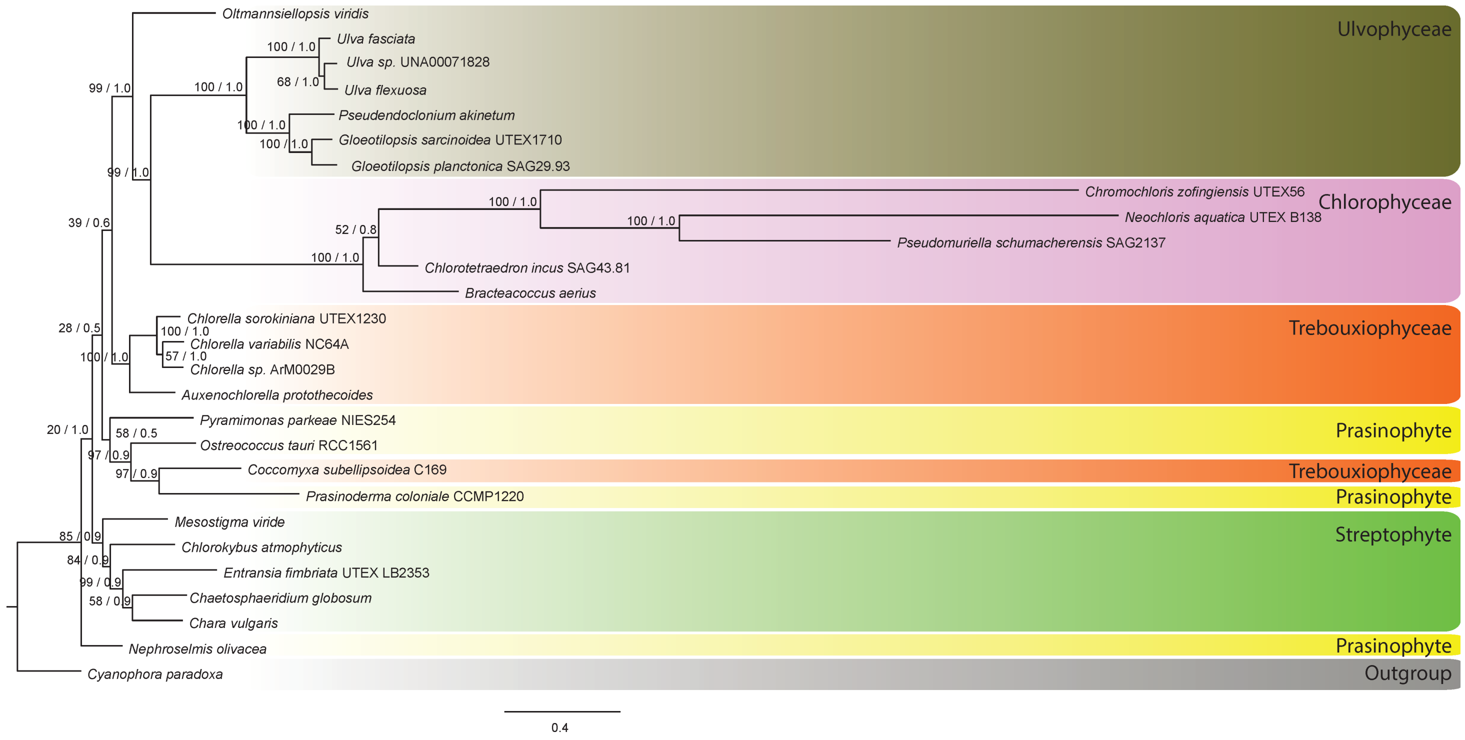

S2 Fig. B. Maximum-Likelihood tree inferred from mitochondrial protein-coding sequences encode for cytochrome c oxidase (complex IV) subunits of algal taxa in Table 1 using a GTR+Γ+I nucleotide substitution model. Bootstrap value from ML and posterior probability from Bayesian inference are shown at the node. The scale bar represents the estimated number of nucleotide substitution per site.

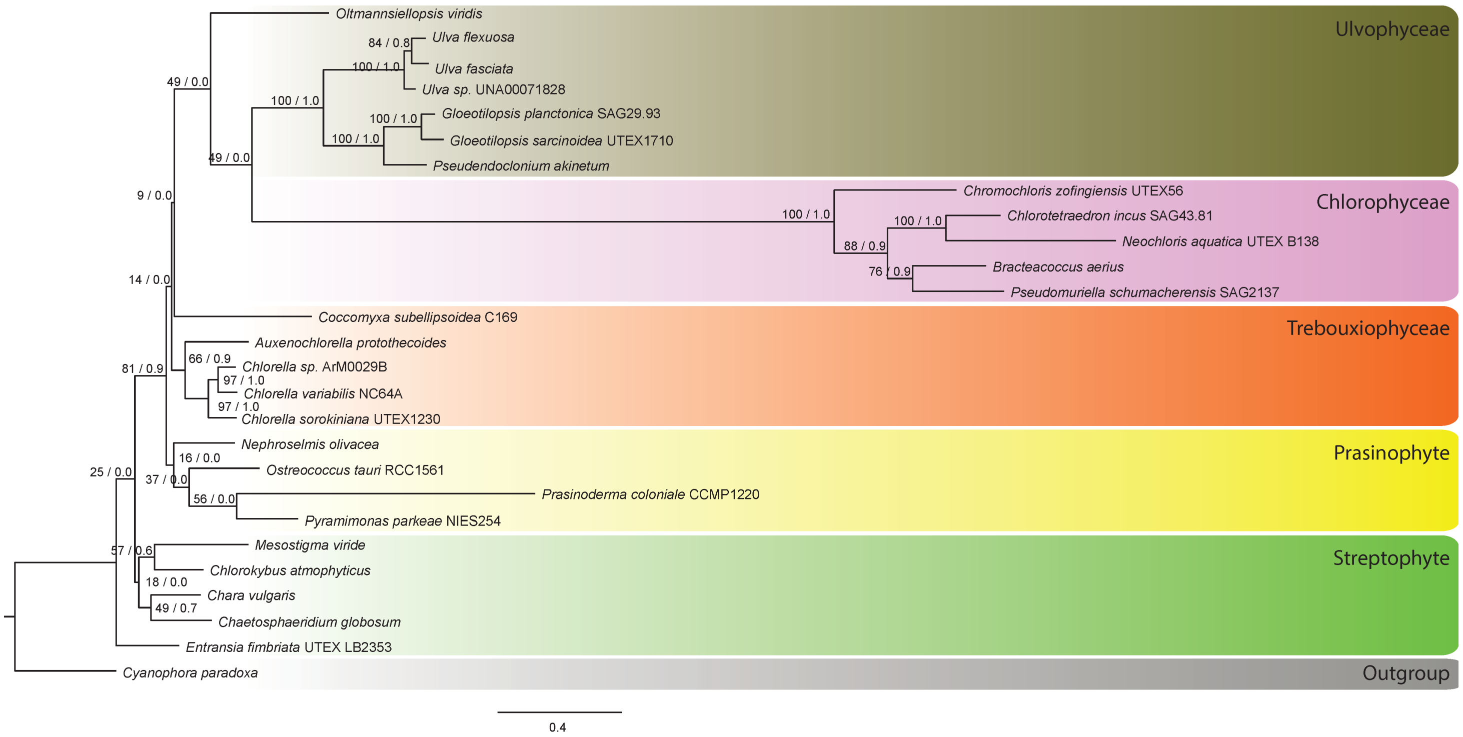

S2 Fig. C. Maximum-Likelihood tree inferred from mitochondrial protein-coding sequences encode for ATP synthase (complex V) subunits of algal taxa in Table 1 using a GTR+Γ+I nucleotide substitution model. Bootstrap value from ML and posterior probability from Bayesian inference are shown at the node. The scale bar represents the estimated number of nucleotide substitution per site.

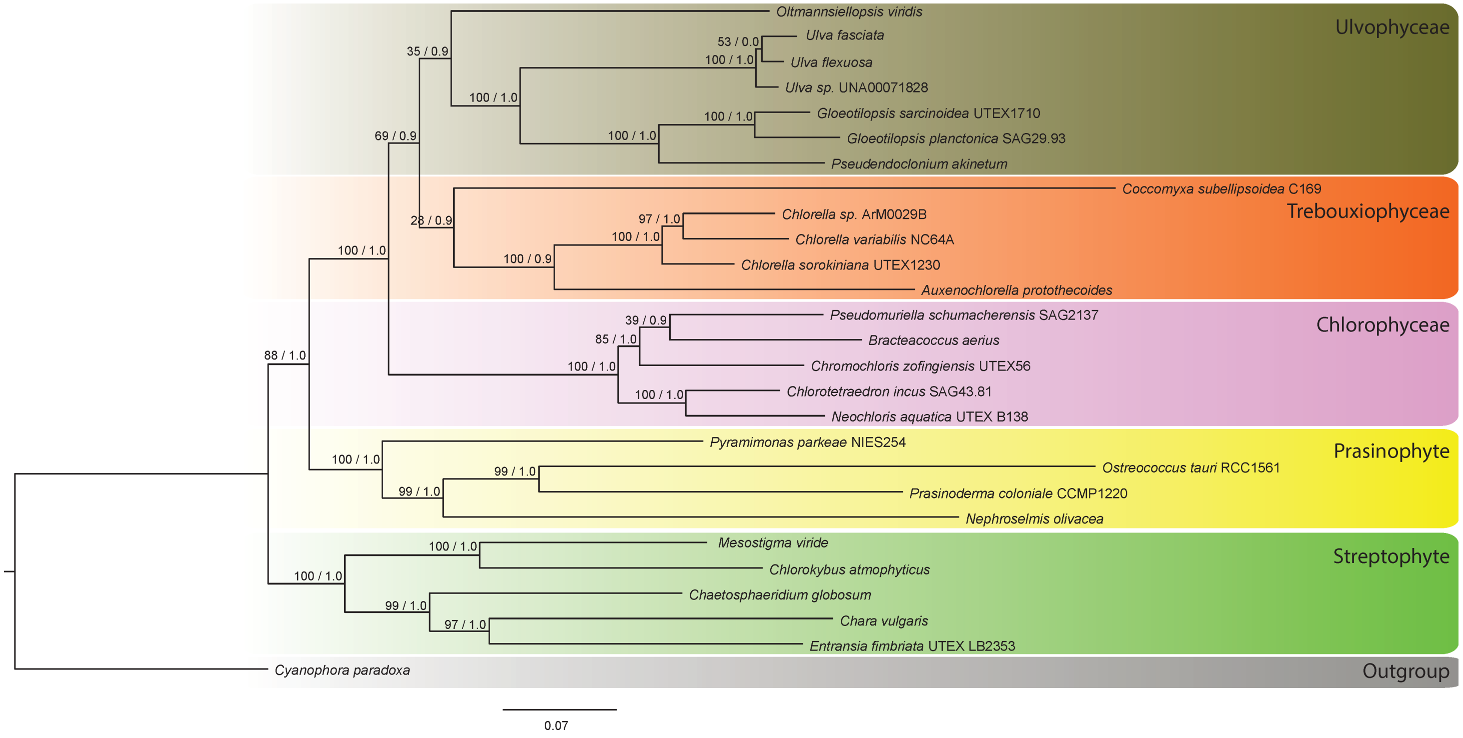

S2 Fig. D. Maximum-Likelihood tree inferred from plastid protein-coding sequences encode for ATP synthase subunits of algal taxa in Table 1 using a GTR+Γ+I nucleotide substitution model. Bootstrap value from ML and posterior probability from Bayesian inference are shown at the node. The scale bar represents the estimated number of nucleotide substitution per site.

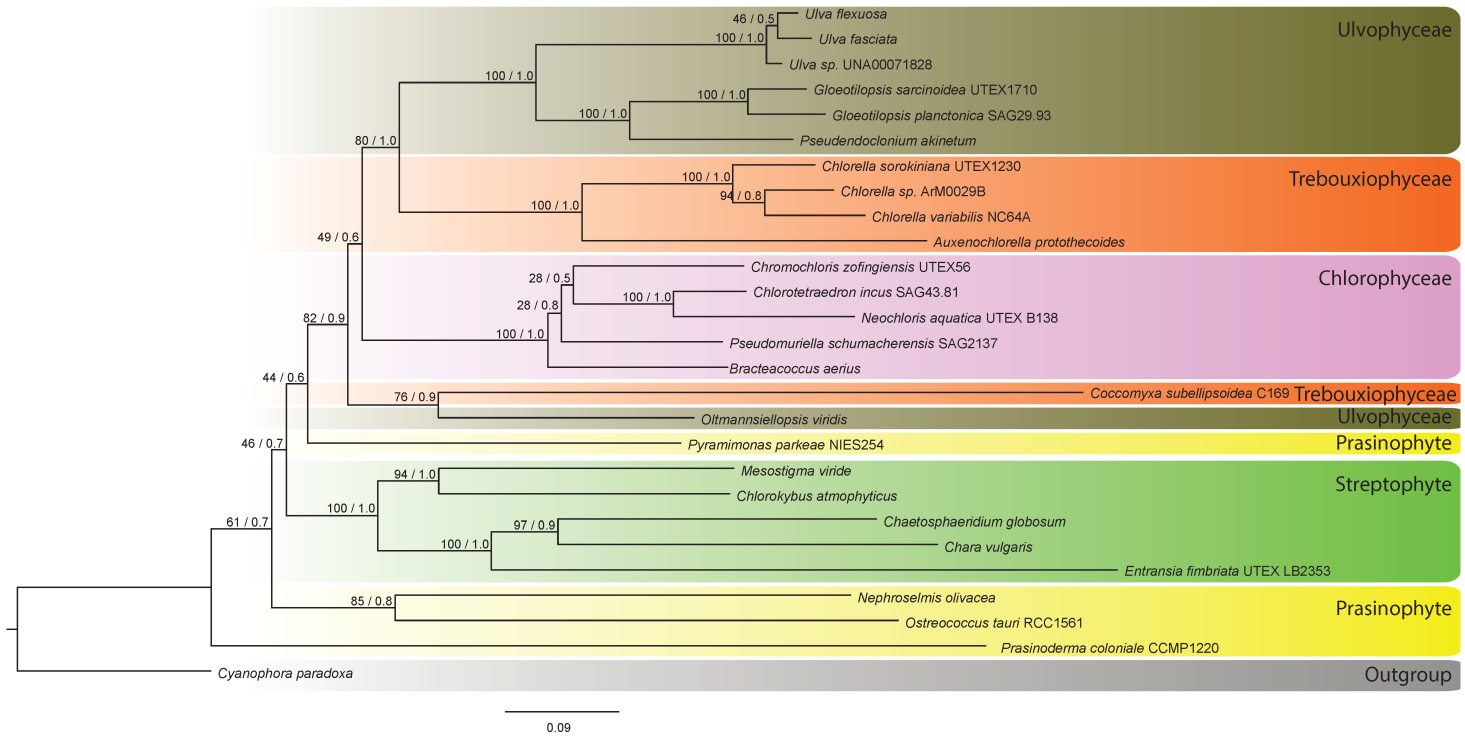

S2 Fig. E. Maximum-Likelihood tree inferred from plastid protein-coding sequences encode for ribosomal protein L of algal taxa in Table 1 using a GTR+Γ+I nucleotide substitution model. Bootstrap value from ML and posterior probability from Bayesian inference are shown at the node. The scale bar represents the estimated number of nucleotide substitution per site.

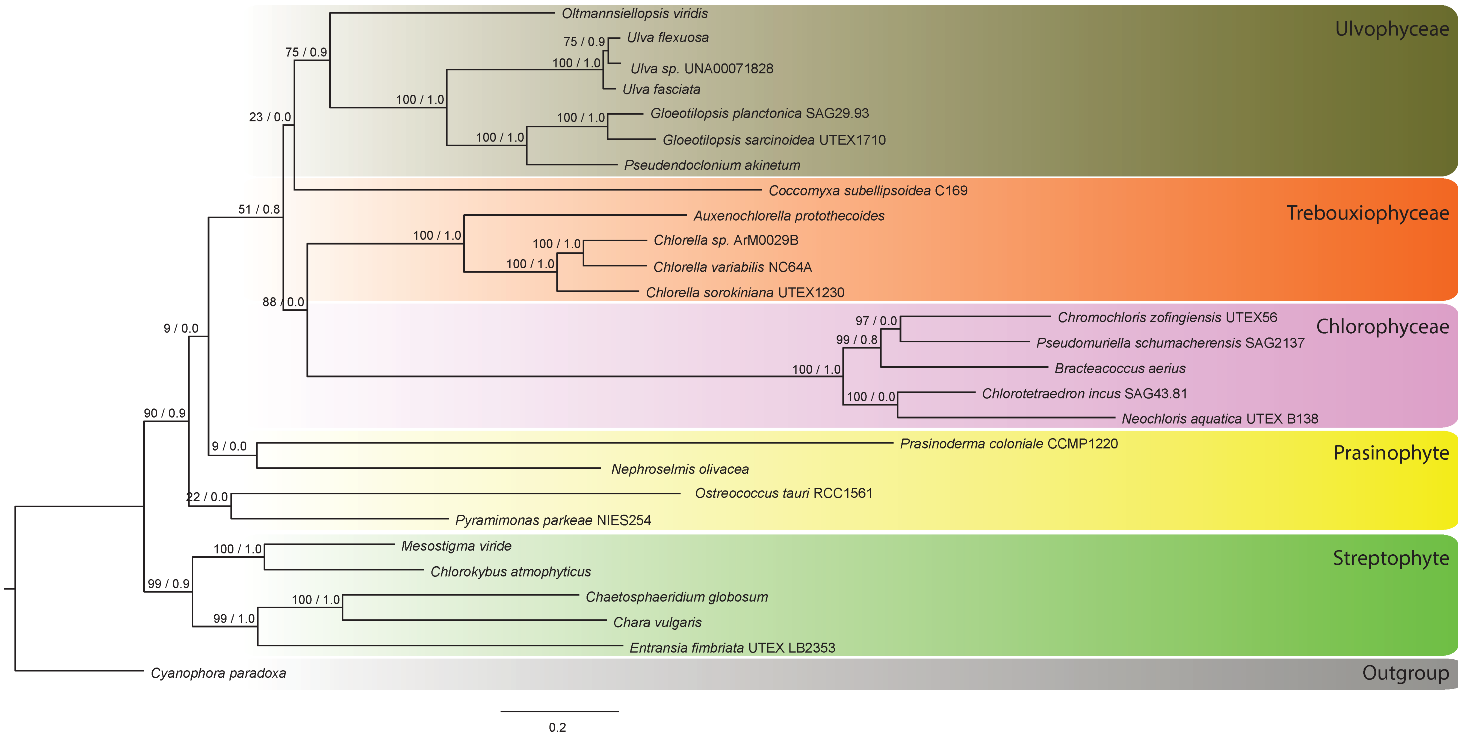

S2 Fig. F. Maximum-Likelihood tree inferred from plastid protein-coding sequences encode for ribosomal protein S of algal taxa in Table 1 using a GTR+Γ+I nucleotide substitution model. Bootstrap value from ML and posterior probability from Bayesian inference are shown at the node. The scale bar represents the estimated number of nucleotide substitution per site.

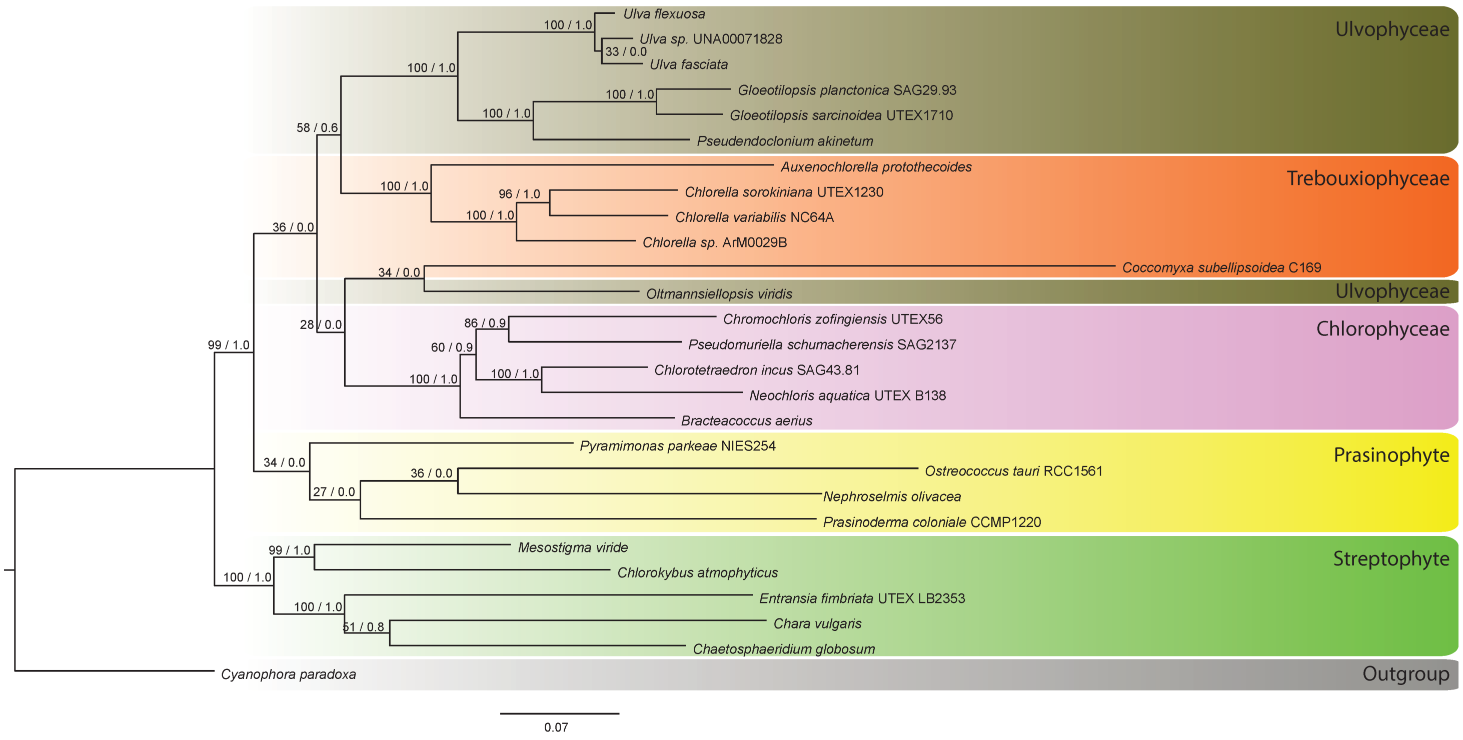

S2 Fig. G. Maximum-Likelihood tree inferred from plastid protein-coding sequences encode for PSI subunits of algal taxa in Table 1 using a GTR+Γ+I nucleotide substitution model. Bootstrap value from ML and posterior probability from Bayesian inference are shown at the node. The scale bar represents the estimated number of nucleotide substitution per site.

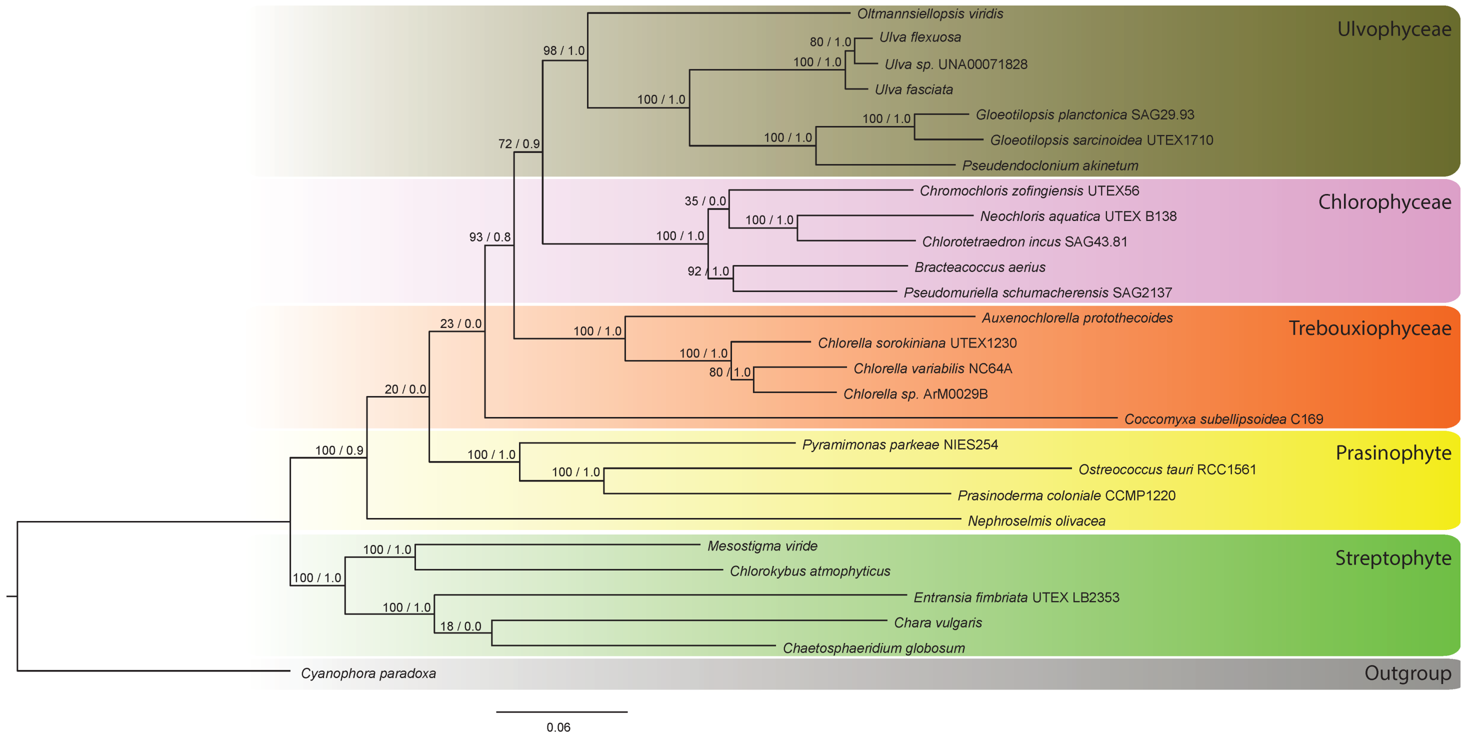

S2 Fig. H. Maximum-Likelihood tree inferred from plastid protein-coding sequences encode for PSII subunits of algal taxa in Table 1 using a GTR+Γ+I nucleotide substitution model. Bootstrap value from ML and posterior probability from Bayesian inference are shown at the node. The scale bar represents the estimated number of nucleotide substitution per site.

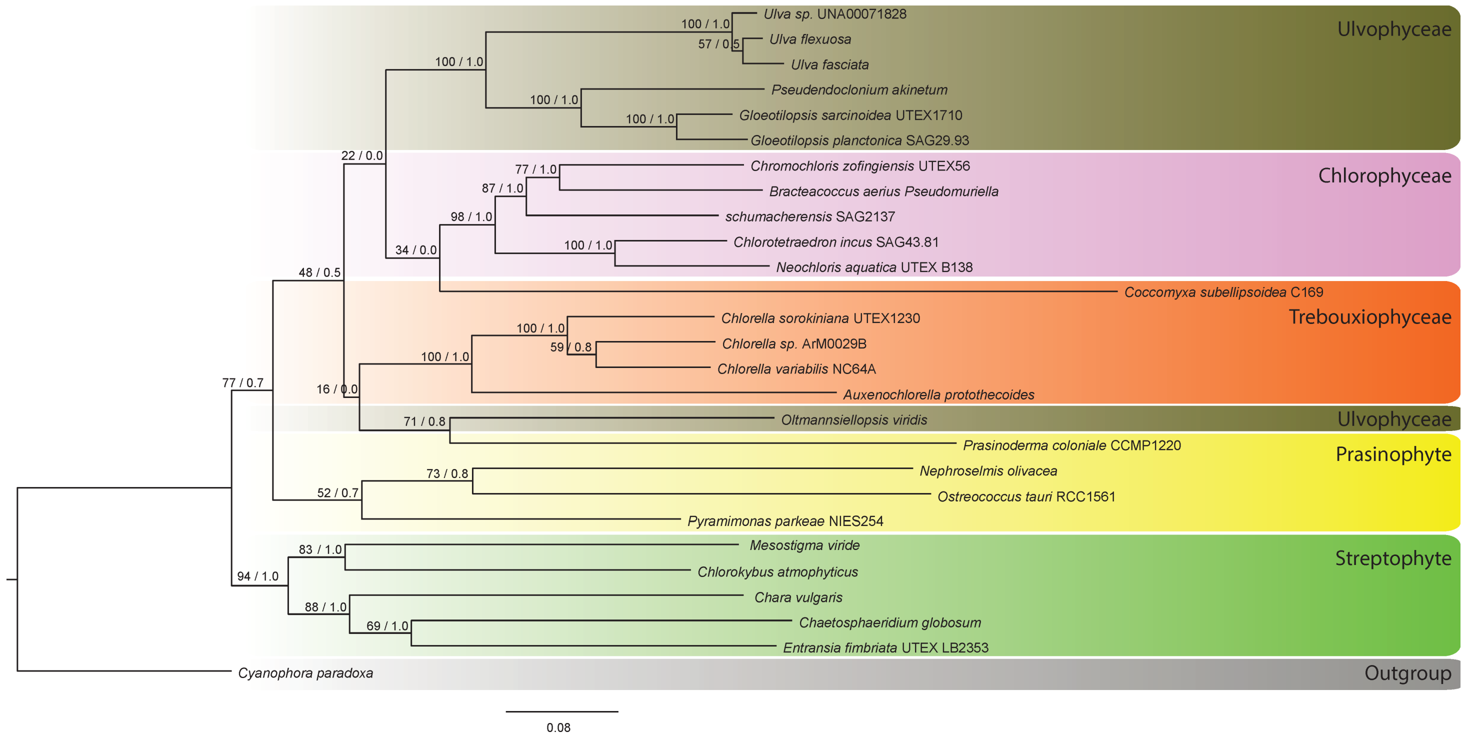

S2 Fig. I. Maximum-Likelihood tree inferred from plastid protein-coding sequences encode for cytochrome b6f complex of algal taxa in Table 1 using a GTR+Γ+I nucleotide substitution model. Bootstrap value from ML and posterior probability from Bayesian inference are shown at the node. The scale bar represents the estimated number of nucleotide substitution per site.
