# Supplementary material for: Evolution of organellar genes of chlorophyte algae: Relevance to phylogenetic inference
Source: PLoS One. 2019 May 6;14(5):e0216608. doi: 10.1371/journal.pone.0216608 (PMC6502327; doi:10.1371/journal.pone.0216608)
Supplement: S3 File — (PDF) [file pone.0216608.s006.pdf]

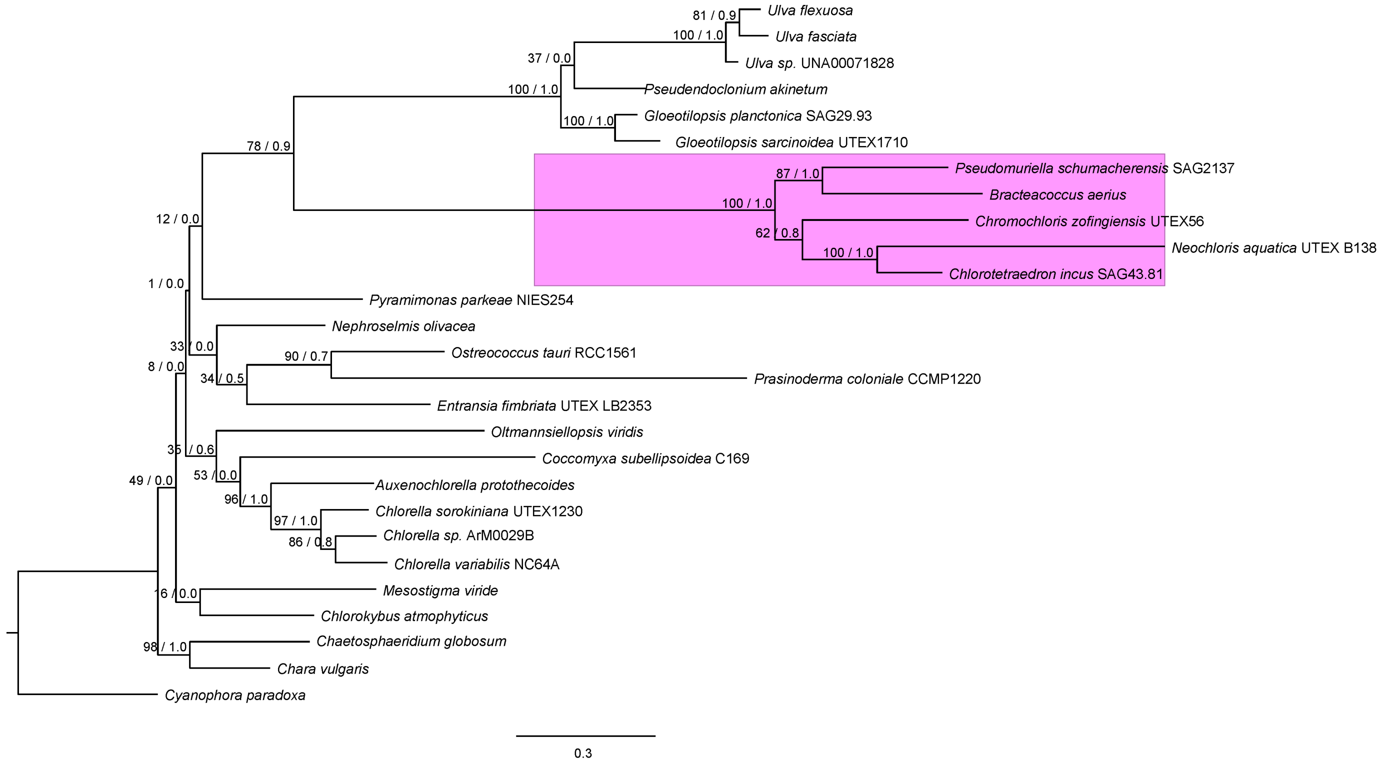

S3 Fig. A. Maximum-Likelihood tree inferred from mitochondrial *nad*1 of algal taxa in Table 1 using a GTR+Γ+I nucleotide substitution model. Bootstrap value from ML and posterior probability from Bayesian inference are shown at the node. The scale bar represents the estimated number of nucleotide substitution per site.

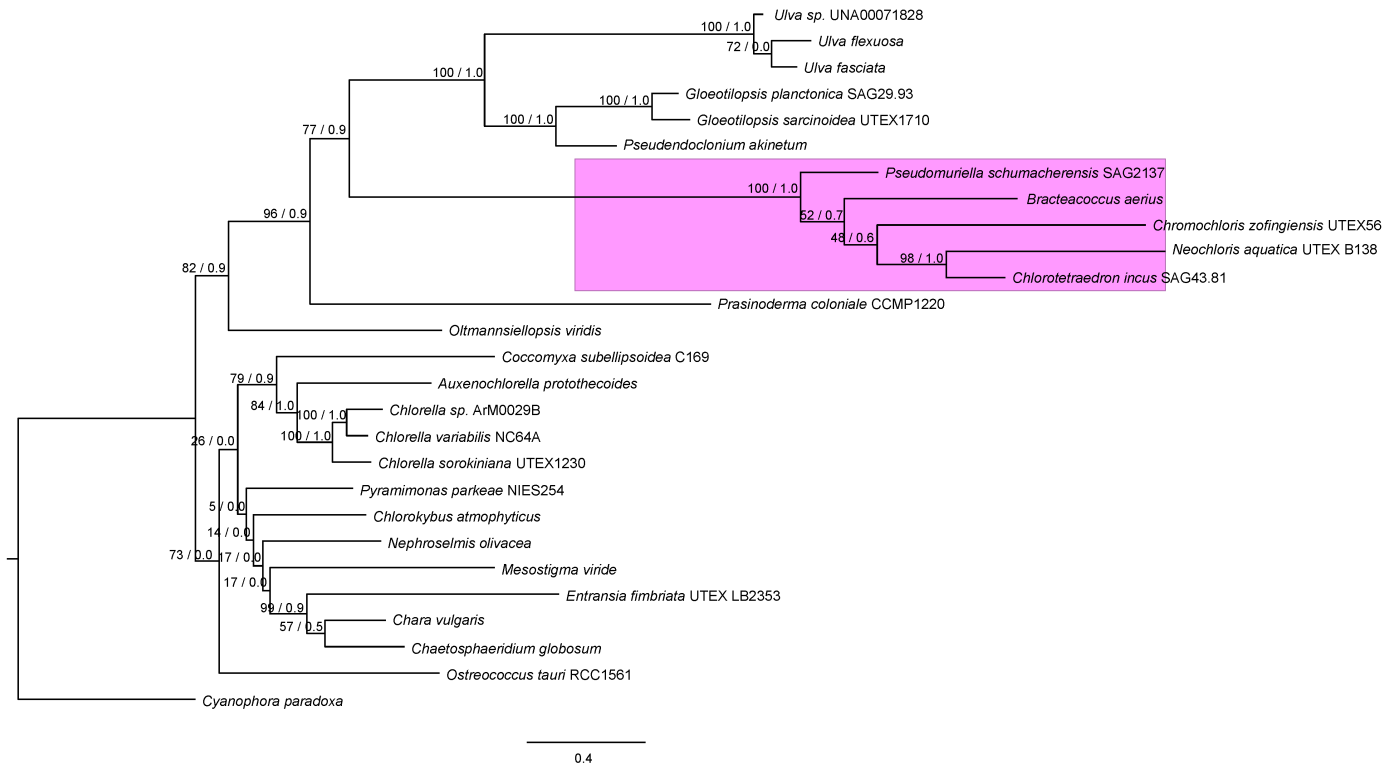

S3 Fig. B. Maximum-Likelihood tree inferred from mitochondrial *nad*2 of algal taxa in Table 1 using a GTR+Γ+I nucleotide substitution model. Bootstrap value from ML and posterior probability from Bayesian inference are shown at the node. The scale bar represents the estimated number of nucleotide substitution per site.

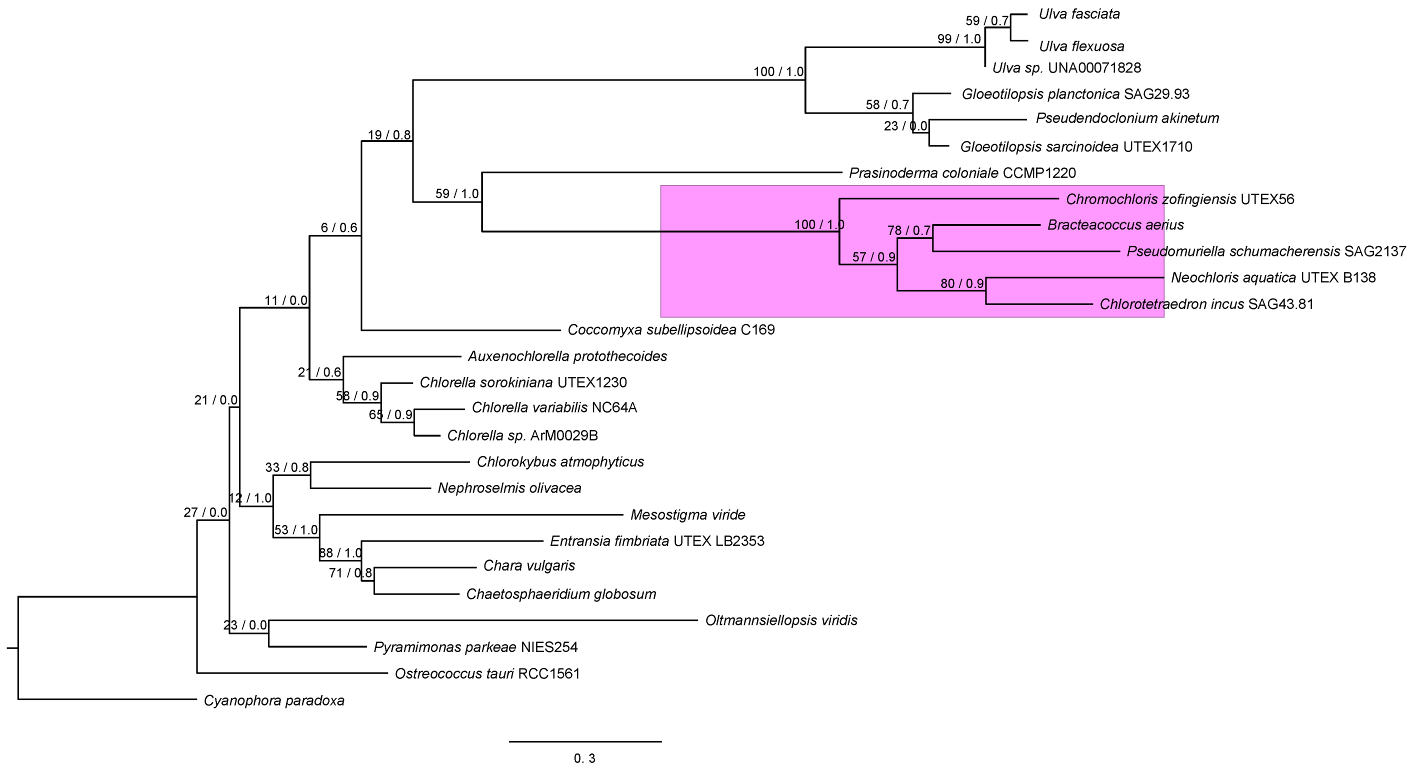

S3 Fig. C. Maximum-Likelihood tree inferred from mitochondrial *nad*3 of algal taxa in Table 1 using a GTR+Γ+I nucleotide substitution model. Bootstrap value from ML and posterior probability from Bayesian inference are shown at the node. The scale bar represents the estimated number of nucleotide substitution per site.

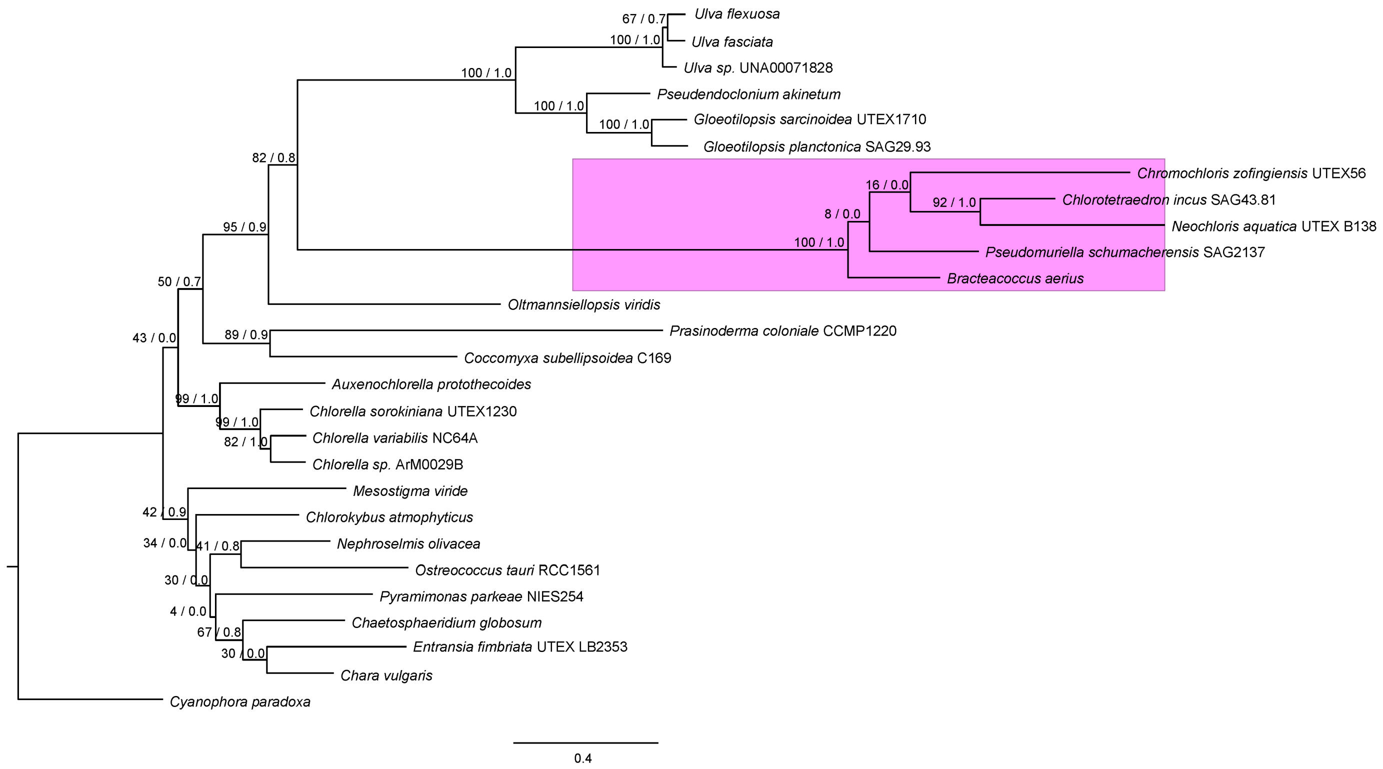

S3 Fig. D. Maximum-Likelihood tree inferred from mitochondrial *nad*4 of algal taxa in Table 1 using a GTR+Γ+I nucleotide substitution model. Bootstrap value from ML and posterior probability from Bayesian inference are shown at the node. The scale bar represents the estimated number of nucleotide substitution per site.

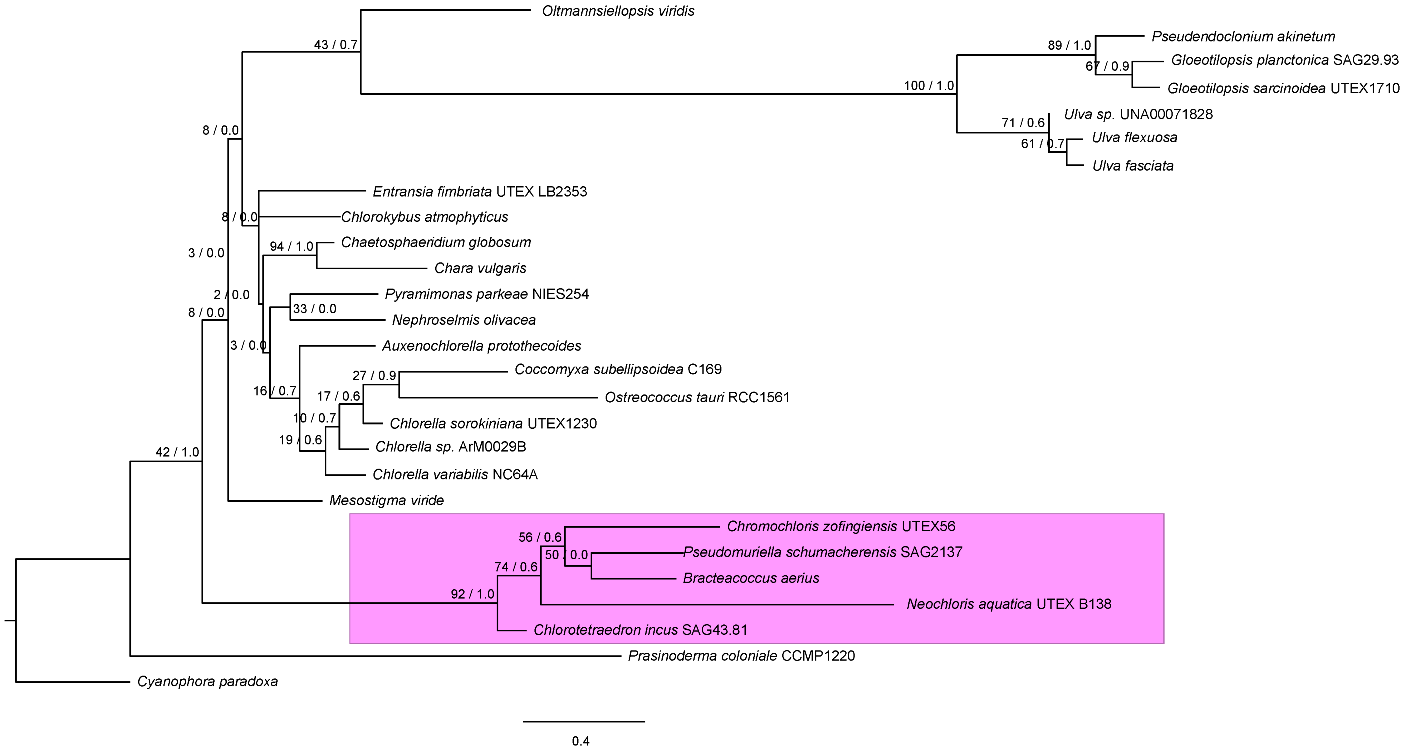

S3 Fig. E. Maximum-Likelihood tree inferred from mitochondrial *nad*4L of algal taxa in Table 1 using a GTR+Γ+I nucleotide substitution model. Bootstrap value from ML and posterior probability from Bayesian inference are shown at the node. The scale bar represents the estimated number of nucleotide substitution per site.

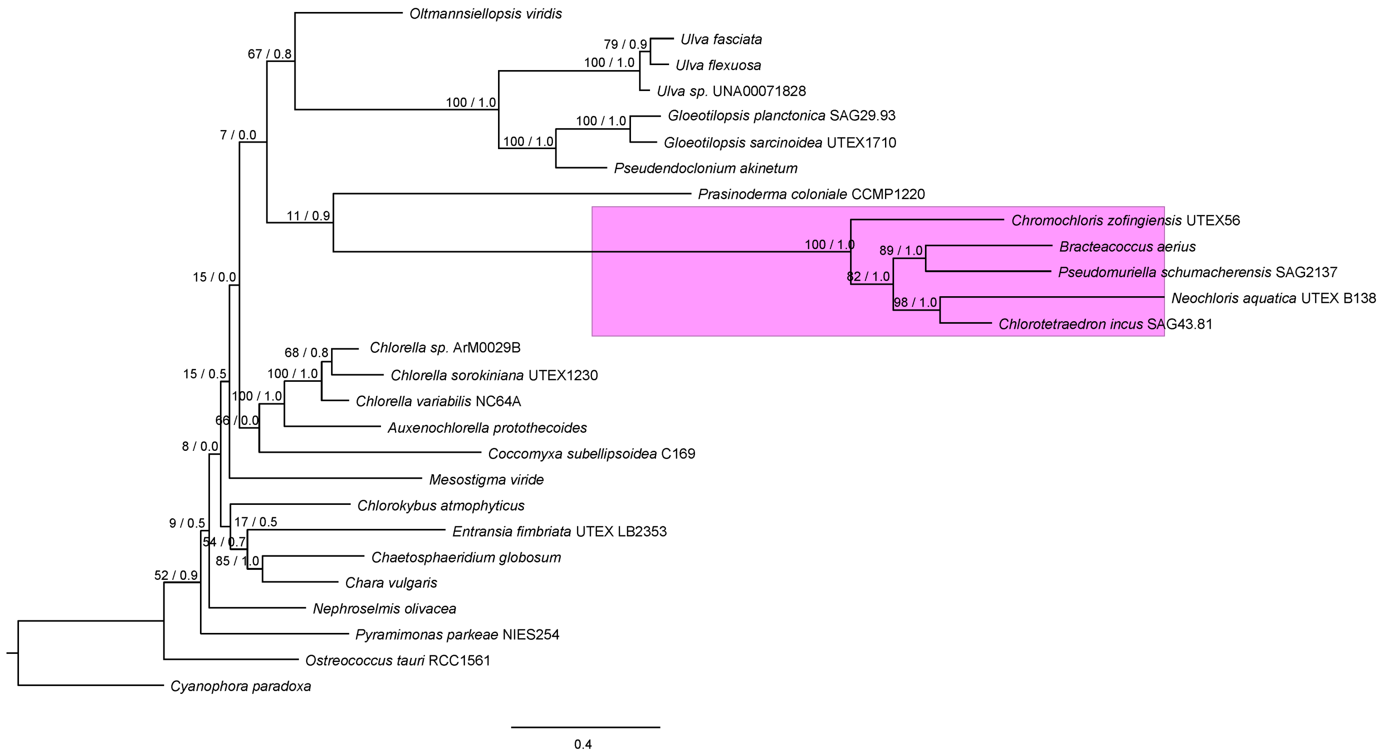

S3 Fig. F. Maximum-Likelihood tree inferred from mitochondrial *nad*5 of algal taxa in Table 1 using a GTR+Γ+I nucleotide substitution model. Bootstrap value from ML and posterior probability from Bayesian inference are shown at the node. The scale bar represents the estimated number of nucleotide substitution per site.

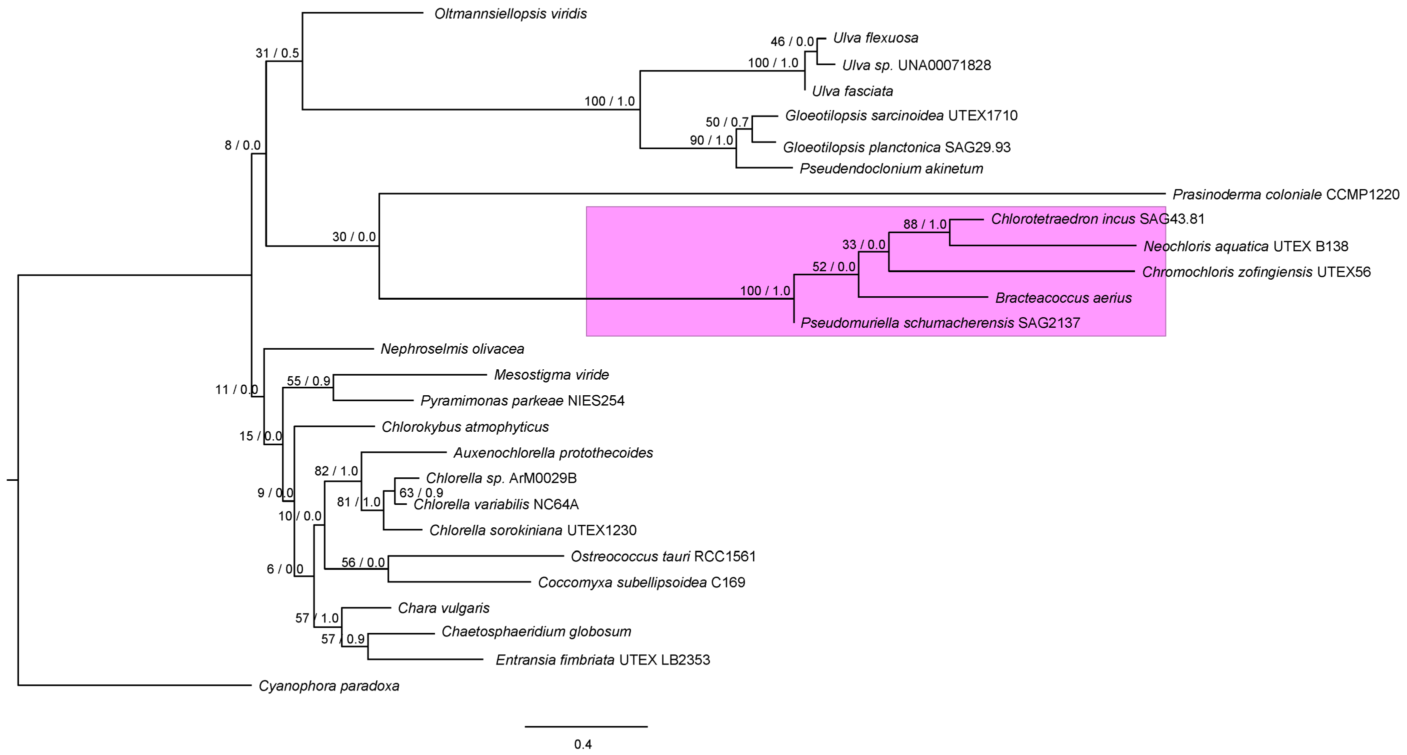

S3 Fig. G. Maximum-Likelihood tree inferred from mitochondrial *nad*6 of algal taxa in Table 1 using a GTR+Γ+I nucleotide substitution model. Bootstrap value from ML and posterior probability from Bayesian inference are shown at the node. The scale bar represents the estimated number of nucleotide substitution per site.

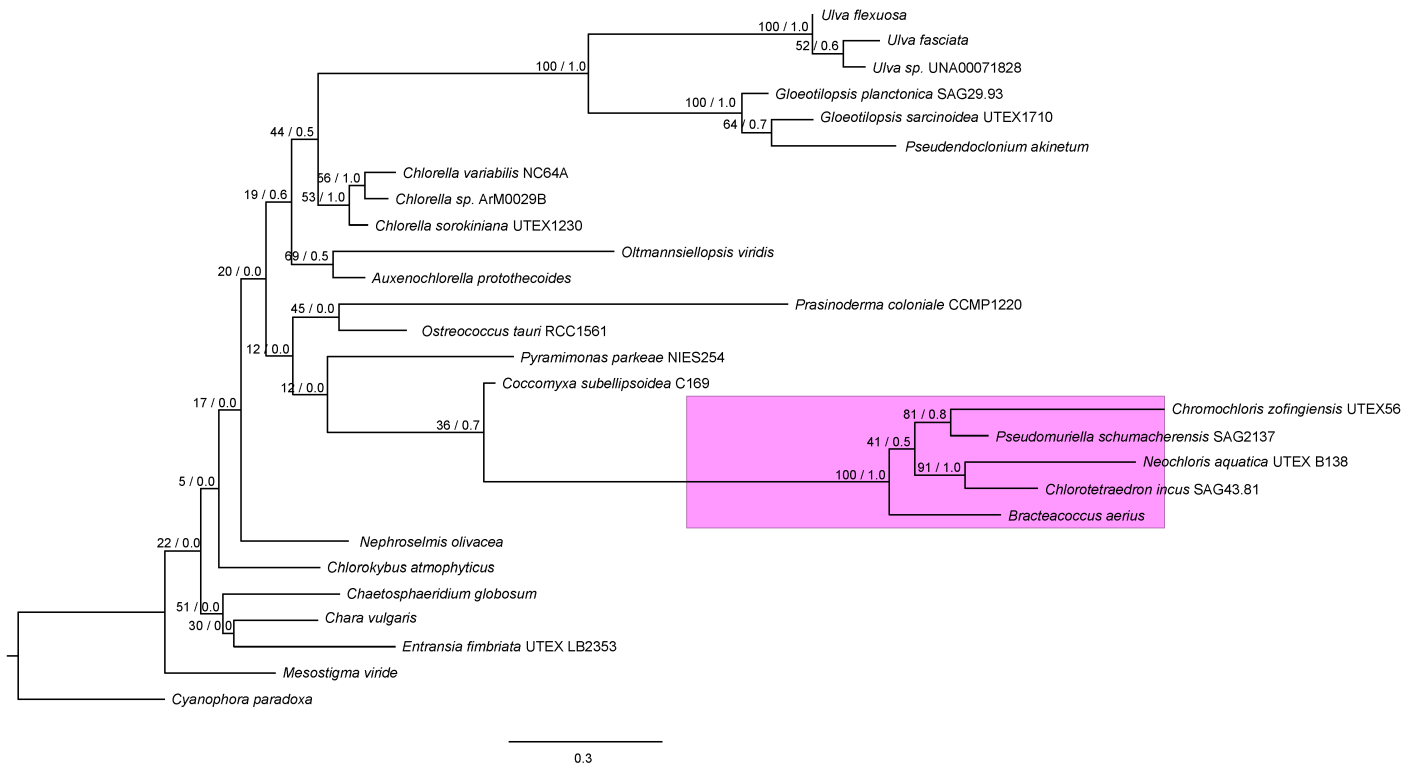

S3 Fig. H. Maximum-Likelihood tree inferred from mitochondrial *cox*2 of algal taxa in Table 1 using a GTR+Γ+I nucleotide substitution model. Bootstrap value from ML and posterior probability from Bayesian inference are shown at the node. The scale bar represents the estimated number of nucleotide substitution per site.

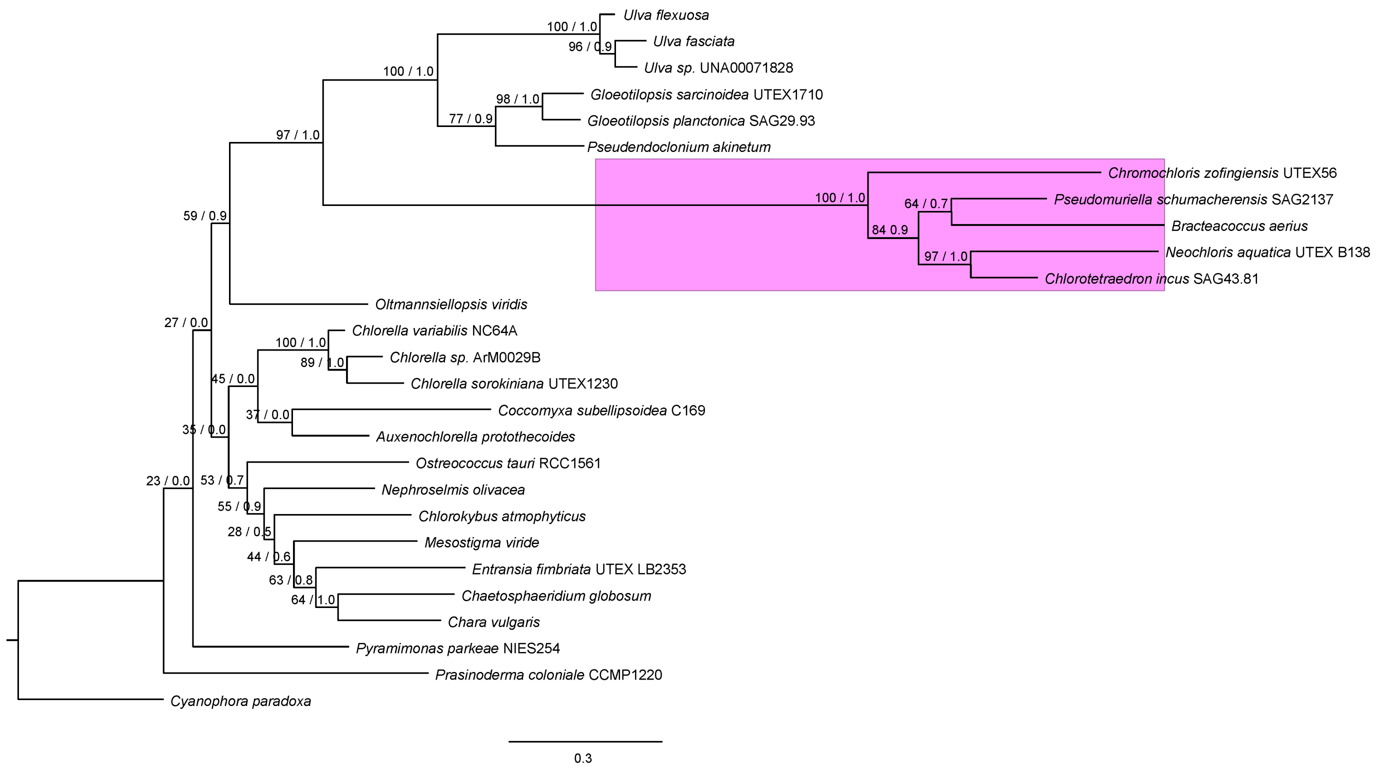

S3 Fig. I. Maximum-Likelihood tree inferred from mitochondrial *cox*3 of algal taxa in Table 1 using a GTR+Γ+I nucleotide substitution model. Bootstrap value from ML and posterior probability from Bayesian inference are shown at the node. The scale bar represents the estimated number of nucleotide substitution per site.

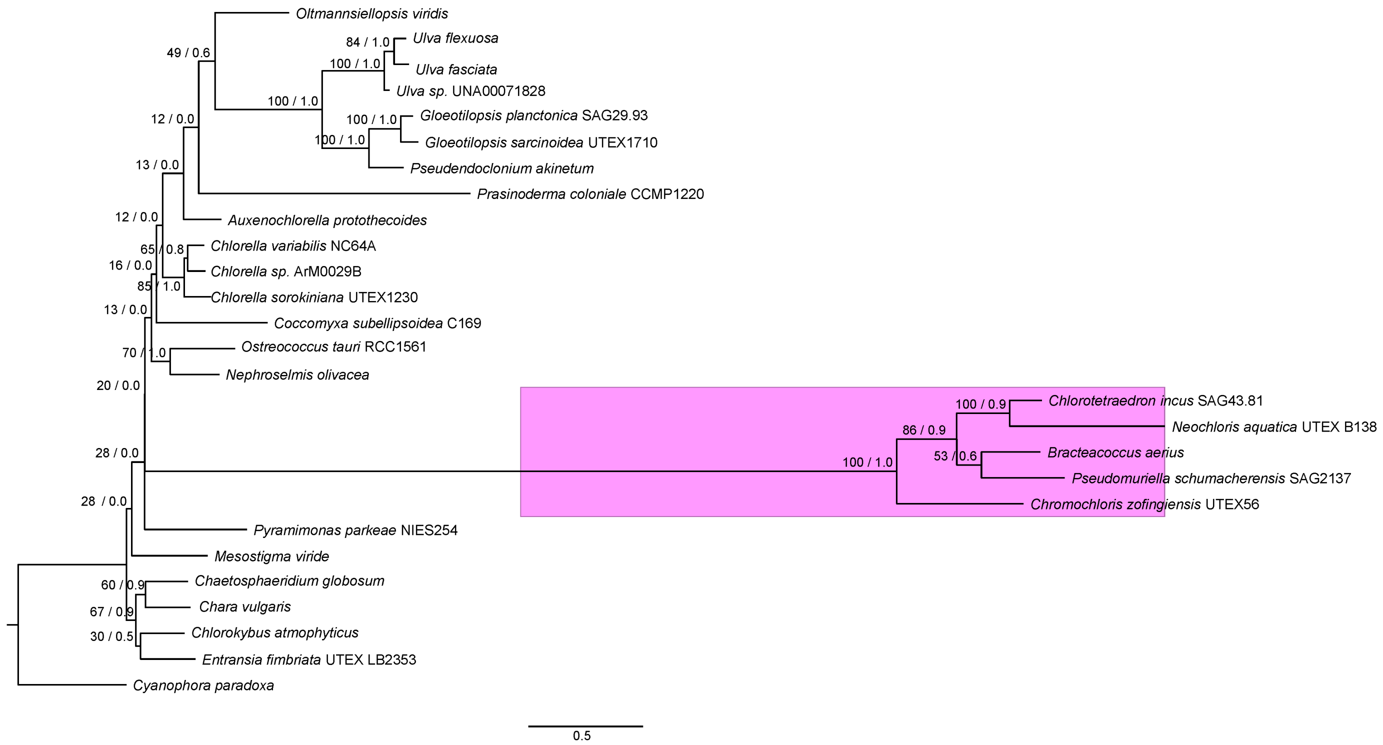

S3 Fig. J. Maximum-Likelihood tree inferred from mitochondrial *atp*6 of algal taxa in Table 1 using a GTR+Γ+I nucleotide substitution model. Bootstrap value from ML and posterior probability from Bayesian inference are shown at the node. The scale bar represents the estimated number of nucleotide substitution per site.

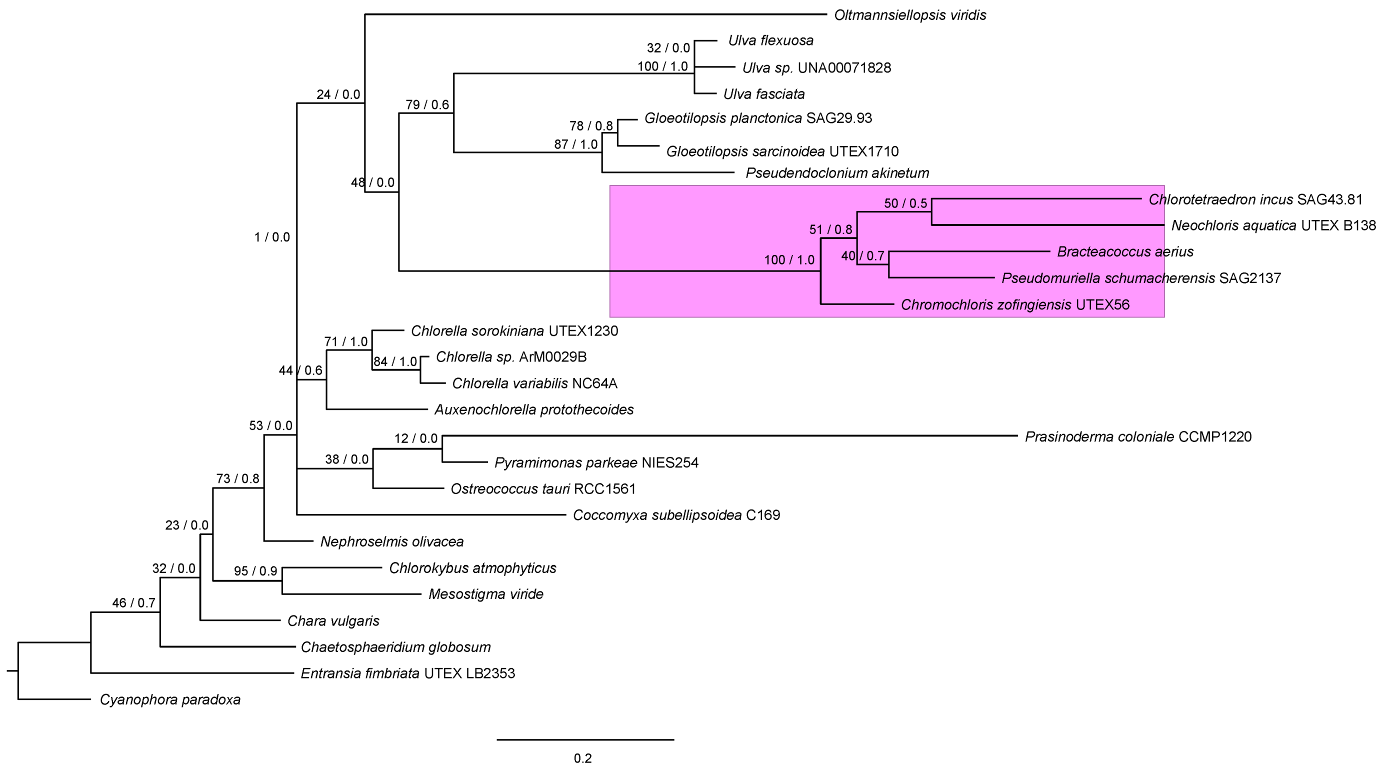

S3 Fig. K. Maximum-Likelihood tree inferred from mitochondrial *atp*9 of algal taxa in Table 1 using a GTR+Γ+I nucleotide substitution model. Bootstrap value from ML and posterior probability from Bayesian inference are shown at the node. The scale bar represents the estimated number of nucleotide substitution per site.

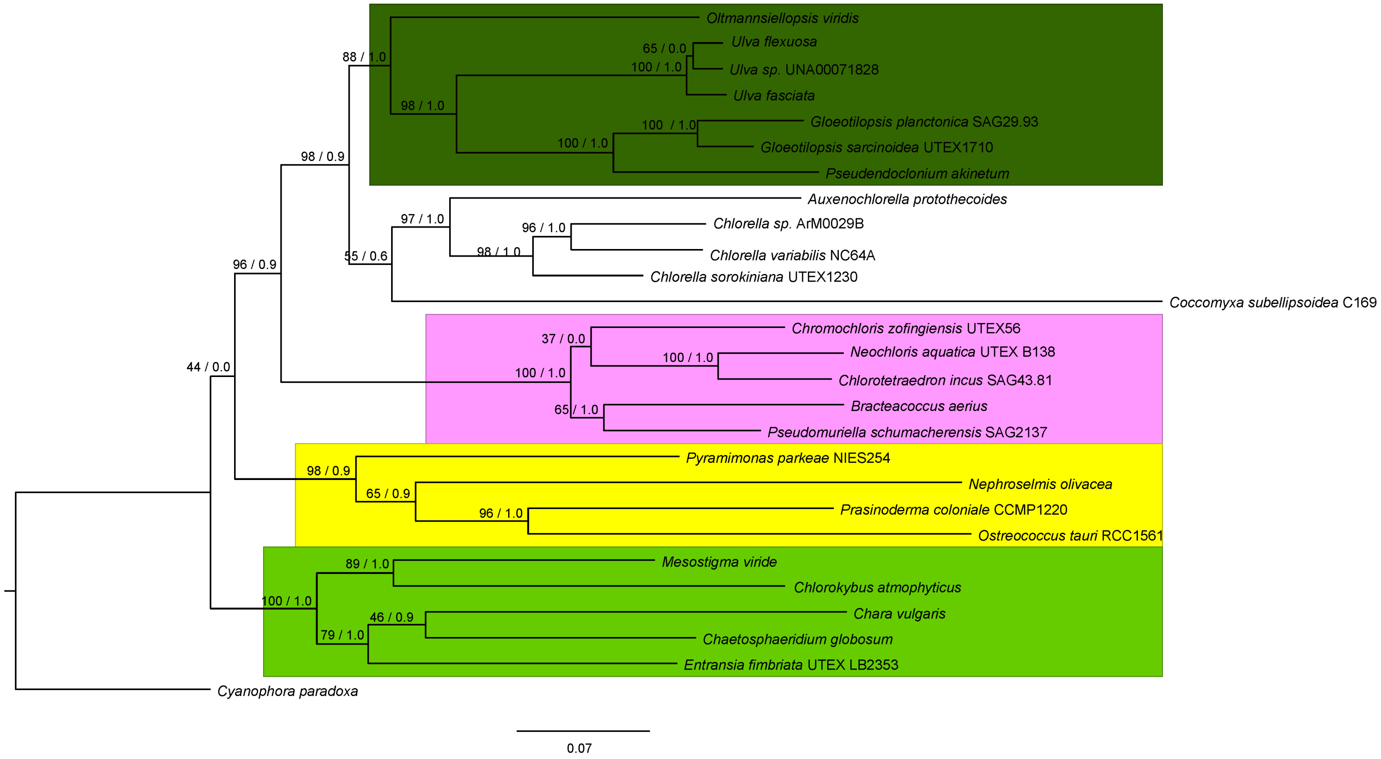

S3 Fig. L. Maximum-Likelihood tree inferred from plastid *atp*A of algal taxa in Table 1 using a GTR+Γ+I nucleotide substitution model. Bootstrap value from ML and posterior probability from Bayesian inference are shown at the node. The scale bar represents the estimated number of nucleotide substitution per site.

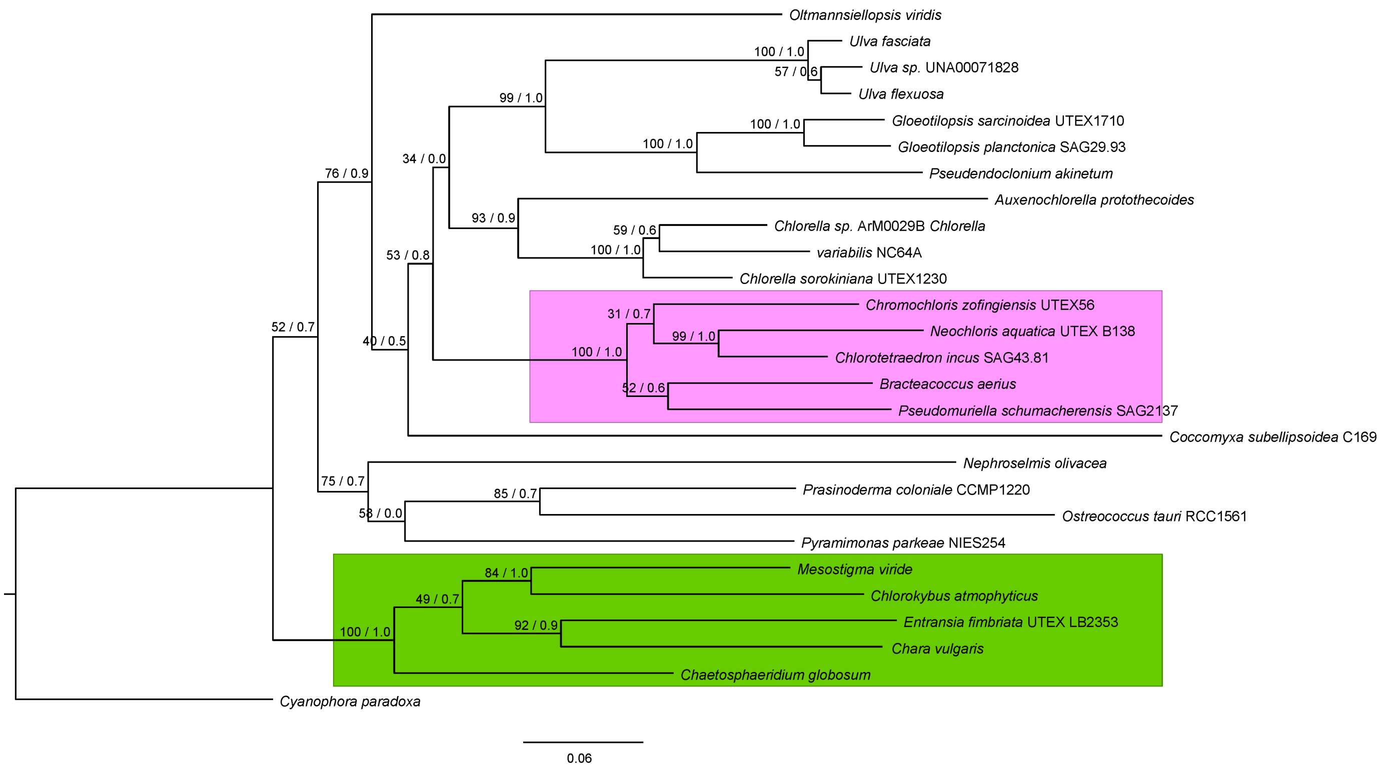

S3 Fig. M. Maximum-Likelihood tree inferred from plastid *atp*B of algal taxa in Table 1 using a GTR+Γ+I nucleotide substitution model. Bootstrap value from ML and posterior probability from Bayesian inference are shown at the node. The scale bar represents the estimated number of nucleotide substitution per site.

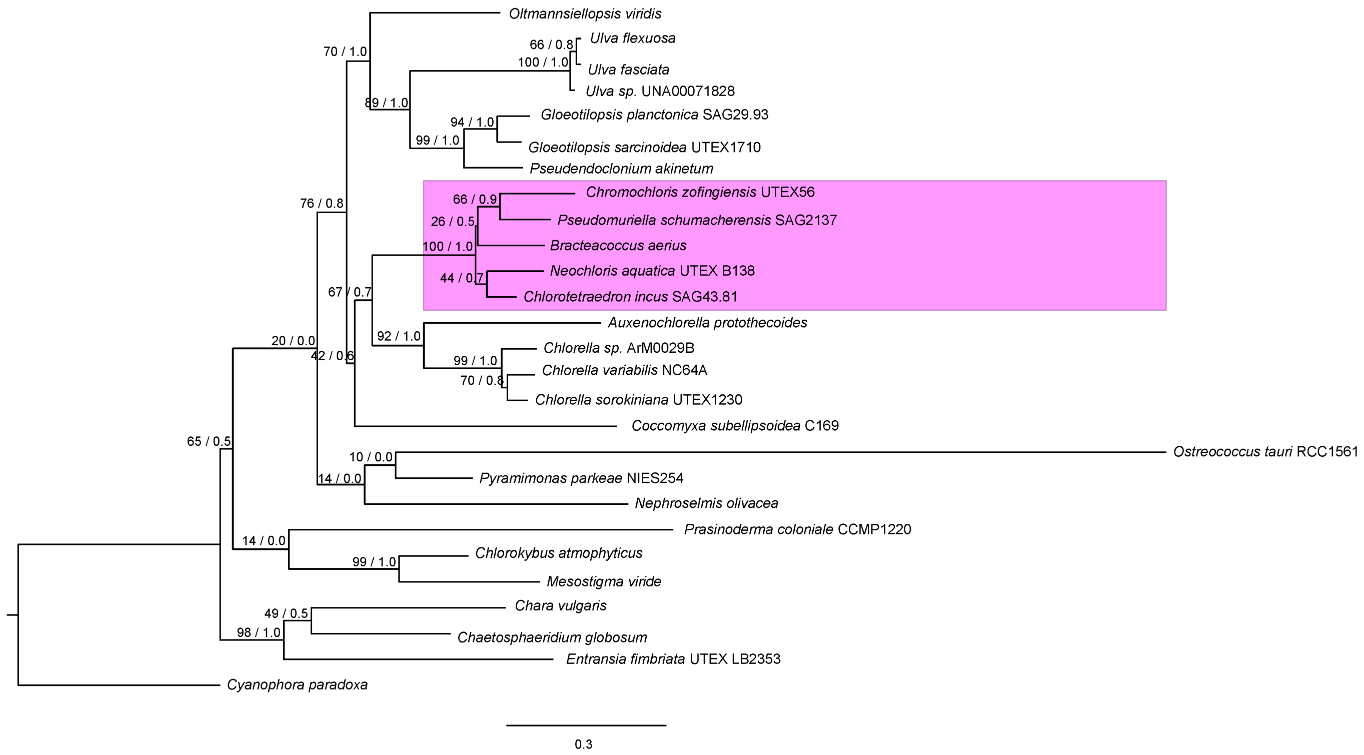

S3 Fig. N. Maximum-Likelihood tree inferred from plastid *atp*E of algal taxa in Table 1 using a GTR+Γ+I nucleotide substitution model. Bootstrap value from ML and posterior probability from Bayesian inference are shown at the node. The scale bar represents the estimated number of nucleotide substitution per site.

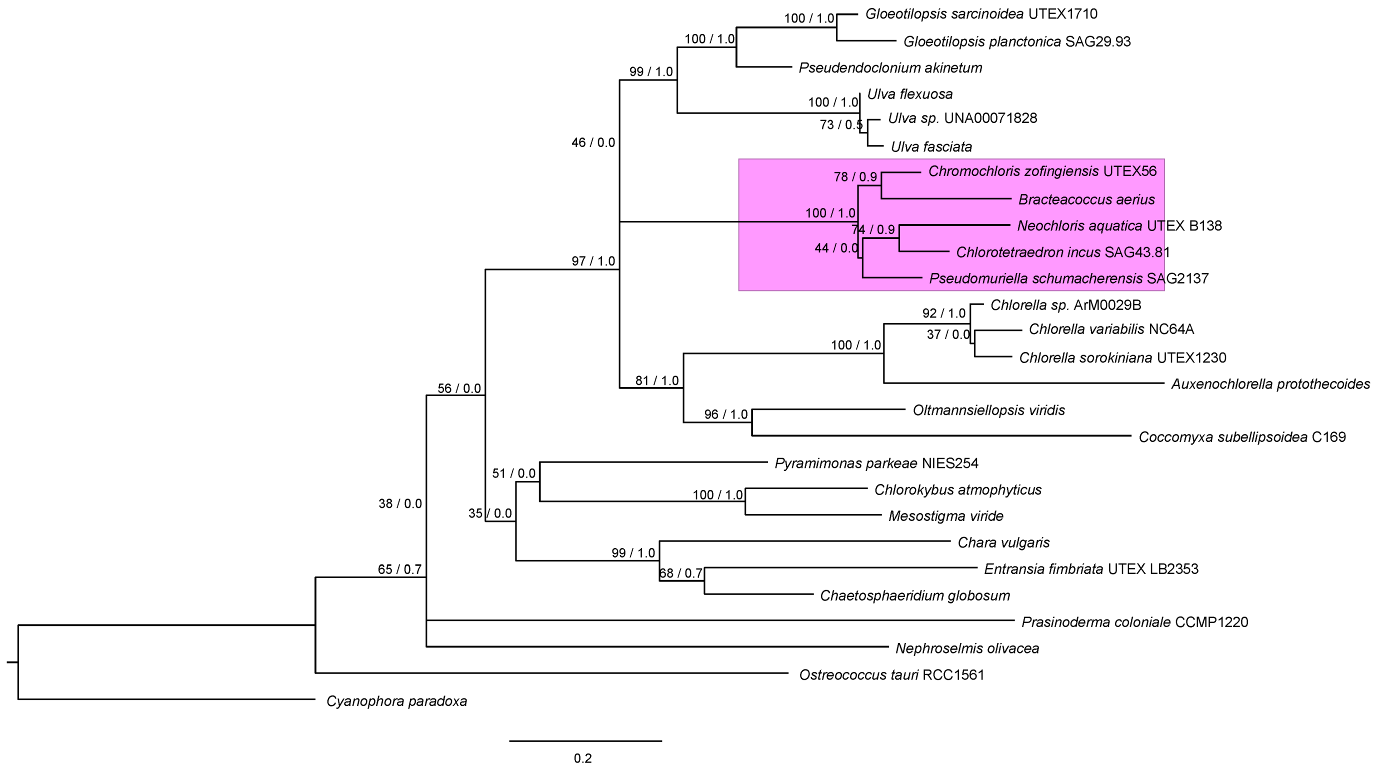

S3 Fig. O. Maximum-Likelihood tree inferred from plastid *atp*F of algal taxa in Table 1 using a GTR+Γ+I nucleotide substitution model. Bootstrap value from ML and posterior probability from Bayesian inference are shown at the node. The scale bar represents the estimated number of nucleotide substitution per site.

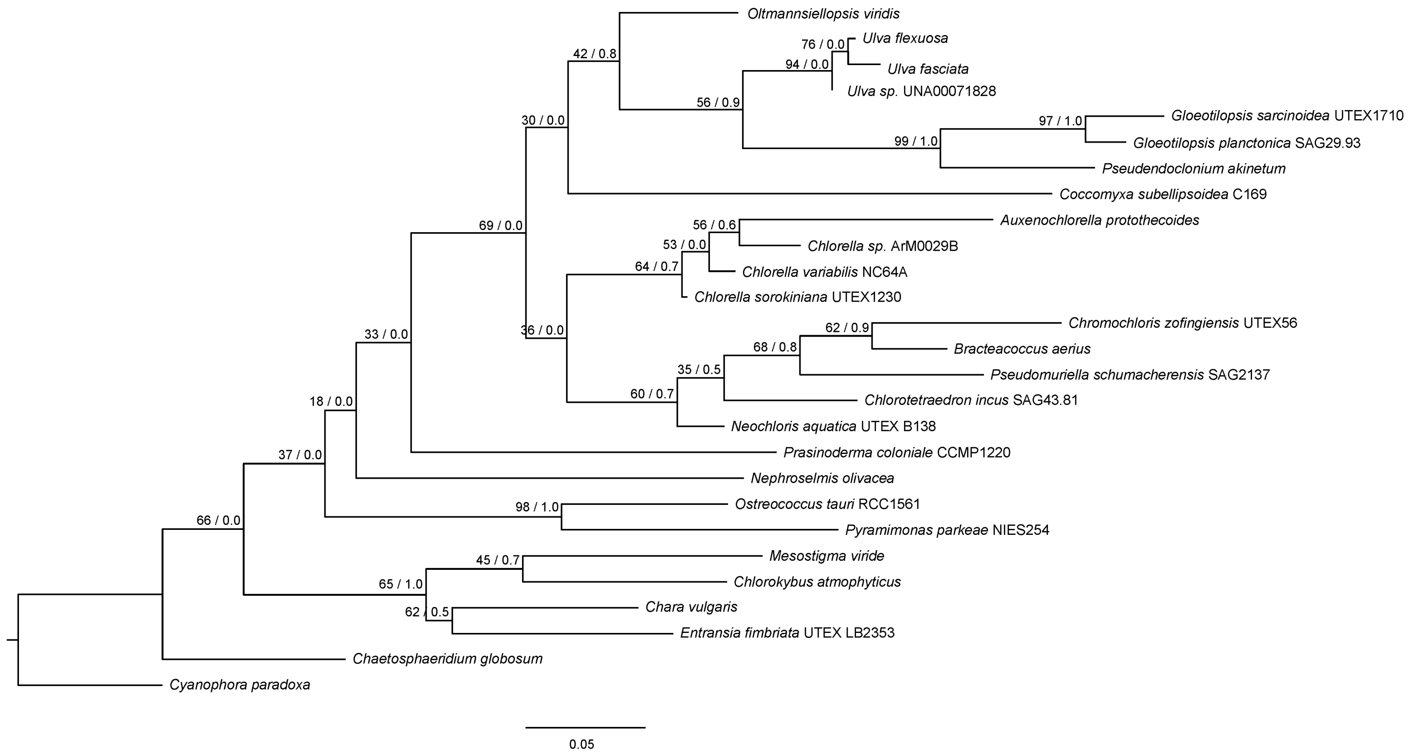

S3 Fig. P. Maximum-Likelihood tree inferred from plastid *atp*H of algal taxa in Table 1 using a GTR+Γ+I nucleotide substitution model. Bootstrap value from ML and posterior probability from Bayesian inference are shown at the node. The scale bar represents the estimated number of nucleotide substitution per site.

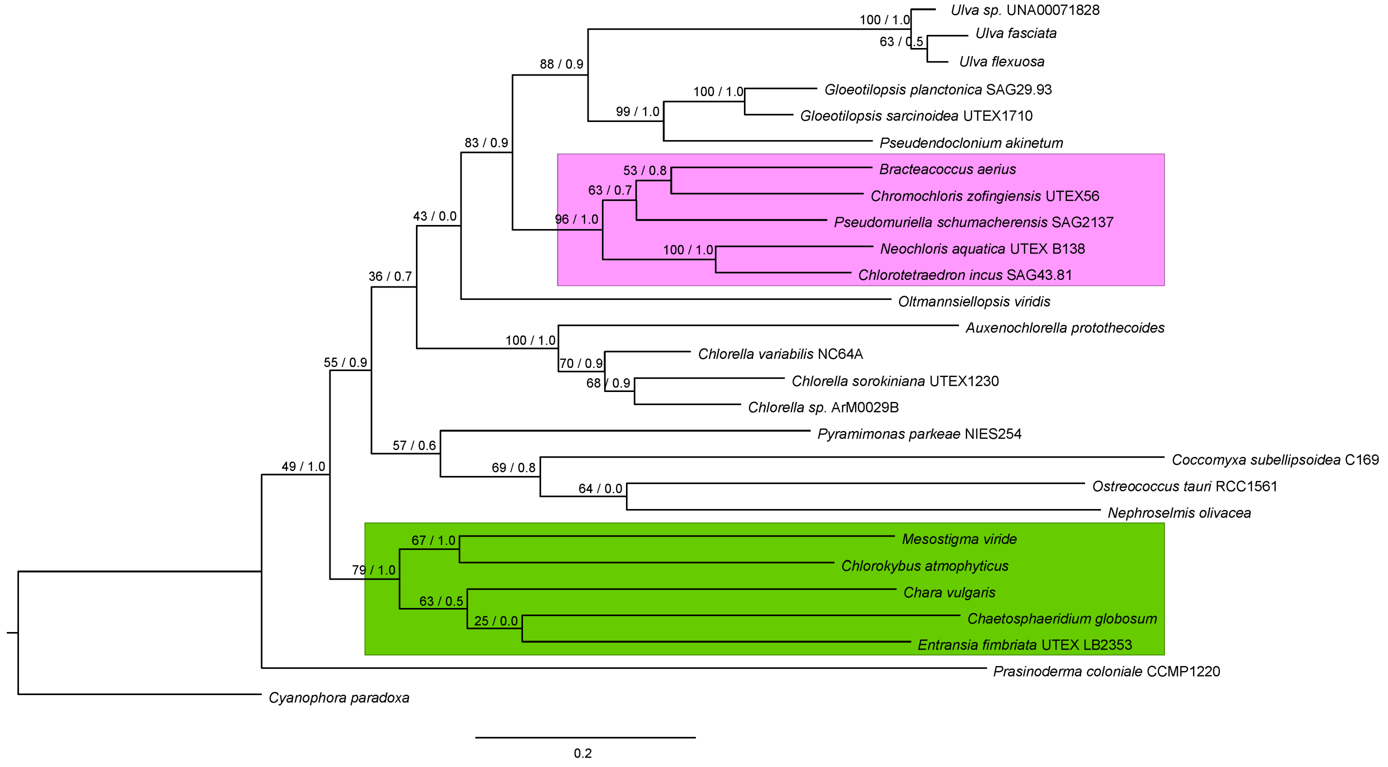

S3 Fig. Q. Maximum-Likelihood tree inferred from plastid *pet*A of algal taxa in Table 1 using a GTR+Γ+I nucleotide substitution model. Bootstrap value from ML and posterior probability from Bayesian inference are shown at the node. The scale bar represents the estimated number of nucleotide substitution per site.

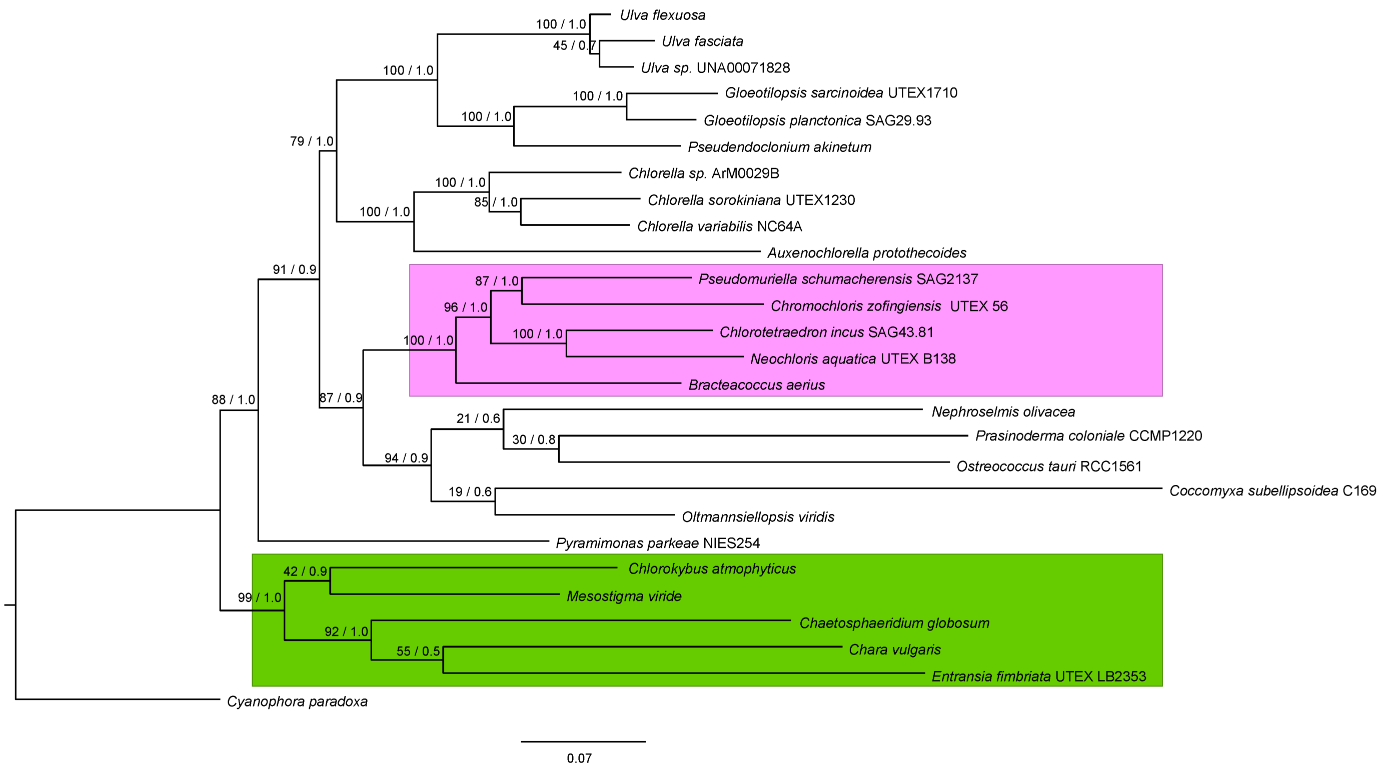

S3 Fig. R Maximum-Likelihood tree inferred from plastid *psa*A of algal taxa in Table 1 using a GTR+Γ+I nucleotide substitution model. Bootstrap value from ML and posterior probability from Bayesian inference are shown at the node. The scale bar represents the estimated number of nucleotide substitution per site.

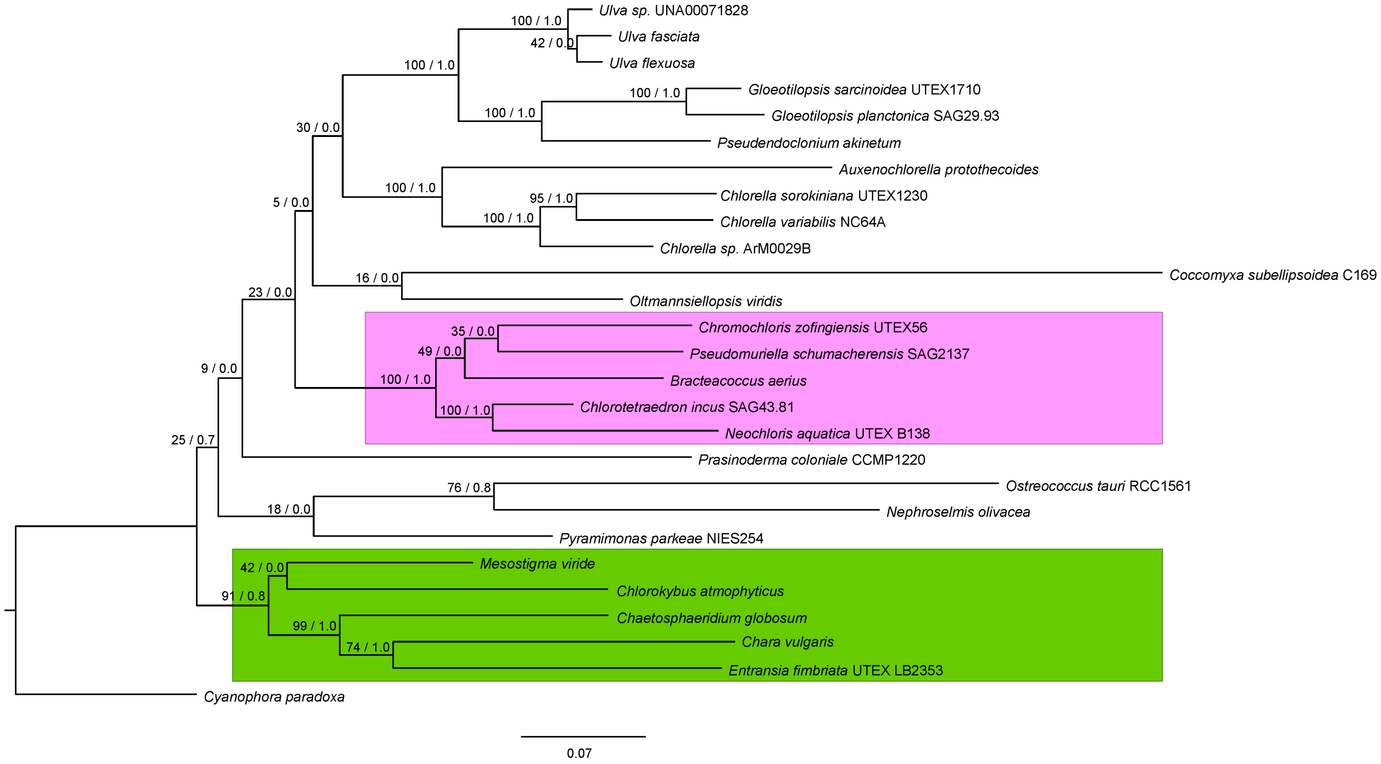

S3 Fig. S. Maximum-Likelihood tree inferred from plastid *psa*B of algal taxa in Table 1 using a GTR+Γ+I nucleotide substitution model. Bootstrap value from ML and posterior probability from Bayesian inference are shown at the node. The scale bar represents the estimated number of nucleotide substitution per site.

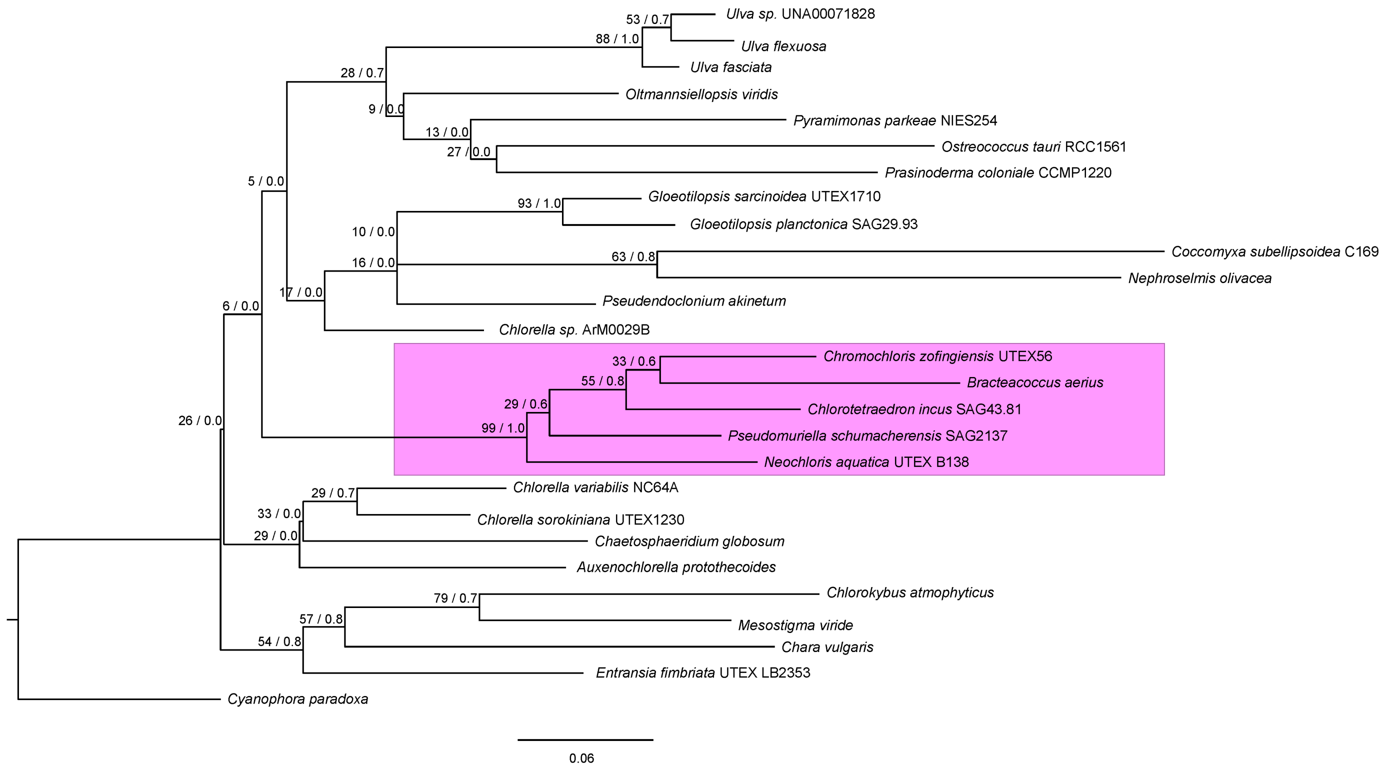

S3 Fig. T. Maximum-Likelihood tree inferred from plastid *psa*C of algal taxa in Table 1 using a GTR+Γ+I nucleotide substitution model. Bootstrap value from ML and posterior probability from Bayesian inference are shown at the node. The scale bar represents the estimated number of nucleotide substitution per site.

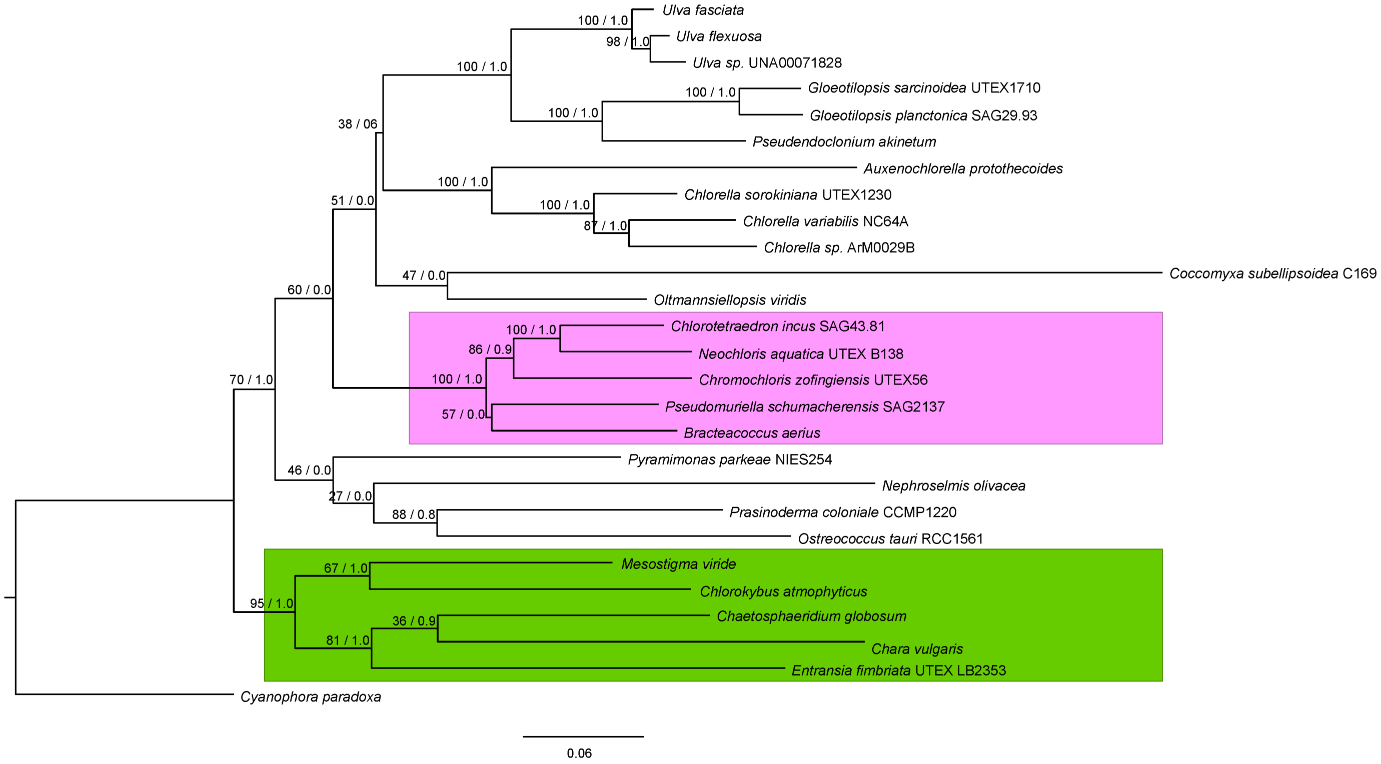

S3 Fig. U. Maximum-Likelihood tree inferred from plastid *psb*B of algal taxa in Table 1 using a GTR+Γ+I nucleotide substitution model. Bootstrap value from ML and posterior probability from Bayesian inference are shown at the node. The scale bar represents the estimated number of nucleotide substitution per site.

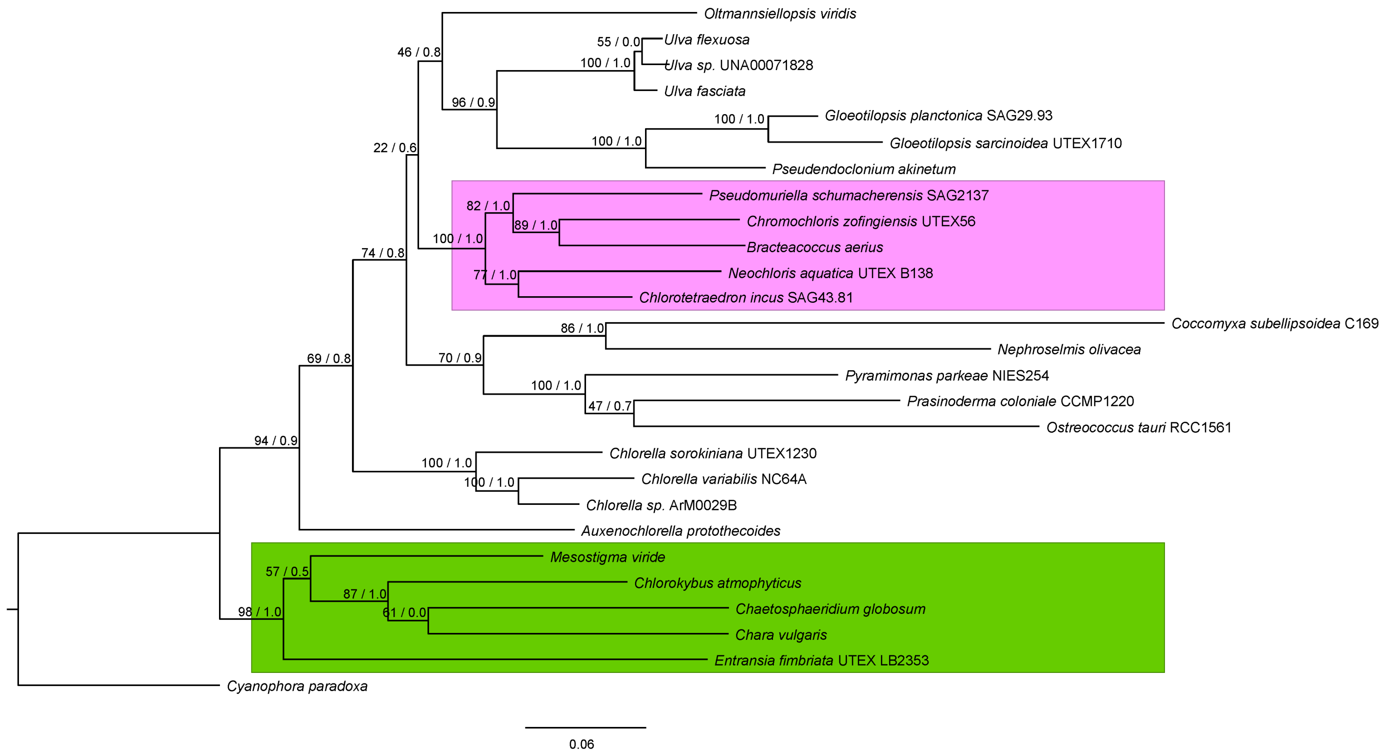

S3 Fig. V. Maximum-Likelihood tree inferred from plastid *psb*C of algal taxa in Table 1 using a GTR+Γ+I nucleotide substitution model. Bootstrap value from ML and posterior probability from Bayesian inference are shown at the node. The scale bar represents the estimated number of nucleotide substitution per site.

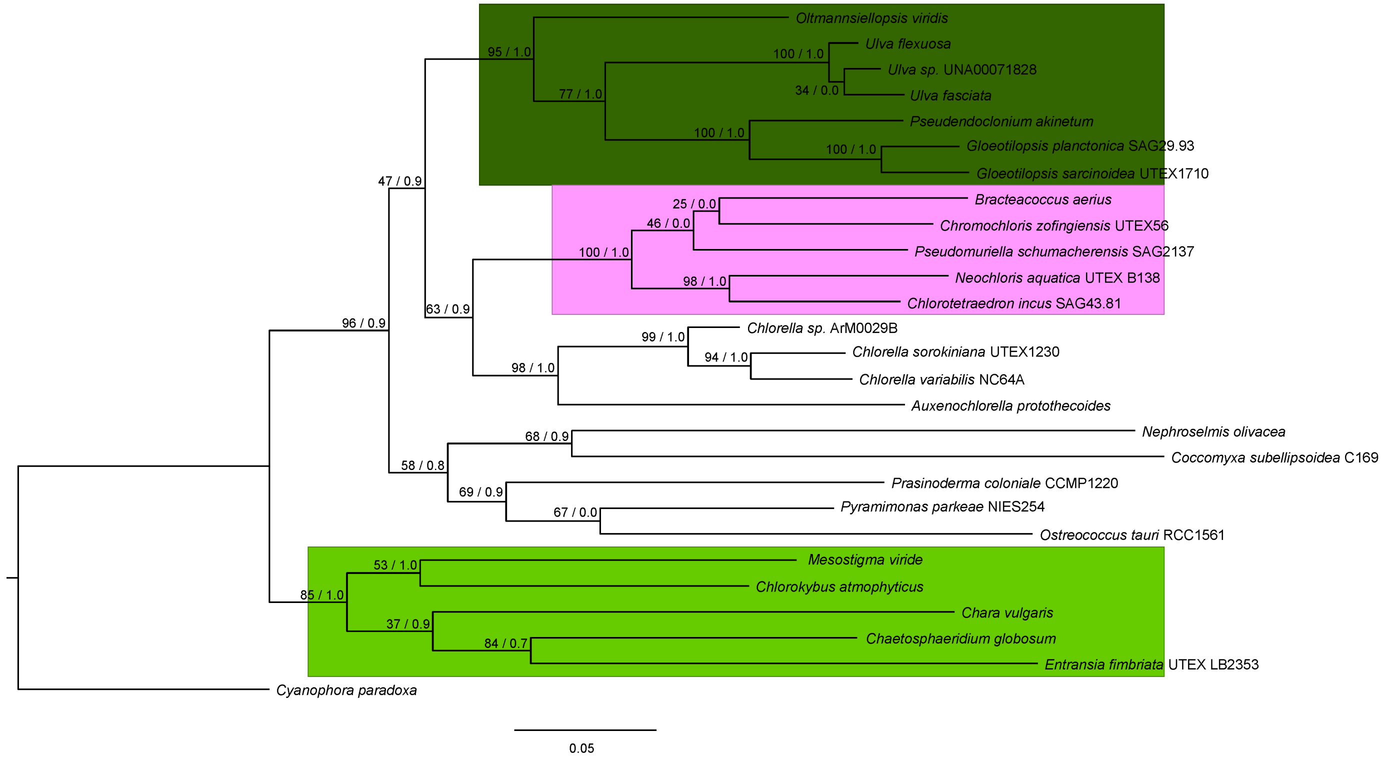

S3 Fig. W. Maximum-Likelihood tree inferred from plastid *psb*D of algal taxa in Table 1 using a GTR+Γ+I nucleotide substitution model. Bootstrap value from ML and posterior probability from Bayesian inference are shown at the node. The scale bar represents the estimated number of nucleotide substitution per site.

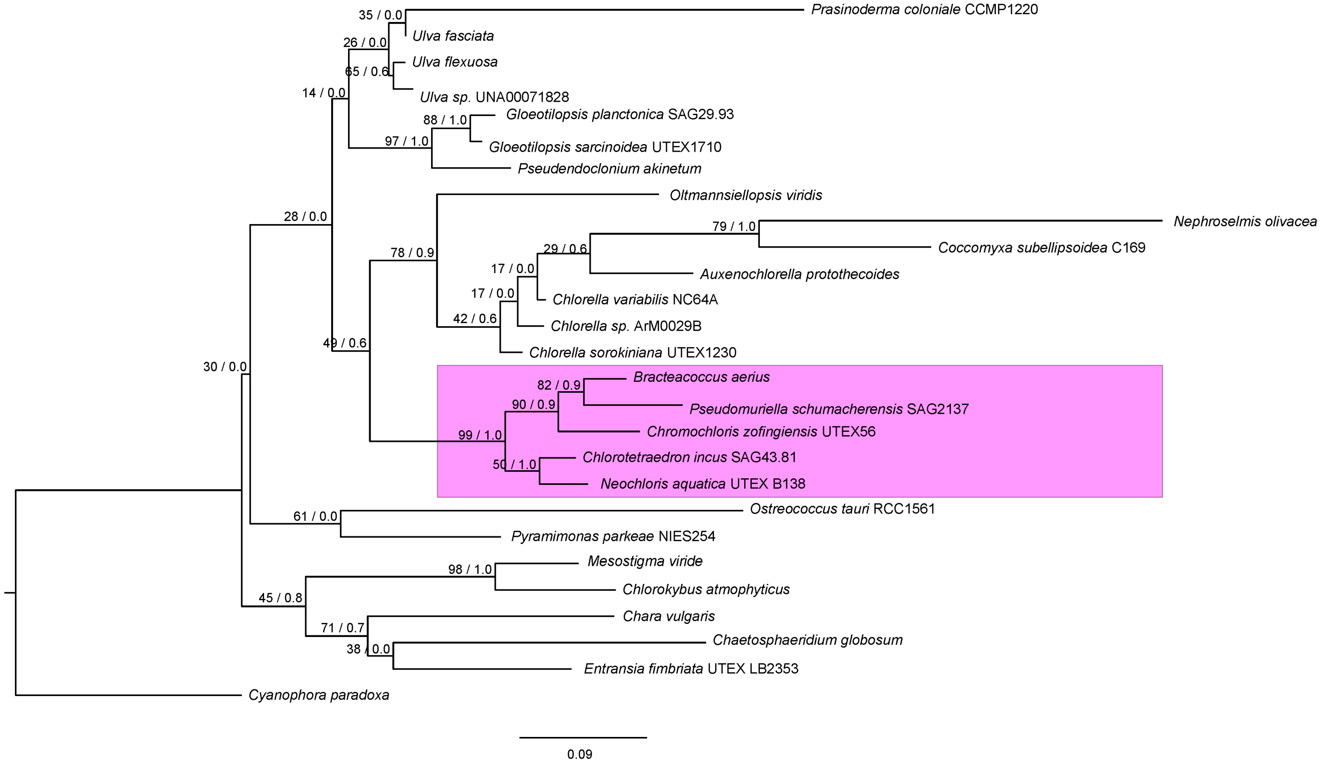

S3 Fig. X. Maximum-Likelihood tree inferred from plastid *psb*E of algal taxa in Table 1 using a GTR+Γ+I nucleotide substitution model. Bootstrap value from ML and posterior probability from Bayesian inference are shown at the node. The scale bar represents the estimated number of nucleotide substitution per site.

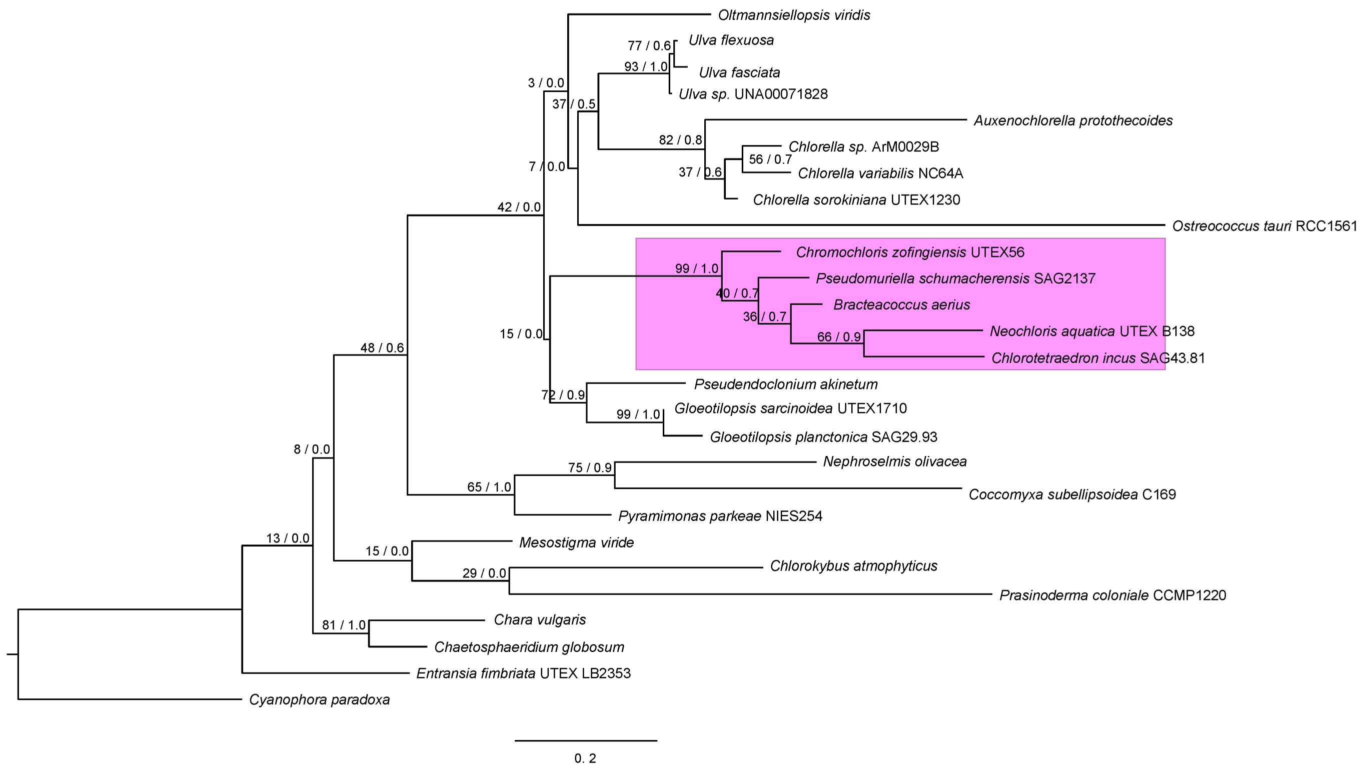

S3 Fig. Y. Maximum-Likelihood tree inferred from plastid *psb*H of algal taxa in Table 1 using a GTR+Γ+I nucleotide substitution model. Bootstrap value from ML and posterior probability from Bayesian inference are shown at the node. The scale bar represents the estimated number of nucleotide substitution per site.

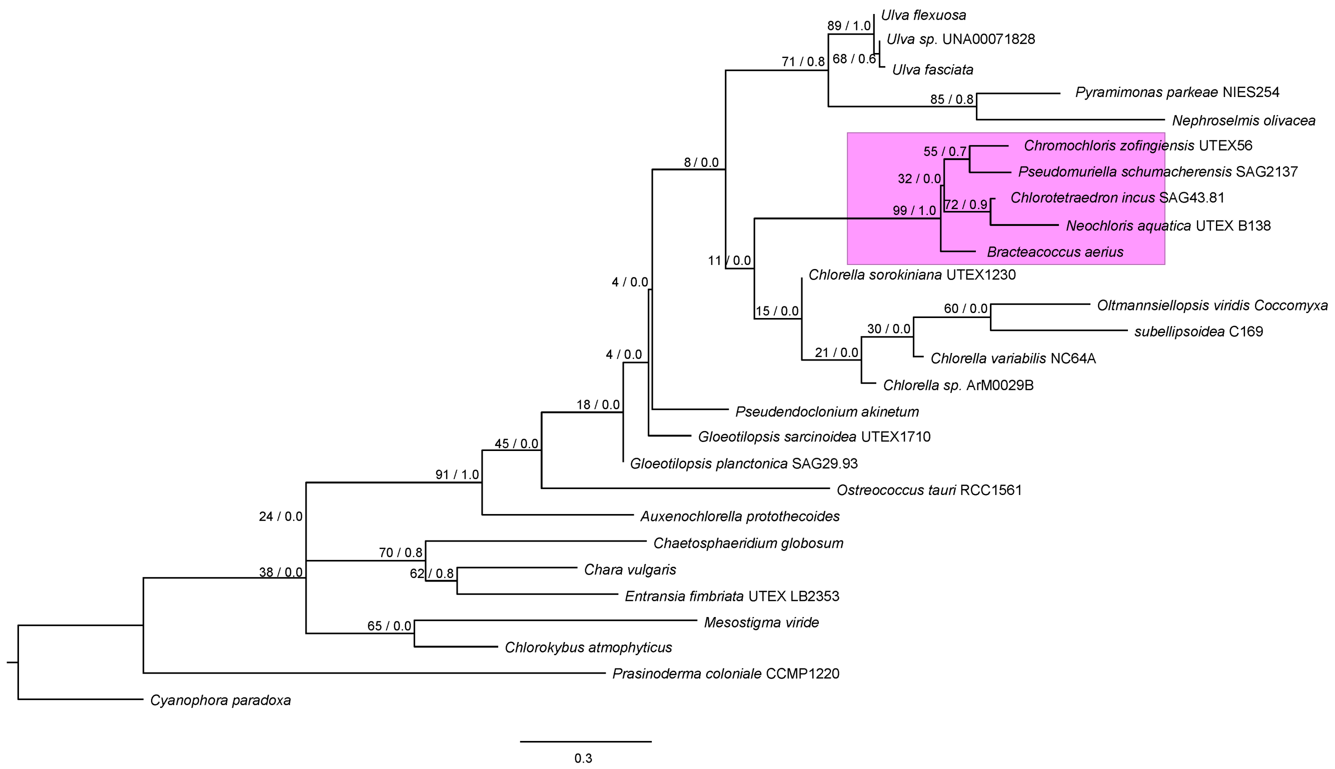

S3 Fig. Z. Maximum-Likelihood tree inferred from plastid *psb*N of algal taxa in Table 1 using a GTR+Γ+I nucleotide substitution model. Bootstrap value from ML and posterior probability from Bayesian inference are shown at the node. The scale bar represents the estimated number of nucleotide substitution per site.

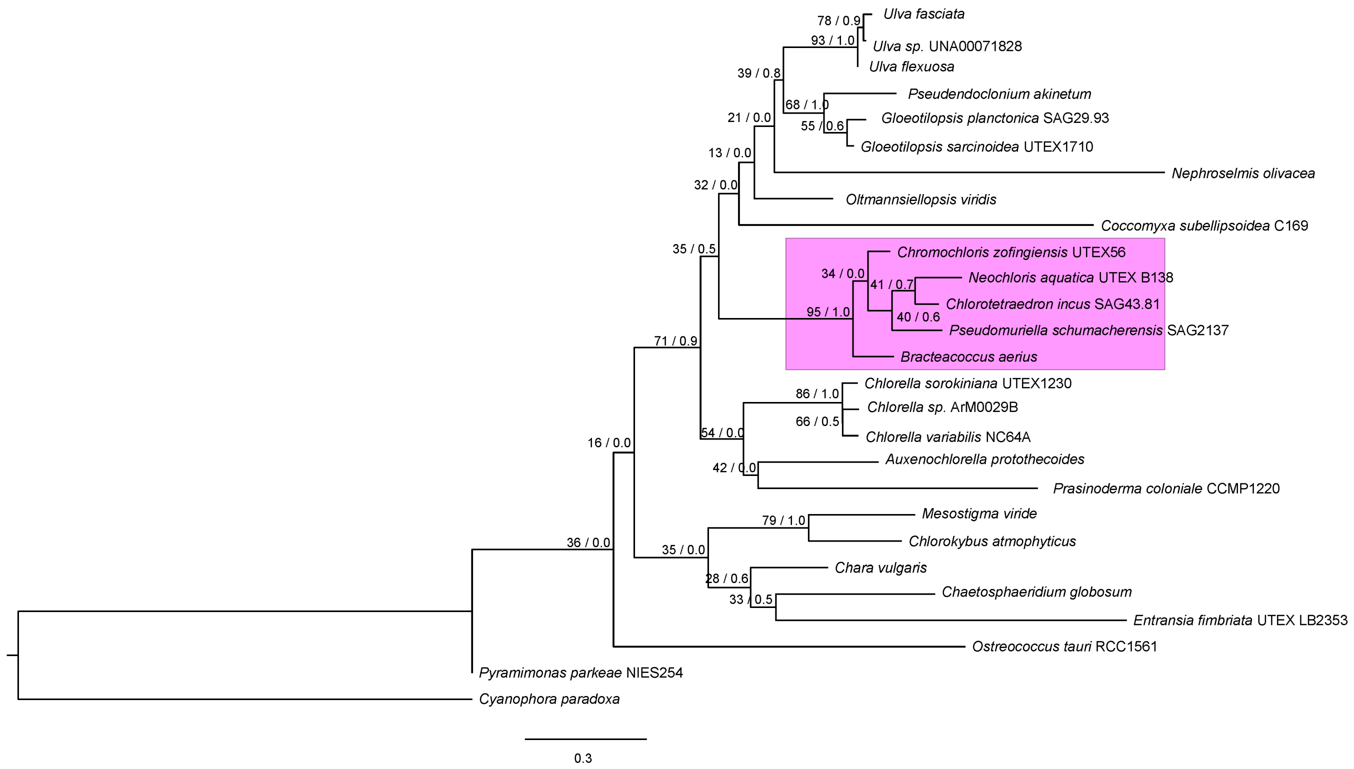

S3 Fig. AA. Maximum-Likelihood tree inferred from plastid *psb*Z of algal taxa in Table 1 using a GTR+Γ+I nucleotide substitution model. Bootstrap value from ML and posterior probability from Bayesian inference are shown at the node. The scale bar represents the estimated number of nucleotide substitution per site.

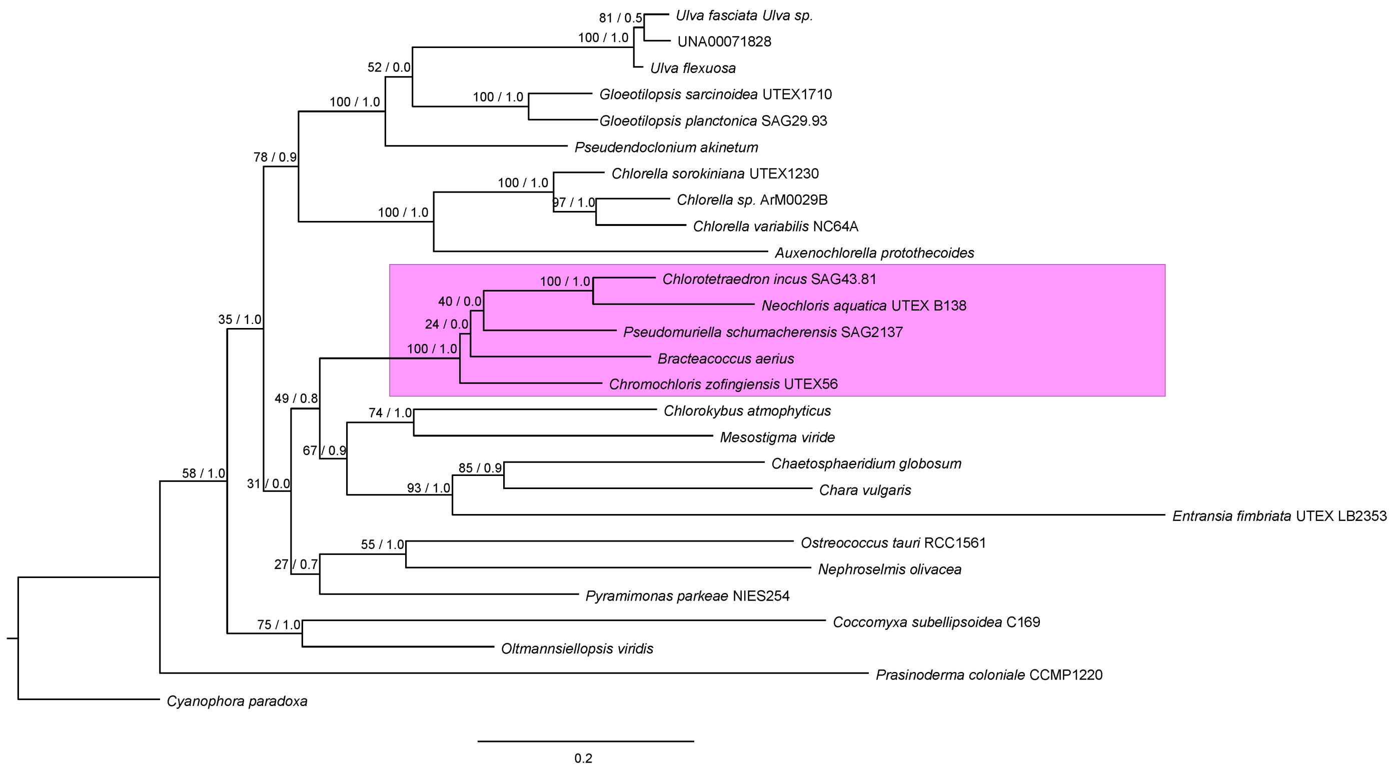

S3 Fig. AB. Maximum-Likelihood tree inferred from plastid *rpl*2 of algal taxa in Table 1 using a GTR+Γ+I nucleotide substitution model. Bootstrap value from ML and posterior probability from Bayesian inference are shown at the node. The scale bar represents the estimated number of nucleotide substitution per site.

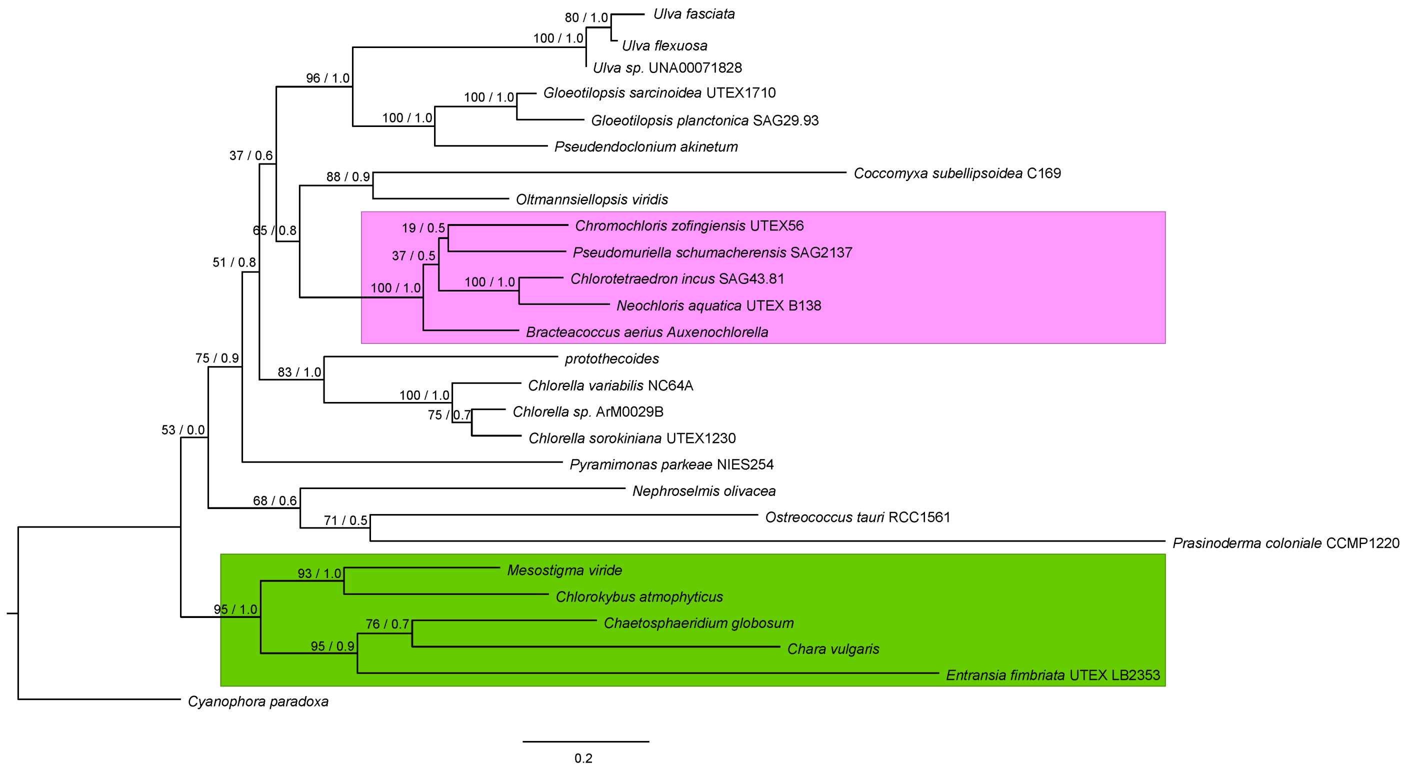

S3 Fig. AC. Maximum-Likelihood tree inferred from plastid *rpl5* of algal taxa in Table 1 using a GTR+Γ+I nucleotide substitution model. Bootstrap value from ML and posterior probability from Bayesian inference are shown at the node. The scale bar represents the estimated number of nucleotide substitution per site.

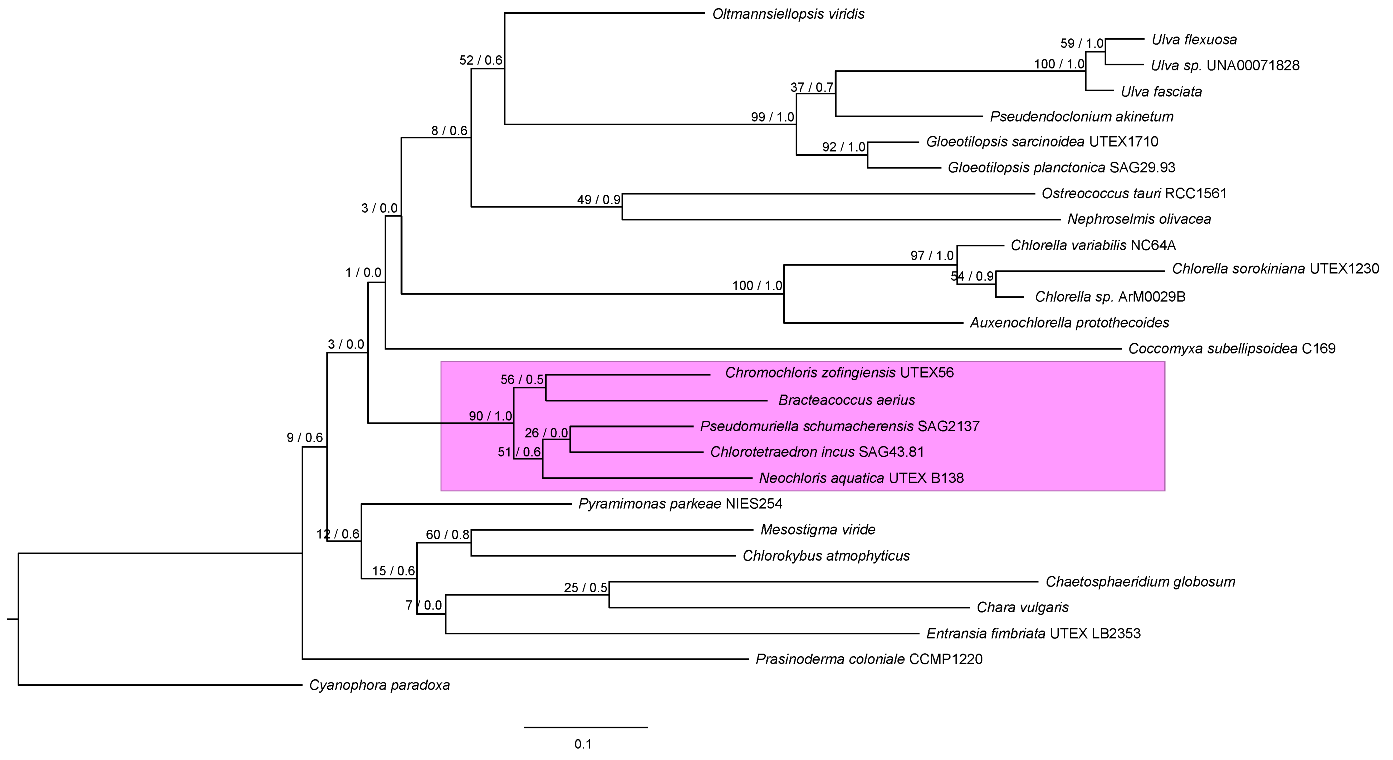

S3 Fig. AD. Maximum-Likelihood tree inferred from plastid *rpl*14 of algal taxa in Table 1 using a GTR+Γ+I nucleotide substitution model. Bootstrap value from ML and posterior probability from Bayesian inference are shown at the node. The scale bar represents the estimated number of nucleotide substitution per site.

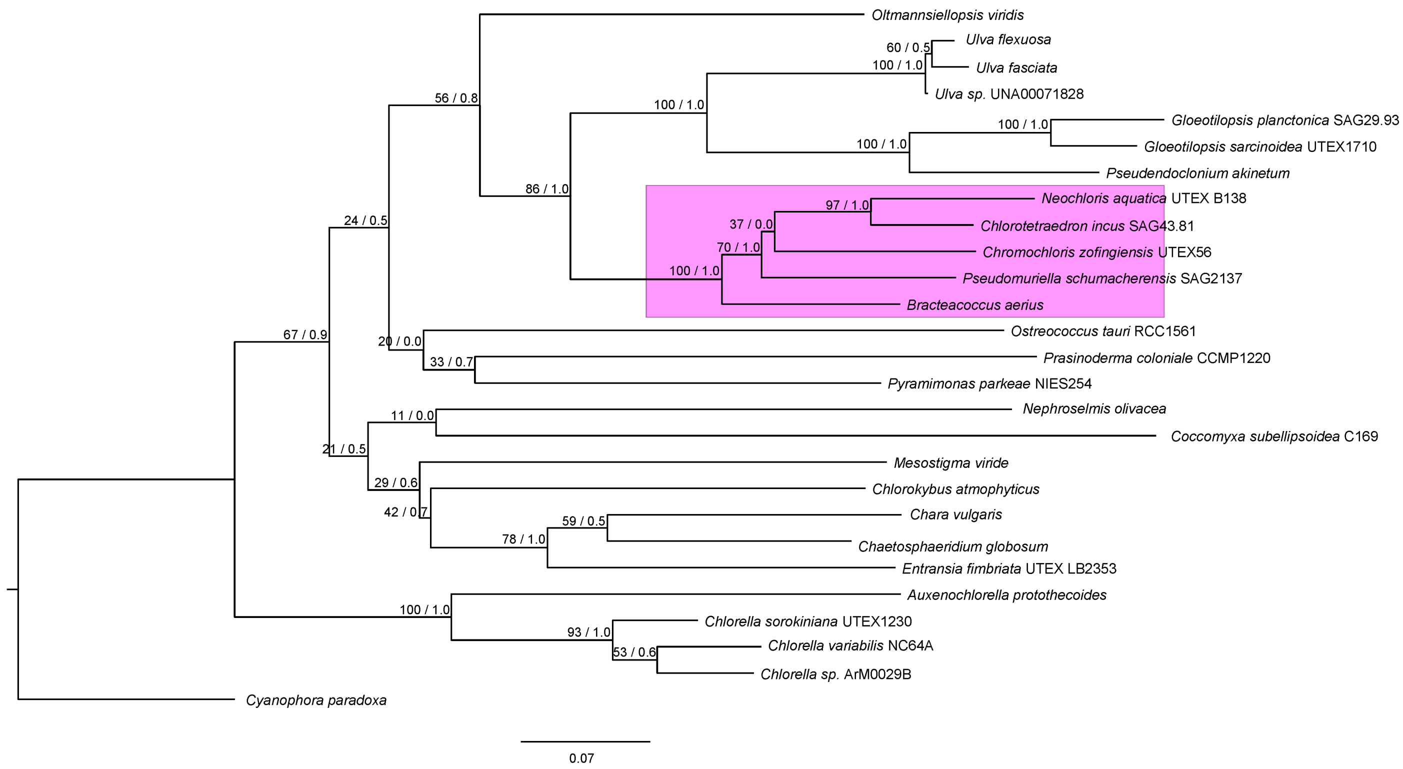

S3 Fig. AE. Maximum-Likelihood tree inferred from plastid *rpl*16 of algal taxa in Table 1 using a GTR+Γ+I nucleotide substitution model. Bootstrap value from ML and posterior probability from Bayesian inference are shown at the node. The scale bar represents the estimated number of nucleotide substitution per site.

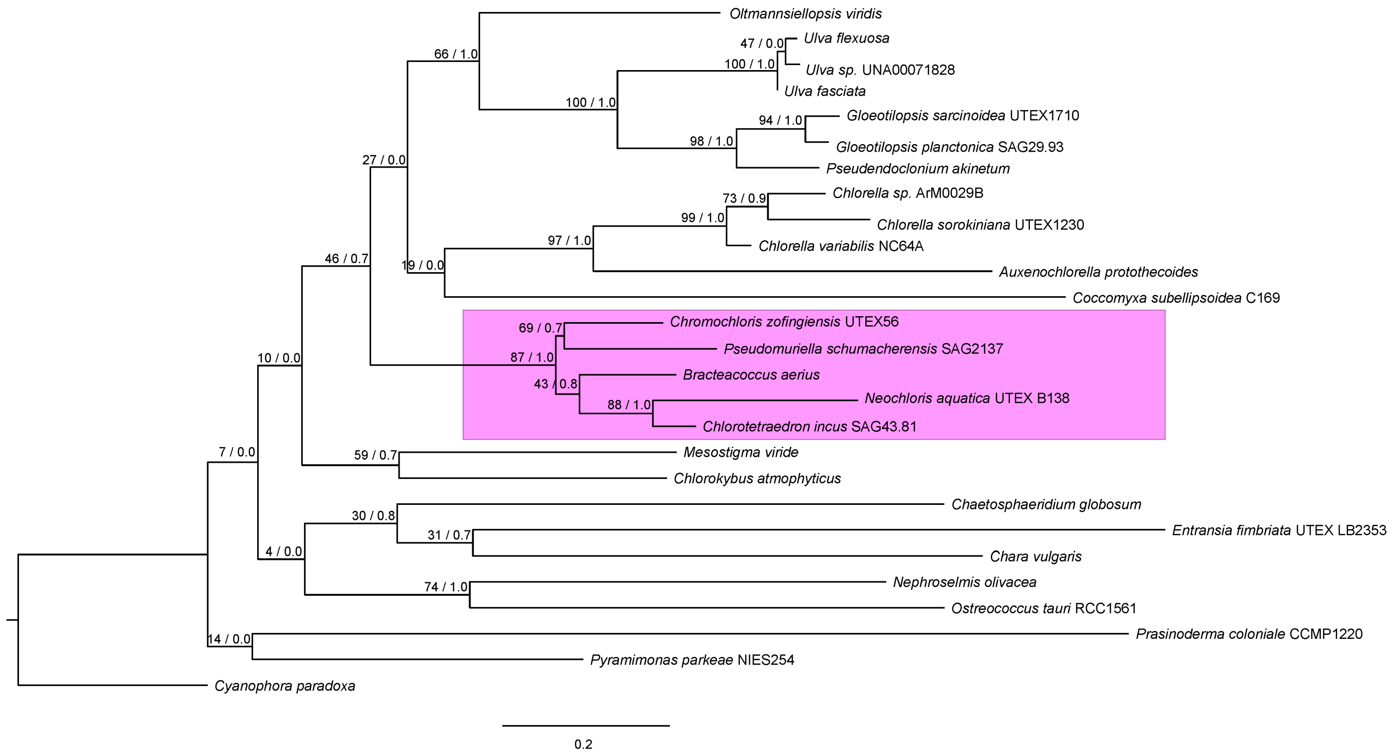

S3 Fig. AF. Maximum-Likelihood tree inferred from plastid *rpl*20 of algal taxa in Table 1 using a GTR+Γ+I nucleotide substitution model. Bootstrap value from ML and posterior probability from Bayesian inference are shown at the node. The scale bar represents the estimated number of nucleotide substitution per site.

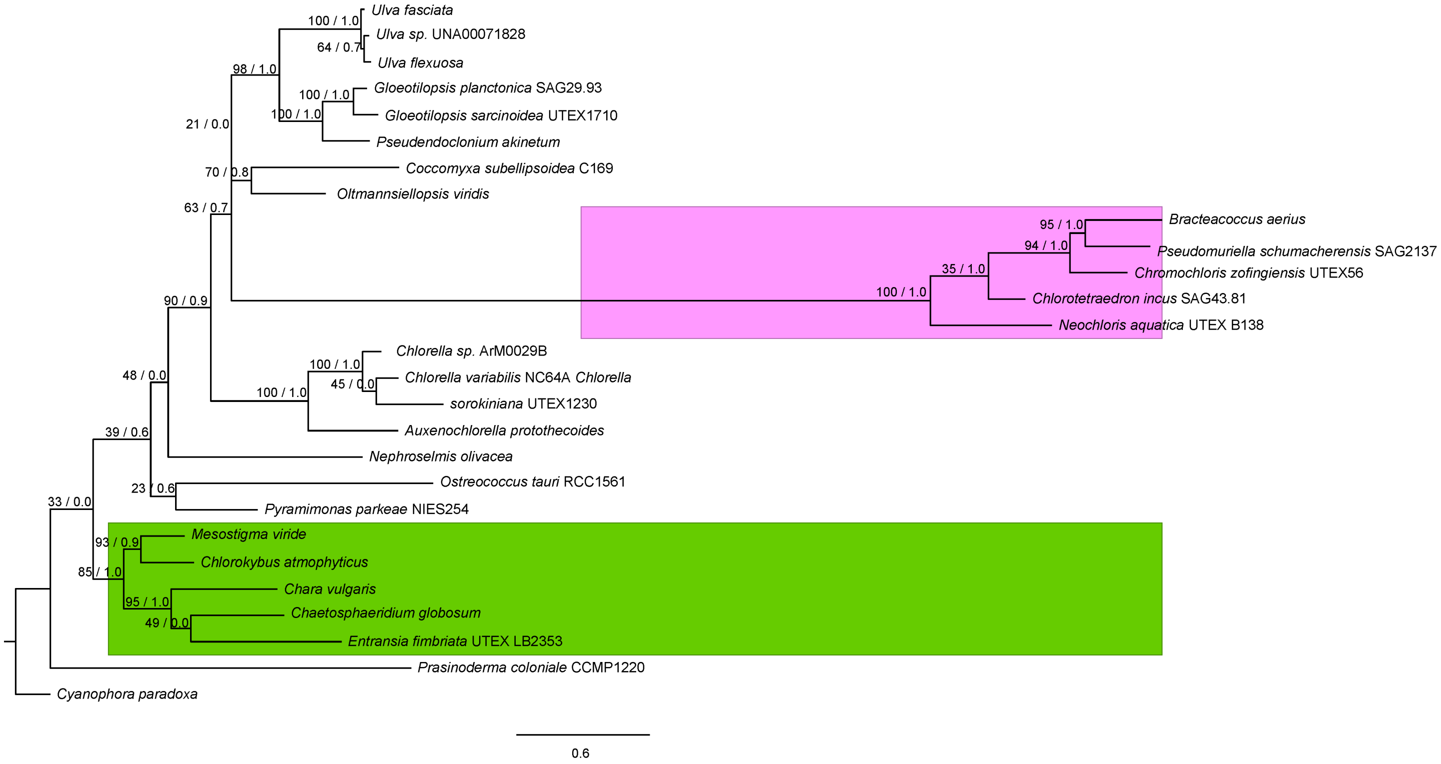

S3 Fig. AG. Maximum-Likelihood tree inferred from plastid *rps*2 of algal taxa in Table 1 using a GTR+Γ+I nucleotide substitution model. Bootstrap value from ML and posterior probability from Bayesian inference are shown at the node. The scale bar represents the estimated number of nucleotide substitution per site.

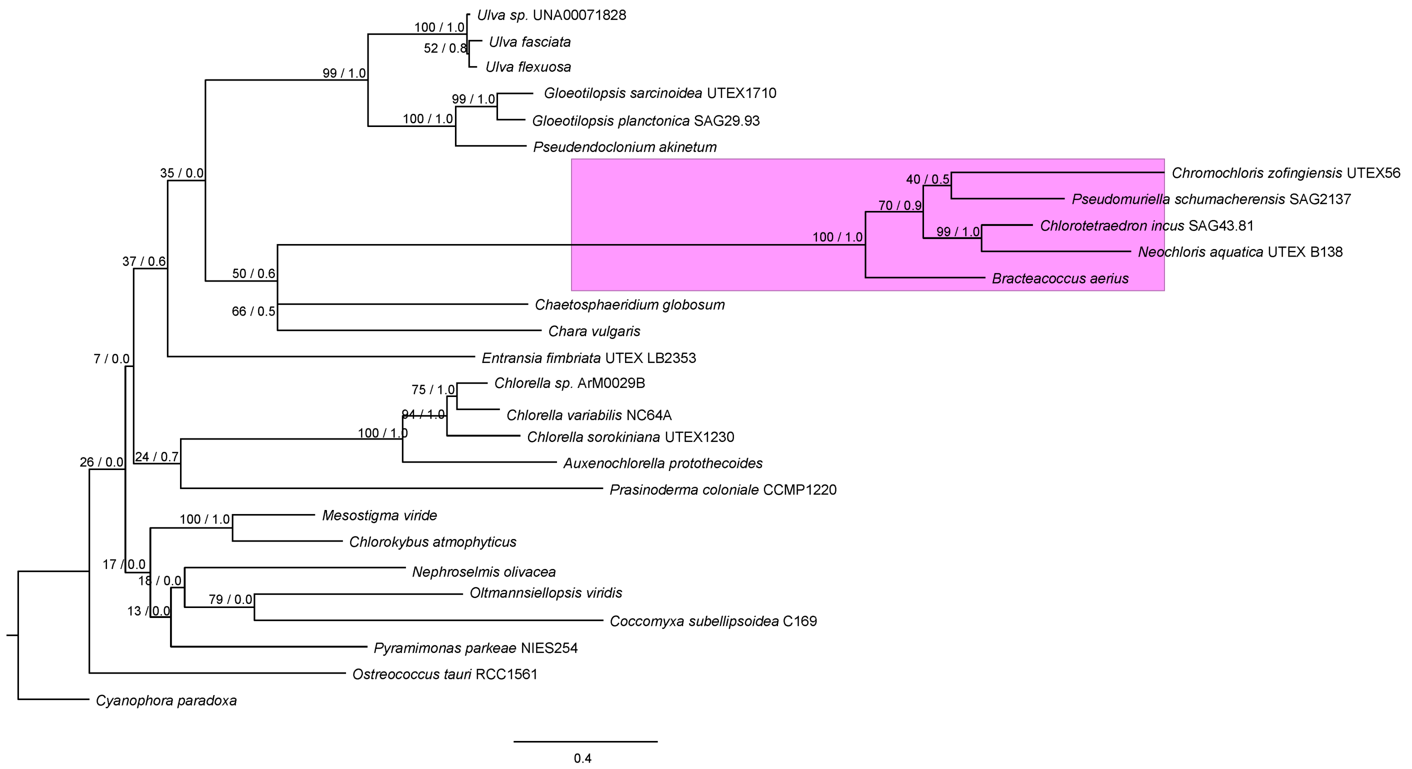

S3 Fig. AH. Maximum-Likelihood tree inferred from plastid *rps*3 of algal taxa in Table 1 using a GTR+Γ+I nucleotide substitution model. Bootstrap value from ML and posterior probability from Bayesian inference are shown at the node. The scale bar represents the estimated number of nucleotide substitution per site.

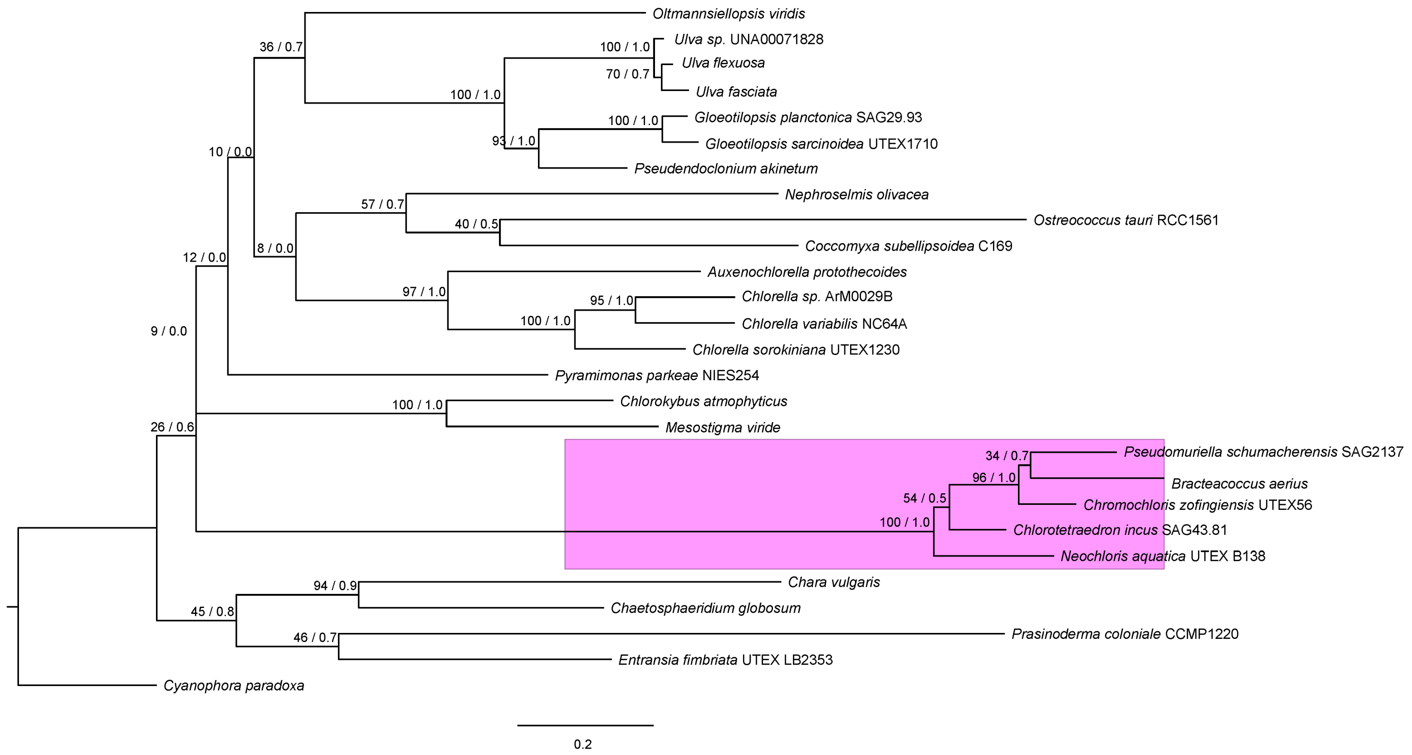

S3 Fig. AI. Maximum-Likelihood tree inferred from plastid *rps*4 of algal taxa in Table 1 using a GTR+Γ+I nucleotide substitution model. Bootstrap value from ML and posterior probability from Bayesian inference are shown at the node. The scale bar represents the estimated number of nucleotide substitution per site.

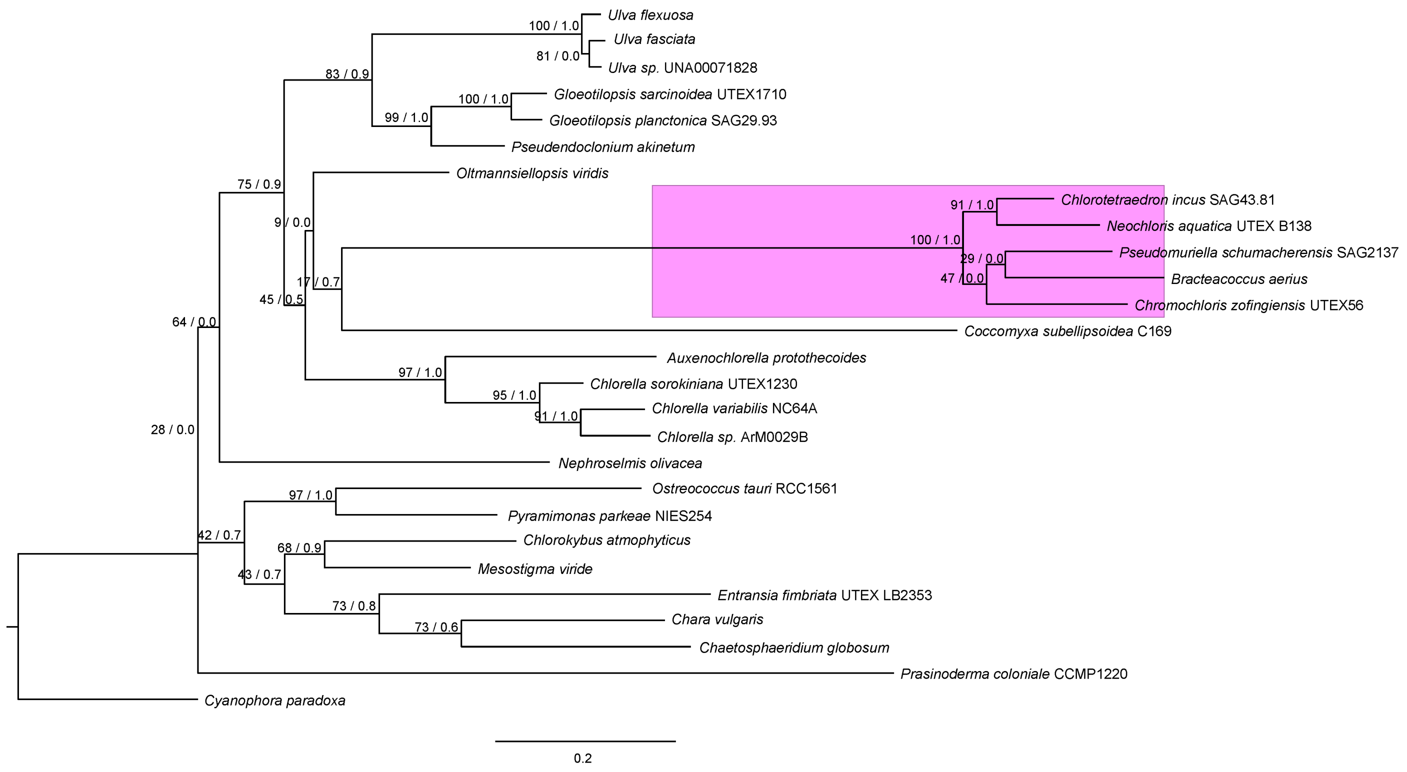

S3 Fig. AJ. Maximum-Likelihood tree inferred from plastid *rps*7 of algal taxa in Table 1 using a GTR+Γ+I nucleotide substitution model. Bootstrap value from ML and posterior probability from Bayesian inference are shown at the node. The scale bar represents the estimated number of nucleotide substitution per site.

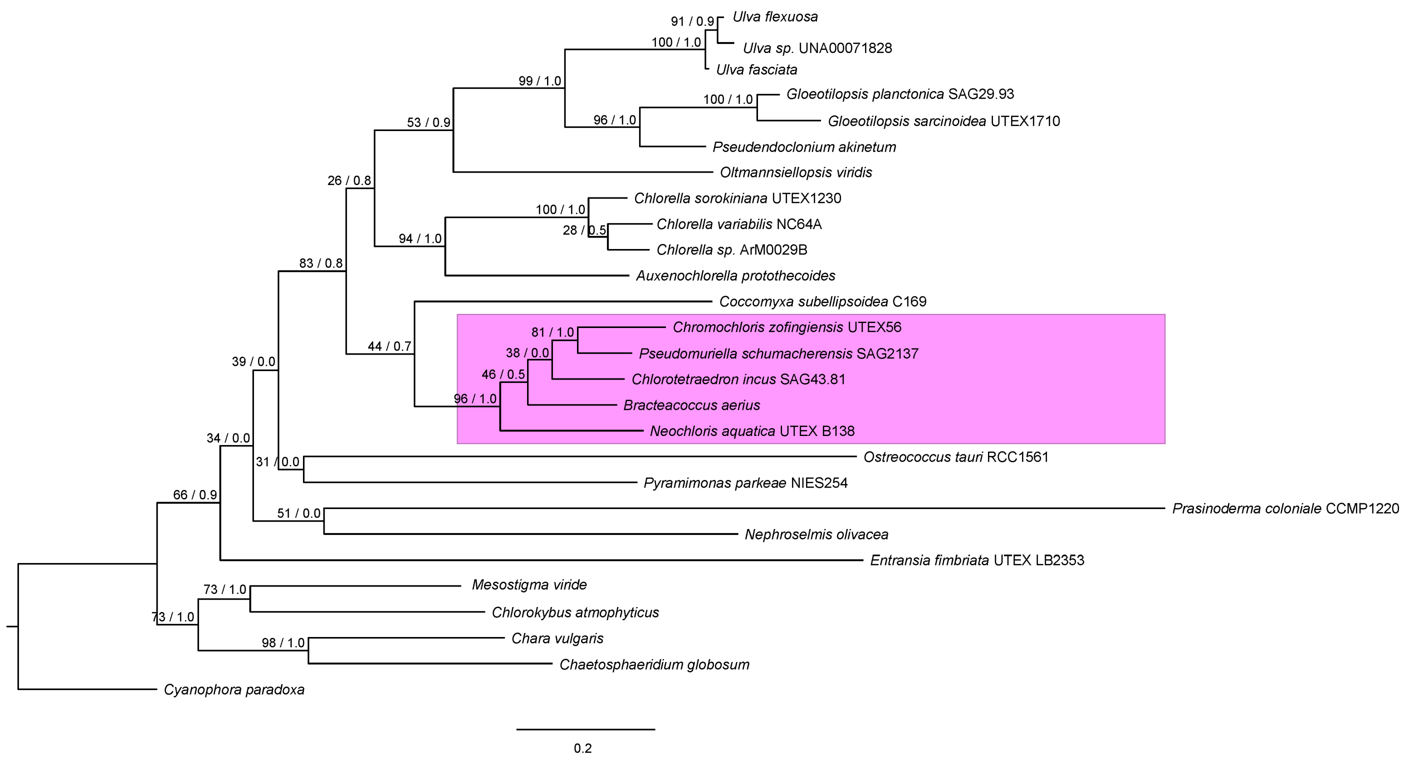

S3 Fig. AK. Maximum-Likelihood tree inferred from plastid *rps*8 of algal taxa in Table 1 using a GTR+Γ+I nucleotide substitution model. Bootstrap value from ML and posterior probability from Bayesian inference are shown at the node. The scale bar represents the estimated number of nucleotide substitution per site.

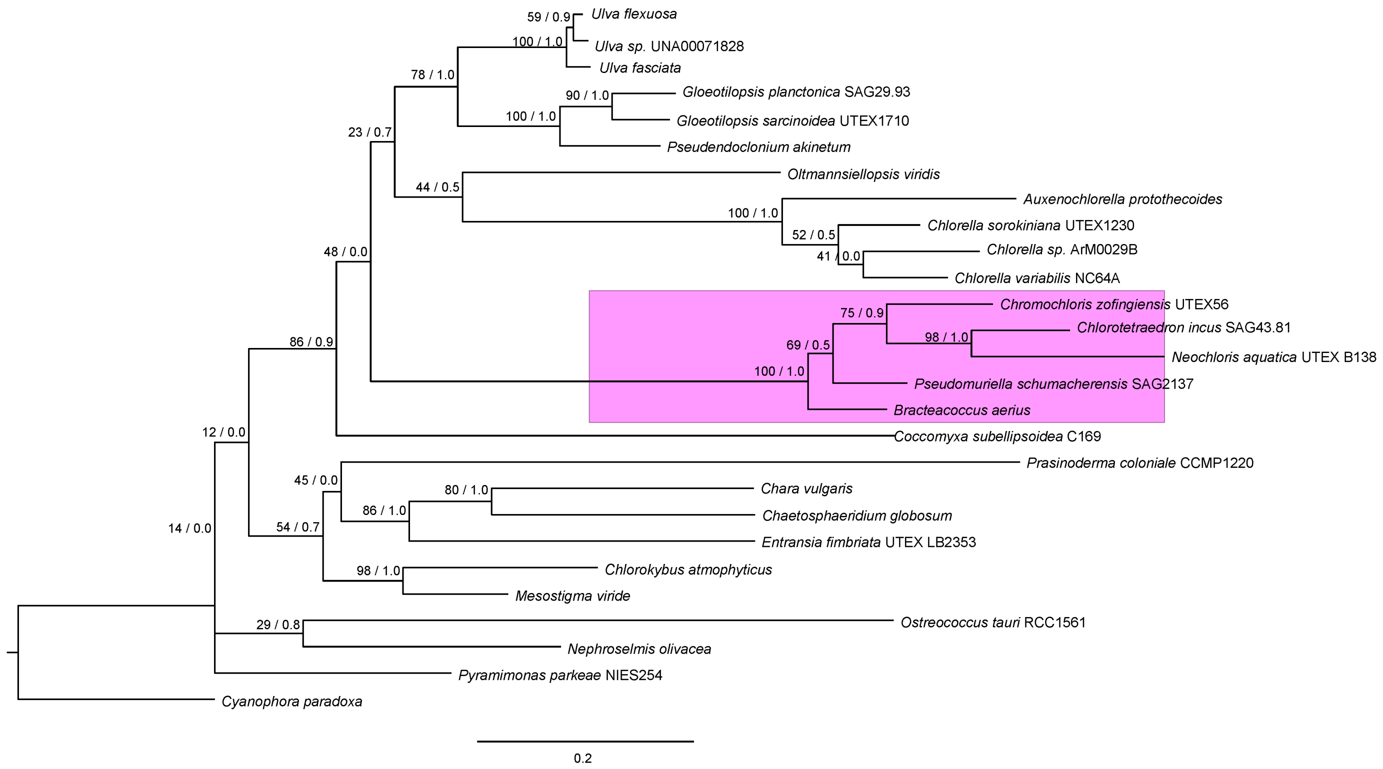

S3 Fig. AL. Maximum-Likelihood tree inferred from plastid *rps*11 of algal taxa in Table 1 using a GTR+Γ+I nucleotide substitution model. Bootstrap value from ML and posterior probability from Bayesian inference are shown at the node. The scale bar represents the estimated number of nucleotide substitution per site.

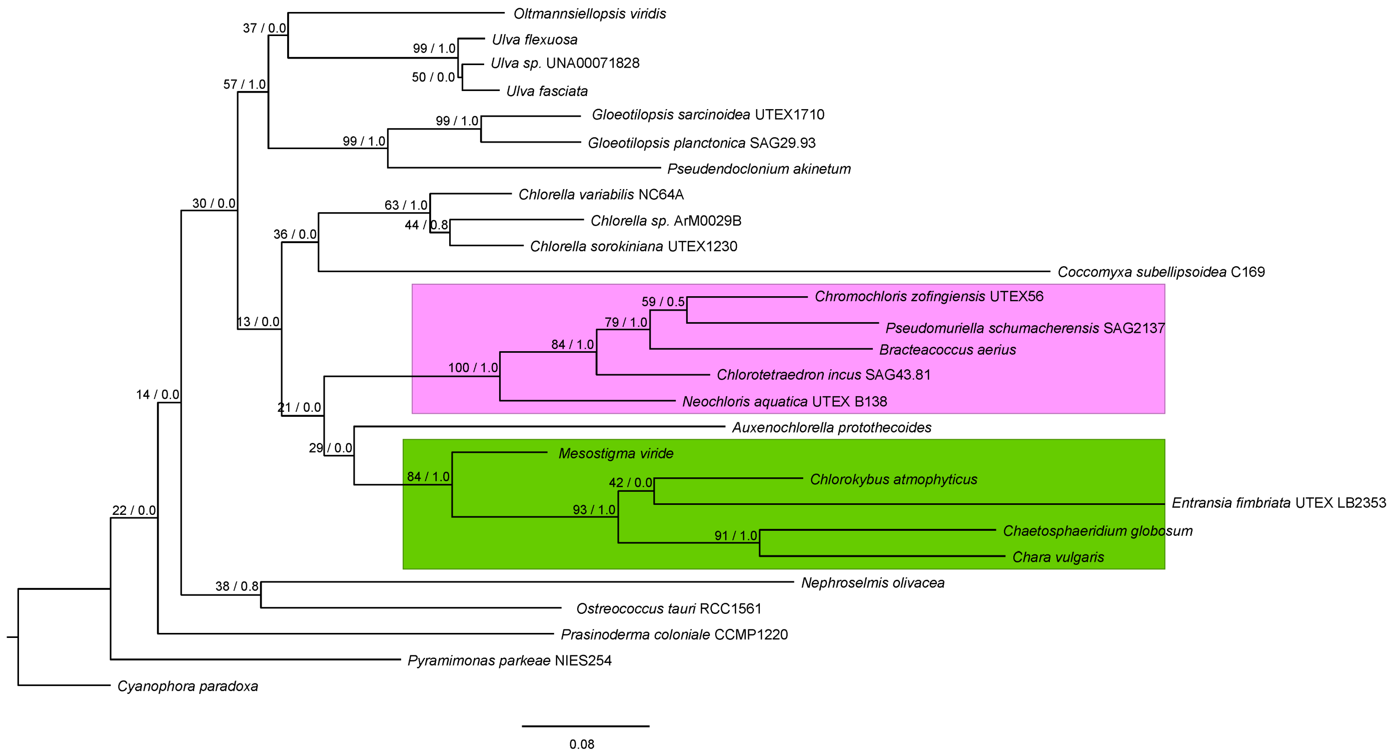

S3 Fig. AM. Maximum-Likelihood tree inferred from plastid *rps*12 of algal taxa in Table 1 using a GTR+Γ+I nucleotide substitution model. Bootstrap value from ML and posterior probability from Bayesian inference are shown at the node. The scale bar represents the estimated number of nucleotide substitution per site.

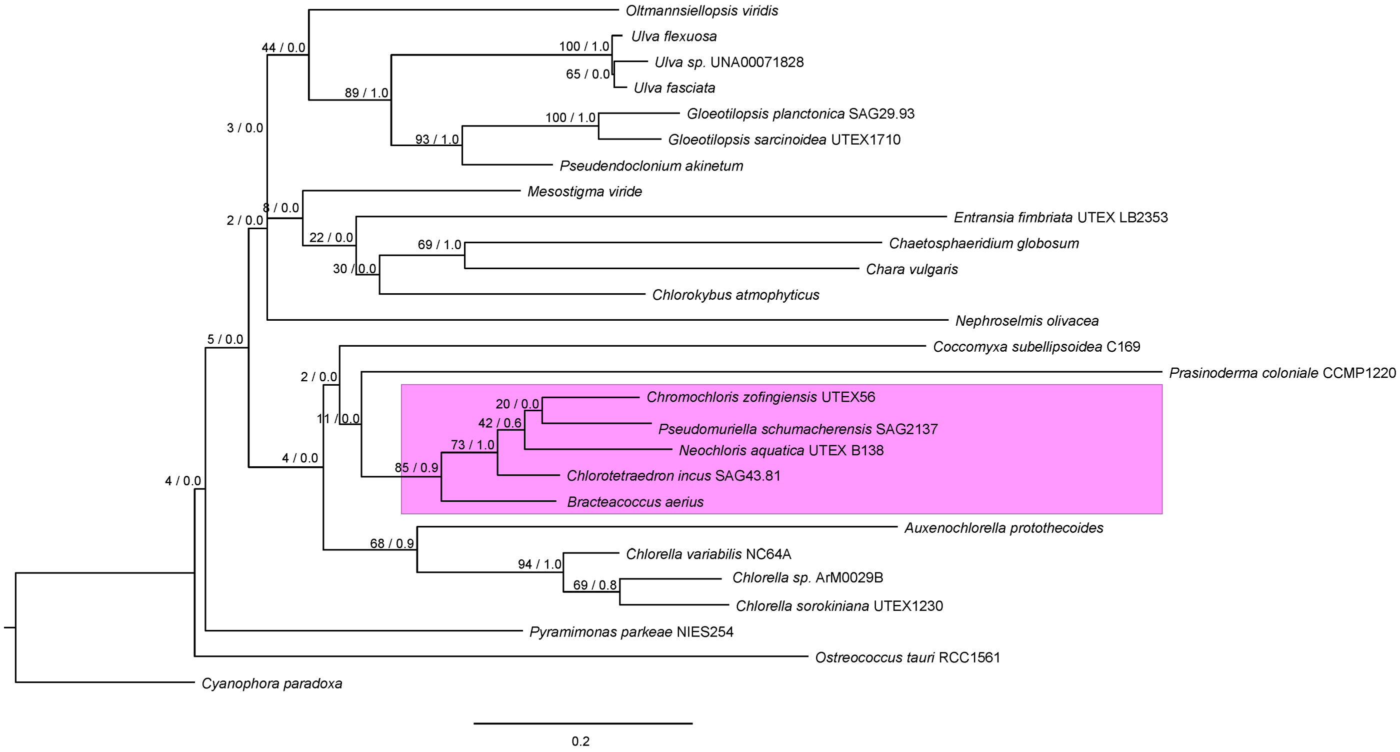

S3 Fig. AN. Maximum-Likelihood tree inferred from plastid *rps*14 of algal taxa in Table 1 using a GTR+Γ+I nucleotide substitution model. Bootstrap value from ML and posterior probability from Bayesian inference are shown at the node. The scale bar represents the estimated number of nucleotide substitution per site.

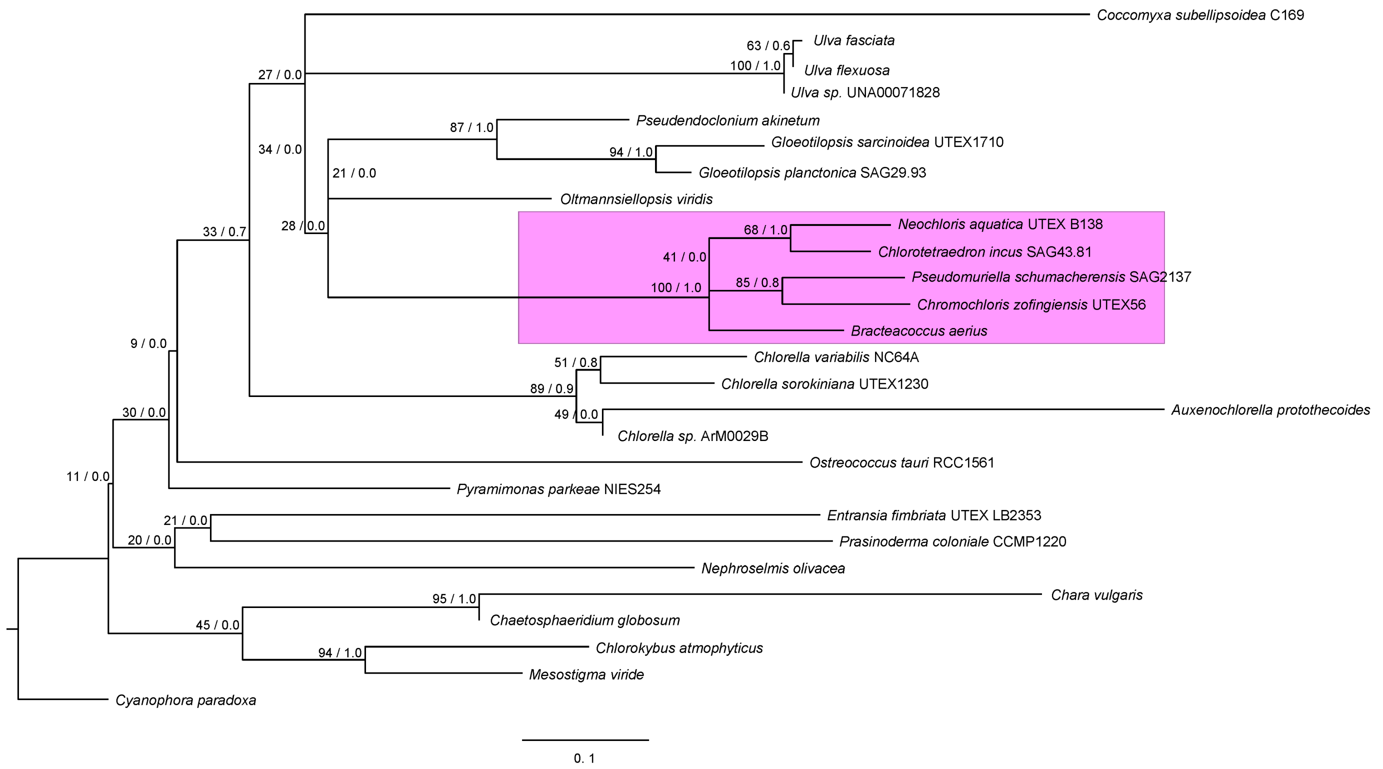

S3 Fig. AO. Maximum-Likelihood tree inferred from plastid *rps*18 of algal taxa in Table 1 using a GTR+Γ+I nucleotide substitution model. Bootstrap value from ML and posterior probability from Bayesian inference are shown at the node. The scale bar represents the estimated number of nucleotide substitution per site.

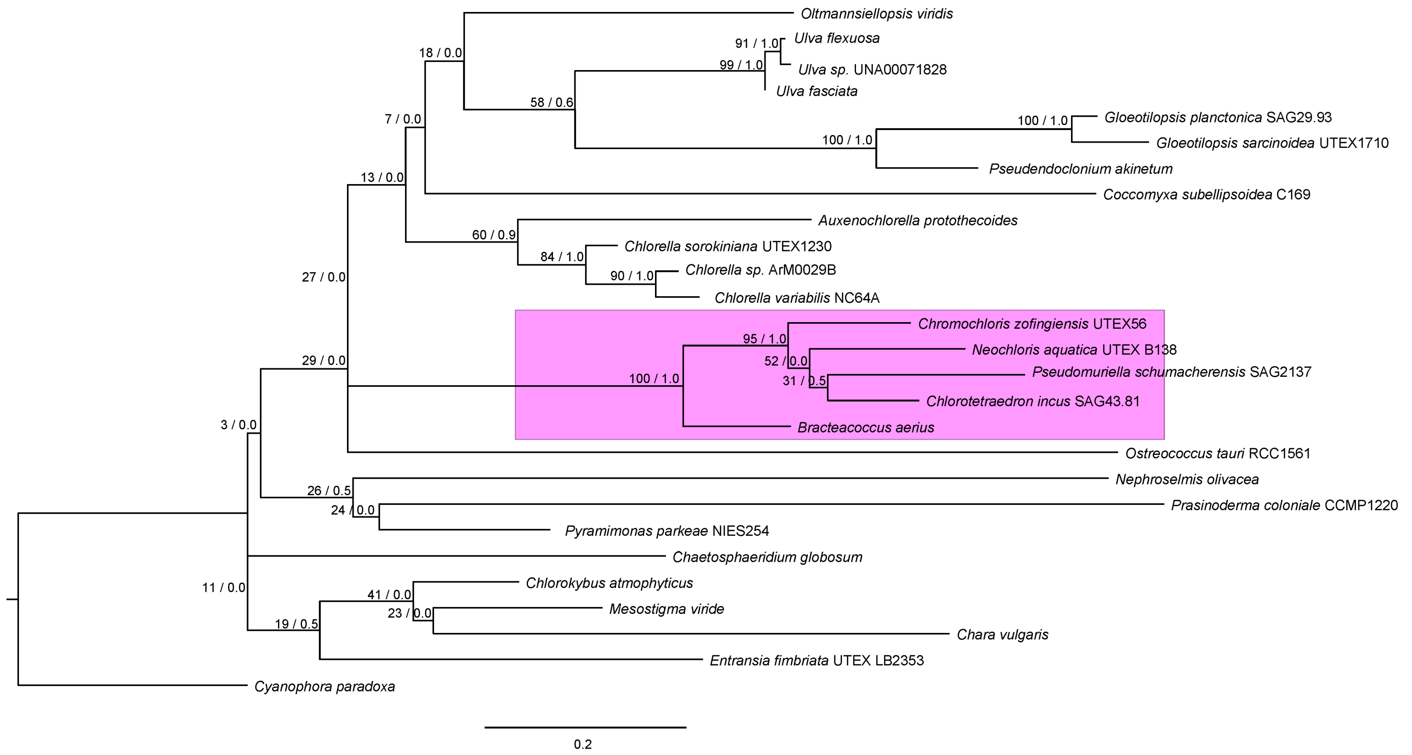

S3 Fig. AP. Maximum-Likelihood tree inferred from plastid *rps*19 of algal taxa in Table 1 using a GTR+Γ+I nucleotide substitution model. Bootstrap value from ML and posterior probability from Bayesian inference are shown at the node. The scale bar represents the estimated number of nucleotide substitution per site.

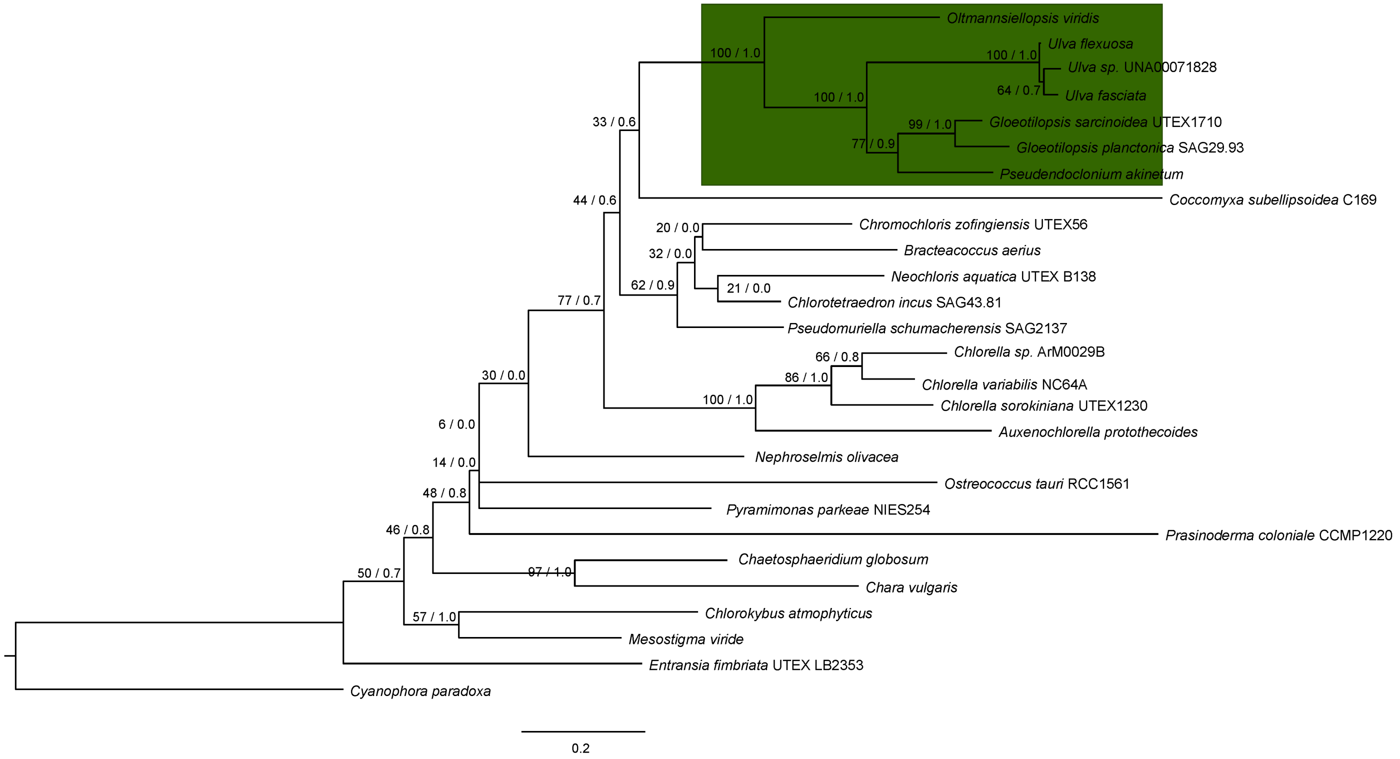

S3 Fig. AQ. Maximum-Likelihood tree inferred from plastid *ycf*3 of algal taxa in Table 1 using a GTR+Γ+I nucleotide substitution model. Bootstrap value from ML and posterior probability from Bayesian inference are shown at the node. The scale bar represents the estimated number of nucleotide substitution per site.

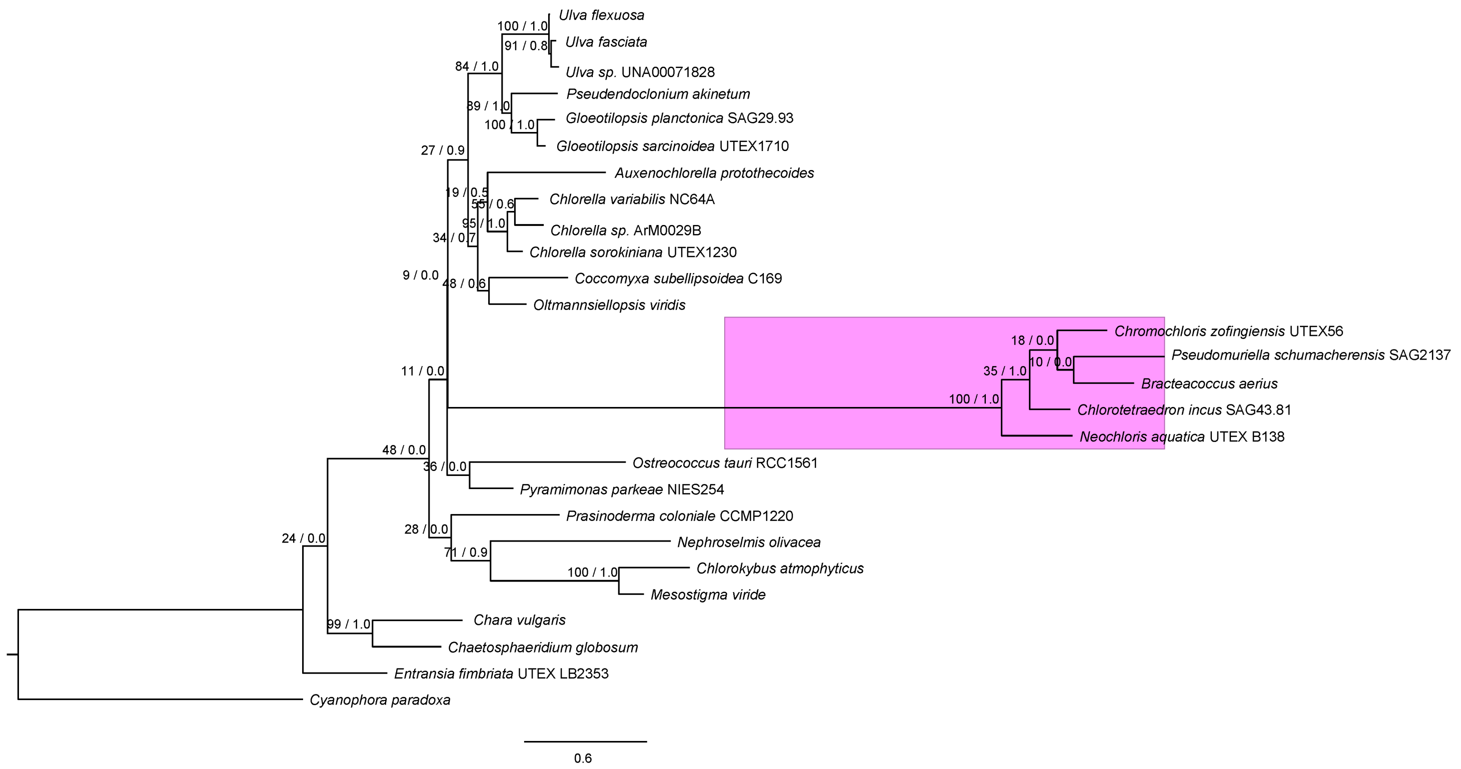

S3 Fig. AR. Maximum-Likelihood tree inferred from plastid *clp*P of algal taxa in Table 1 using a GTR+Γ+I nucleotide substitution model. Bootstrap value from ML and posterior probability from Bayesian inference are shown at the node. The scale bar represents the estimated number of nucleotide substitution per site.

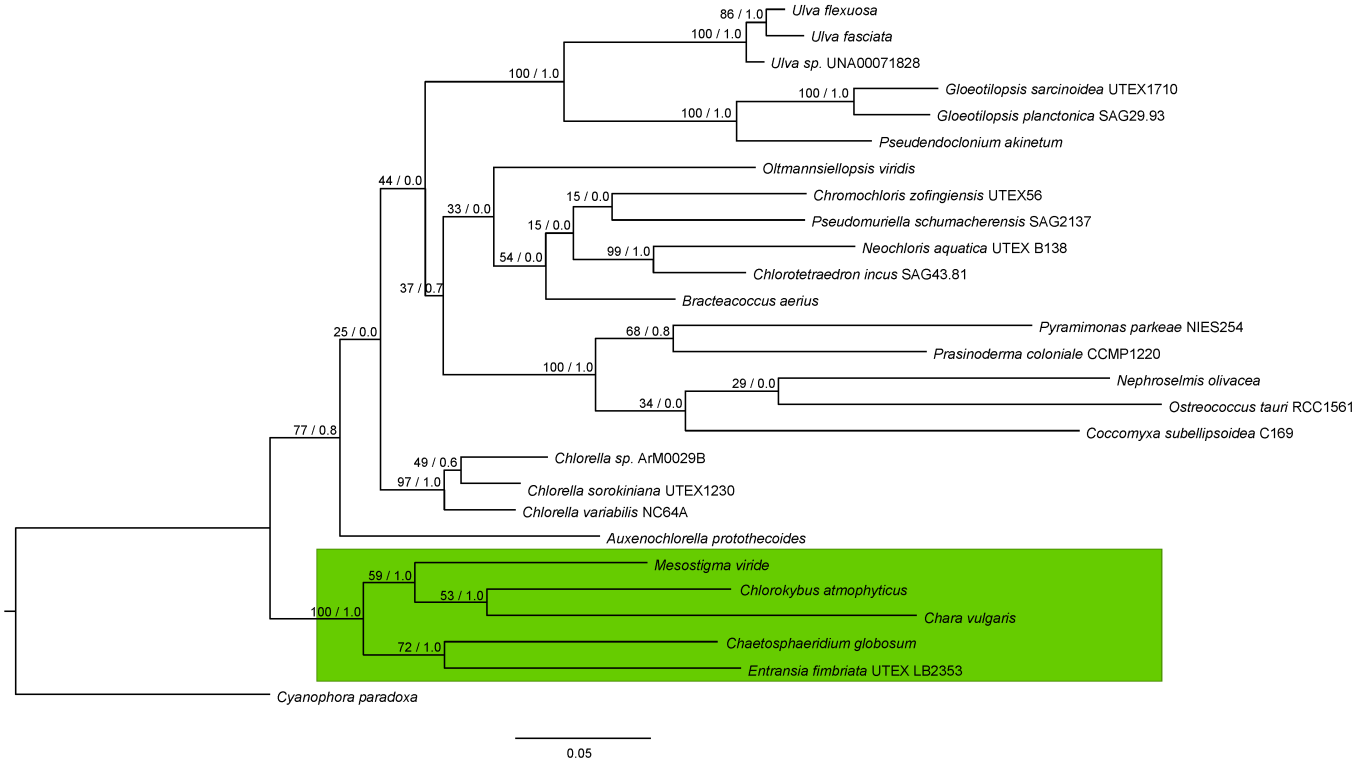

S3 Fig. AS. Maximum-Likelihood tree inferred from plastid *rbc*L of algal taxa in Table 1 using a GTR+Γ+I nucleotide substitution model. Bootstrap value from ML and posterior probability from Bayesian inference are shown at the node. The scale bar represents the estimated number of nucleotide substitution per site.

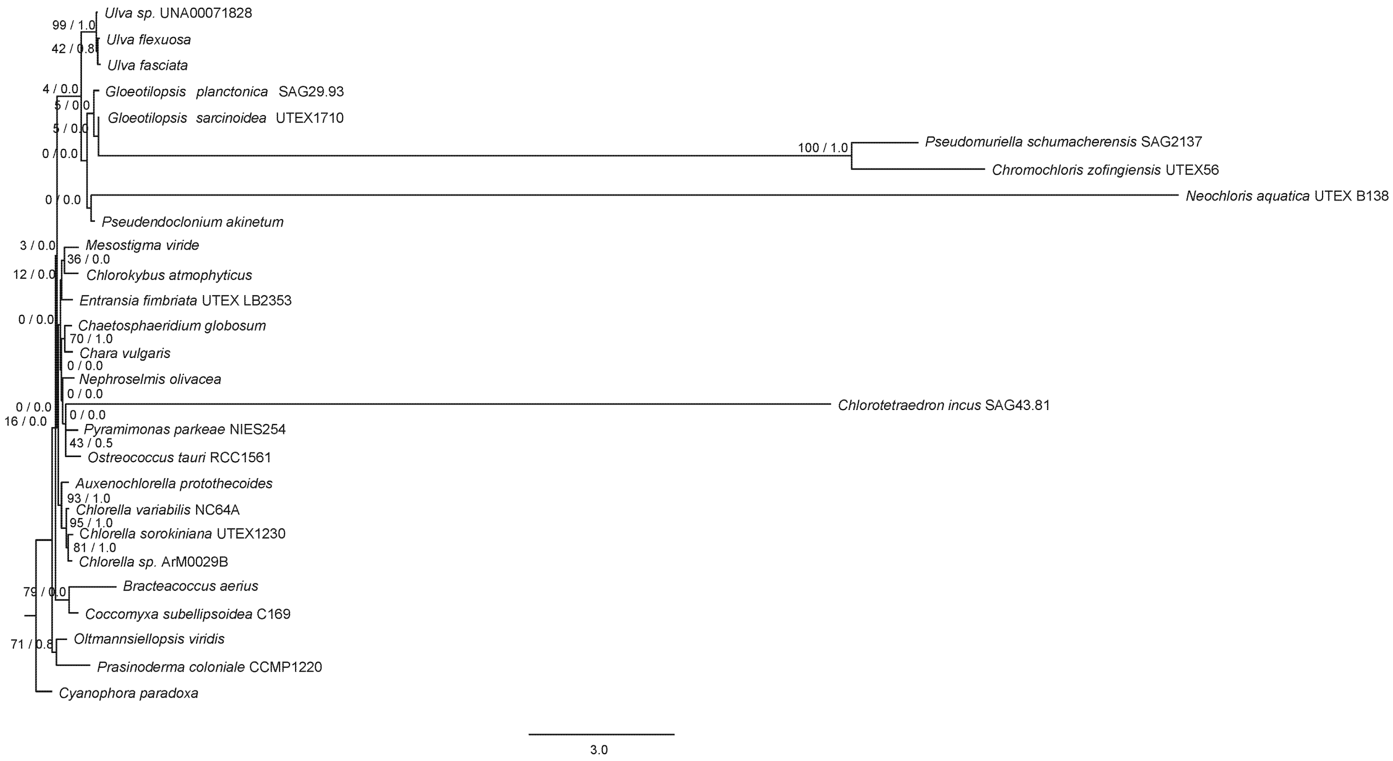

S3 Fig. AT. Maximum-Likelihood tree inferred from mitochondrial *cob* of algal taxa in Table 1 using a GTR+Γ+I nucleotide substitution model. Bootstrap value from ML and posterior probability from Bayesian inference are shown at the node. The scale bar represents the estimated number of nucleotide substitution per site.

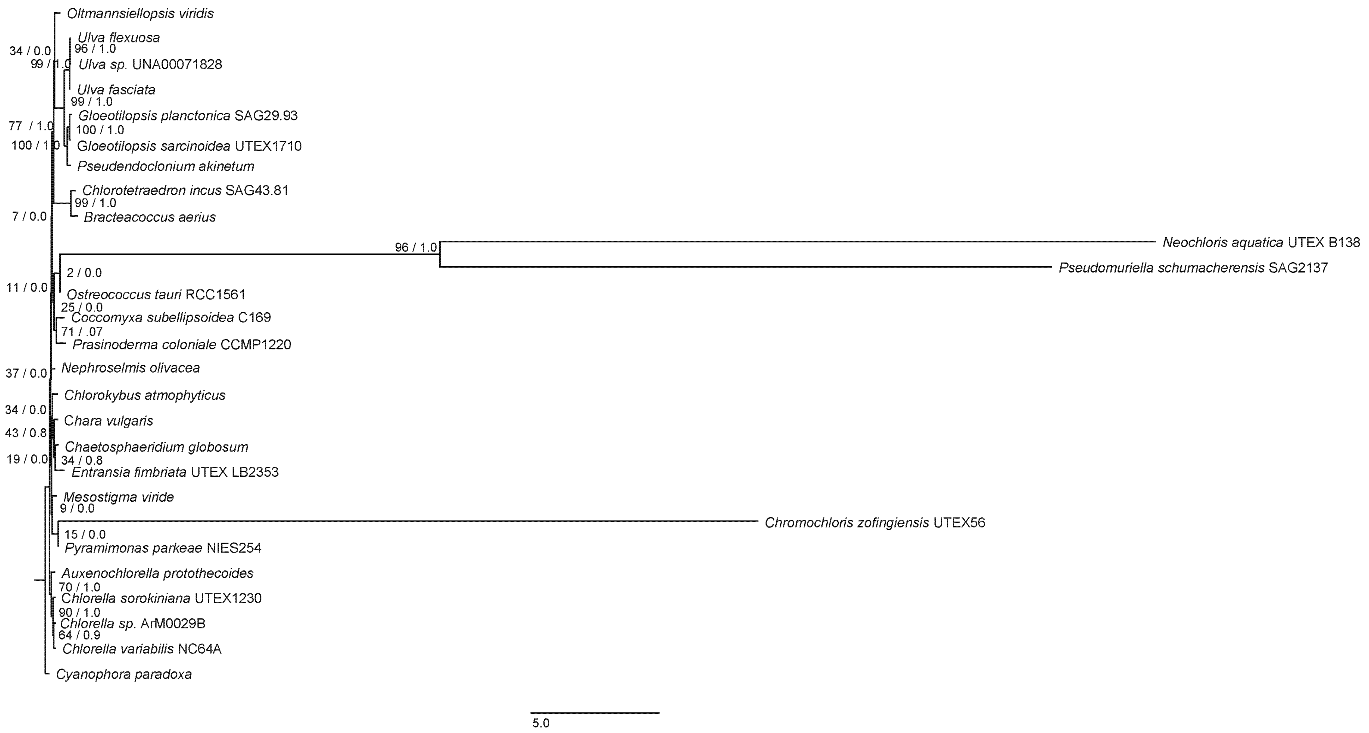

S3 Fig. AU. Maximum-Likelihood tree inferred from mitochondrial *cox*1 of algal taxa in Table 1 using a GTR+Γ+I nucleotide substitution model. Bootstrap value from ML and posterior probability from Bayesian inference are shown at the node. The scale bar represents the estimated number of nucleotide substitution per site.

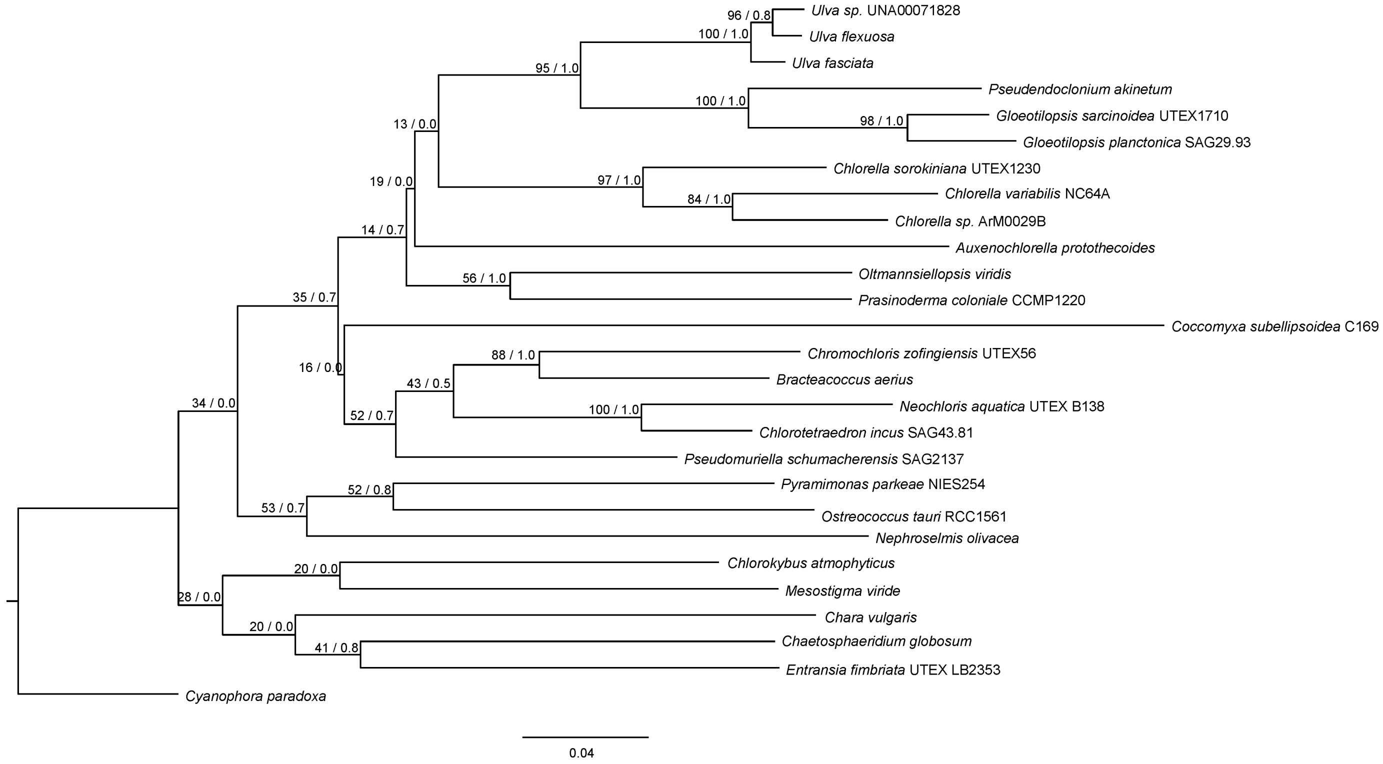

S3 Fig. AV. Maximum-Likelihood tree inferred from plastid *pet*B of algal taxa in Table 1 using a GTR+Γ+I nucleotide substitution model. Bootstrap value from ML and posterior probability from Bayesian inference are shown at the node. The scale bar represents the estimated number of nucleotide substitution per site.

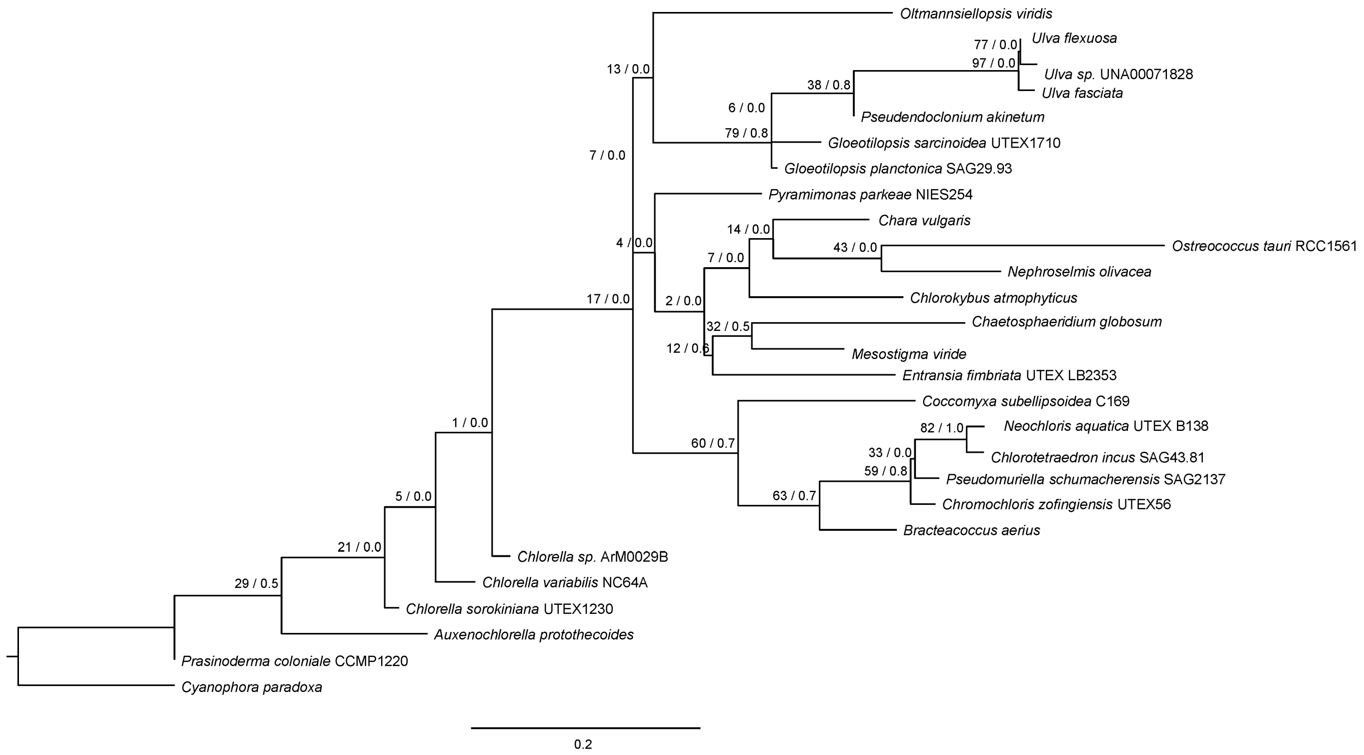

S3 Fig. AW. Maximum-Likelihood tree inferred from plastid *pet*G of algal taxa in Table 1 using a GTR+Γ+I nucleotide substitution model. Bootstrap value from ML and posterior probability from Bayesian inference are shown at the node. The scale bar represents the estimated number of nucleotide substitution per site.

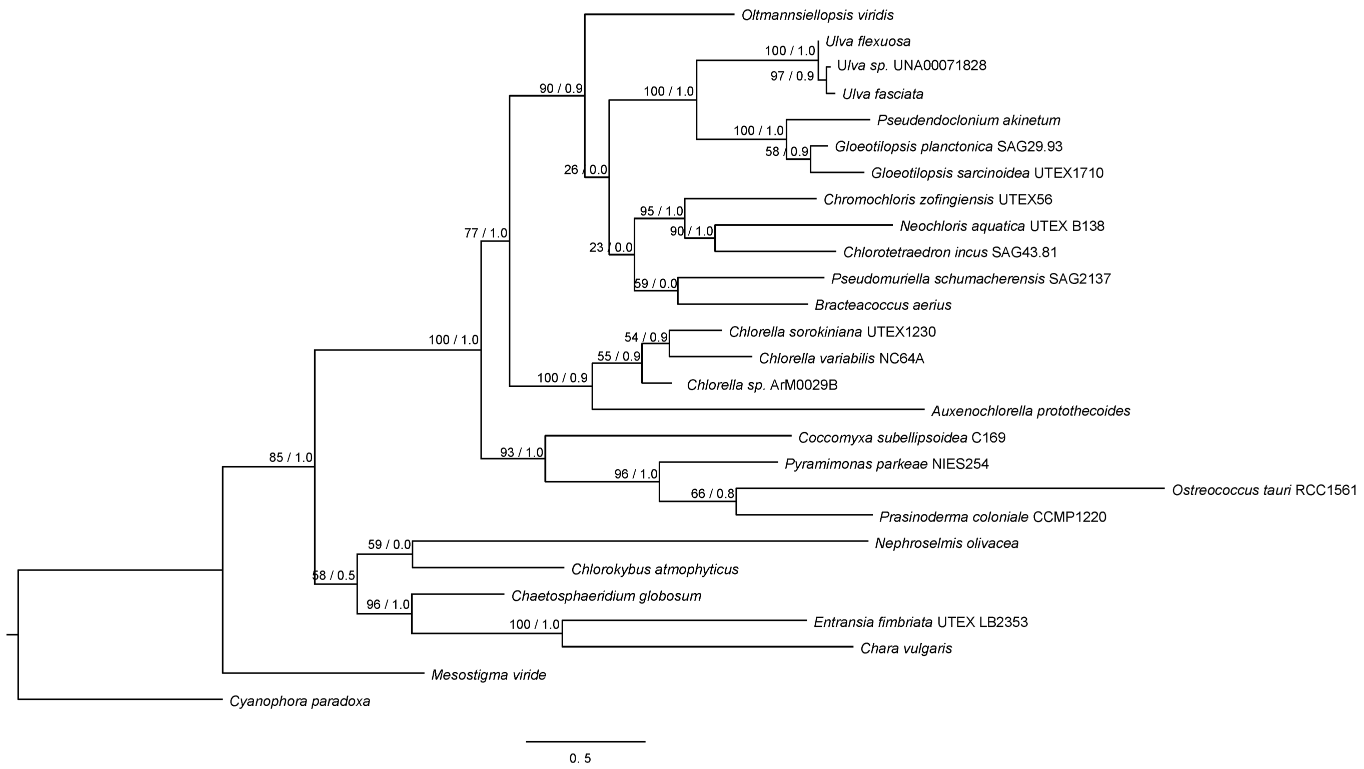

S3 Fig. AX. Maximum-Likelihood tree inferred from plastid *psb*A of algal taxa in Table 1 using a GTR+Γ+I nucleotide substitution model. Bootstrap value from ML and posterior probability from Bayesian inference are shown at the node. The scale bar represents the estimated number of nucleotide substitution per site.

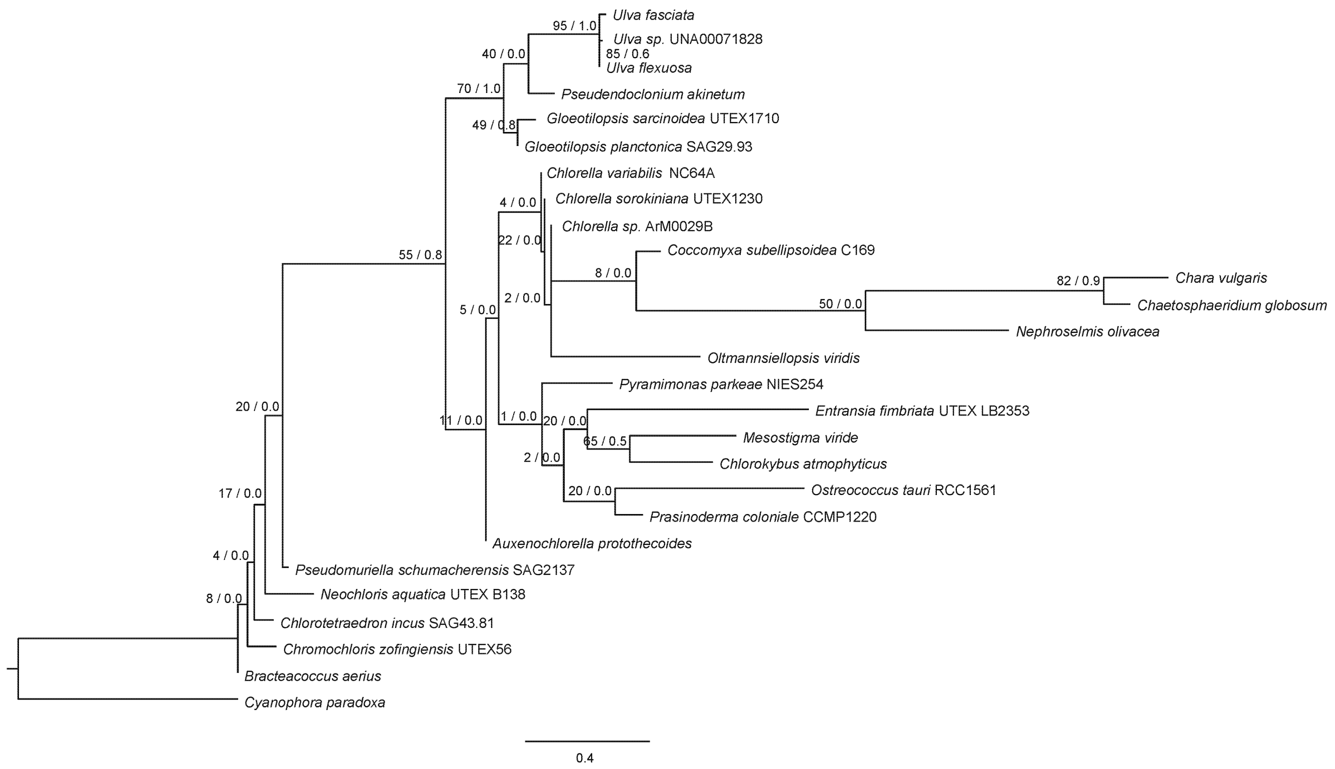

S3 Fig. AY. Maximum-Likelihood tree inferred from plastid *psb*F of algal taxa in Table 1 using a GTR+Γ+I nucleotide substitution model. Bootstrap value from ML and posterior probability from Bayesian inference are shown at the node. The scale bar represents the estimated number of nucleotide substitution per site.

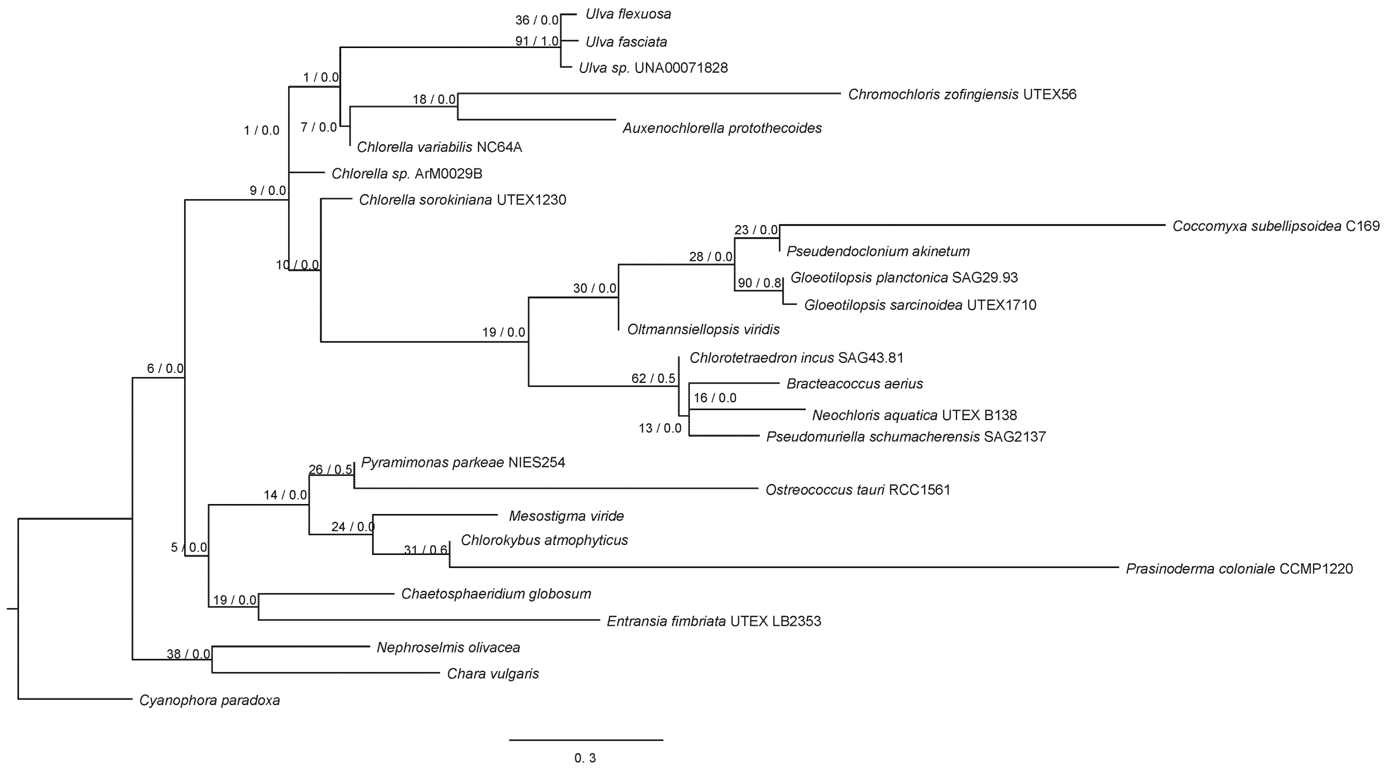

S3 Fig. AZ. Maximum-Likelihood tree inferred from plastid *psb*I of algal taxa in Table 1 using a GTR+Γ+I nucleotide substitution model. Bootstrap value from ML and posterior probability from Bayesian inference are shown at the node. The scale bar represents the estimated number of nucleotide substitution per site.

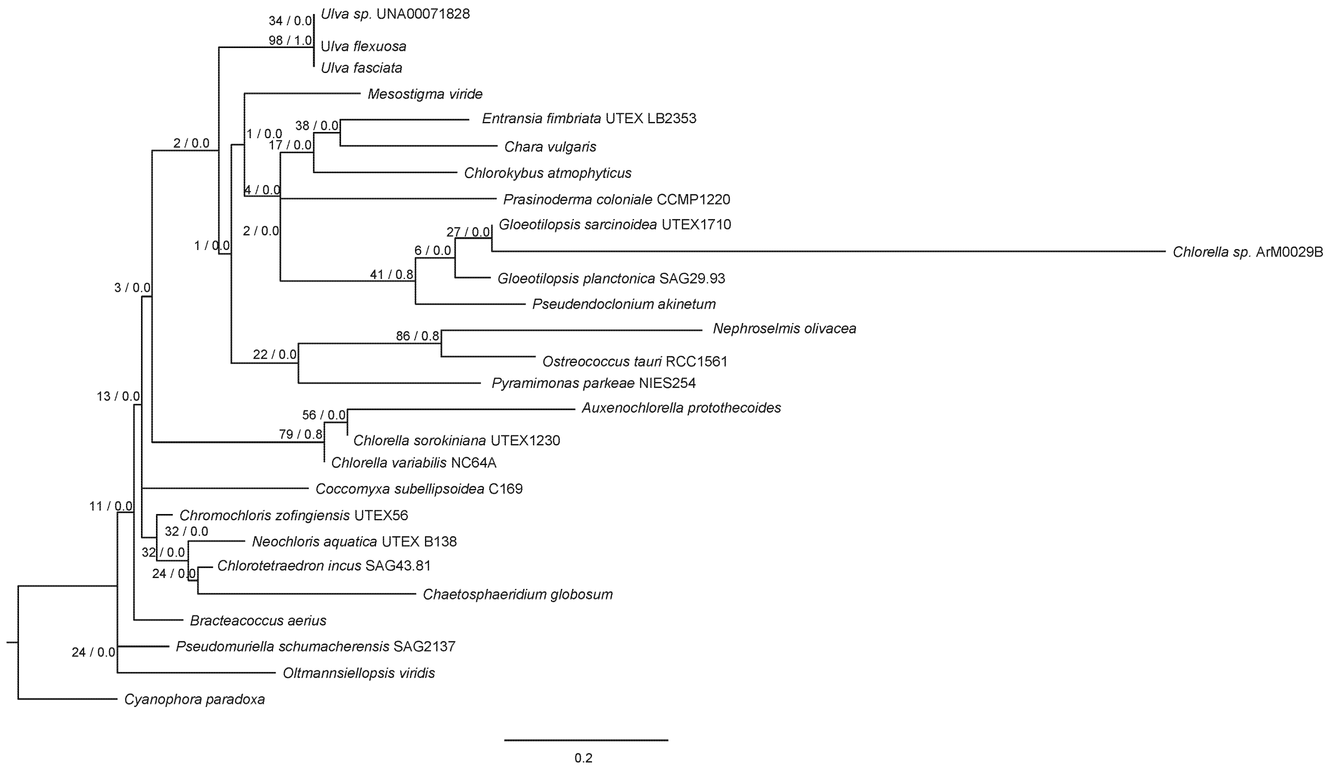

S3 Fig. BA. Maximum-Likelihood tree inferred from plastid *psb*J of algal taxa in Table 1 using a GTR+Γ+I nucleotide substitution model. Bootstrap value from ML and posterior probability from Bayesian inference are shown at the node. The scale bar represents the estimated number of nucleotide substitution per site.

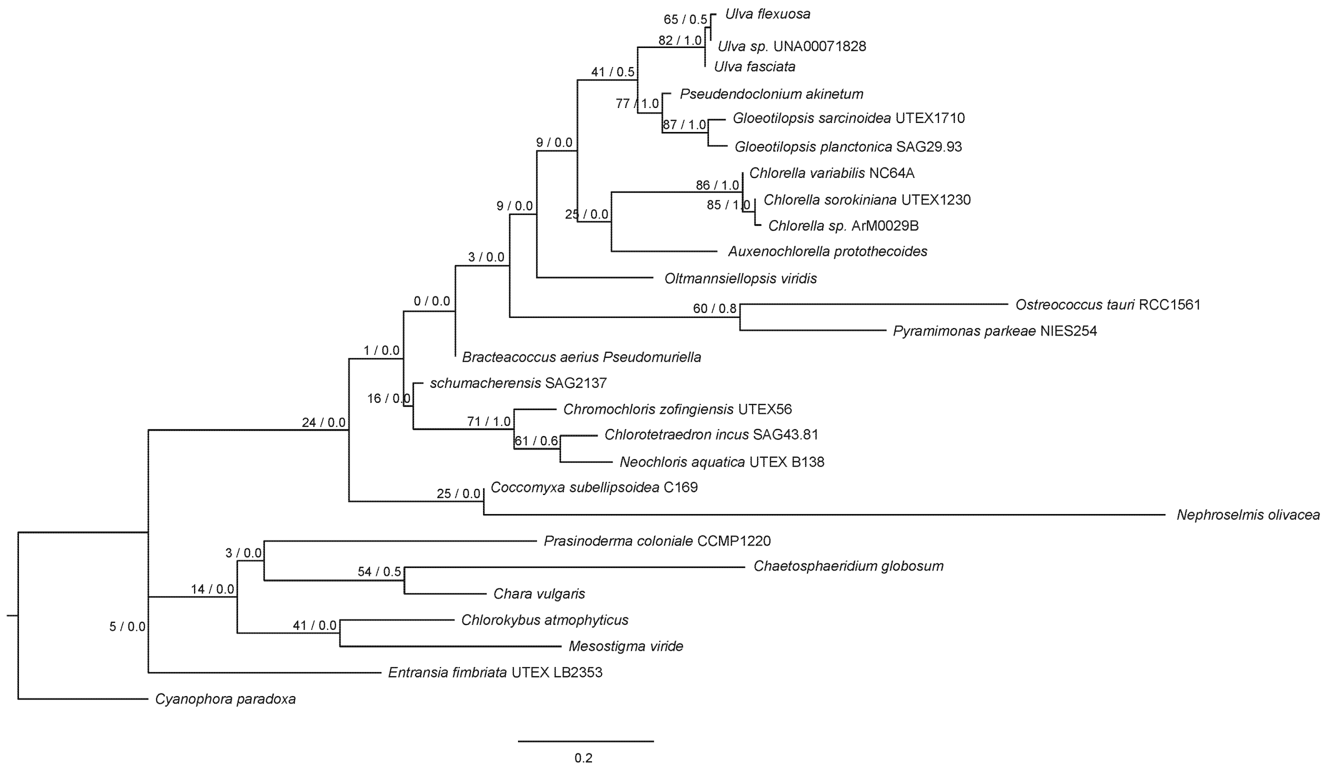

S3 Fig. BB. Maximum-Likelihood tree inferred from plastid *psb*K of algal taxa in Table 1 using a GTR+Γ+I nucleotide substitution model. Bootstrap value from ML and posterior probability from Bayesian inference are shown at the node. The scale bar represents the estimated number of nucleotide substitution per site.

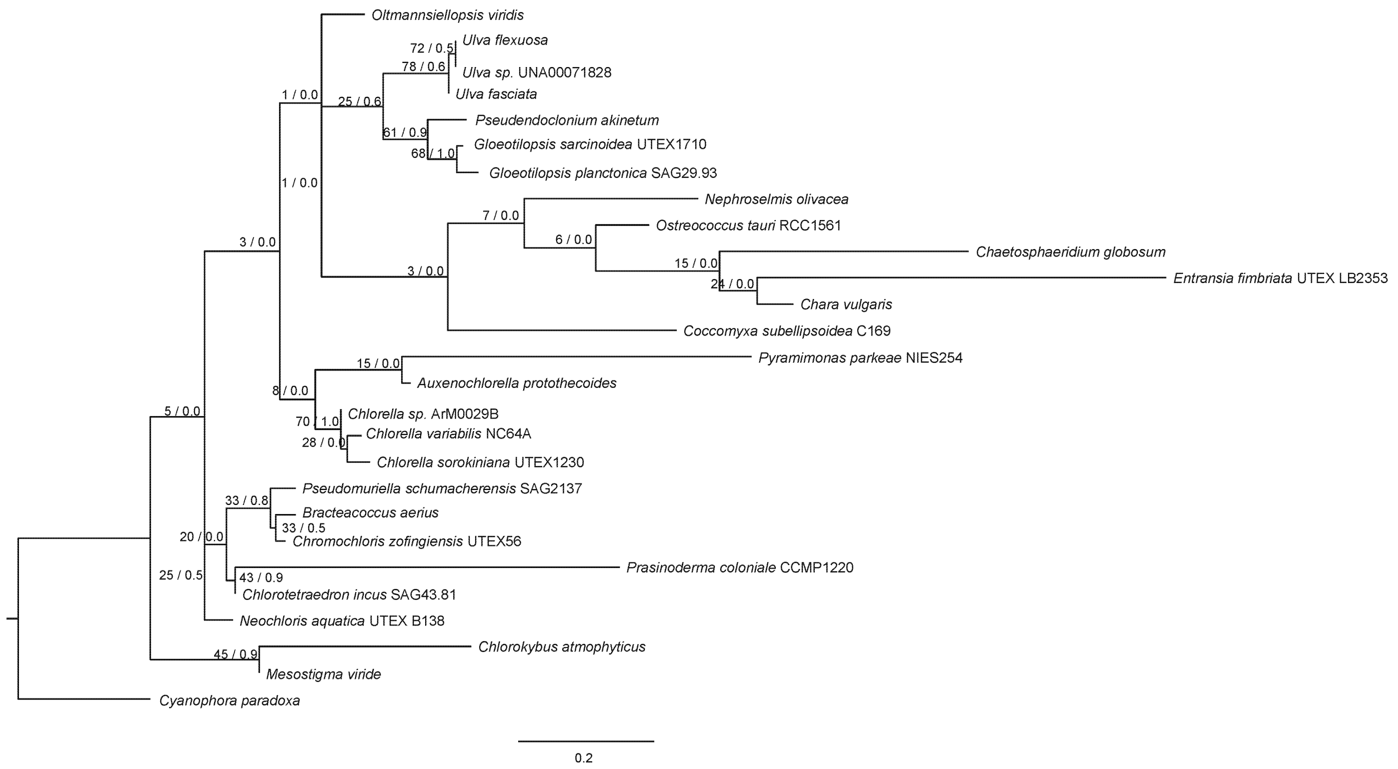

S3 Fig. BC. Maximum-Likelihood tree inferred from plastid *psb*L of algal taxa in Table 1 using a GTR+Γ+I nucleotide substitution model. Bootstrap value from ML and posterior probability from Bayesian inference are shown at the node. The scale bar represents the estimated number of nucleotide substitution per site.

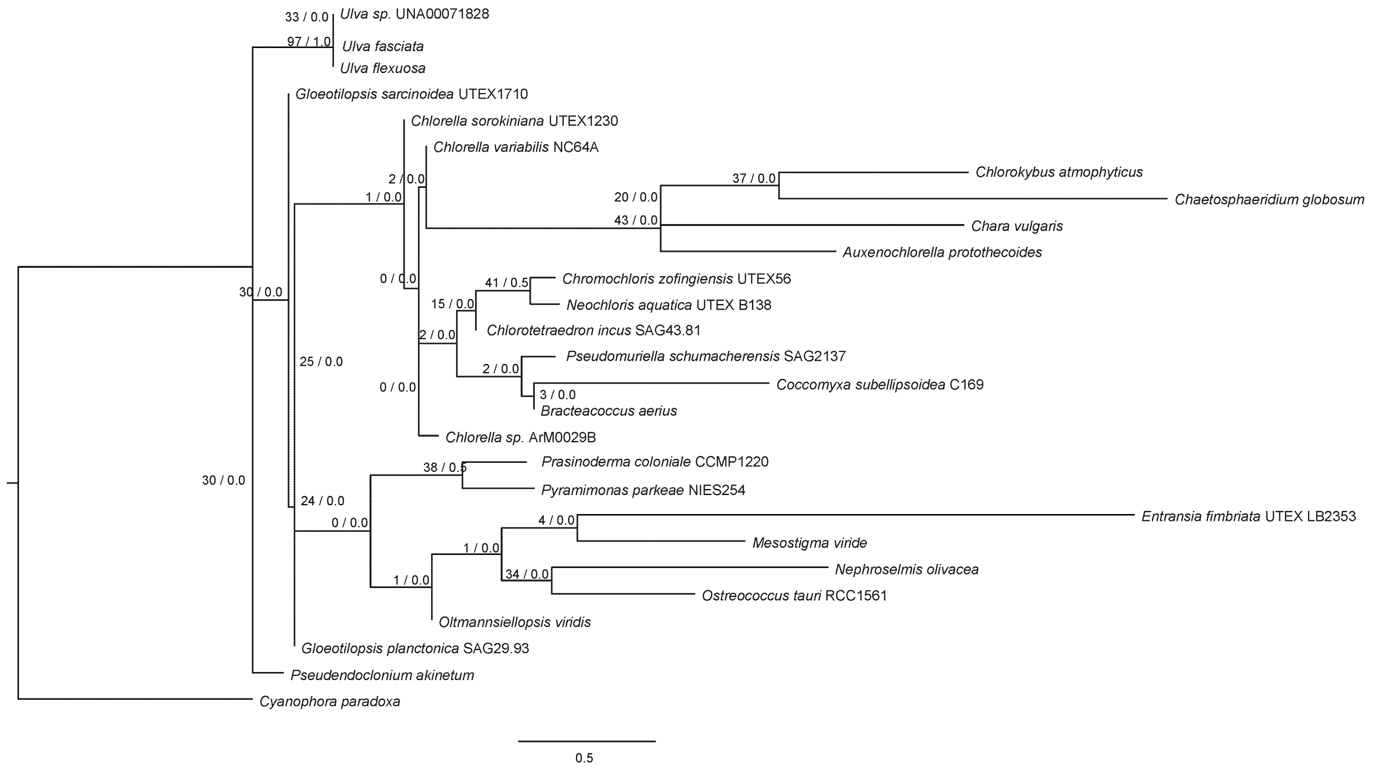

S3 Fig. BD. Maximum-Likelihood tree inferred from plastid *psb*T of algal taxa in Table 1 using a GTR+Γ+I nucleotide substitution model. Bootstrap value from ML and posterior probability from Bayesian inference are shown at the node. The scale bar represents the estimated number of nucleotide substitution per site.

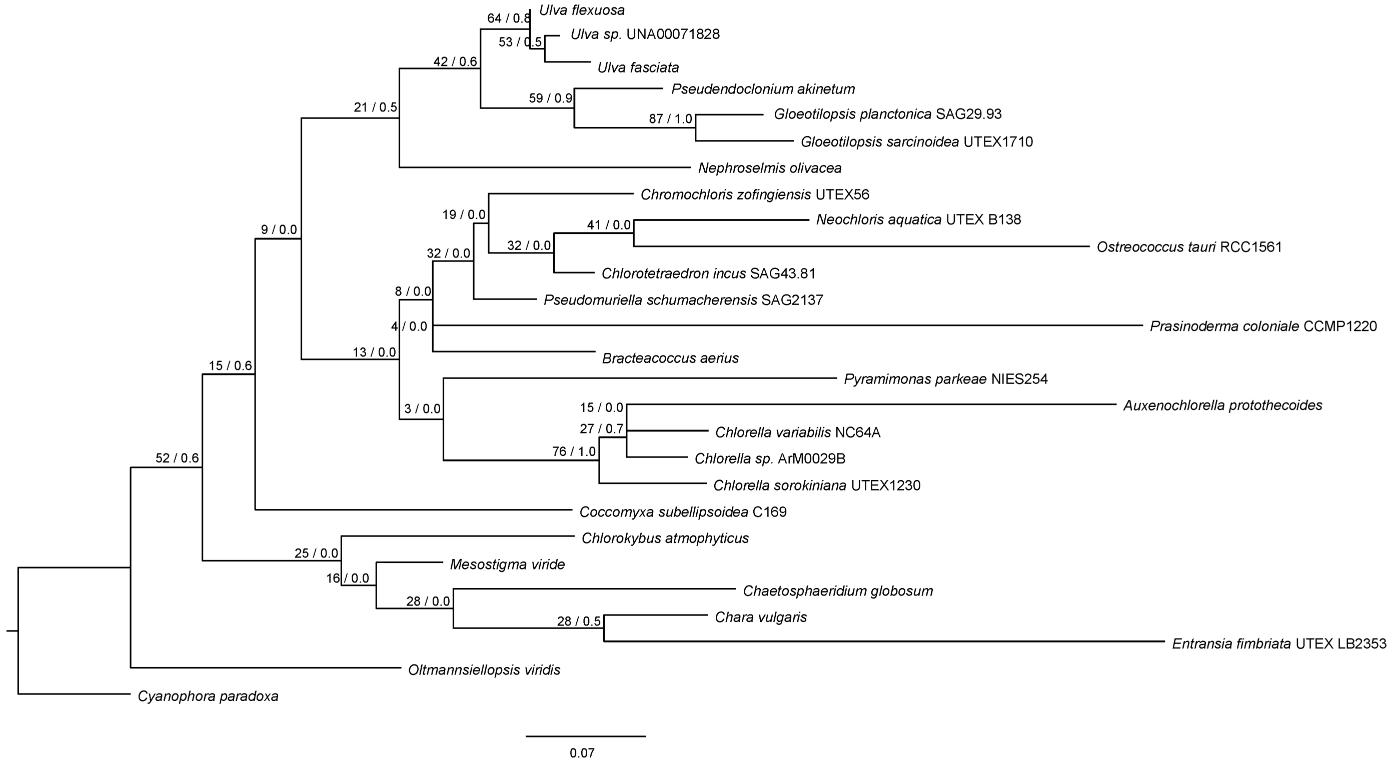

S3 Fig. BE. Maximum-Likelihood tree inferred from plastid *rpl*36 of algal taxa in Table 1 using a GTR+Γ+I nucleotide substitution model. Bootstrap value from ML and posterior probability from Bayesian inference are shown at the node. The scale bar represents the estimated number of nucleotide substitution per site.

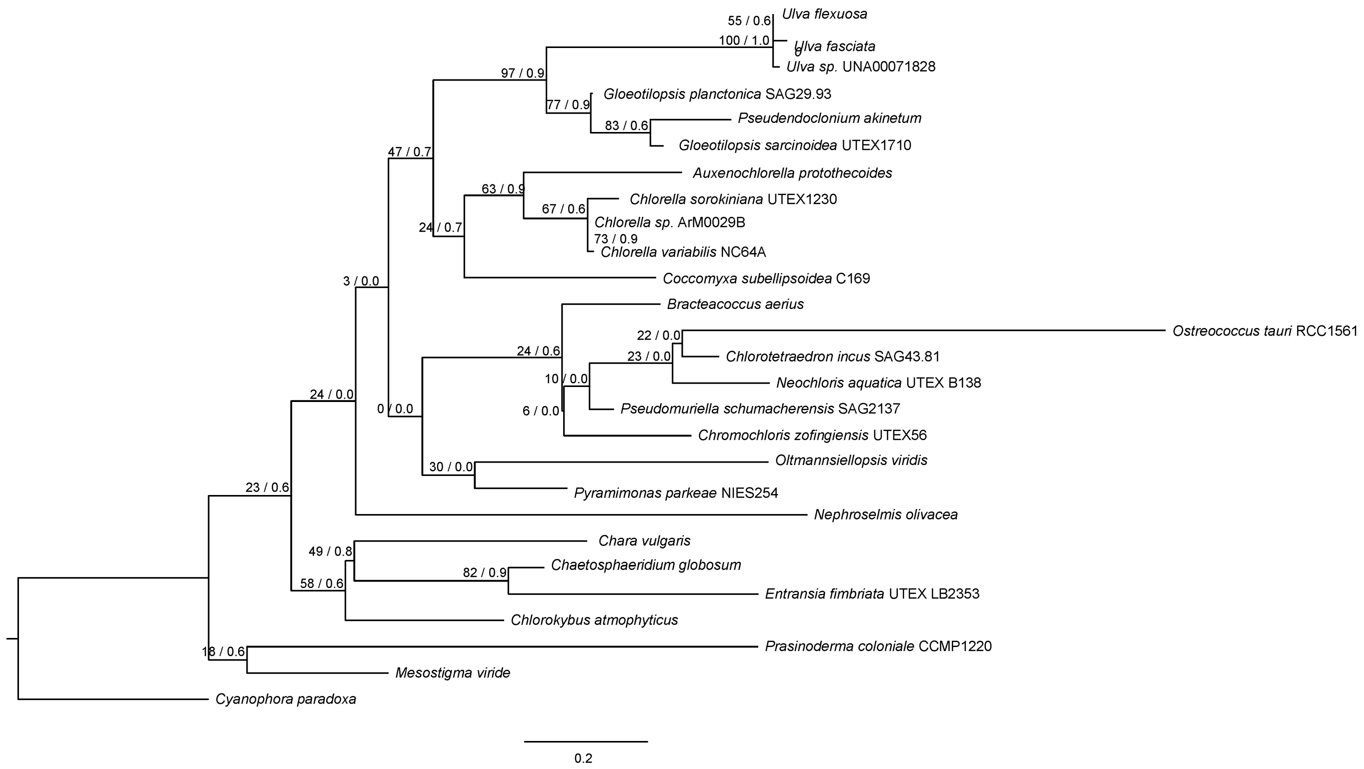

S3 Fig. BF. Maximum-Likelihood tree inferred from plastid *ycf*12 of algal taxa in Table 1 using a GTR+Γ+I nucleotide substitution model. Bootstrap value from ML and posterior probability from Bayesian inference are shown at the node. The scale bar represents the estimated number of nucleotide substitution per site.

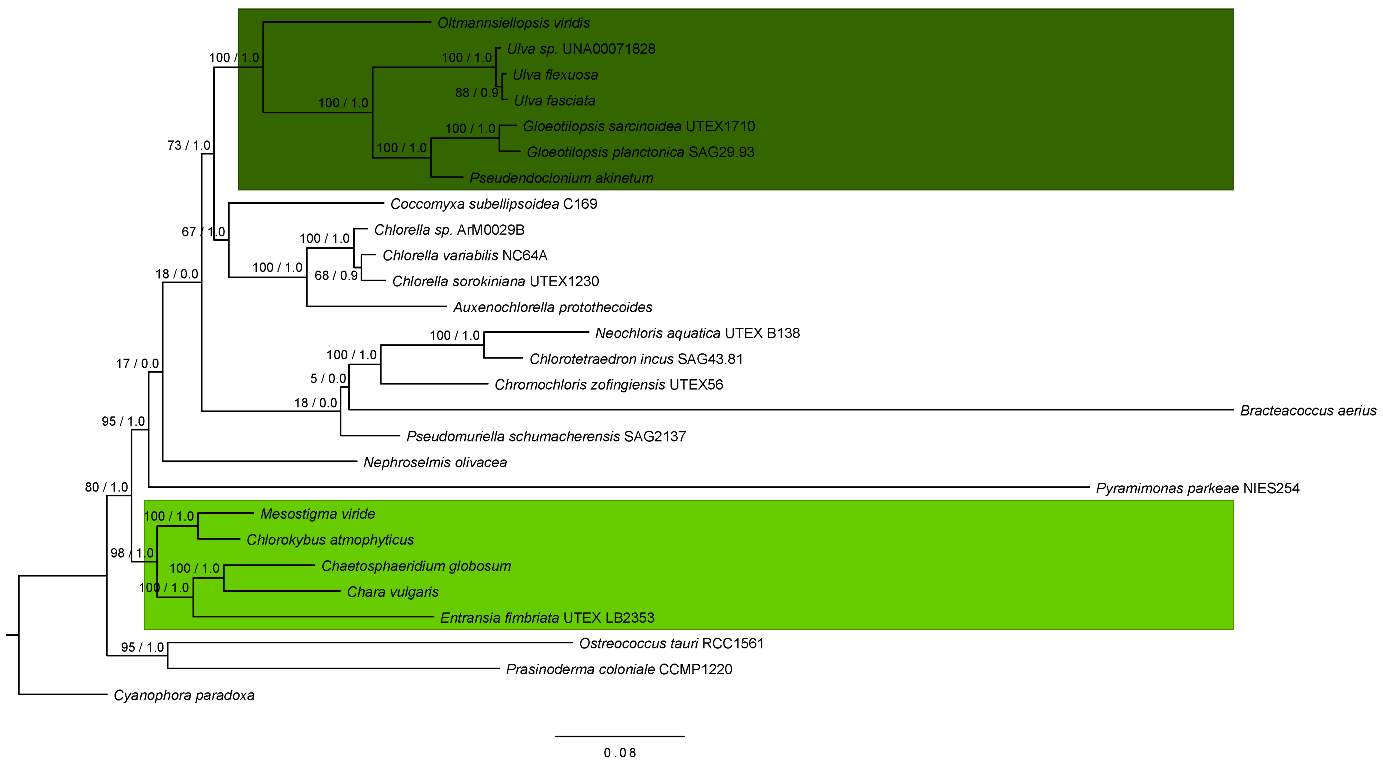

S3 Fig. BG. Maximum-Likelihood tree inferred from 2 rDNA sequences (*rrn*L and *rrs*) of algal taxa in Table 1 using a GTR+Γ+I nucleotide substitution model. Bootstrap value from ML and posterior probability from Bayesian inference are shown at the node. The scale bar represents the estimated number of nucleotide substitution per site.
