# Supplementary material for: Evolution of organellar genes of chlorophyte algae: Relevance to phylogenetic inference
Source: PLoS One. 2019 May 6;14(5):e0216608. doi: 10.1371/journal.pone.0216608 (PMC6502327; doi:10.1371/journal.pone.0216608)
Supplement: S4 File — (PDF) [file pone.0216608.s007.pdf]

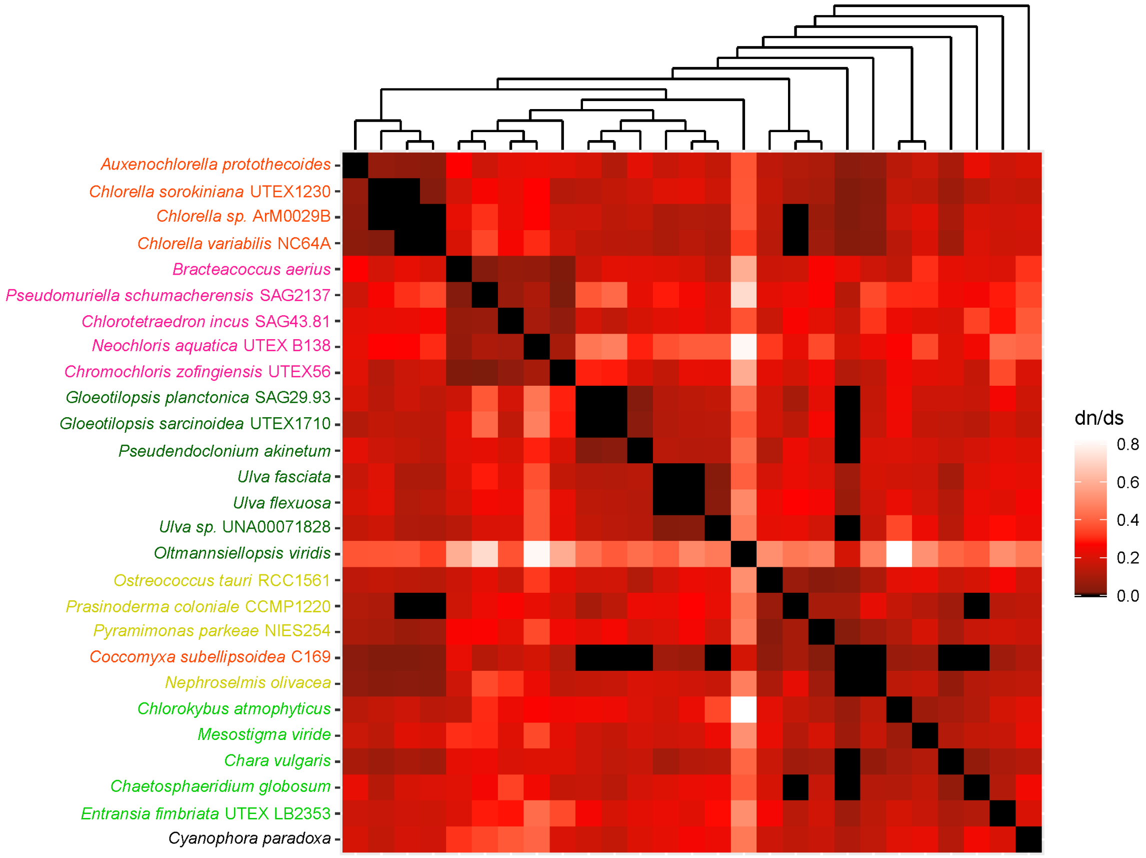

S4 Fig. A. Heatmap of pairwise dN/dS ratios calculated from alignment of mitochondrial *atp*9 of algal taxa in Table 1 by MEGA7.0 using modified Nei-Gojobori method (Jukes-Cantor). The scale bar showed the color corresponded to dn/ds ratios. The cladogram above the heatmap corresponded to its single-gene phylogenetic tree.

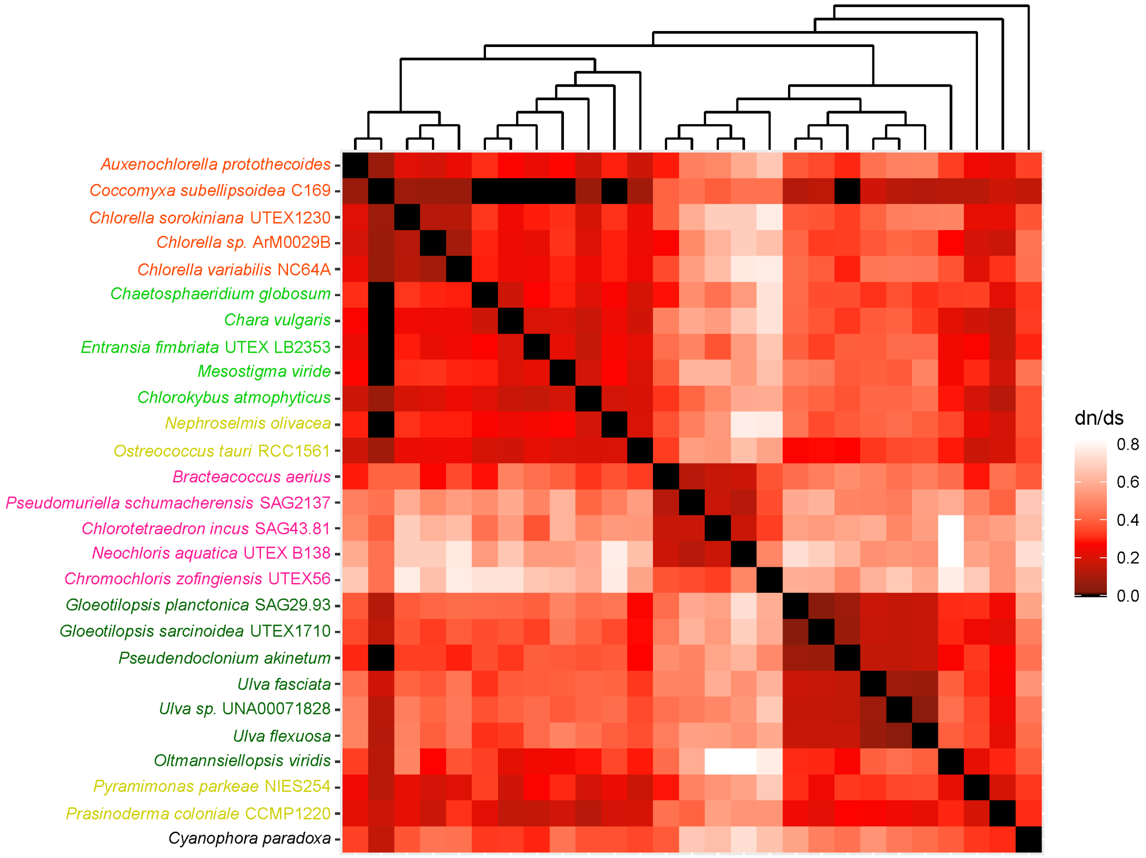

S4 Fig. B. Heatmap of pairwise dN/dS ratios calculated from alignment of mitochondrial *cox*3 of algal taxa in Table 1 by MEGA7.0 using modified Nei-Gojobori method (Jukes-Cantor). The scale bar showed the color corresponded to dn/ds ratios. The cladogram above the heatmap corresponded to its single-gene phylogenetic tree.

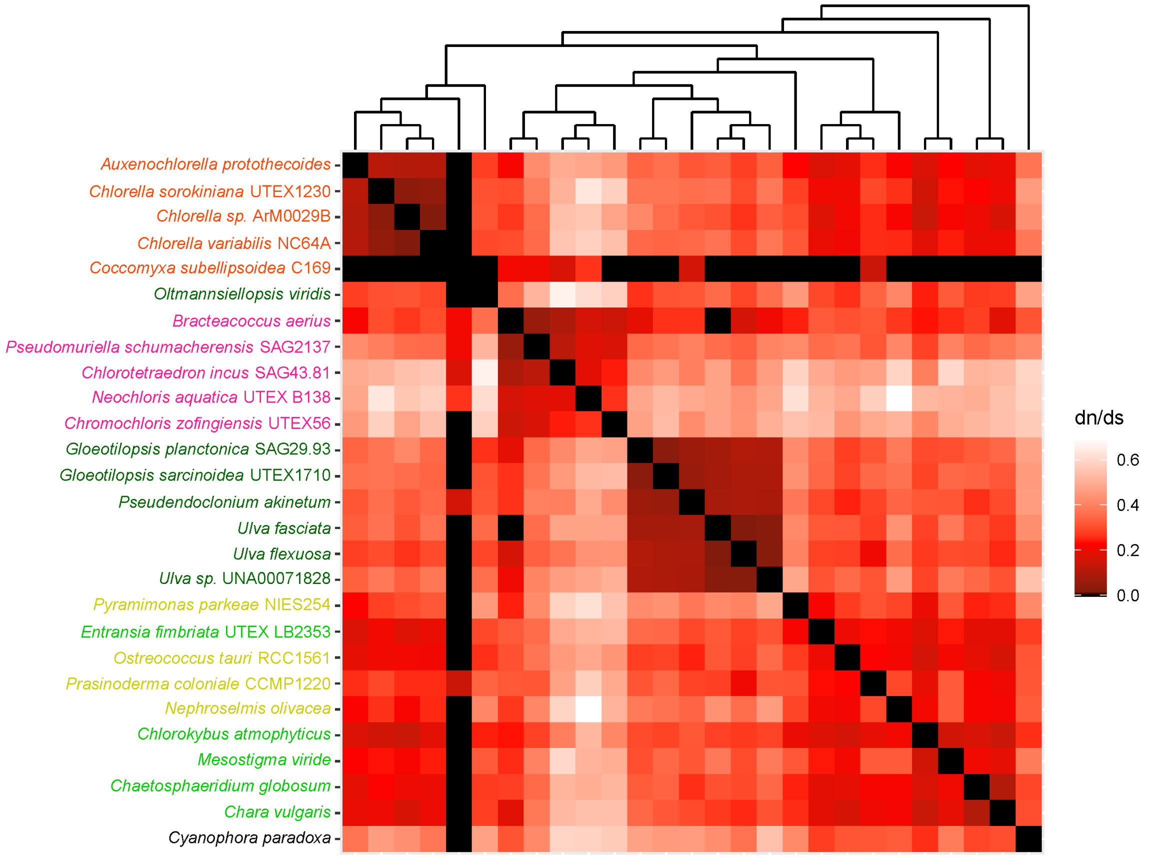

S4 Fig. C. Heatmap of pairwise dN/dS ratios calculated from alignment of mitochondrial *nad*1 of algal taxa in Table 1 by MEGA7.0 using modified Nei-Gojobori method (Jukes-Cantor). The scale bar showed the color corresponded to dn/ds ratios. The cladogram above the heatmap corresponded to its single-gene phylogenetic tree.

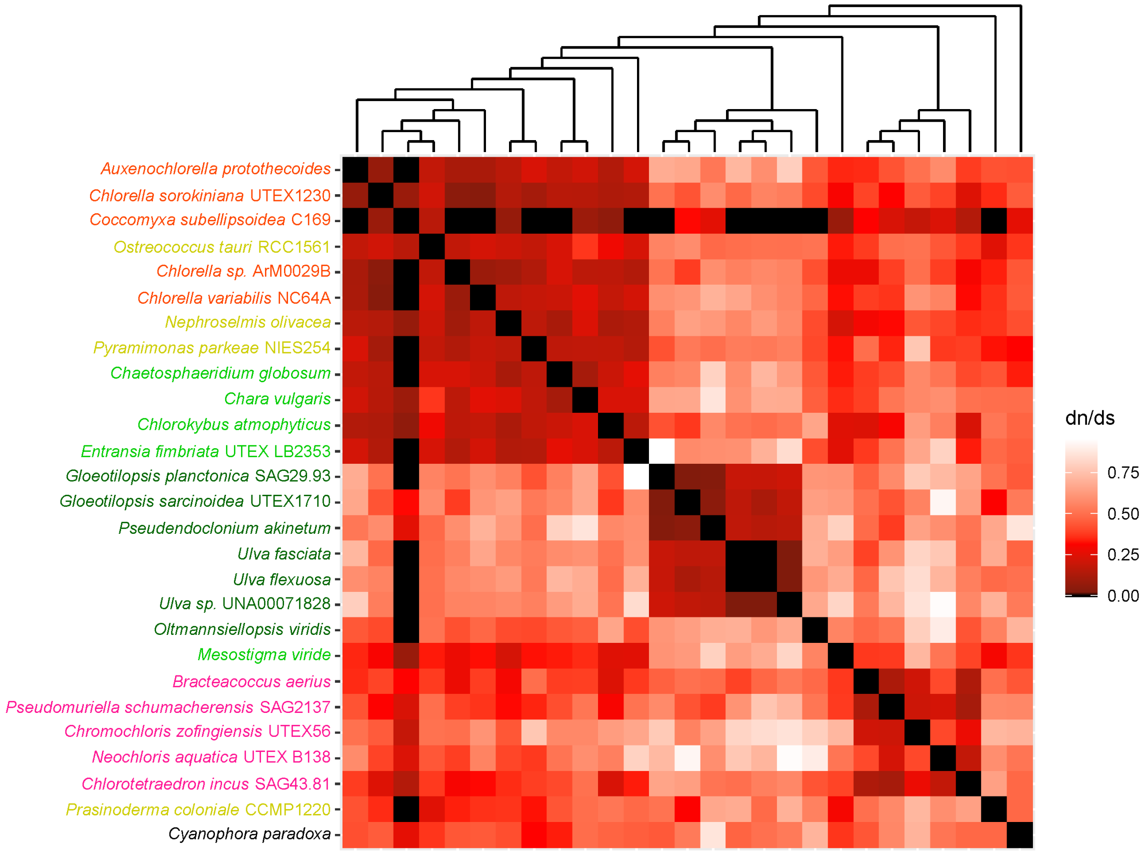

S4 Fig. D. Heatmap of pairwise dN/dS ratios calculated from alignment of mitochondrial *nad*4L of algal taxa in Table 1 by MEGA7.0 using modified Nei-Gojobori method (Jukes-Cantor). The scale bar showed the color corresponded to dn/ds ratios. The cladogram above the heatmap corresponded to its single-gene phylogenetic tree.

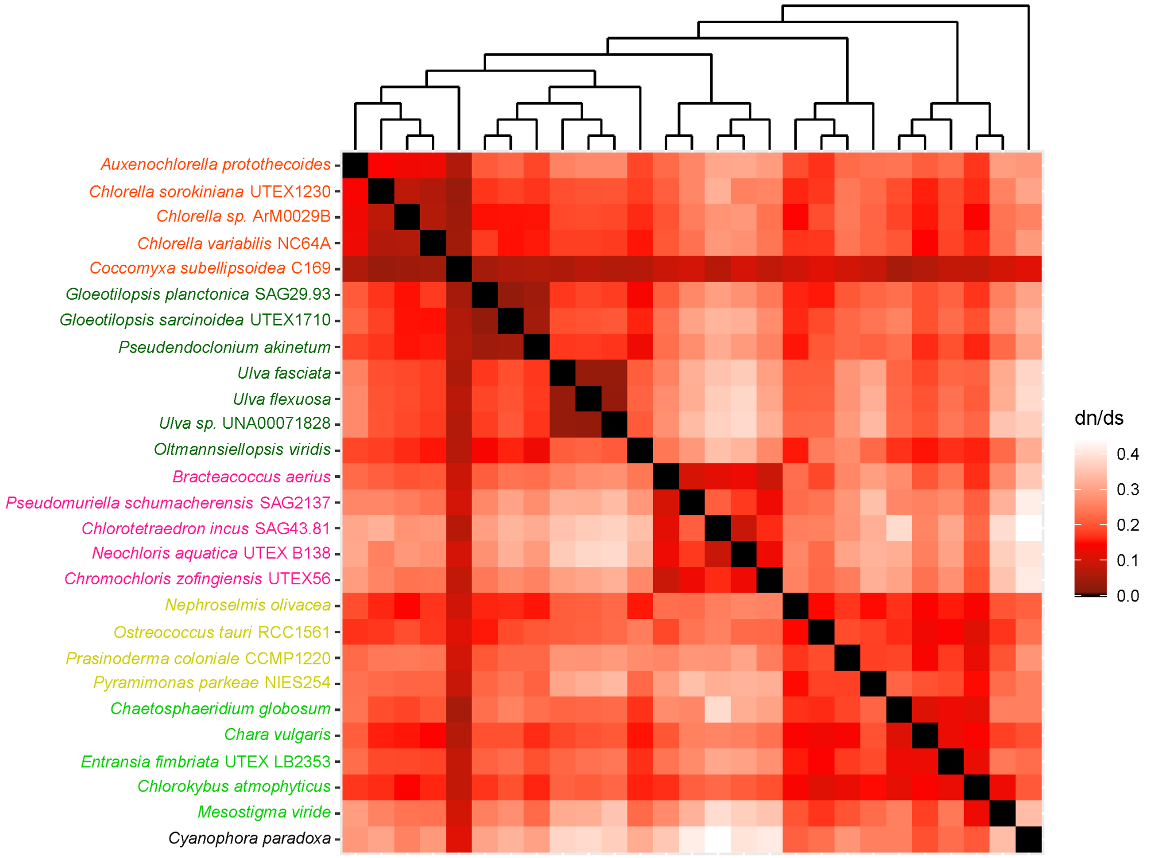

S4 Fig. E. Heatmap of pairwise dN/dS ratios calculated from alignment of plastid *atp*A of algal taxa in Table 1 by MEGA7.0 using modified Nei-Gojobori method (Jukes-Cantor). The scale bar showed the color corresponded to dn/ds ratios. The cladogram above the heatmap corresponded to its single-gene phylogenetic tree.

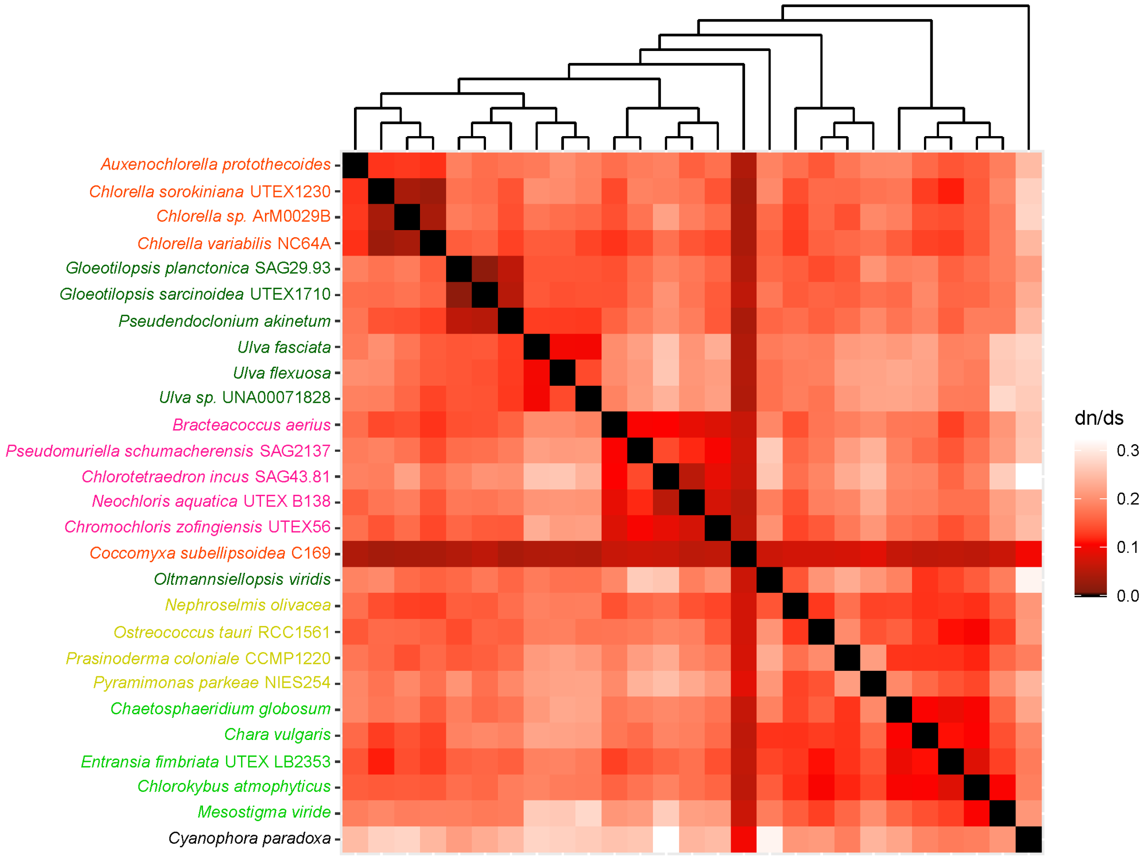

S4 Fig. F. Heatmap of pairwise dN/dS ratios calculated from alignment of plastid *atp*B of algal taxa in Table 1 by MEGA7.0 using modified Nei-Gojobori method (Jukes-Cantor). The scale bar showed the color corresponded to dn/ds ratios. The cladogram above the heatmap corresponded to its single-gene phylogenetic tree.

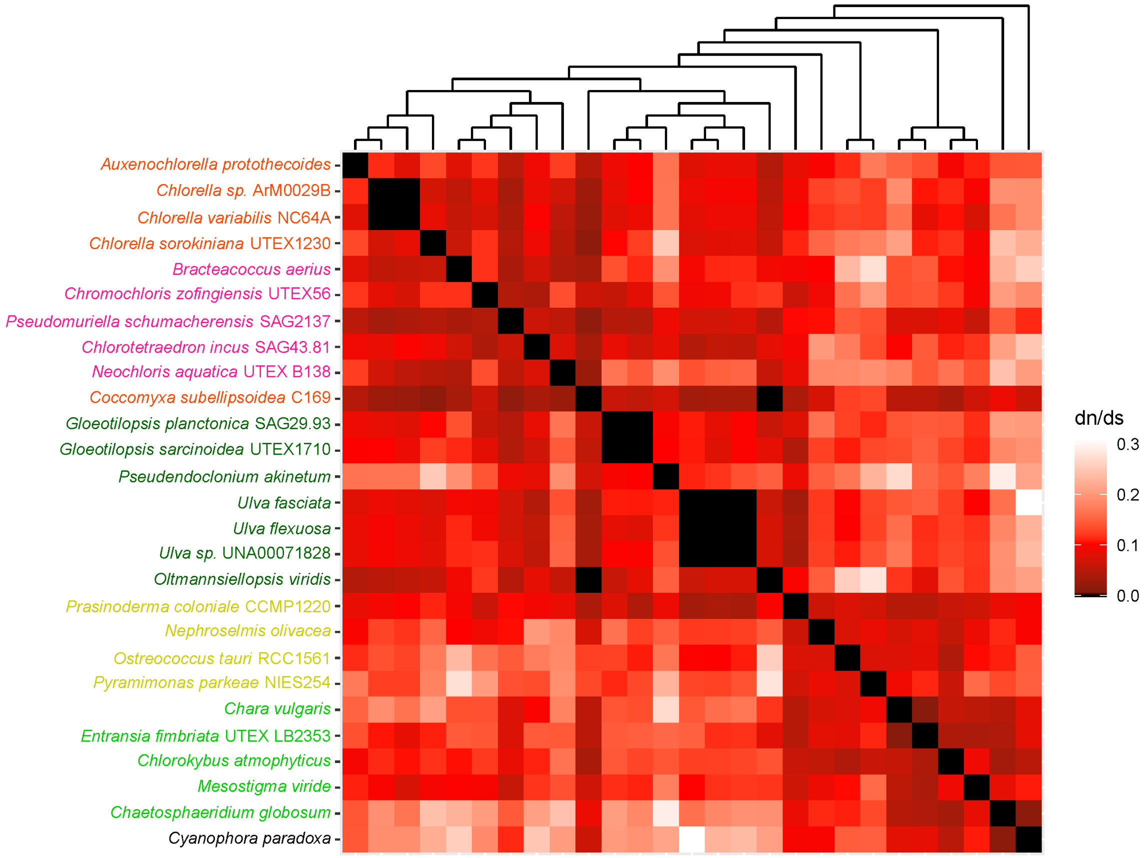

S4 Fig. G. Heatmap of pairwise dN/dS ratios calculated from alignment of plastid *atp*H of algal taxa in Table 1 by MEGA7.0 using modified Nei-Gojobori method (Jukes-Cantor). The scale bar showed the color corresponded to dn/ds ratios. The cladogram above the heatmap corresponded to its single-gene phylogenetic tree.

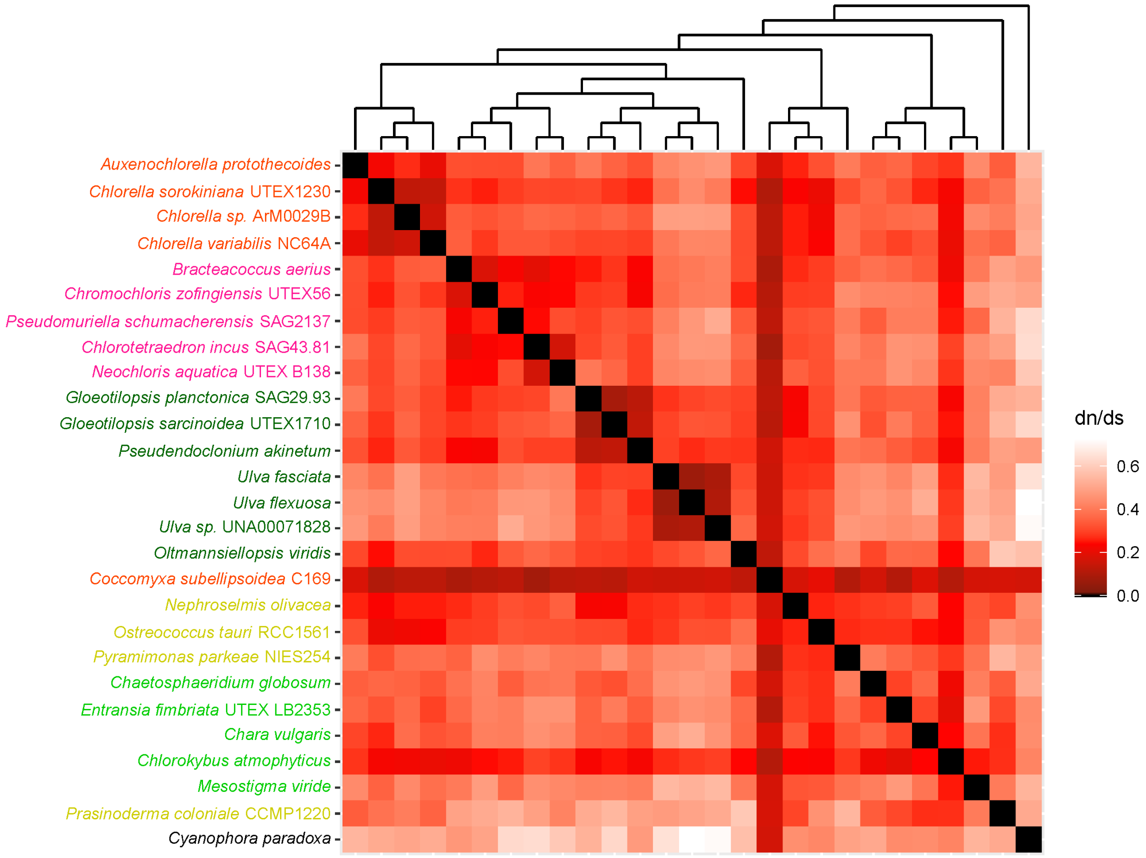

S4 Fig. H. Heatmap of pairwise dN/dS ratios calculated from alignment of plastid *pet*A of algal taxa in Table 1 by MEGA7.0 using modified Nei-Gojobori method (Jukes-Cantor). The scale bar showed the color corresponded to dn/ds ratios. The cladogram above the heatmap corresponded to its single-gene phylogenetic tree.

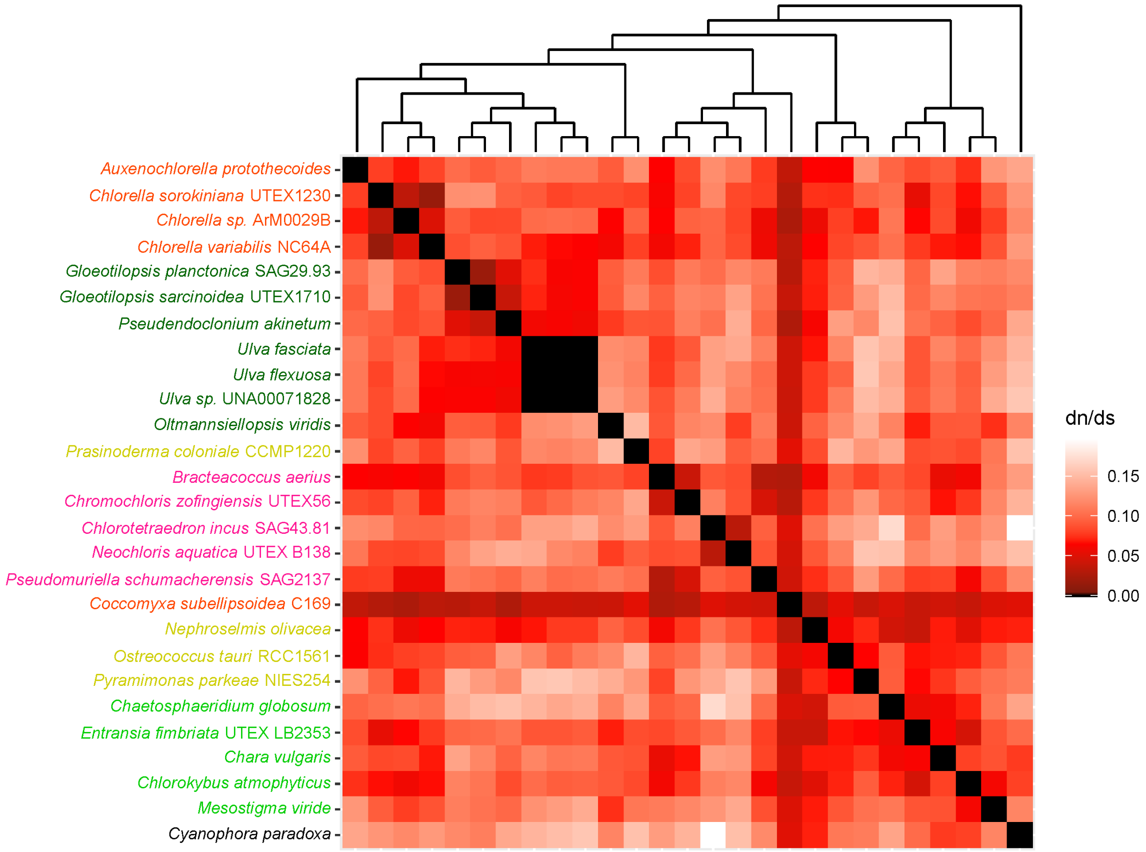

S4 Fig. I. Heatmap of pairwise dN/dS ratios calculated from alignment of plastid *pet*B of algal taxa in Table 1 by MEGA7.0 using modified Nei-Gojobori method (Jukes-Cantor). The scale bar showed the color corresponded to dn/ds ratios. The cladogram above the heatmap corresponded to its single-gene phylogenetic tree.

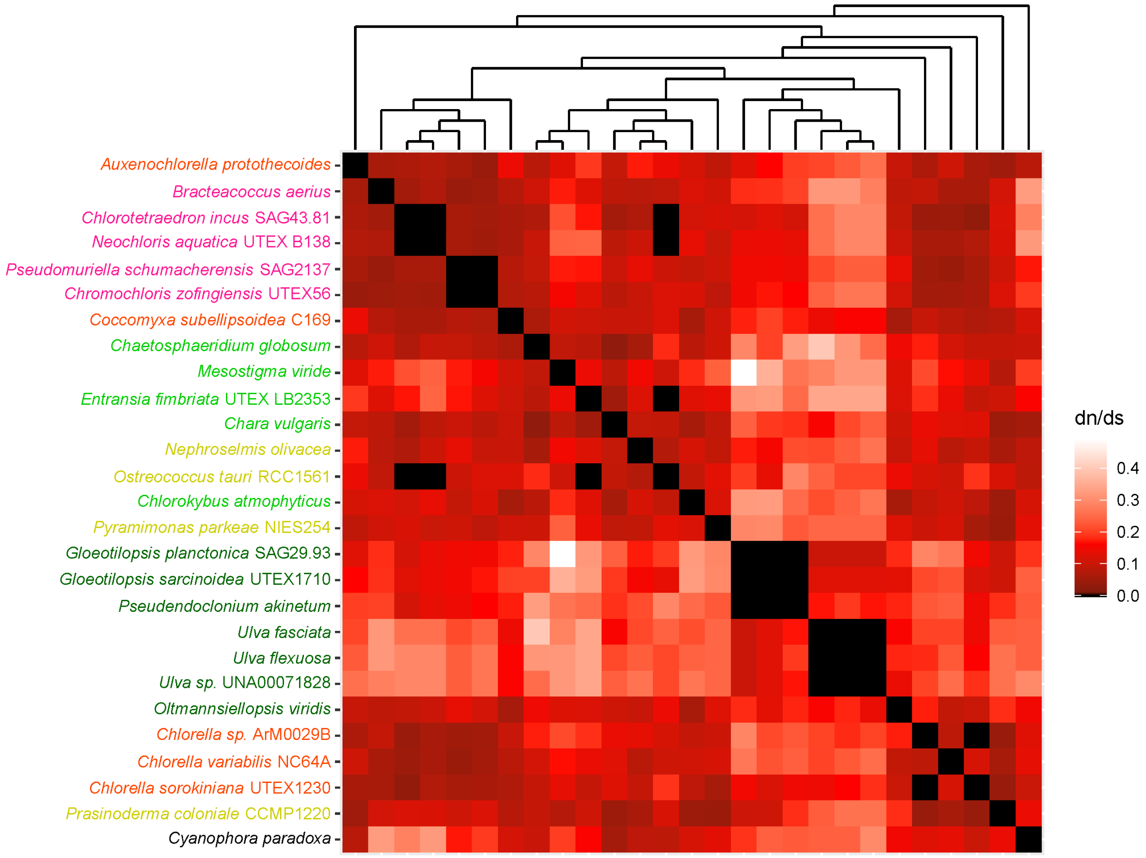

S4 Fig. J. Heatmap of pairwise dN/dS ratios calculated from alignment of plastid *pet*G of algal taxa in Table 1 by MEGA7.0 using modified Nei-Gojobori method (Jukes-Cantor). The scale bar showed the color corresponded to dn/ds ratios. The cladogram above the heatmap corresponded to its single-gene phylogenetic tree.

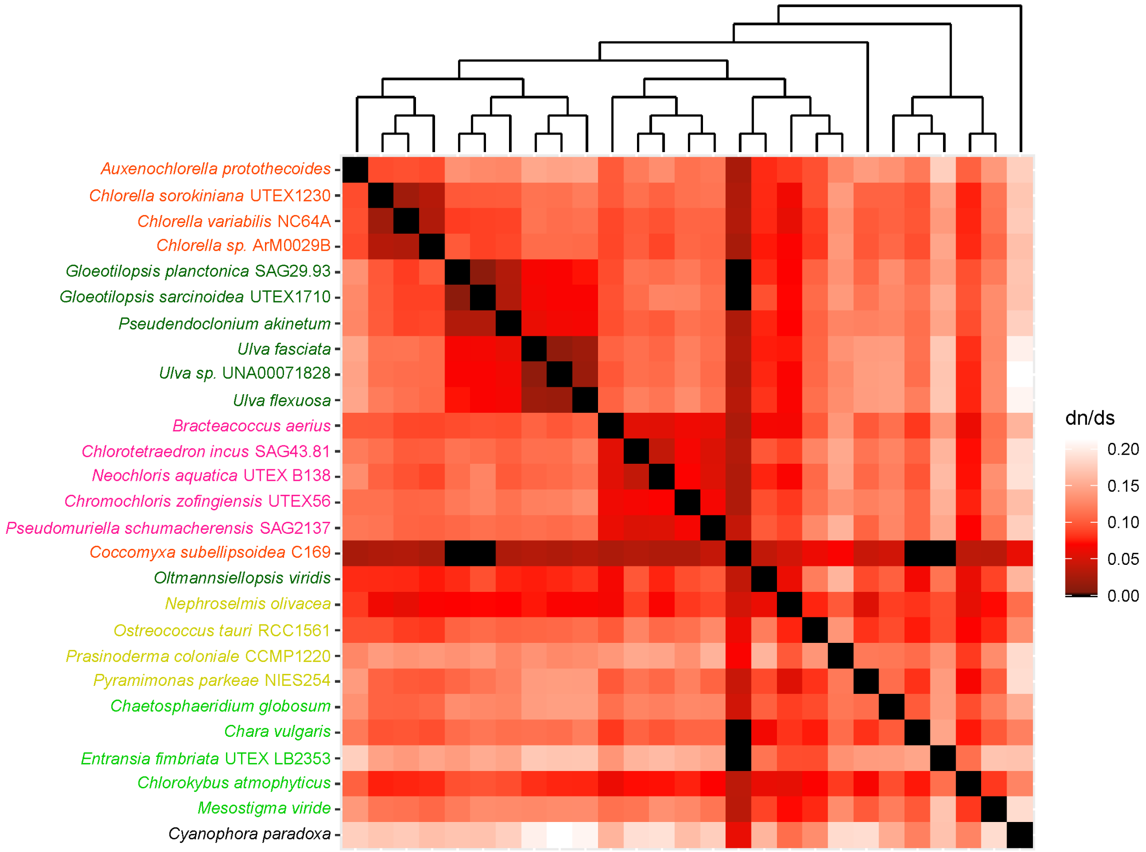

S4 Fig. K. Heatmap of pairwise dN/dS ratios calculated from alignment of plastid *psa*A of algal taxa in Table 1 by MEGA7.0 using modified Nei-Gojobori method (Jukes-Cantor). The scale bar showed the color corresponded to dn/ds ratios. The cladogram above the heatmap corresponded to its single-gene phylogenetic tree.

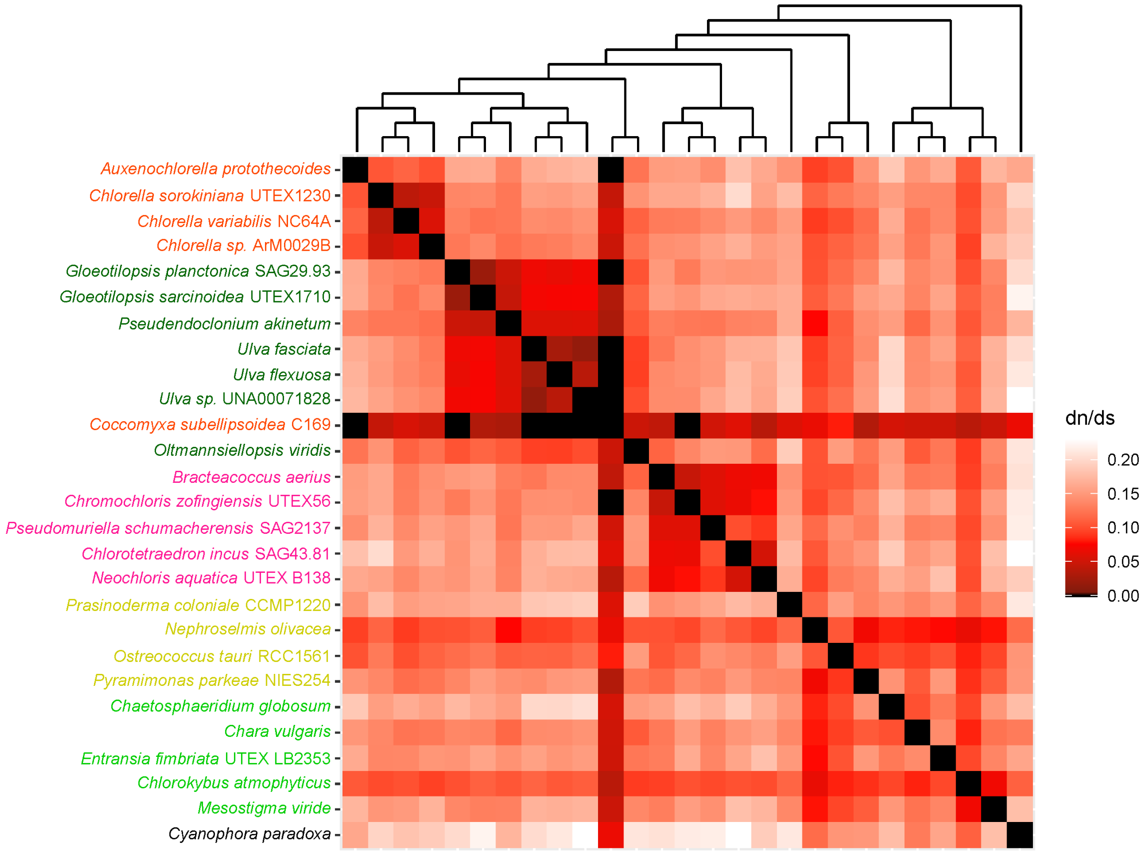

S4 Fig. L. Heatmap of pairwise dN/dS ratios calculated from alignment of plastid *psa*B of algal taxa in Table 1 by MEGA7.0 using modified Nei-Gojobori method (Jukes-Cantor). The scale bar showed the color corresponded to dn/ds ratios. The cladogram above the heatmap corresponded to its single-gene phylogenetic tree.

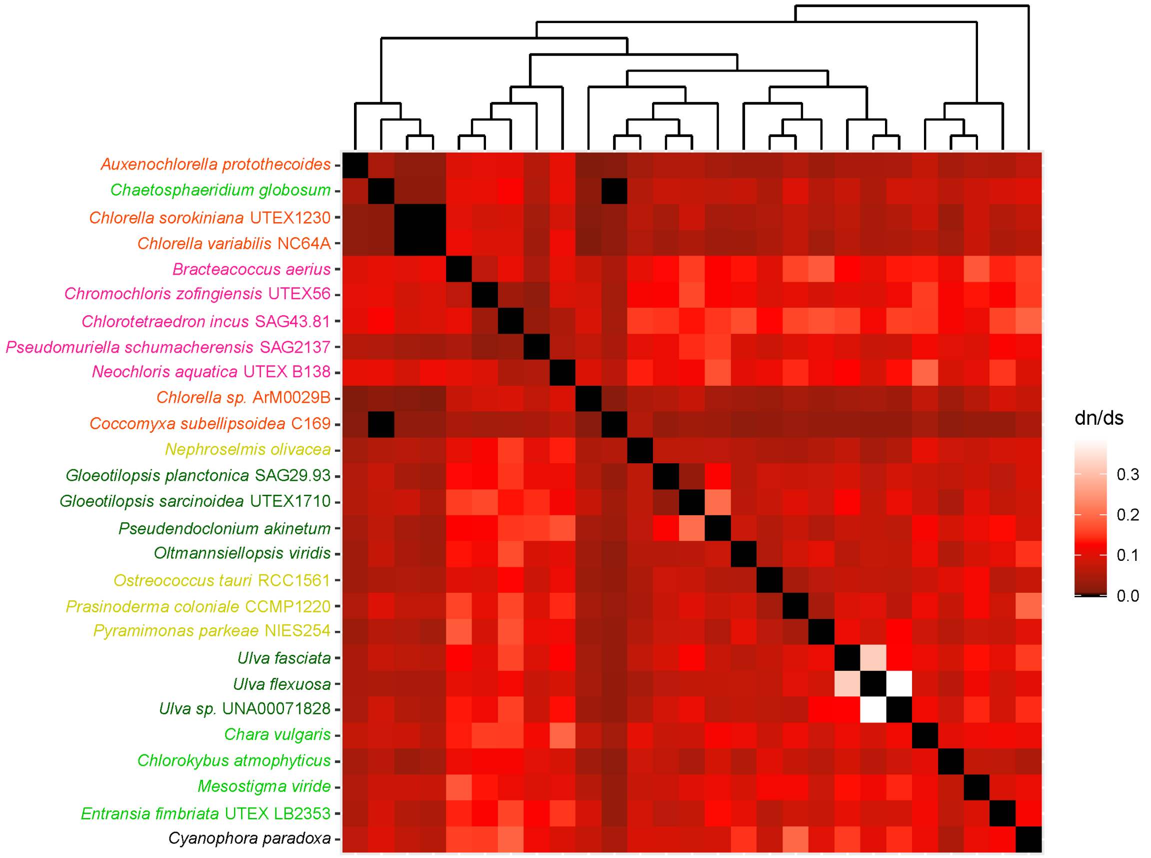

S4 Fig. M. Heatmap of pairwise dN/dS ratios calculated from alignment of plastid *psa*C of algal taxa in Table 1 by MEGA7.0 using modified Nei-Gojobori method (Jukes-Cantor). The scale bar showed the color corresponded to dn/ds ratios. The cladogram above the heatmap corresponded to its single-gene phylogenetic tree.

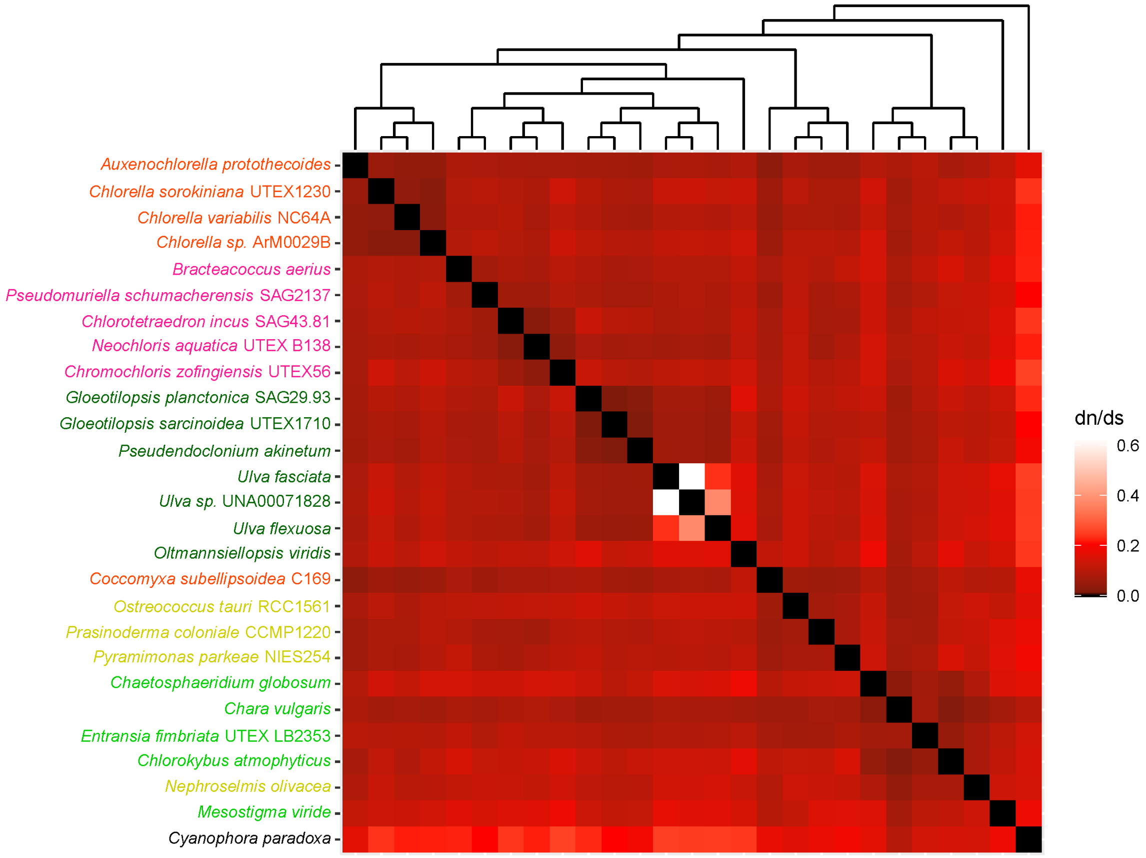

S4 Fig. N. Heatmap of pairwise dN/dS ratios calculated from alignment of plastid *psb*A of algal taxa in Table 1 by MEGA7.0 using modified Nei-Gojobori method (Jukes-Cantor). The scale bar showed the color corresponded to dn/ds ratios. The cladogram above the heatmap corresponded to its single-gene phylogenetic tree.

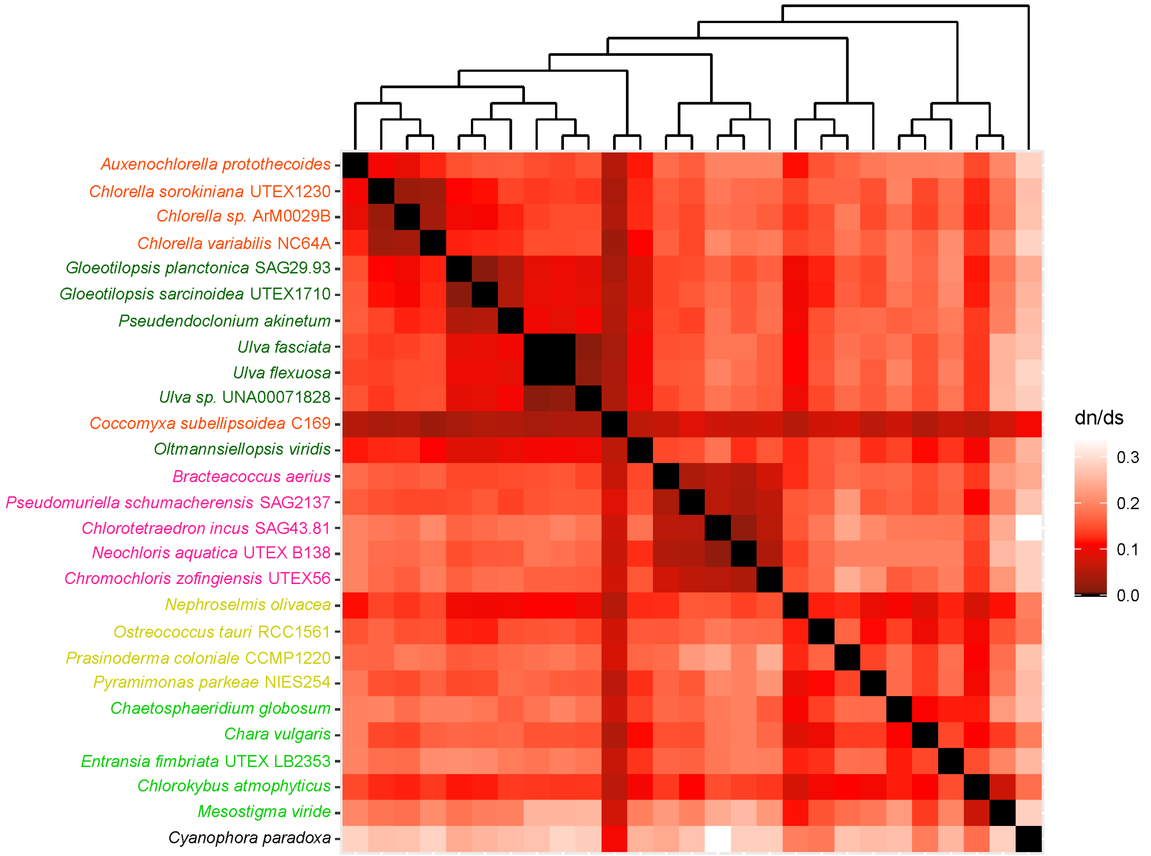

S4 Fig. O. Heatmap of pairwise dN/dS ratios calculated from alignment of plastid *psb*B of algal taxa in Table 1 by MEGA7.0 using modified Nei-Gojobori method (Jukes-Cantor). The scale bar showed the color corresponded to dn/ds ratios. The cladogram above the heatmap corresponded to its single-gene phylogenetic tree.

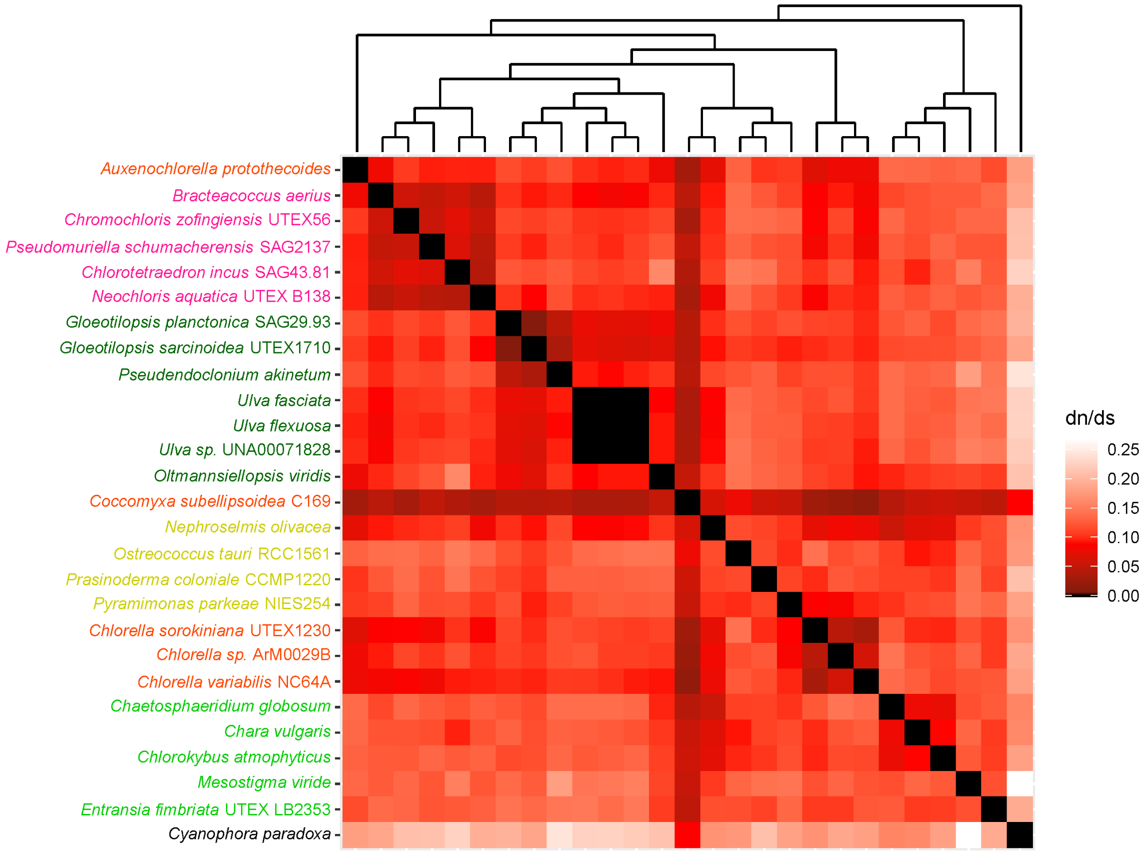

S4 Fig. P. Heatmap of pairwise dN/dS ratios calculated from alignment of plastid *psb*C of algal taxa in Table 1 by MEGA7.0 using modified Nei-Gojobori method (Jukes-Cantor). The scale bar showed the color corresponded to dn/ds ratios. The cladogram above the heatmap corresponded to its single-gene phylogenetic tree.

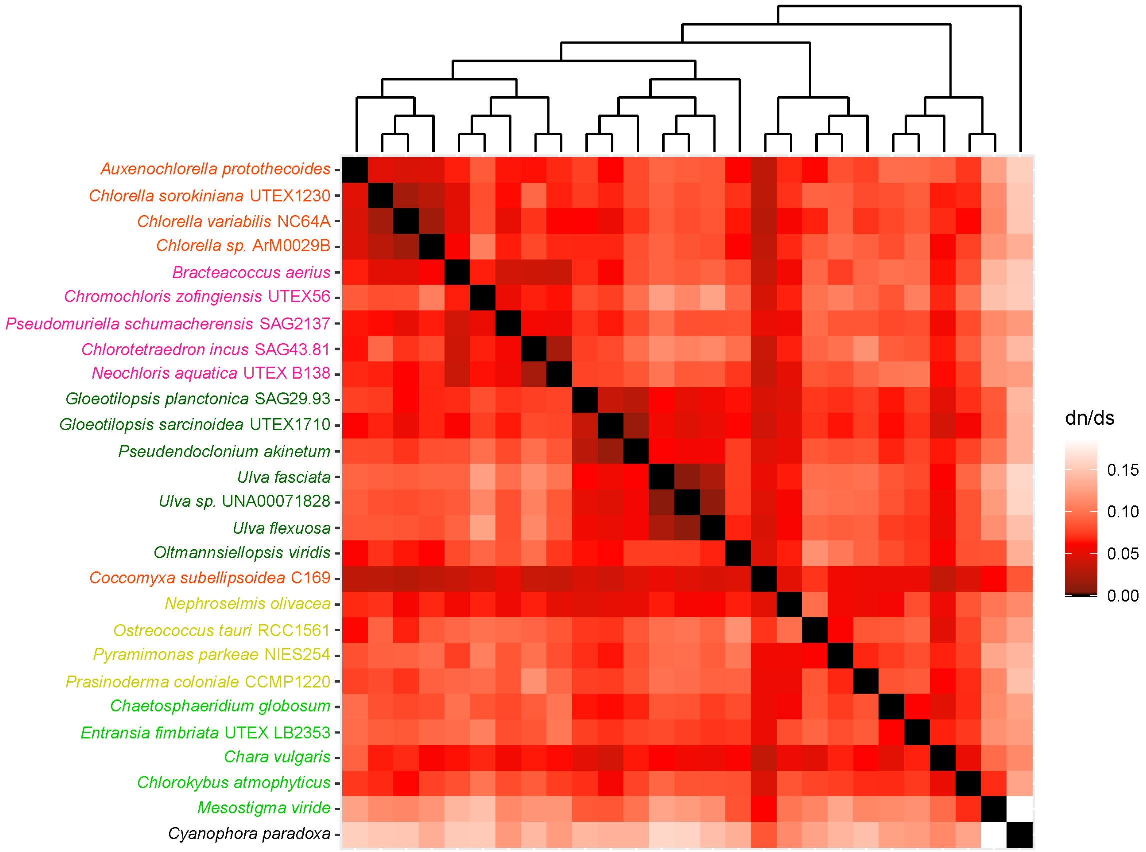

S4 Fig. Q. Heatmap of pairwise dN/dS ratios calculated from alignment of plastid *psb*D of algal taxa in Table 1 by MEGA7.0 using modified Nei-Gojobori method (Jukes-Cantor). The scale bar showed the color corresponded to dn/ds ratios. The cladogram above the heatmap corresponded to its single-gene phylogenetic tree.

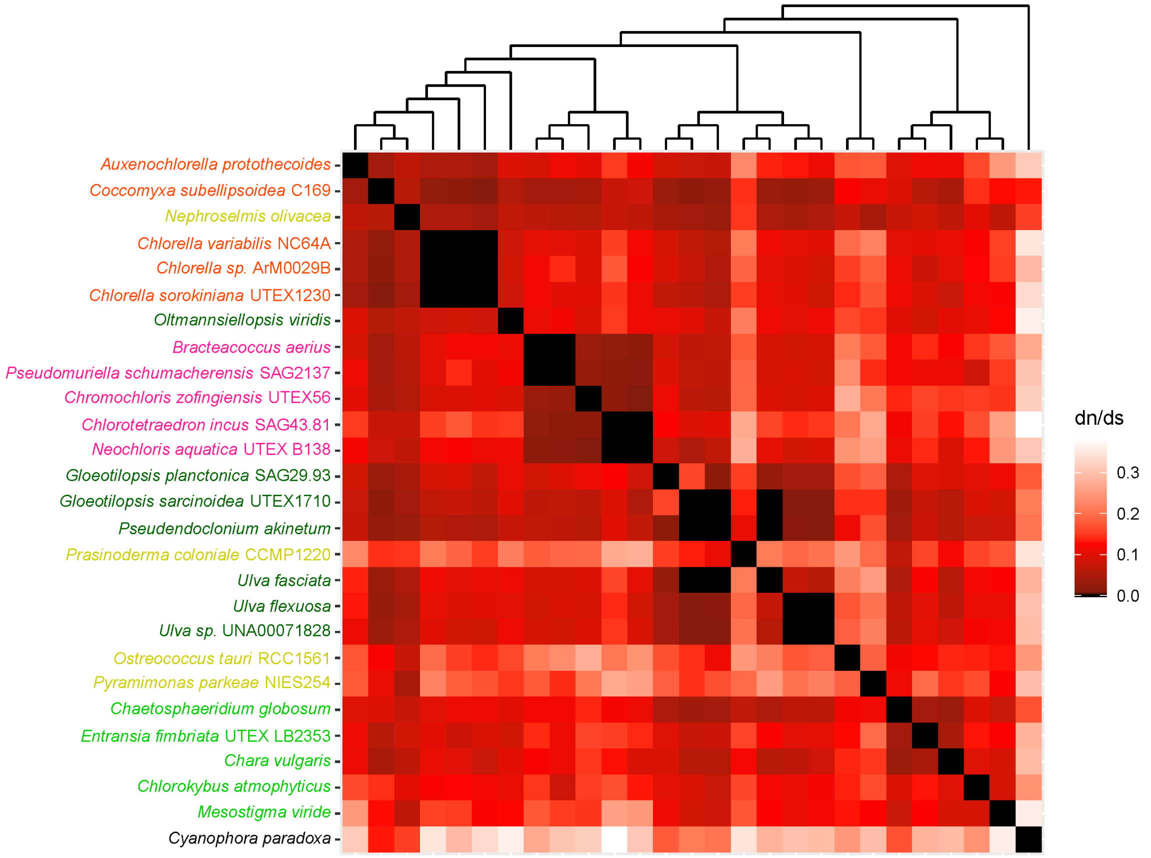

S4 Fig. R. Heatmap of pairwise dN/dS ratios calculated from alignment of plastid *psb*E of algal taxa in Table 1 by MEGA7.0 using modified Nei-Gojobori method (Jukes-Cantor). The scale bar showed the color corresponded to dn/ds ratios. The cladogram above the heatmap corresponded to its single-gene phylogenetic tree.

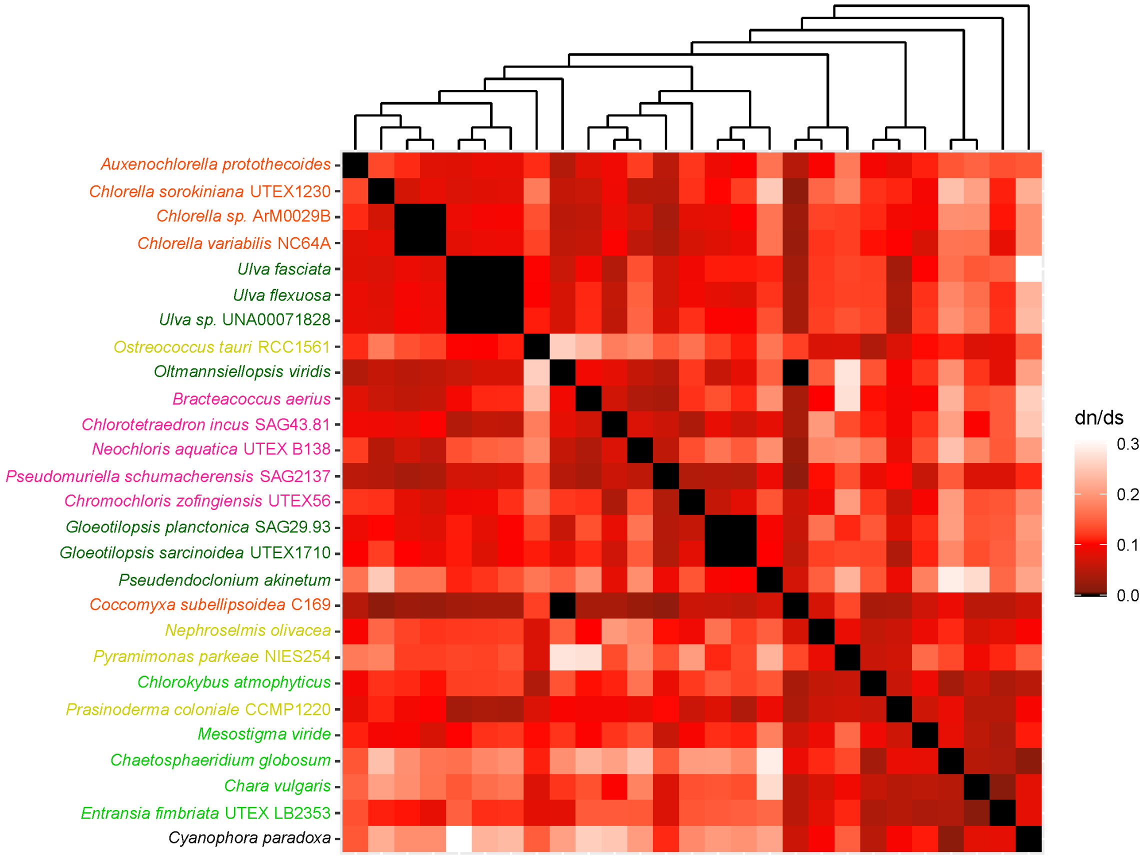

S4 Fig. Heatmap of pairwise dN/dS ratios calculated from alignment of plastid *psb*H of algal taxa in Table 1 by MEGA7.0 using modified Nei-Gojobori method (Jukes-Cantor). The scale bar showed the color corresponded to dn/ds ratios. The cladogram above the heatmap corresponded to its single-gene phylogenetic tree.

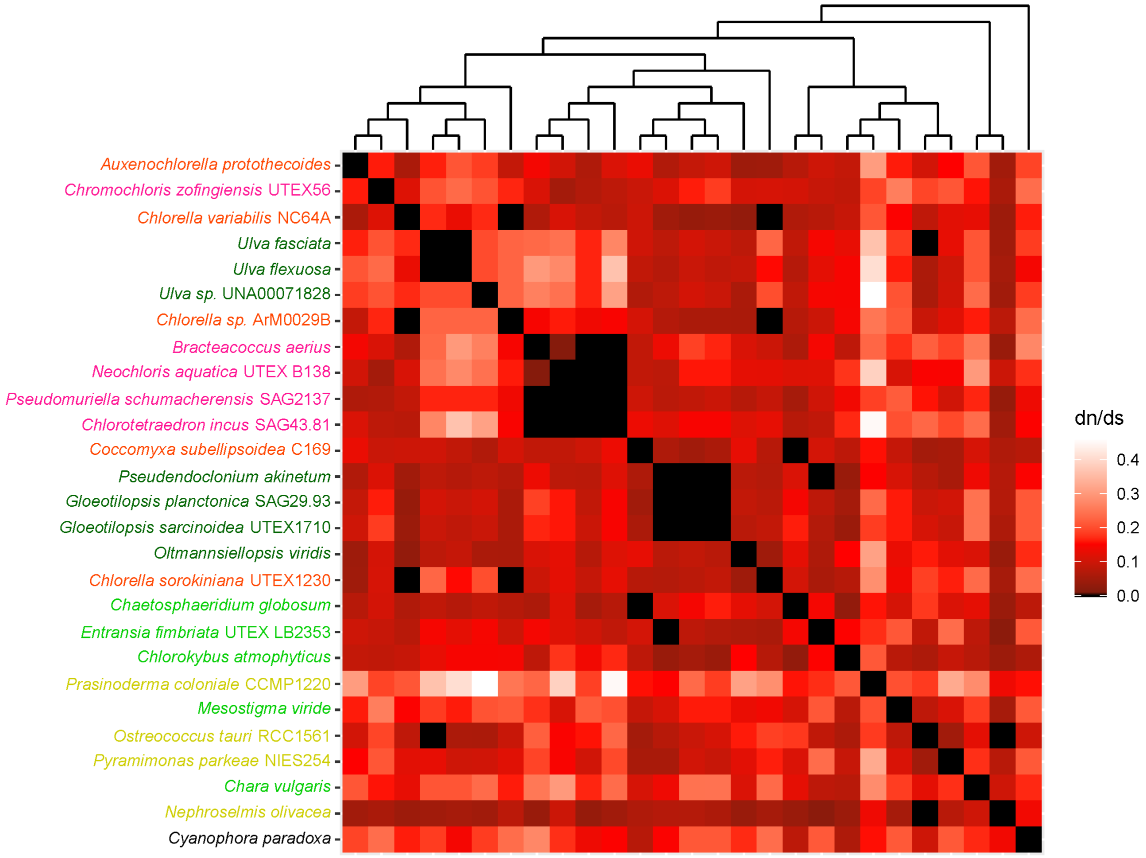

S4 Fig. S. Heatmap of pairwise dN/dS ratios calculated from alignment of plastid *psb*I of algal taxa in Table 1 by MEGA7.0 using modified Nei-Gojobori method (Jukes-Cantor). The scale bar showed the color corresponded to dn/ds ratios. The cladogram above the heatmap corresponded to its single-gene phylogenetic tree.

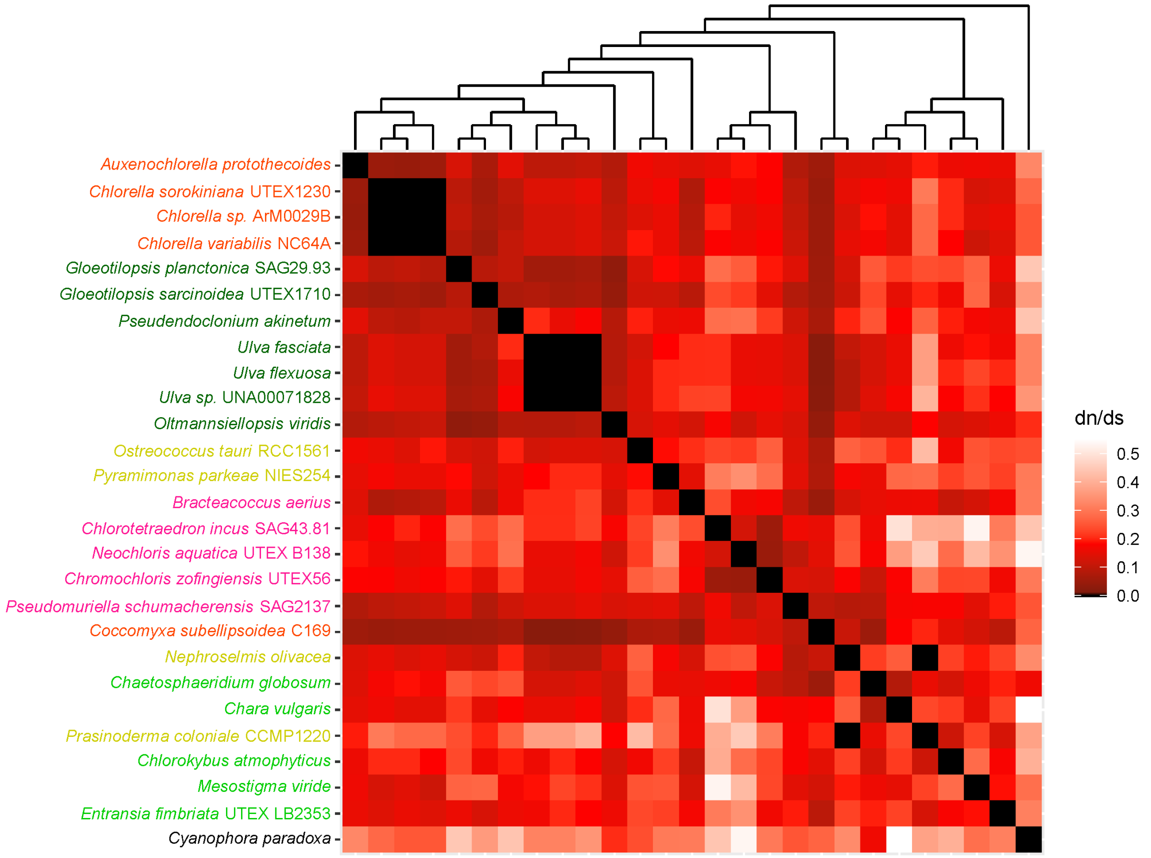

S4 Fig. T. Heatmap of pairwise dN/dS ratios calculated from alignment of plastid *psb*K of algal taxa in Table 1 by MEGA7.0 using modified Nei-Gojobori method (Jukes-Cantor). The scale bar showed the color corresponded to dn/ds ratios. The cladogram above the heatmap corresponded to its single-gene phylogenetic tree.

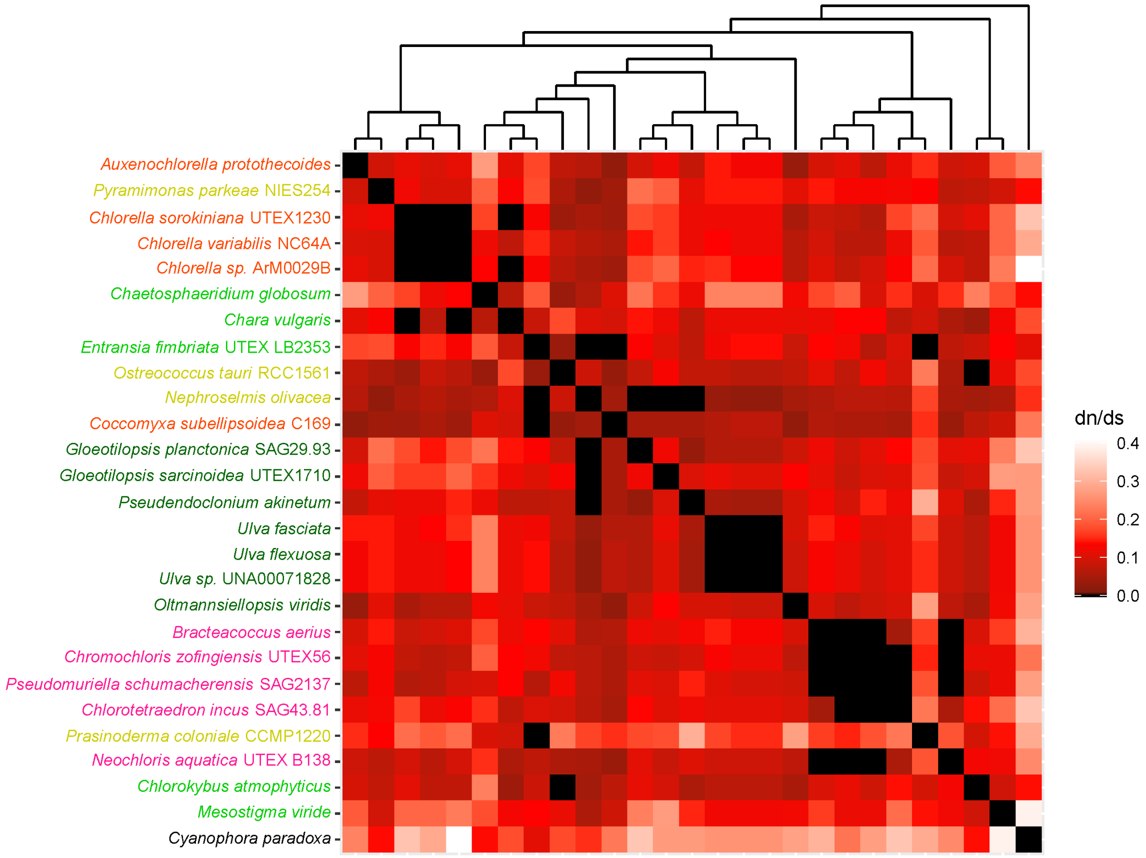

S4 Fig. U. Heatmap of pairwise dN/dS ratios calculated from alignment of plastid *psb*L of algal taxa in Table 1 by MEGA7.0 using modified Nei-Gojobori method (Jukes-Cantor). The scale bar showed the color corresponded to dn/ds ratios. The cladogram above the heatmap corresponded to its single-gene phylogenetic tree.

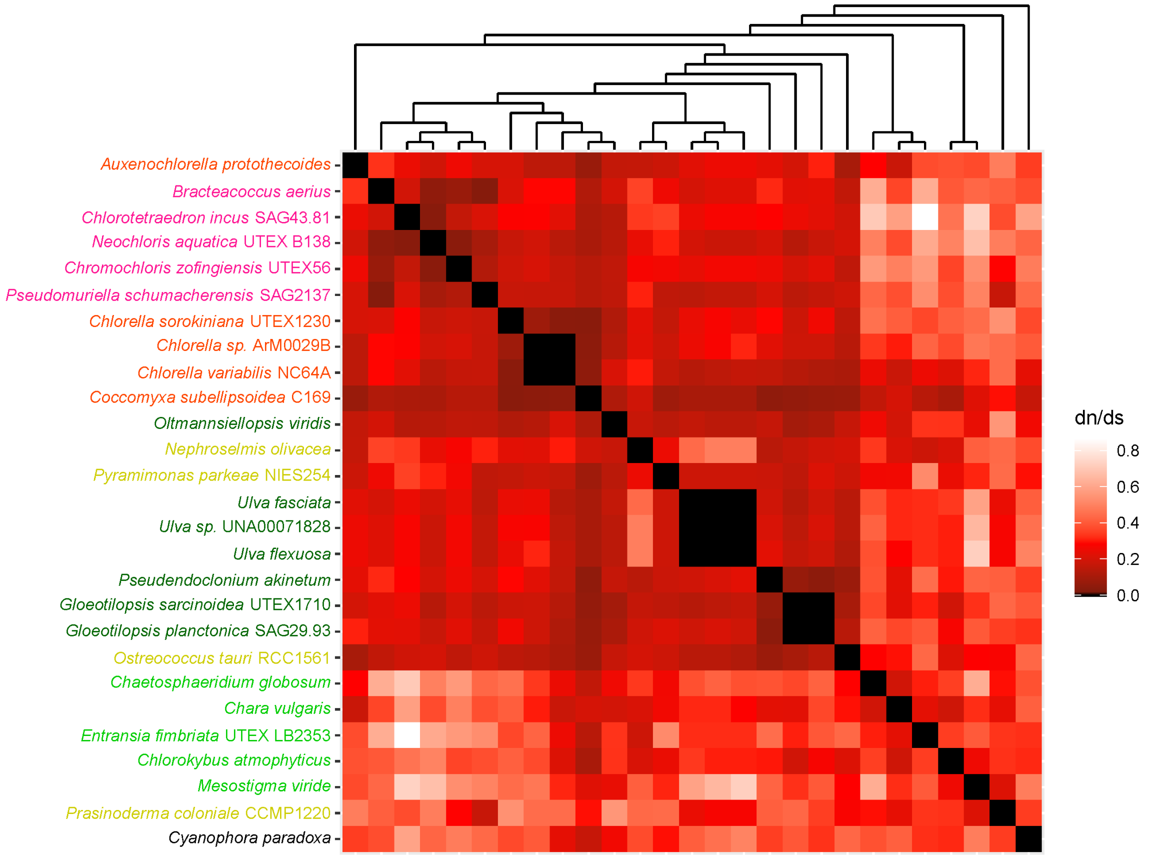

S4 Fig. V. Heatmap of pairwise dN/dS ratios calculated from alignment of plastid *psb*N of algal taxa in Table 1 by MEGA7.0 using modified Nei-Gojobori method (Jukes-Cantor). The scale bar showed the color corresponded to dn/ds ratios. The cladogram above the heatmap corresponded to its single-gene phylogenetic tree.

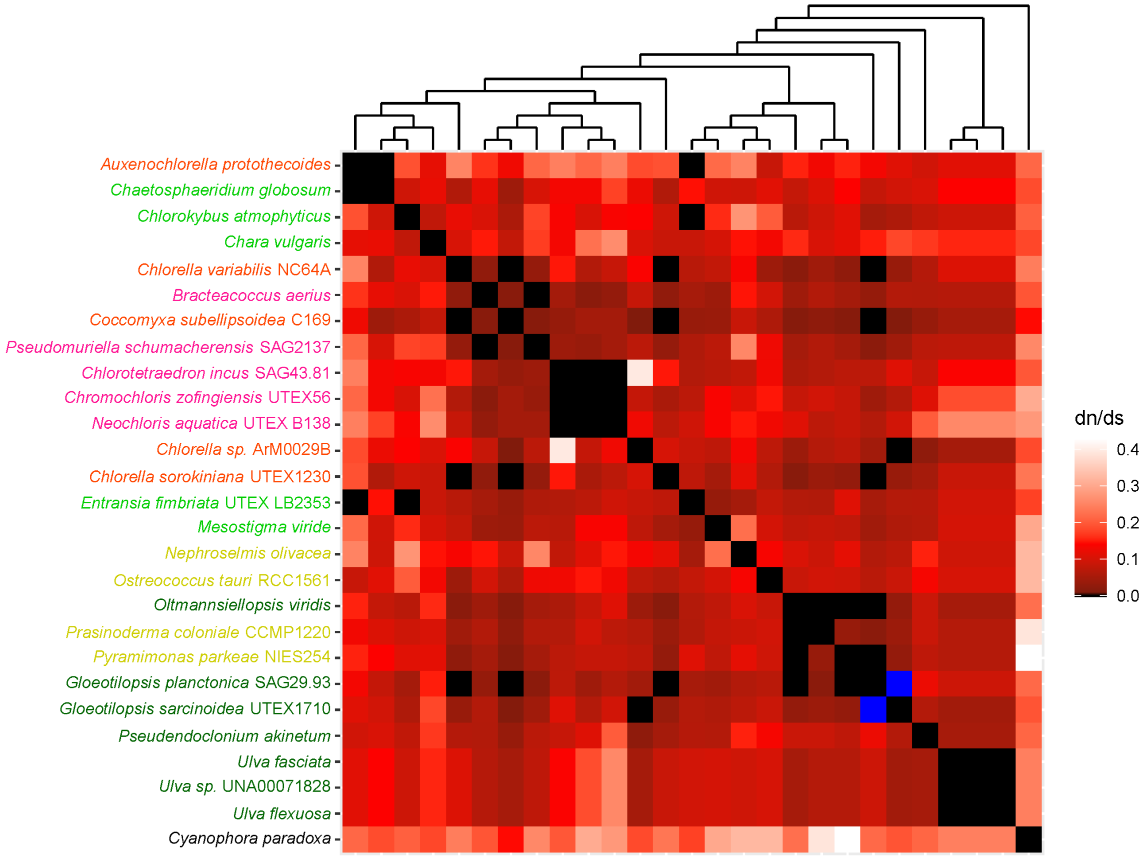

S4 Fig. W. Heatmap of pairwise dN/dS ratios calculated from alignment of plastid *psb*T of algal taxa in Table 1 by MEGA7.0 using modified Nei-Gojobori method (Jukes-Cantor). The scale bar showed the color corresponded to dn/ds ratios. The cladogram above the heatmap corresponded to its single-gene phylogenetic tree.

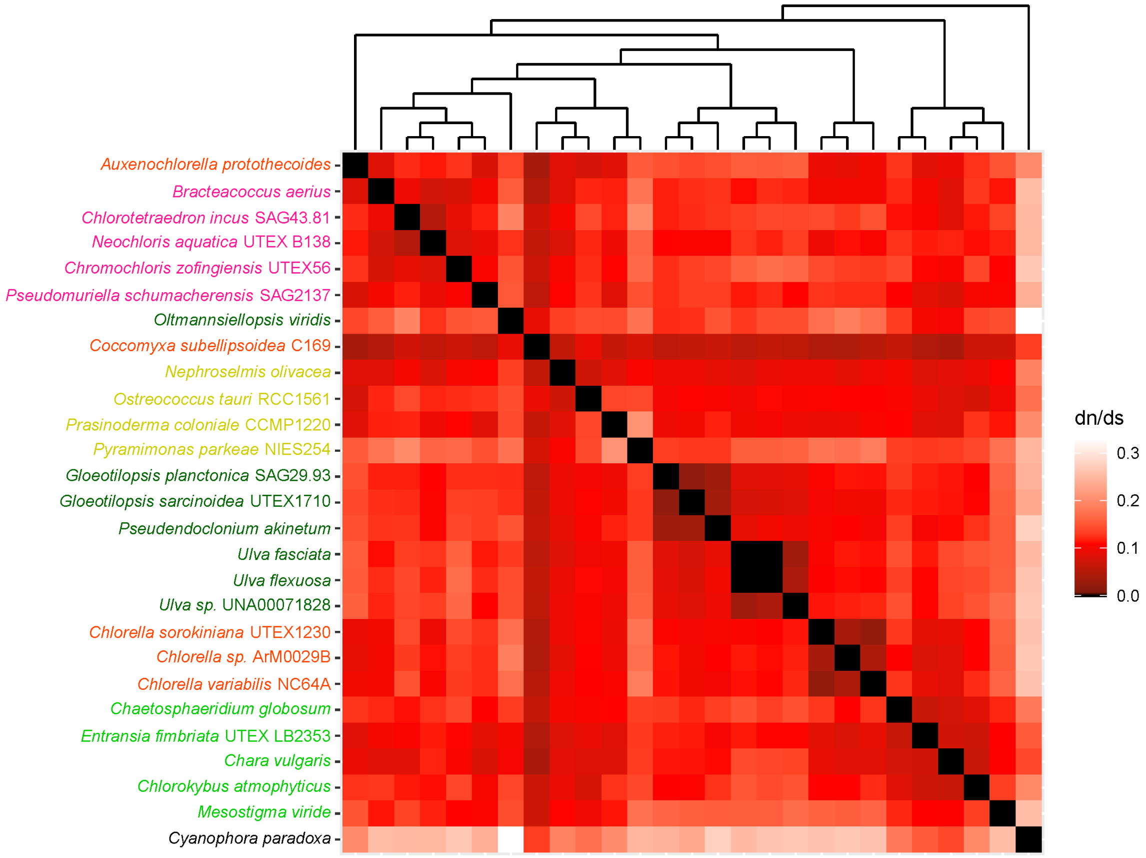

S4 Fig. X. Heatmap of pairwise dN/dS ratios calculated from alignment of plastid *rbc*L of algal taxa in Table 1 by MEGA7.0 using modified Nei-Gojobori method (Jukes-Cantor). The scale bar showed the color corresponded to dn/ds ratios. The cladogram above the heatmap corresponded to its single-gene phylogenetic tree.

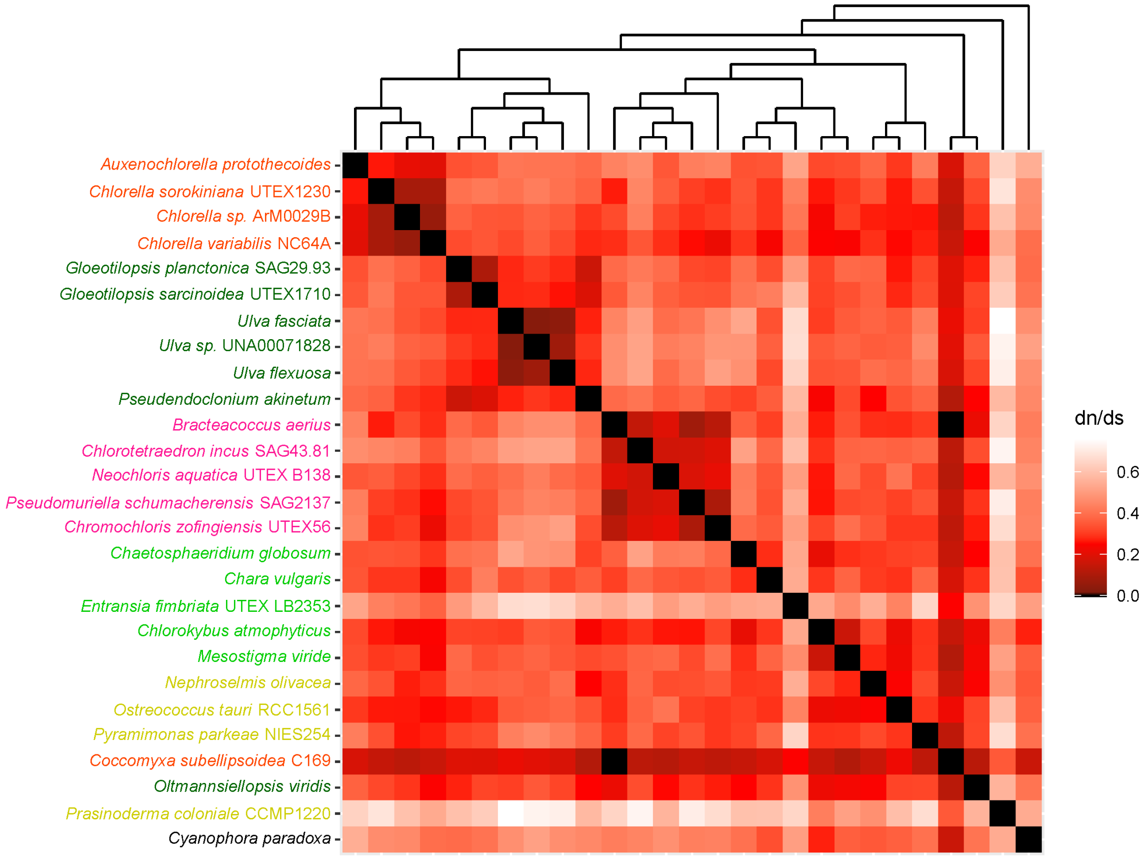

S4 Fig. Y. Heatmap of pairwise dN/dS ratios calculated from alignment of plastid *rpl*2 of algal taxa in Table 1 by MEGA7.0 using modified Nei-Gojobori method (Jukes-Cantor). The scale bar showed the color corresponded to dn/ds ratios. The cladogram above the heatmap corresponded to its single-gene phylogenetic tree.

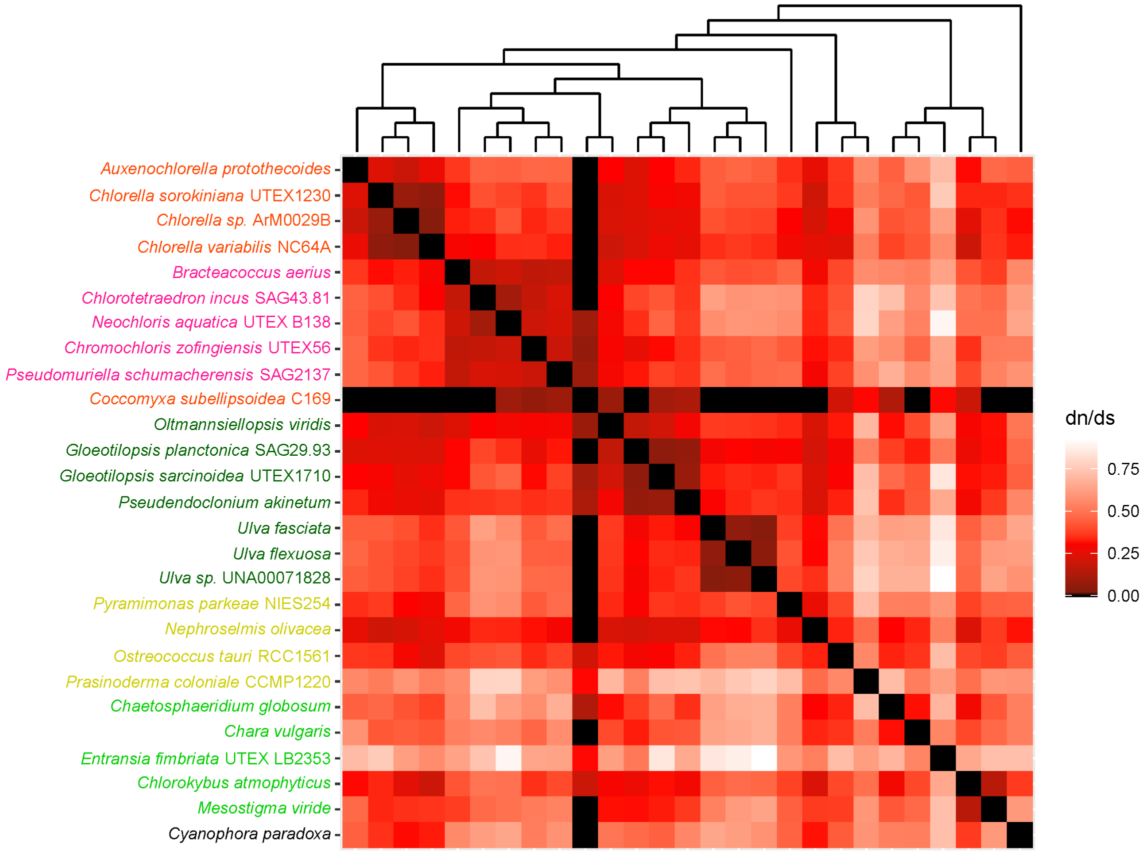

S4 Fig. Z. Heatmap of pairwise dN/dS ratios calculated from alignment of plastid *rpl*5 of algal taxa in Table 1 by MEGA7.0 using modified Nei-Gojobori method (Jukes-Cantor). The scale bar showed the color corresponded to dn/ds ratios. The cladogram above the heatmap corresponded to its single-gene phylogenetic tree.

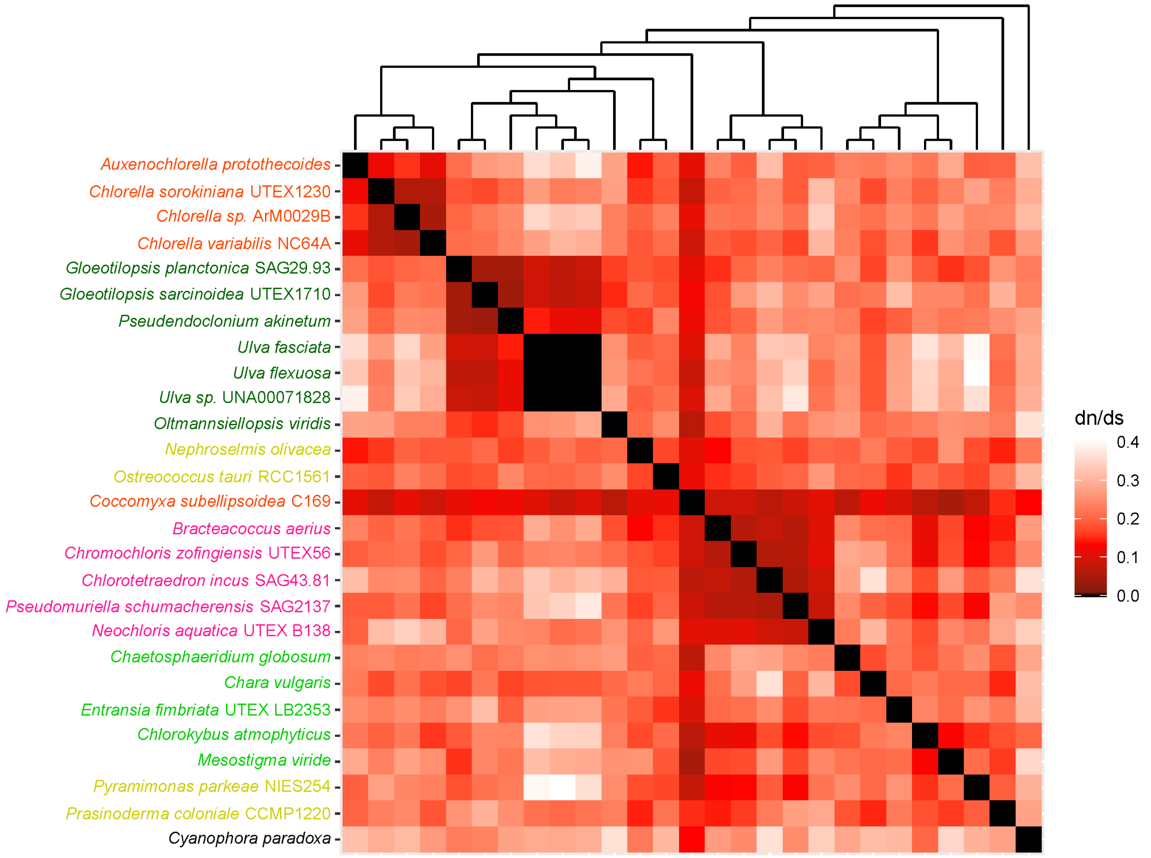

S4 Fig. AA. Heatmap of pairwise dN/dS ratios calculated from alignment of plastid *rpl*14 of algal taxa in Table 1 by MEGA7.0 using modified Nei-Gojobori method (Jukes-Cantor). The scale bar showed the color corresponded to dn/ds ratios. The cladogram above the heatmap corresponded to its single-gene phylogenetic tree.

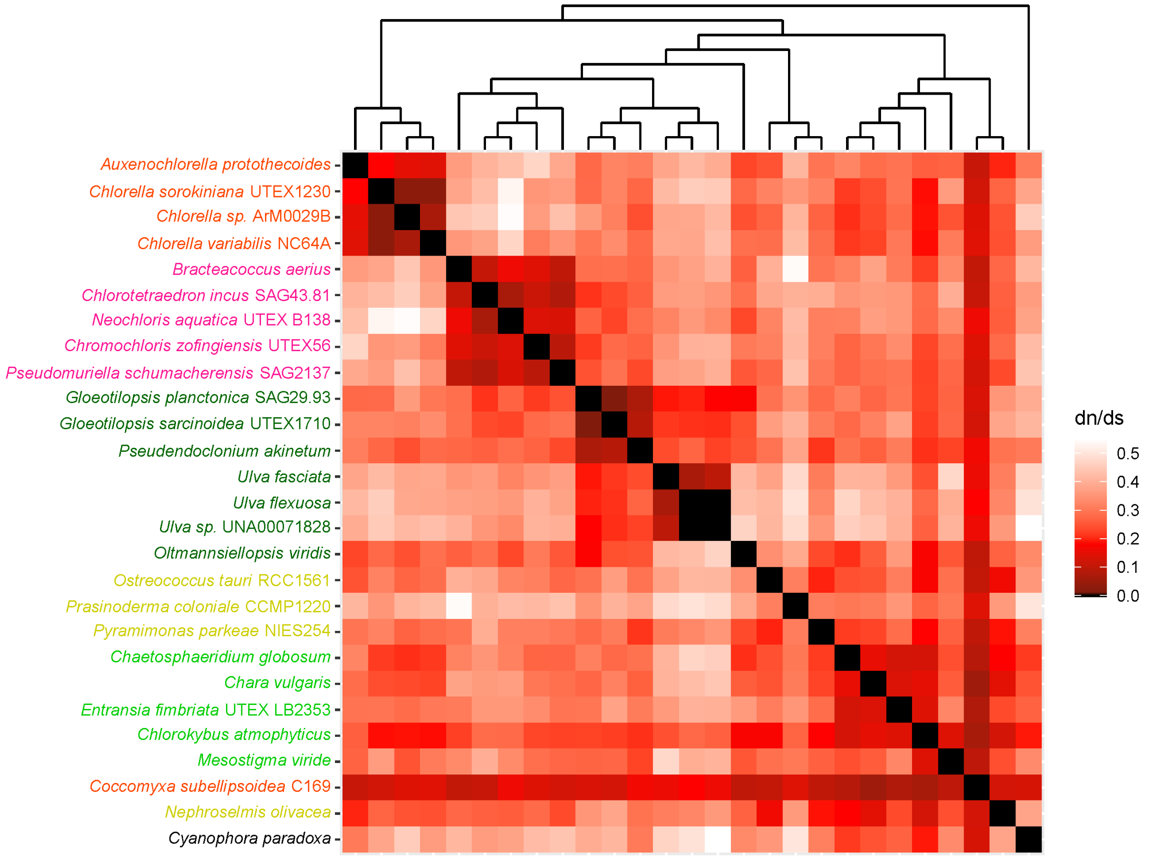

S4 Fig. AB. Heatmap of pairwise dN/dS ratios calculated from alignment of plastid *rpl*16 of algal taxa in Table 1 by MEGA7.0 using modified Nei-Gojobori method (Jukes-Cantor). The scale bar showed the color corresponded to dn/ds ratios. The cladogram above the heatmap corresponded to its single-gene phylogenetic tree.

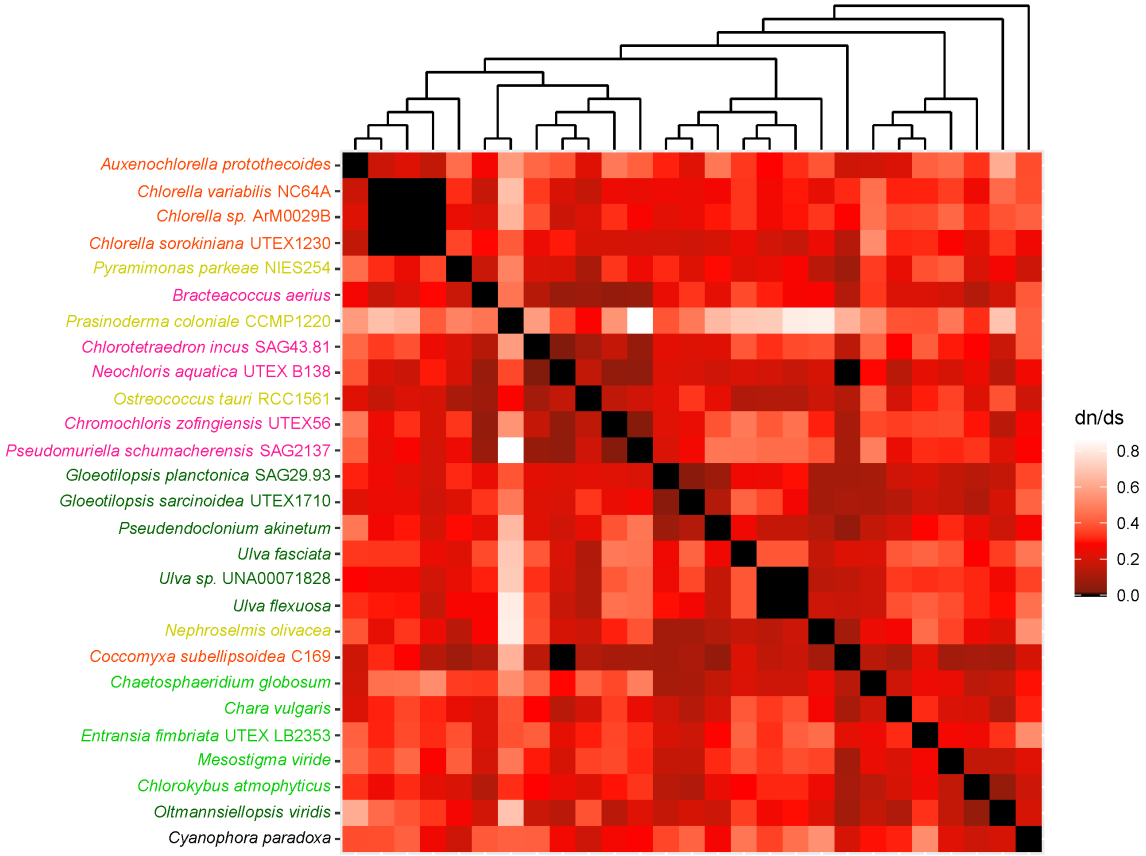

S4 Fig. AC Heatmap of pairwise dN/dS ratios calculated from alignment of plastid *rpl*36 of algal taxa in Table 1 by MEGA7.0 using modified Nei-Gojobori method (Jukes-Cantor). The scale bar showed the color corresponded to dn/ds ratios. The cladogram above the heatmap corresponded to its single-gene phylogenetic tree.

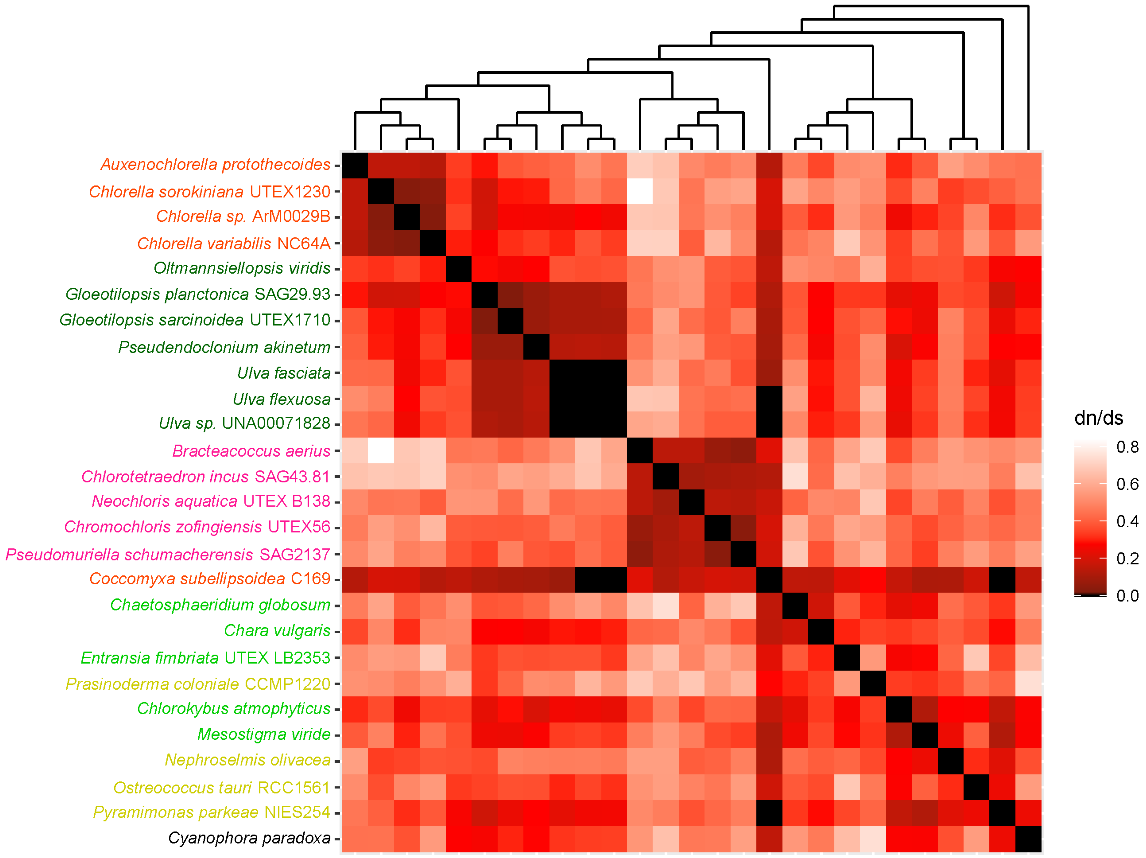

S4 Fig. AD. Heatmap of pairwise dN/dS ratios calculated from alignment of plastid *rps*11 of algal taxa in Table 1 by MEGA7.0 using modified Nei-Gojobori method (Jukes-Cantor). The scale bar showed the color corresponded to dn/ds ratios. The cladogram above the heatmap corresponded to its single-gene phylogenetic tree.

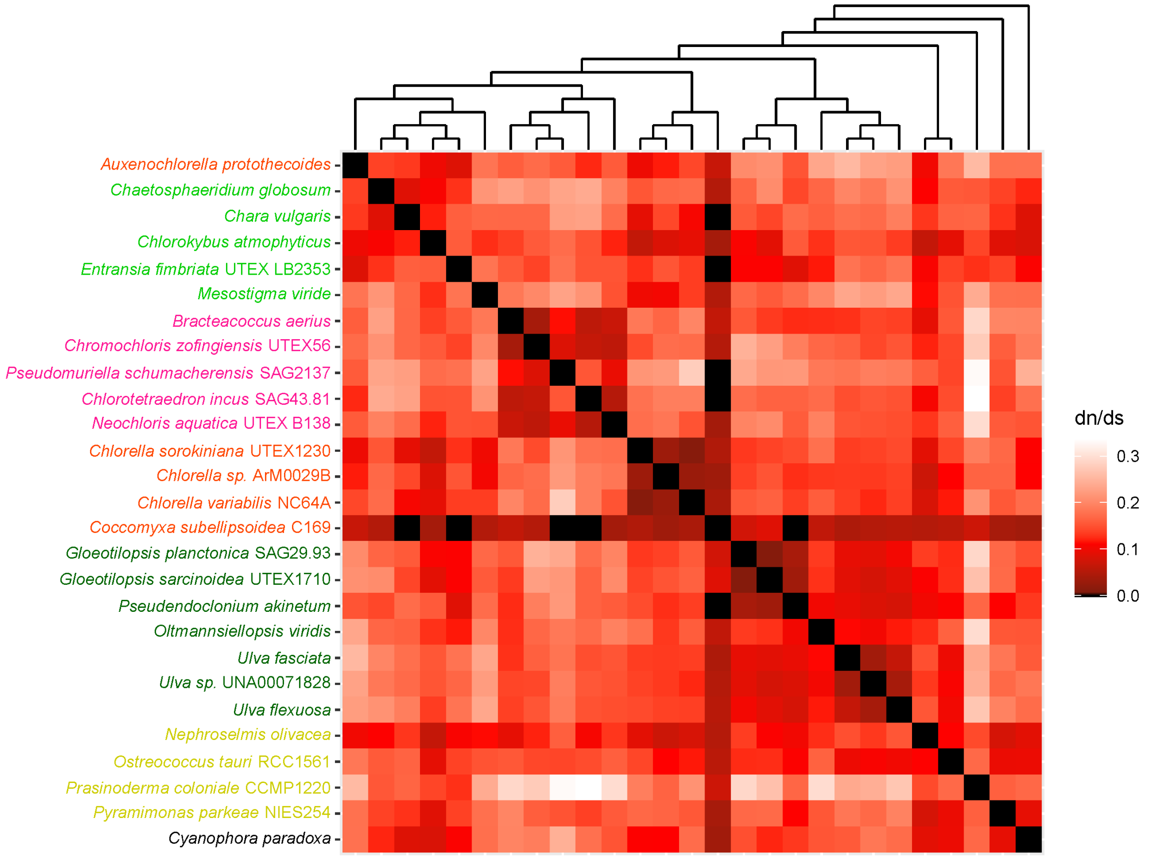

S4 Fig. AE. Heatmap of pairwise dN/dS ratios calculated from alignment of plastid *rps*12 of algal taxa in Table 1 by MEGA7.0 using modified Nei-Gojobori method (Jukes-Cantor). The scale bar showed the color corresponded to dn/ds ratios. The cladogram above the heatmap corresponded to its single-gene phylogenetic tree.

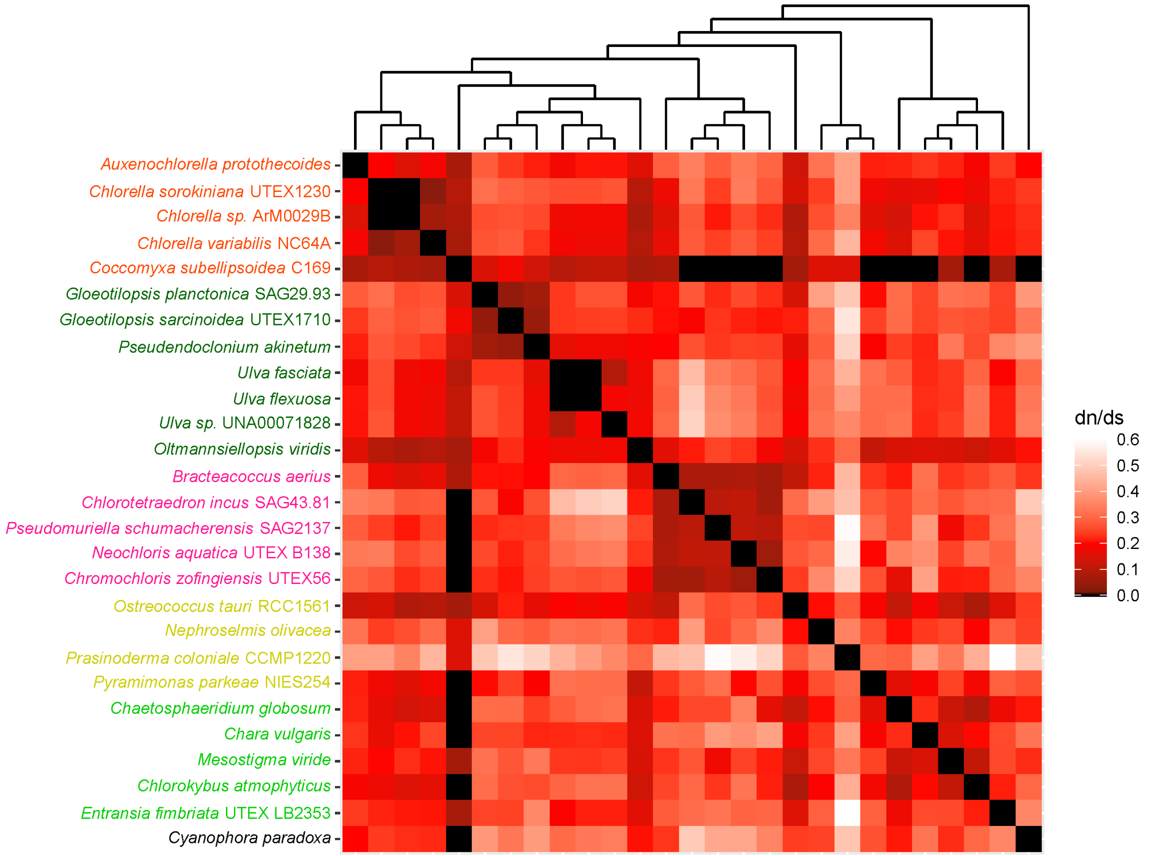

S4 Fig. AF. Heatmap of pairwise dN/dS ratios calculated from alignment of plastid *rps*19 of algal taxa in Table 1 by MEGA7.0 using modified Nei-Gojobori method (Jukes-Cantor). The scale bar showed the color corresponded to dn/ds ratios. The cladogram above the heatmap corresponded to its single-gene phylogenetic tree.

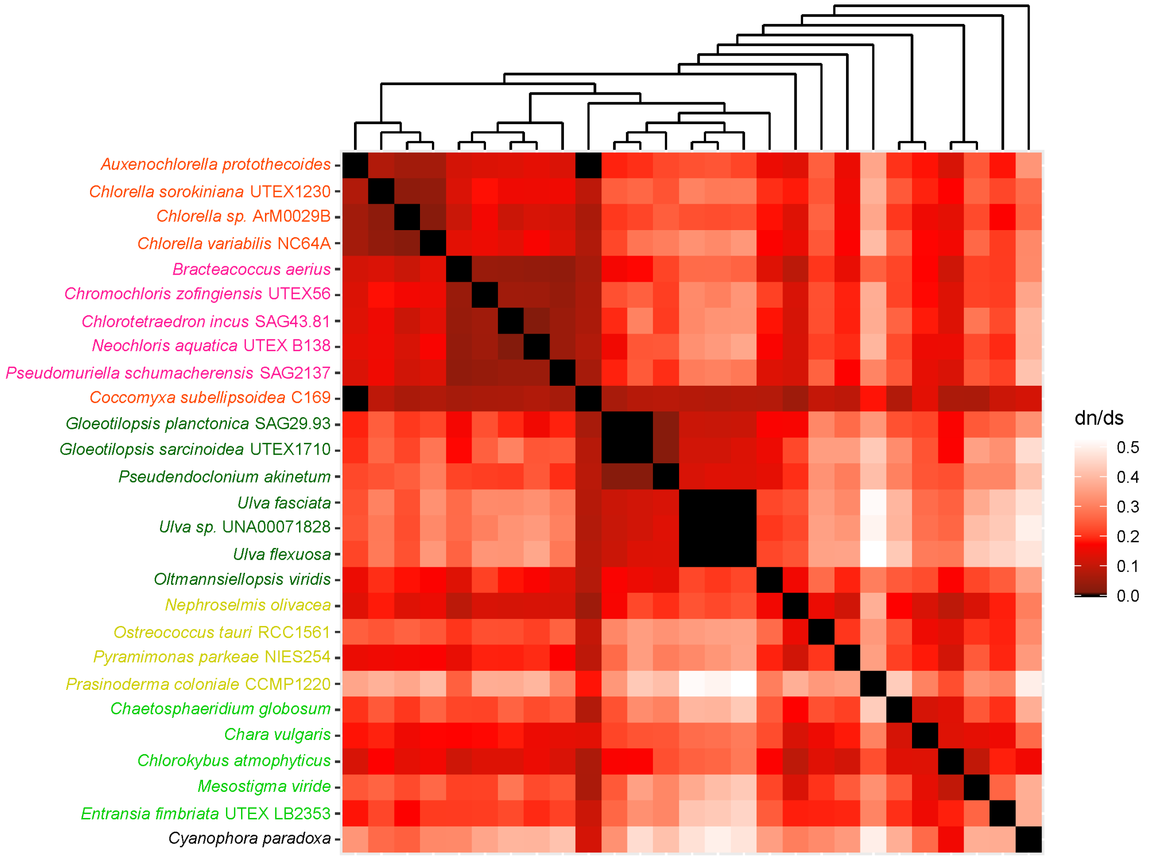

S4 Fig. AG. Heatmap of pairwise dN/dS ratios calculated from alignment of plastid *ycf3* of algal taxa in Table 1 by MEGA7.0 using modified Nei-Gojobori method (Jukes-Cantor). The scale bar showed the color corresponded to dn/ds ratios. The cladogram above the heatmap corresponded to its single-gene phylogenetic tree.

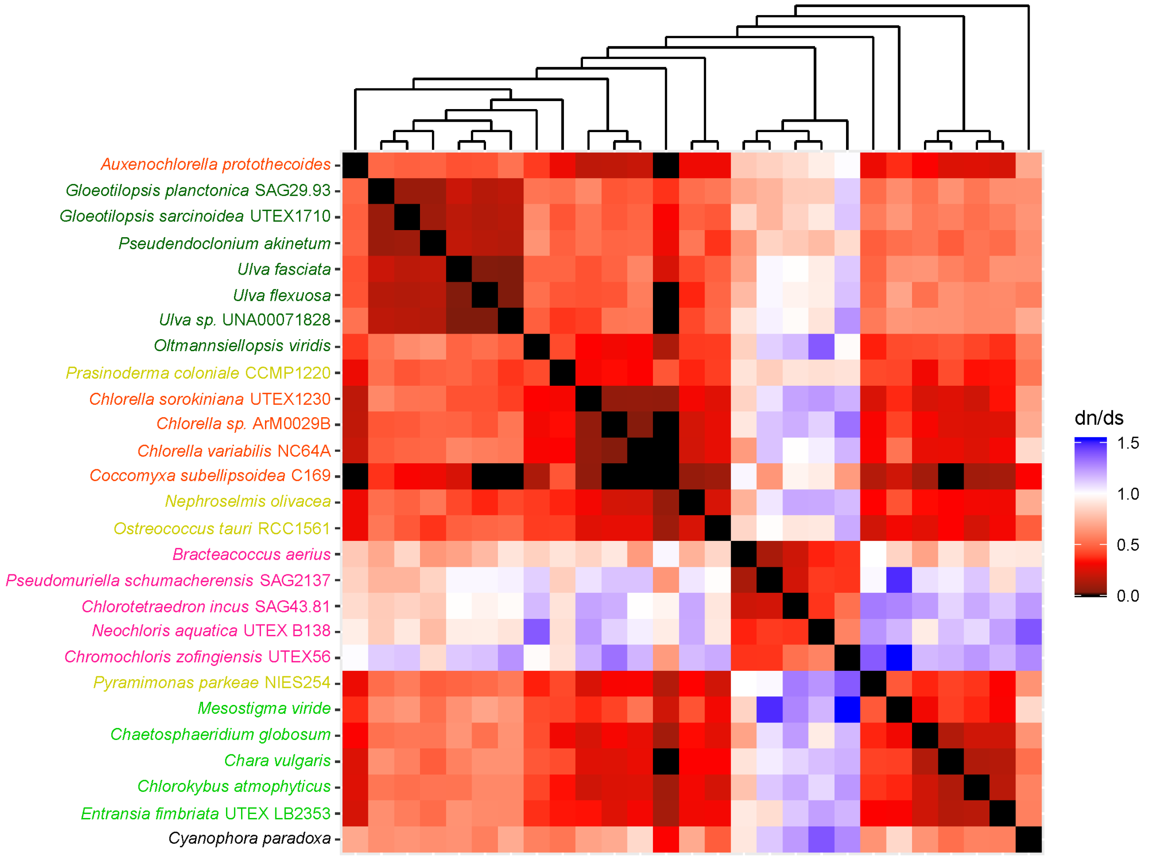

S4 Fig. AH. Heatmap of pairwise dN/dS ratios calculated from alignment of mitochondrial *atp6* of algal taxa in Table 1 by MEGA7.0 using modified Nei-Gojobori method (Jukes-Cantor). The scale bar showed the color corresponded to dn/ds ratios. The cladogram above the heatmap corresponded to its single-gene phylogenetic tree.

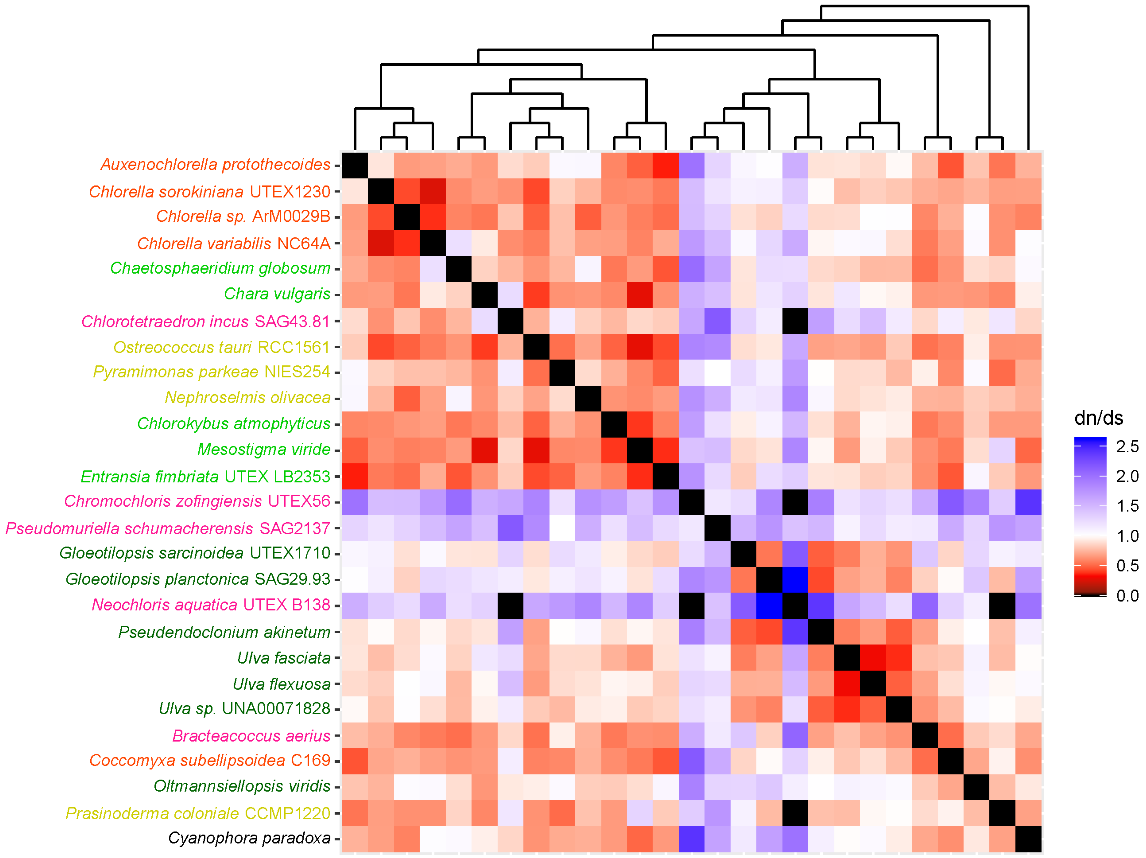

S4 Fig. AI. Heatmap of pairwise dN/dS ratios calculated from alignment of mitochondrial *cob* of algal taxa in Table 1 by MEGA7.0 using modified Nei-Gojobori method (Jukes-Cantor). The scale bar showed the color corresponded to dn/ds ratios. The cladogram above the heatmap corresponded to its single-gene phylogenetic tree.

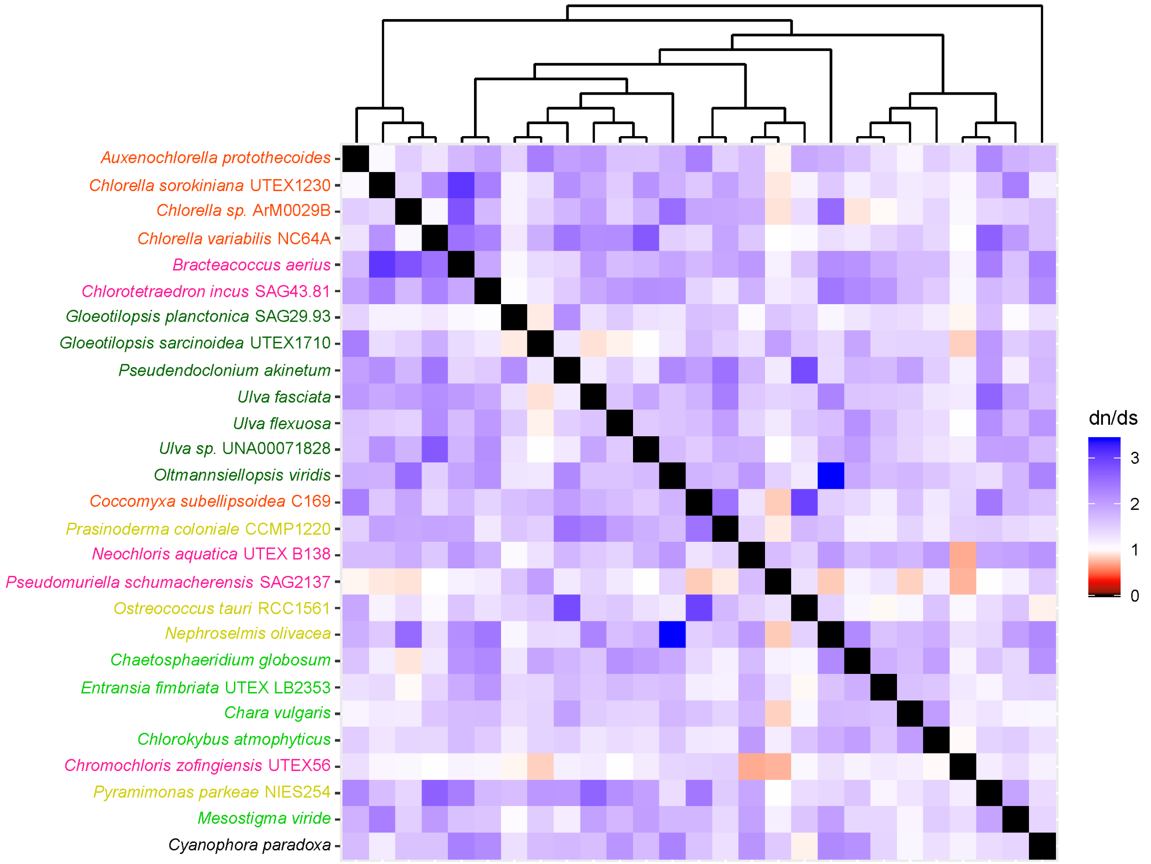

S4 Fig. AJ. Heatmap of pairwise dN/dS ratios calculated from alignment of mitochondrial *cox*1 of algal taxa in Table 1 by MEGA7.0 using modified Nei-Gojobori method (Jukes-Cantor). The scale bar showed the color corresponded to dn/ds ratios. The cladogram above the heatmap corresponded to its single-gene phylogenetic tree.

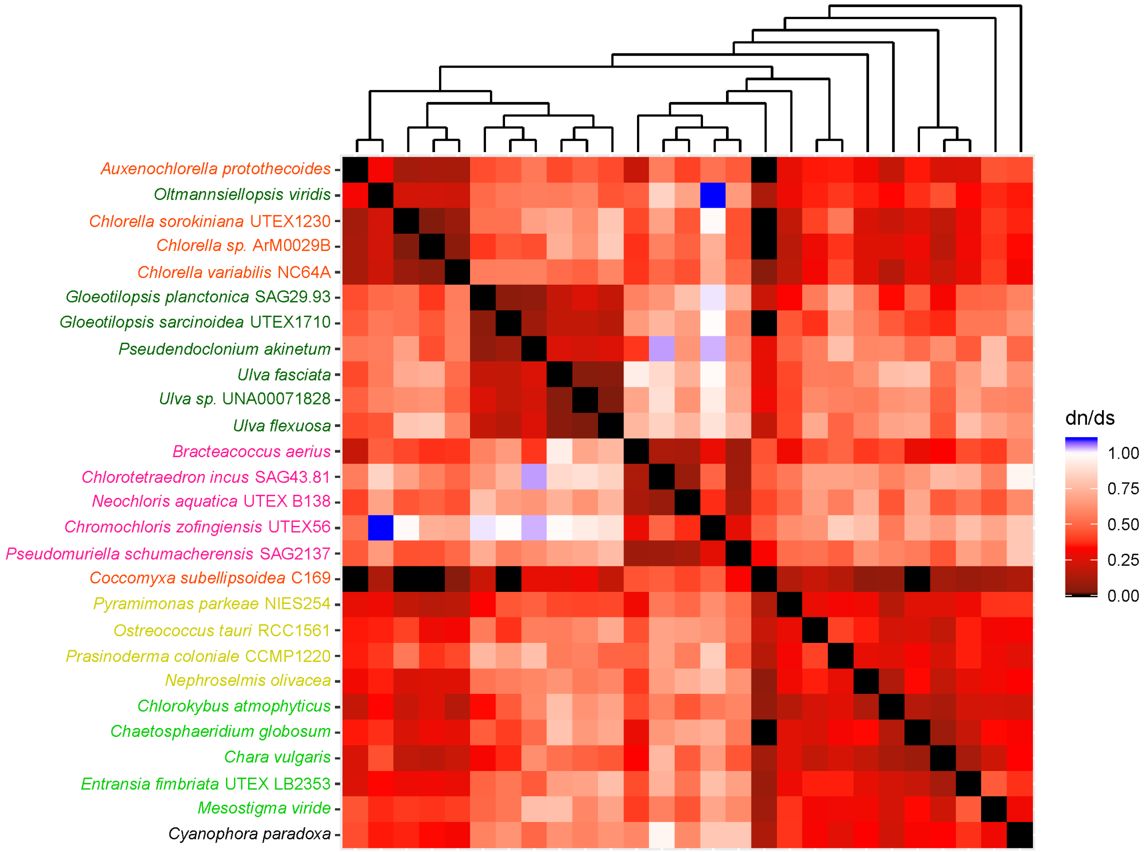

S4 Fig. AK. Heatmap of pairwise dN/dS ratios calculated from alignment of mitochondrial *cox*2 of algal taxa in Table 1 by MEGA7.0 using modified Nei-Gojobori method (Jukes-Cantor). The scale bar showed the color corresponded to dn/ds ratios. The cladogram above the heatmap corresponded to its single-gene phylogenetic tree.

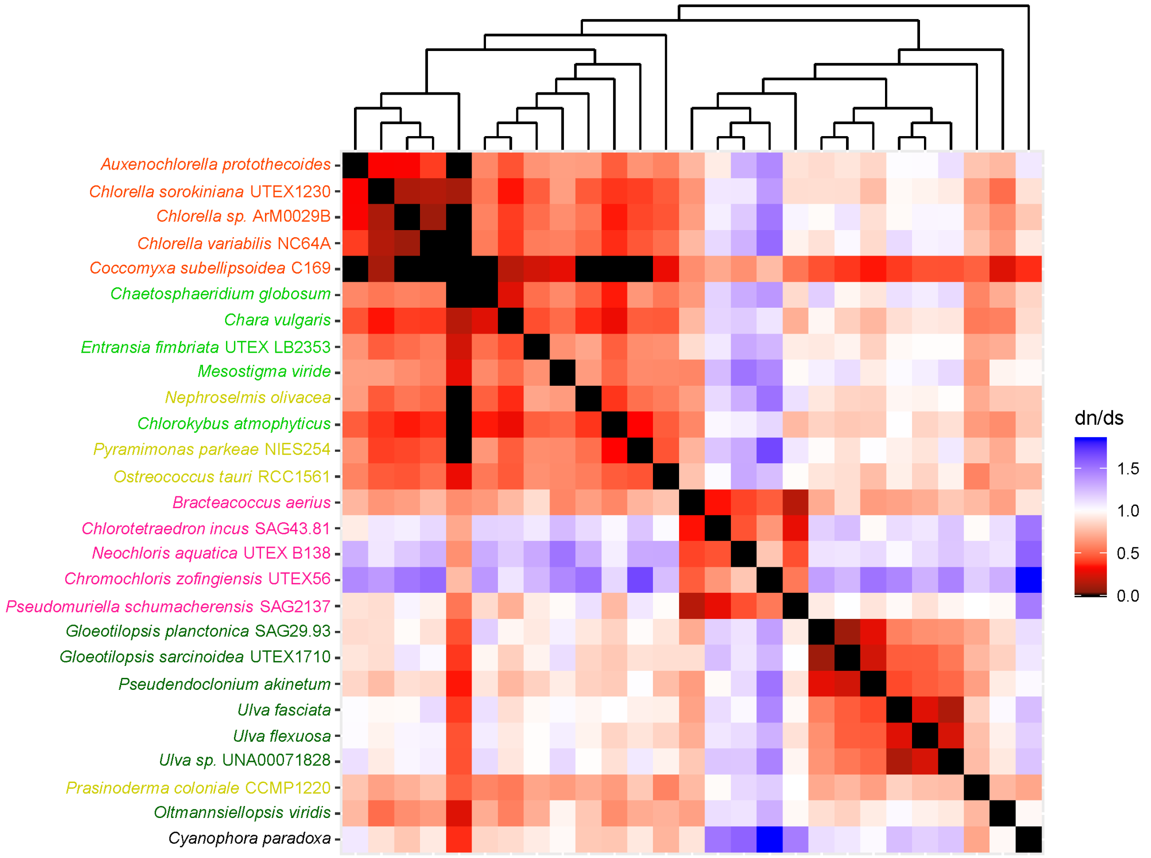

S4 Fig. AL. Heatmap of pairwise dN/dS ratios calculated from alignment of mitochondrial *nad*2 of algal taxa in Table 1 by MEGA7.0 using modified Nei-Gojobori method (Jukes-Cantor). The scale bar showed the color corresponded to dn/ds ratios. The cladogram above the heatmap corresponded to its single-gene phylogenetic tree.

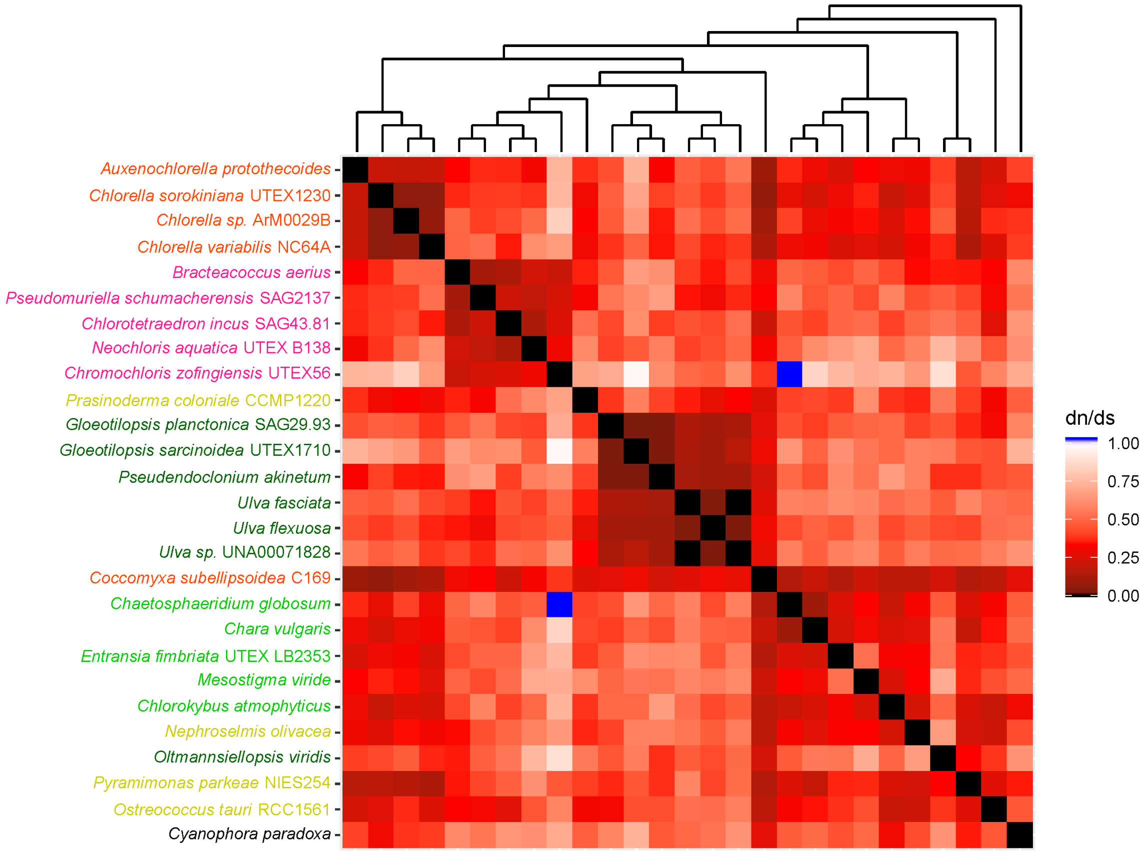

S4 Fig. AM. Heatmap of pairwise dN/dS ratios calculated from alignment of mitochondrial *nad3* of algal taxa in Table 1 by MEGA7.0 using modified Nei-Gojobori method (Jukes-Cantor). The scale bar showed the color corresponded to dn/ds ratios. The cladogram above the heatmap corresponded to its single-gene phylogenetic tree.

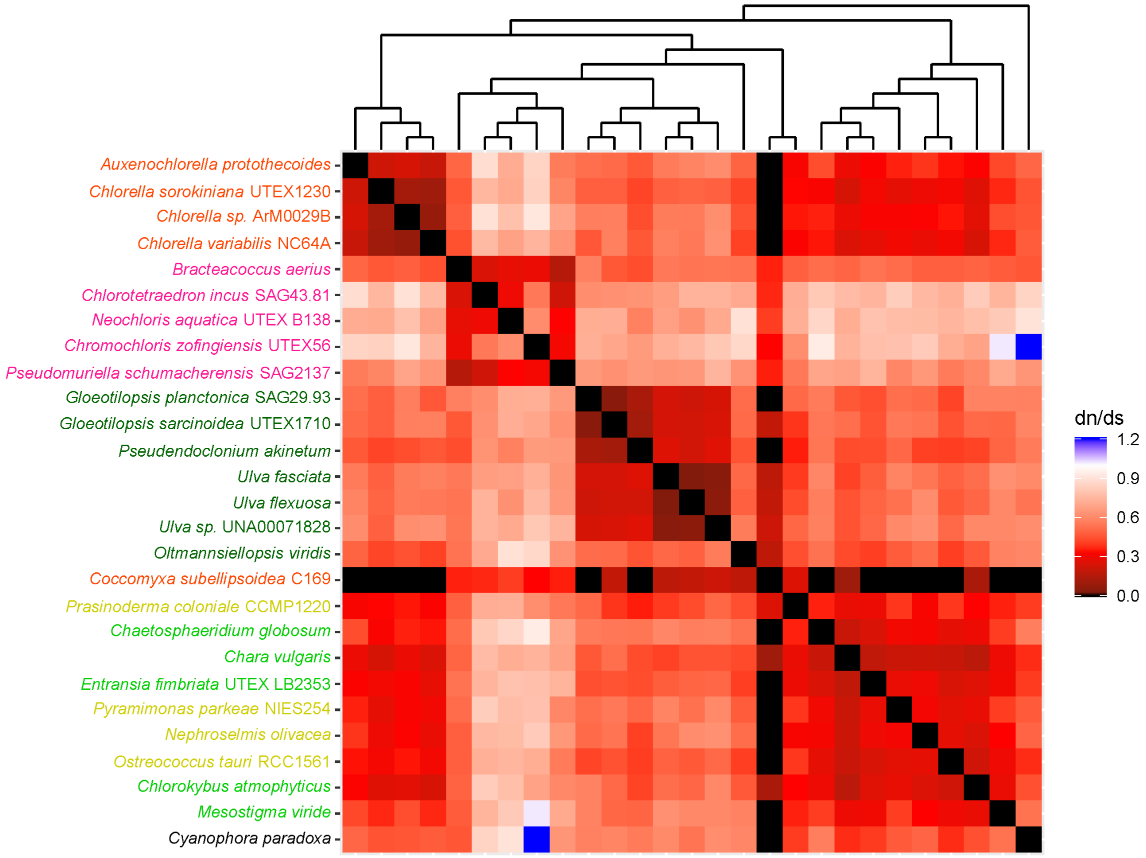

S4 Fig. AN. Heatmap of pairwise dN/dS ratios calculated from alignment of mitochondrial *nad*4 of algal taxa in Table 1 by MEGA7.0 using modified Nei-Gojobori method (Jukes-Cantor). The scale bar showed the color corresponded to dn/ds ratios. The cladogram above the heatmap corresponded to its single-gene phylogenetic tree.

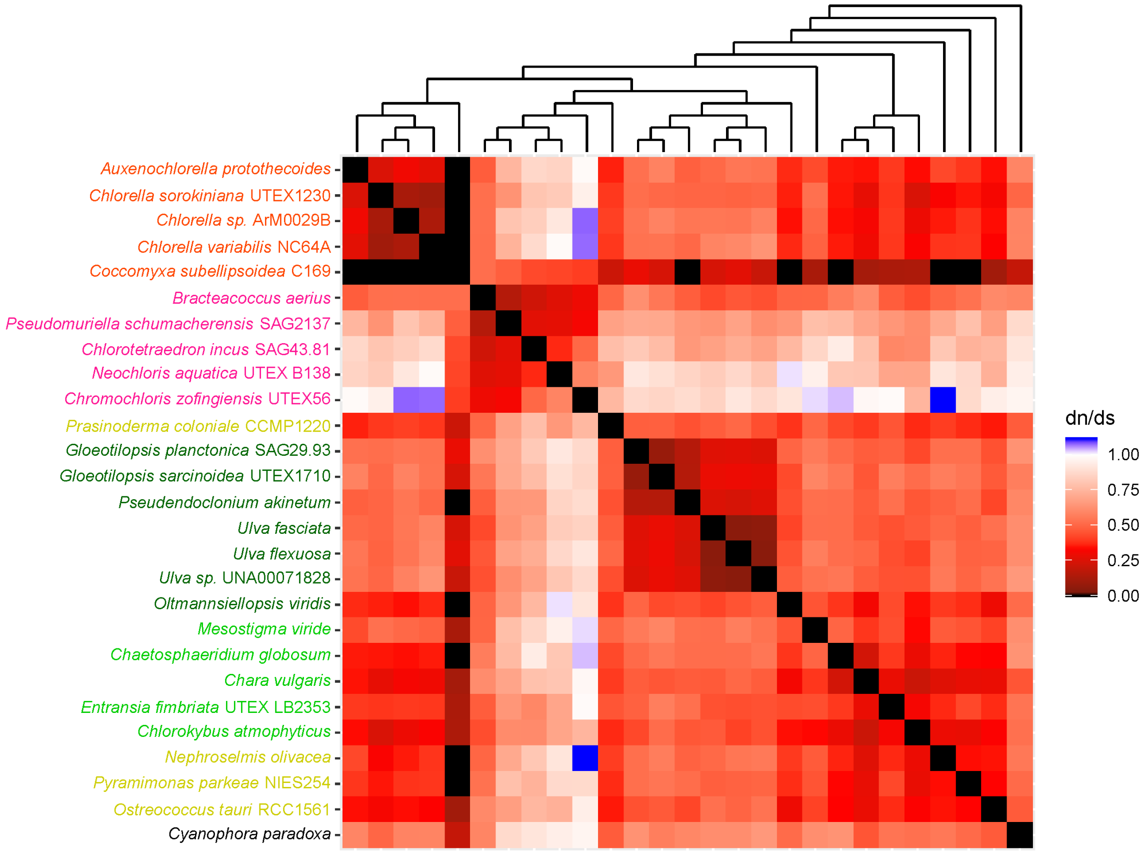

S4 Fig. AO. Heatmap of pairwise dN/dS ratios calculated from alignment of mitochondrial *nad*5 of algal taxa in Table 1 by MEGA7.0 using modified Nei-Gojobori method (Jukes-Cantor). The scale bar showed the color corresponded to dn/ds ratios. The cladogram above the heatmap corresponded to its single-gene phylogenetic tree.

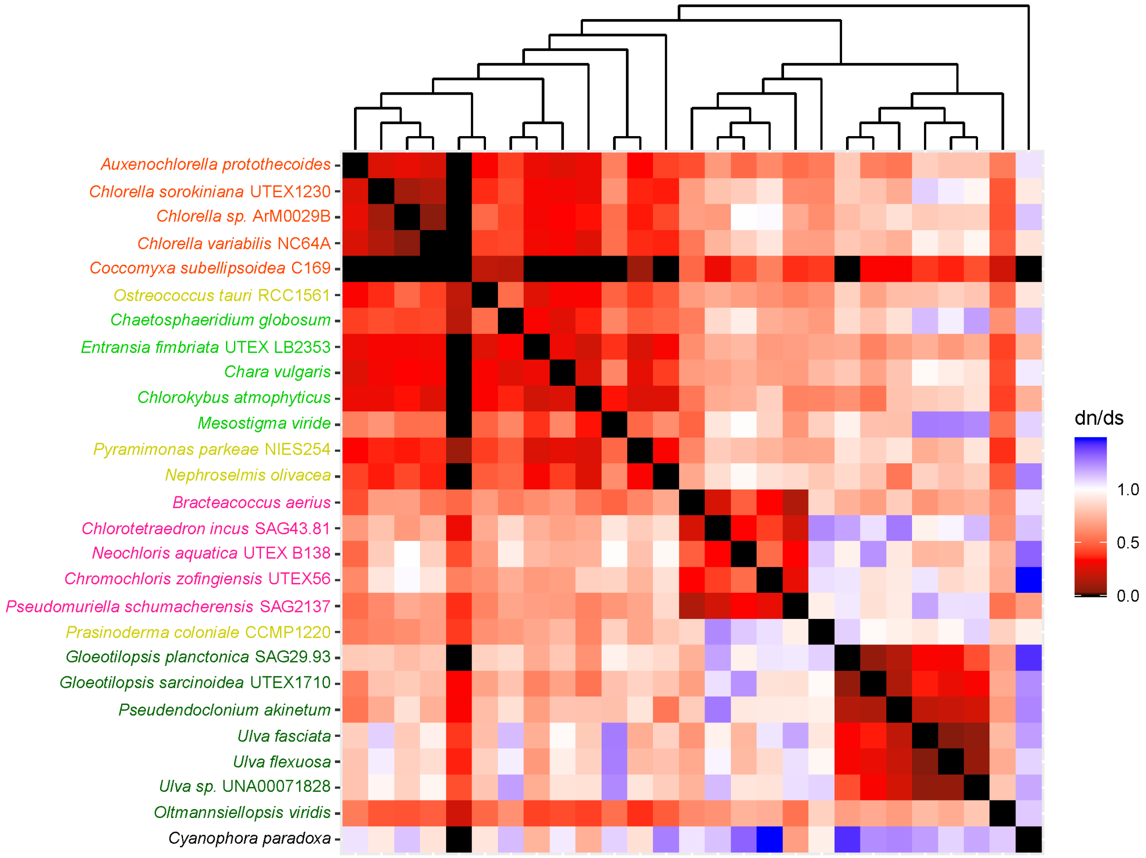

S4 Fig. AP. Heatmap of pairwise dN/dS ratios calculated from alignment of mitochondrial *nad6* of algal taxa in Table 1 by MEGA7.0 using modified Nei-Gojobori method (Jukes-Cantor). The scale bar showed the color corresponded to dn/ds ratios. The cladogram above the heatmap corresponded to its single-gene phylogenetic tree.

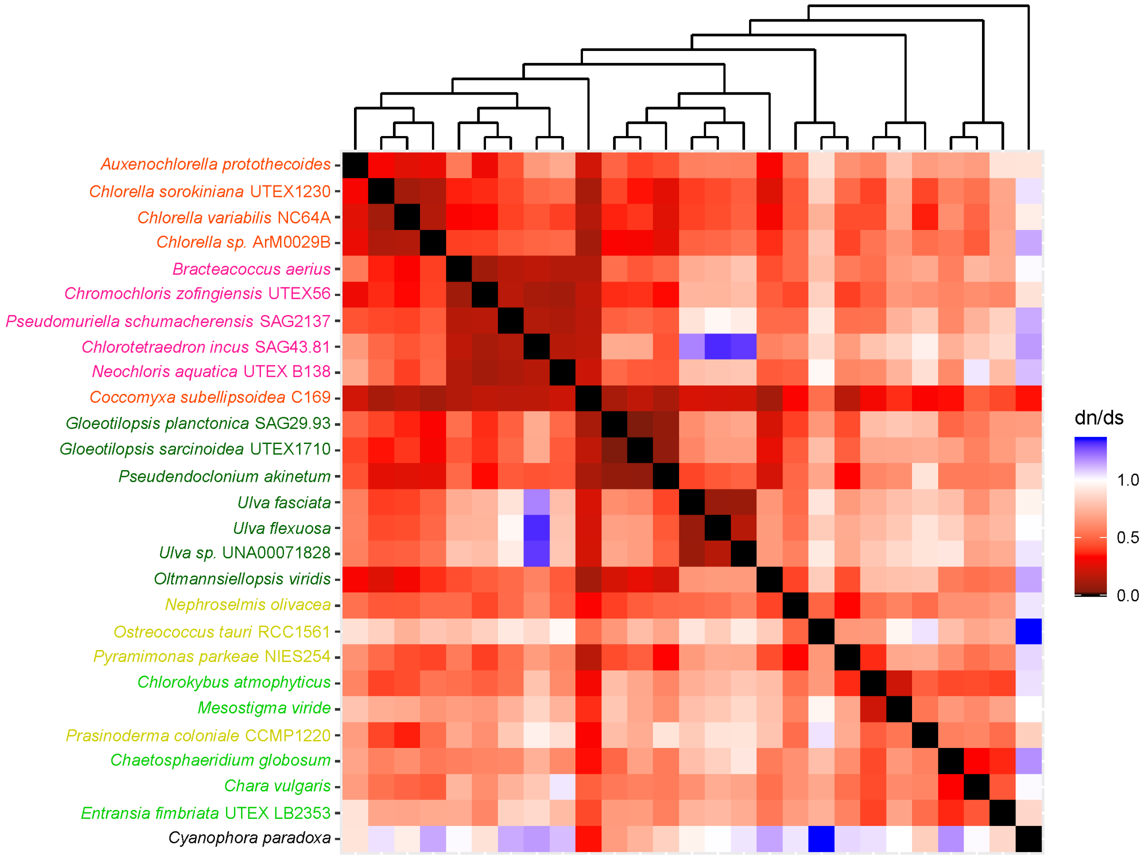

S4 Fig. AQ. Heatmap of pairwise dN/dS ratios calculated from alignment of plastid *atp*E of algal taxa in Table 1 by MEGA7.0 using modified Nei-Gojobori method (Jukes-Cantor). The scale bar showed the color corresponded to dn/ds ratios. The cladogram above the heatmap corresponded to its single-gene phylogenetic tree.

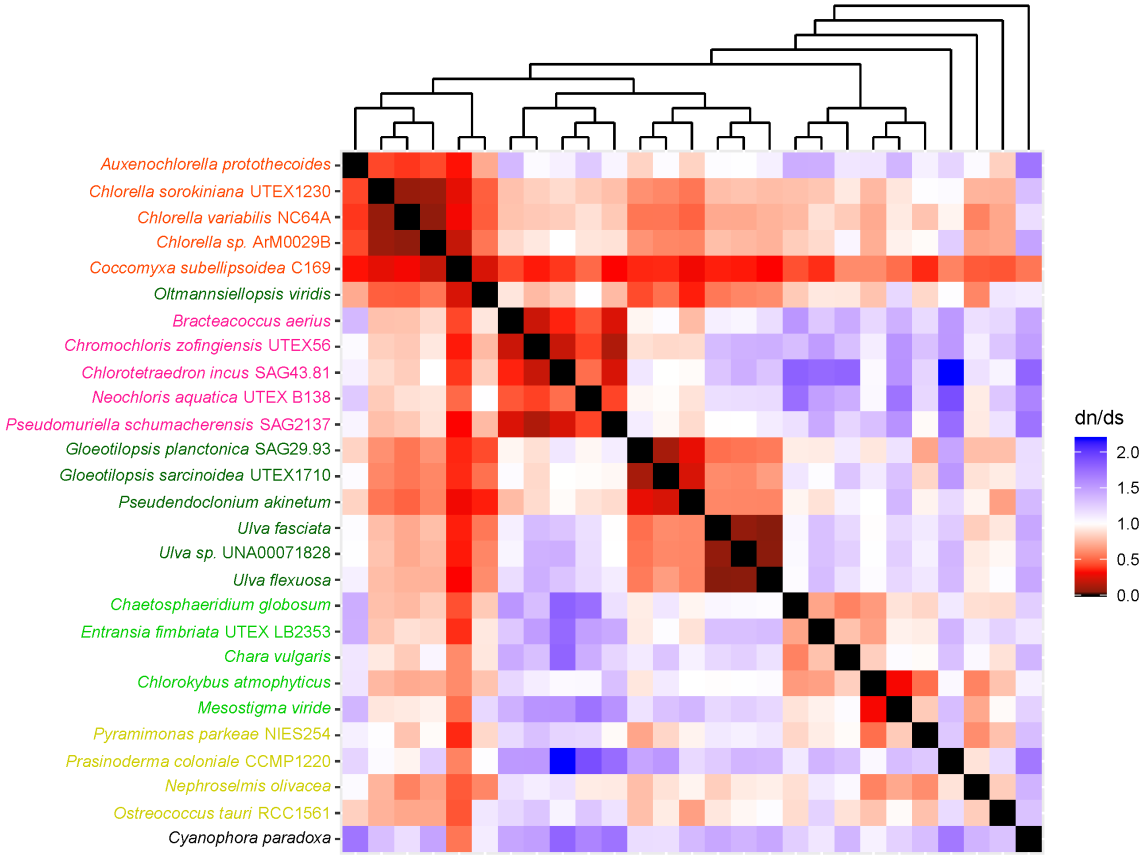

S4 Fig. AR. Heatmap of pairwise dN/dS ratios calculated from alignment of plastid *atp*F of algal taxa in Table 1 by MEGA7.0 using modified Nei-Gojobori method (Jukes-Cantor). The scale bar showed the color corresponded to dn/ds ratios. The cladogram above the heatmap corresponded to its single-gene phylogenetic tree.

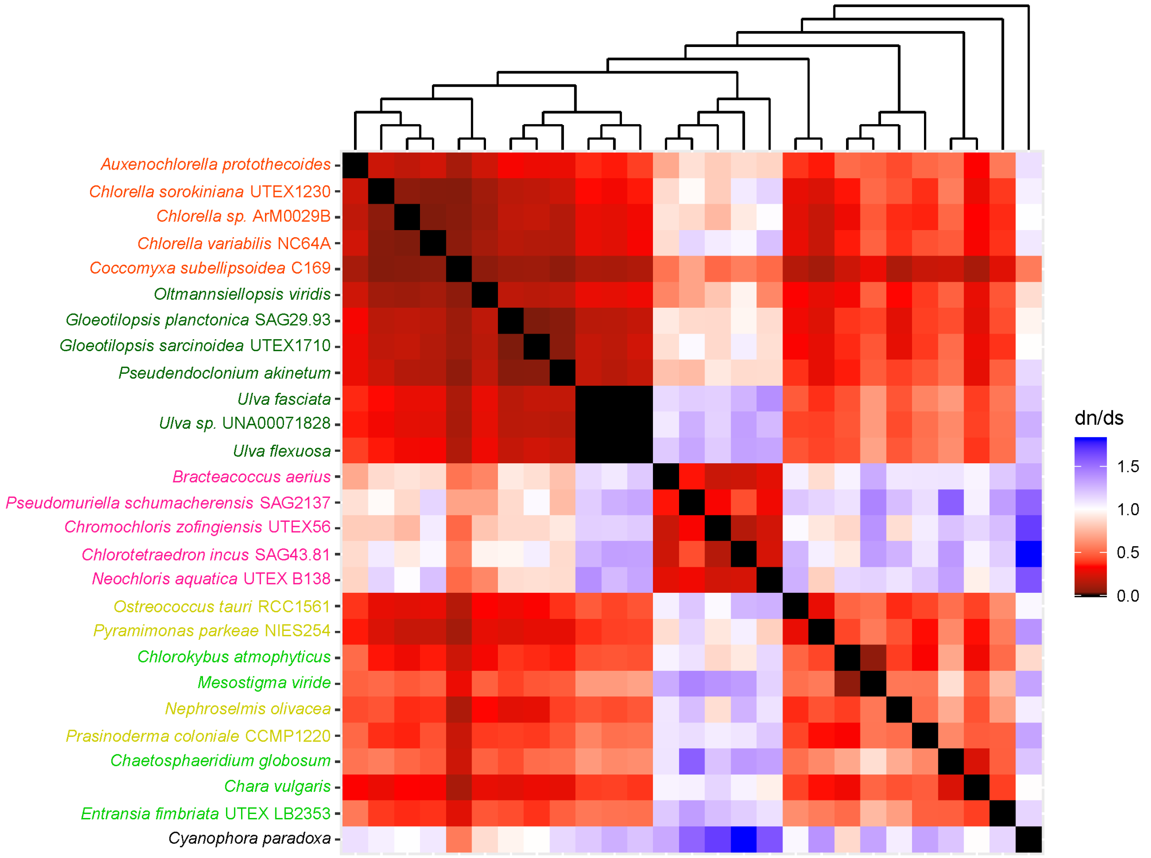

S4 Fig. AS. Heatmap of pairwise dN/dS ratios calculated from alignment of plastid *clp*P of algal taxa in Table 1 by MEGA7.0 using modified Nei-Gojobori method (Jukes-Cantor). The scale bar showed the color corresponded to dn/ds ratios. The cladogram above the heatmap corresponded to its single-gene phylogenetic tree.

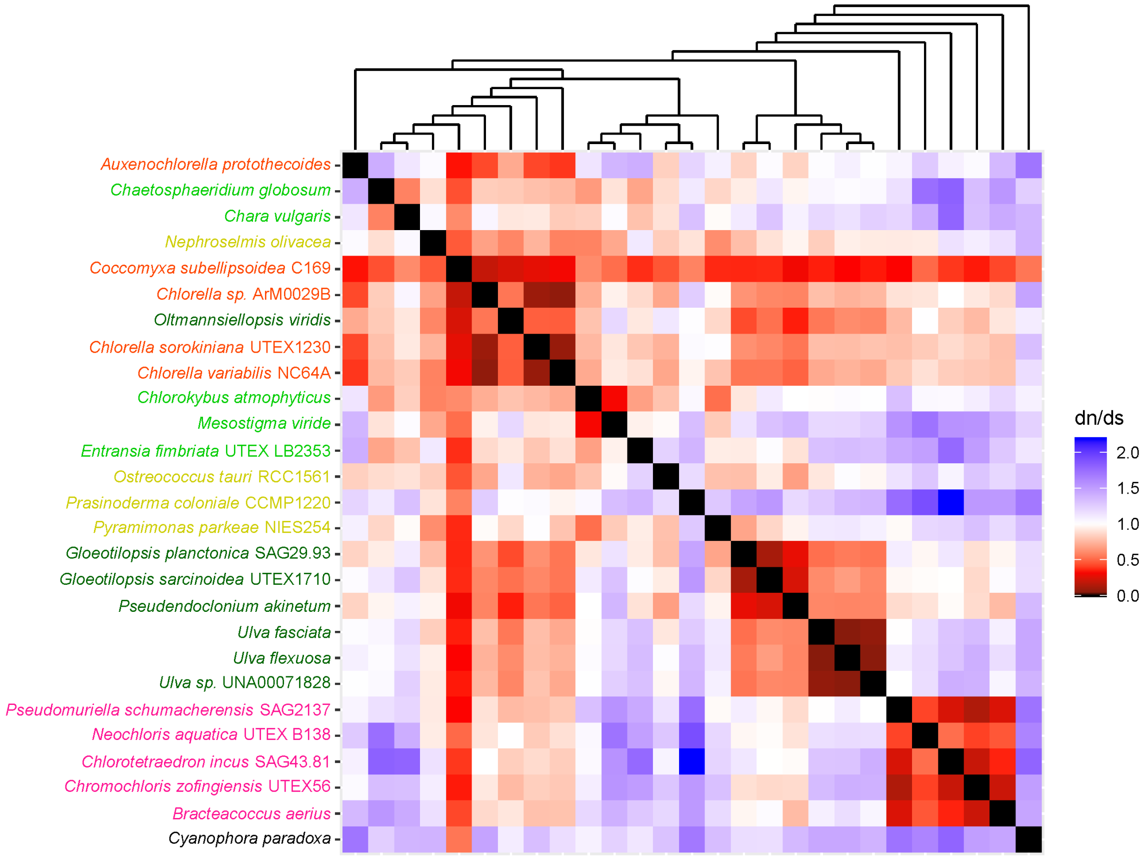

S4 Fig. AT. Heatmap of pairwise dN/dS ratios calculated from alignment of plastid *psb*F of algal taxa in Table 1 by MEGA7.0 using modified Nei-Gojobori method (Jukes-Cantor). The scale bar showed the color corresponded to dn/ds ratios. The cladogram above the heatmap corresponded to its single-gene phylogenetic tree.

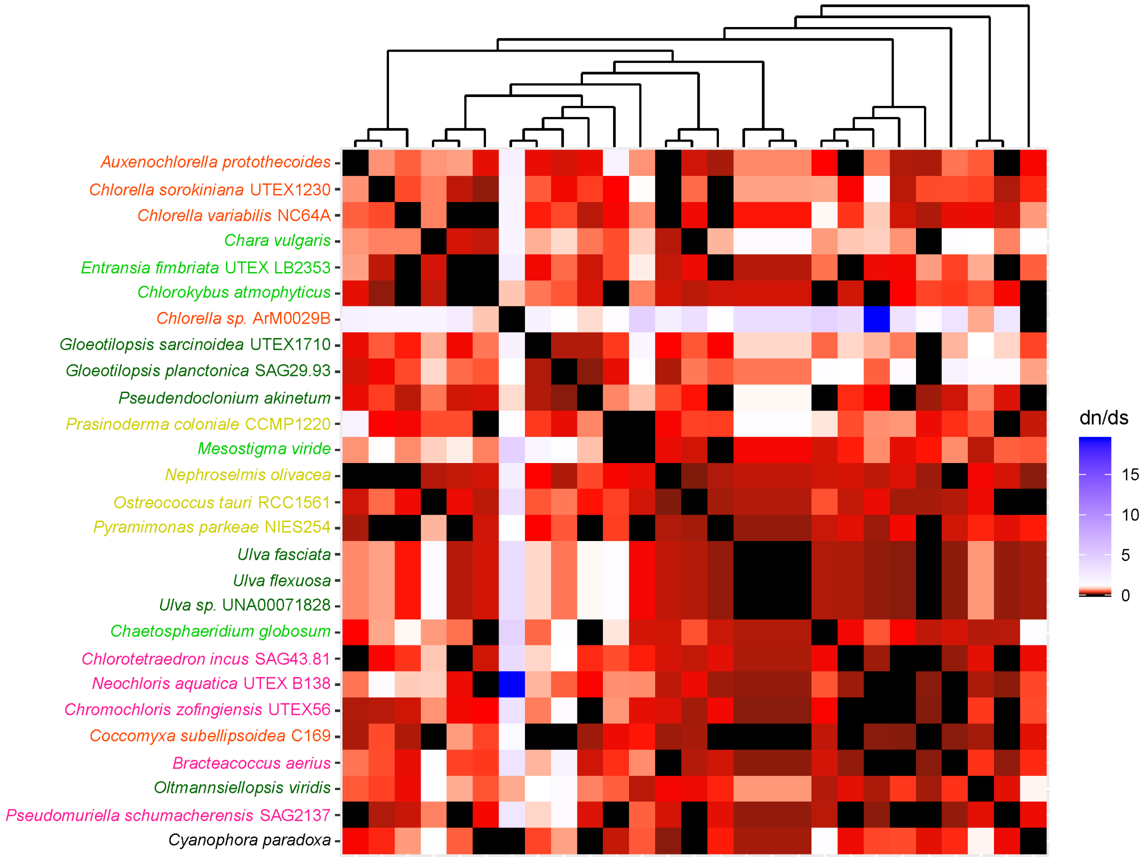

S4 Fig. AU. Heatmap of pairwise dN/dS ratios calculated from alignment of plastid *psb*J of algal taxa in Table 1 by MEGA7.0 using modified Nei-Gojobori method (Jukes-Cantor). The scale bar showed the color corresponded to dn/ds ratios. The cladogram above the heatmap corresponded to its single-gene phylogenetic tree.

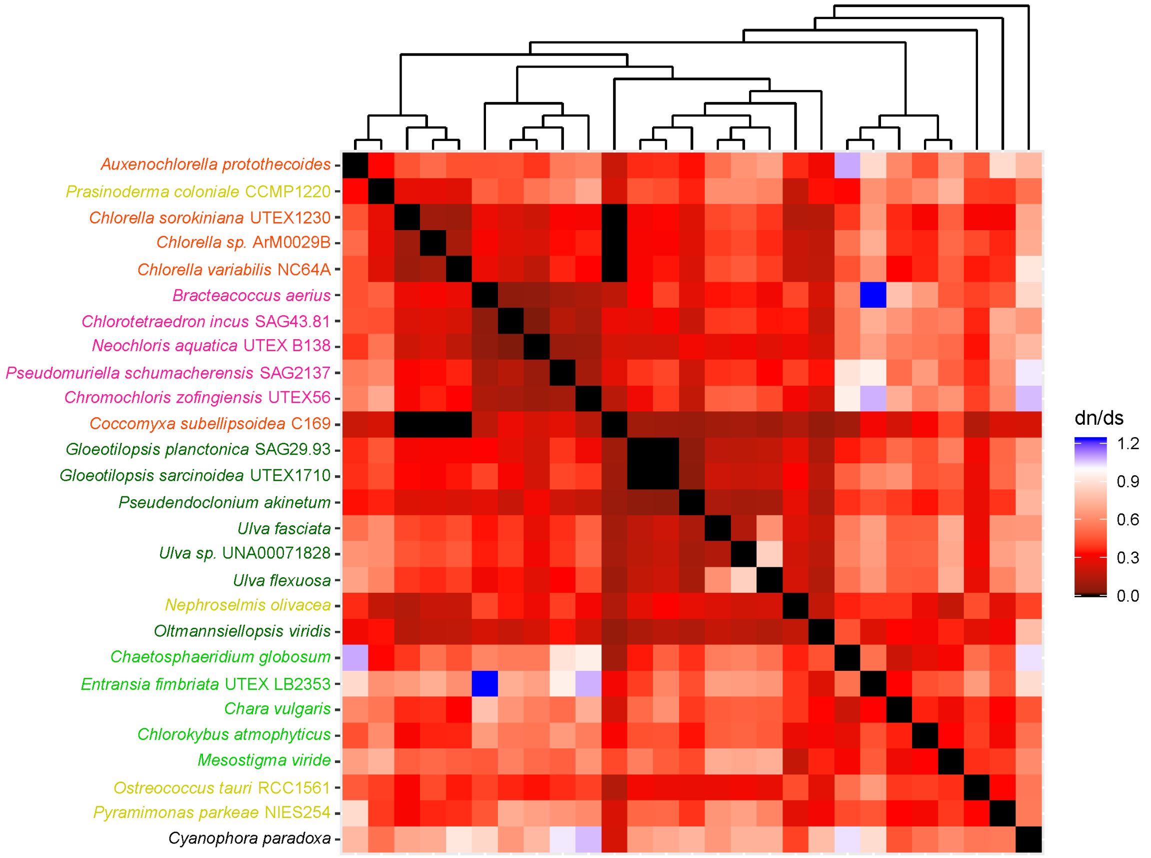

S4 Fig. AV. Heatmap of pairwise dN/dS ratios calculated from alignment of plastid *psbZ* of algal taxa in Table 1 by MEGA7.0 using modified Nei-Gojobori method (Jukes-Cantor). The scale bar showed the color corresponded to dn/ds ratios. The cladogram above the heatmap corresponded to its single-gene phylogenetic tree.

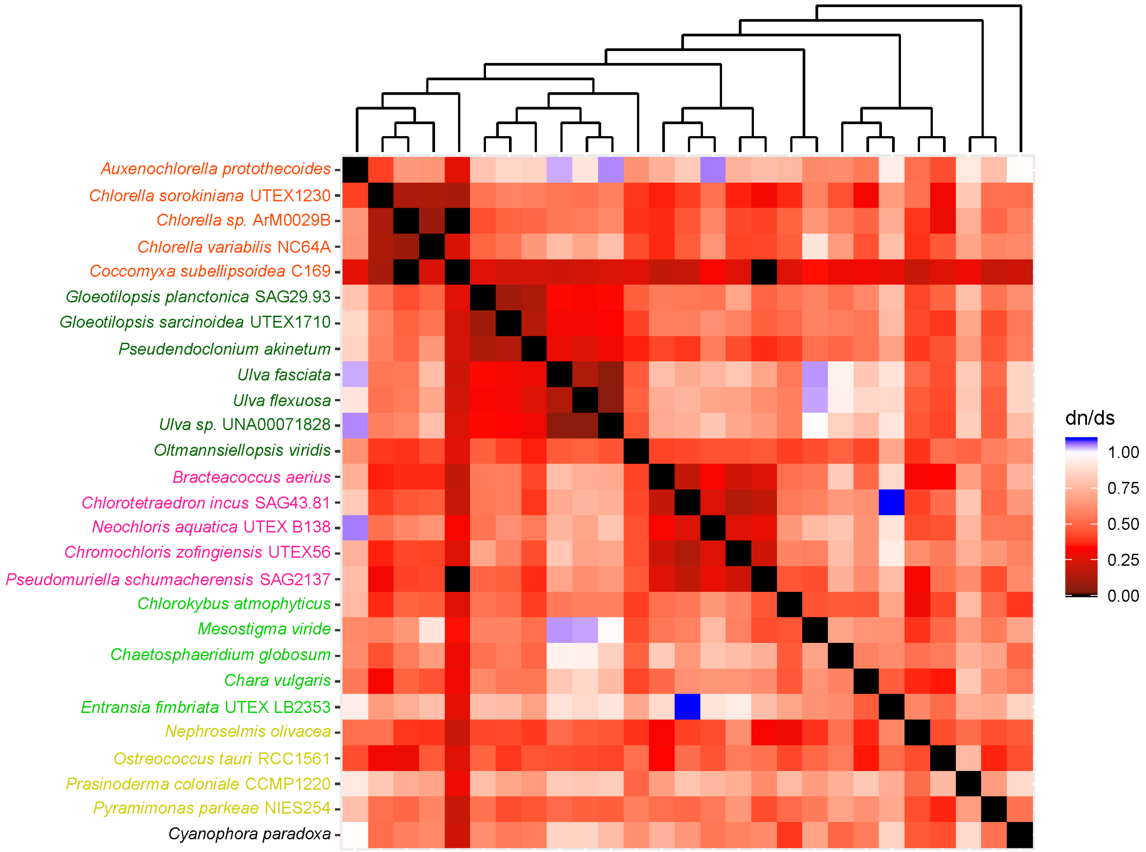

S4 Fig. AW. Heatmap of pairwise dN/dS ratios calculated from alignment of plastid *rpl*20 of algal taxa in Table 1 by MEGA7.0 using modified Nei-Gojobori method (Jukes-Cantor). The scale bar showed the color corresponded to dn/ds ratios. The cladogram above the heatmap corresponded to its single-gene phylogenetic tree.

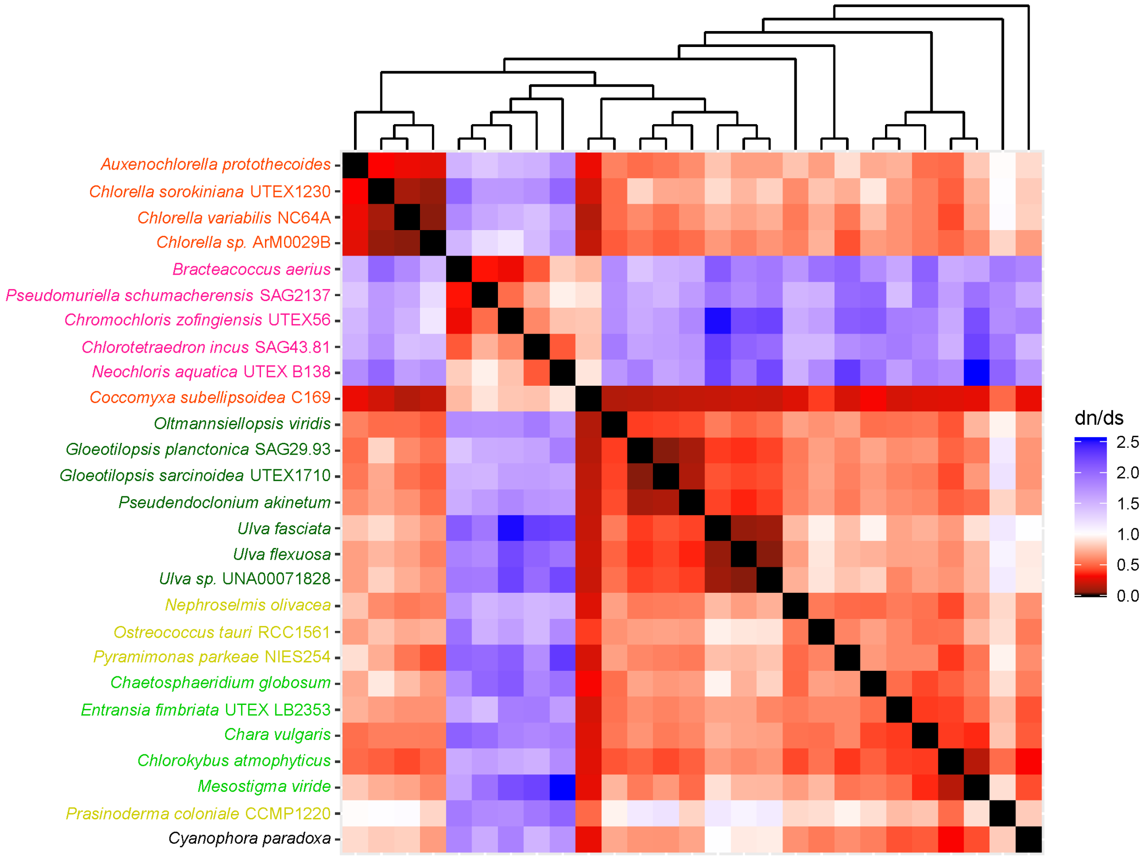

S4 Fig. AX. Heatmap of pairwise dN/dS ratios calculated from alignment of plastid *rps*2 of algal taxa in Table 1 by MEGA7.0 using modified Nei-Gojobori method (Jukes-Cantor). The scale bar showed the color corresponded to dn/ds ratios. The cladogram above the heatmap corresponded to its single-gene phylogenetic tree.

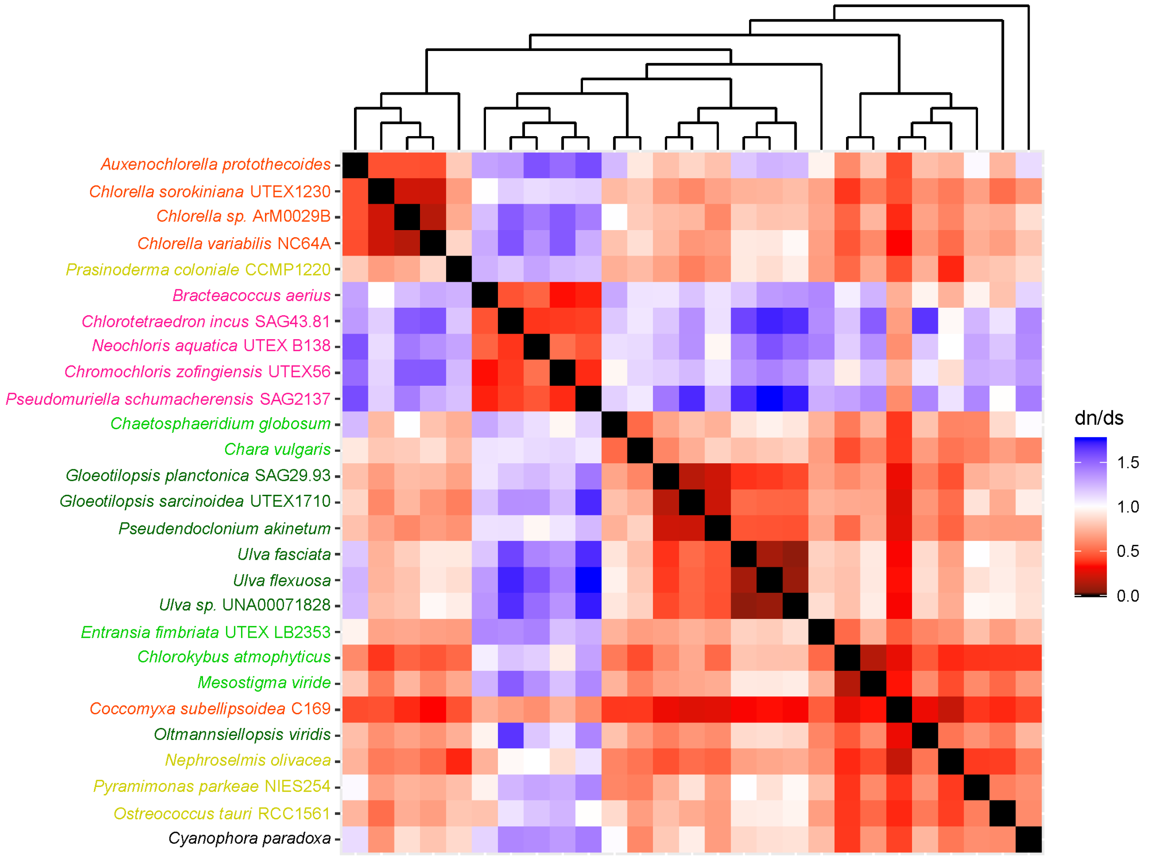

S4 Fig. AY. Heatmap of pairwise dN/dS ratios calculated from alignment of plastid *rps*3 of algal taxa in Table 1 by MEGA7.0 using modified Nei-Gojobori method (Jukes-Cantor). The scale bar showed the color corresponded to dn/ds ratios. The cladogram above the heatmap corresponded to its single-gene phylogenetic tree.

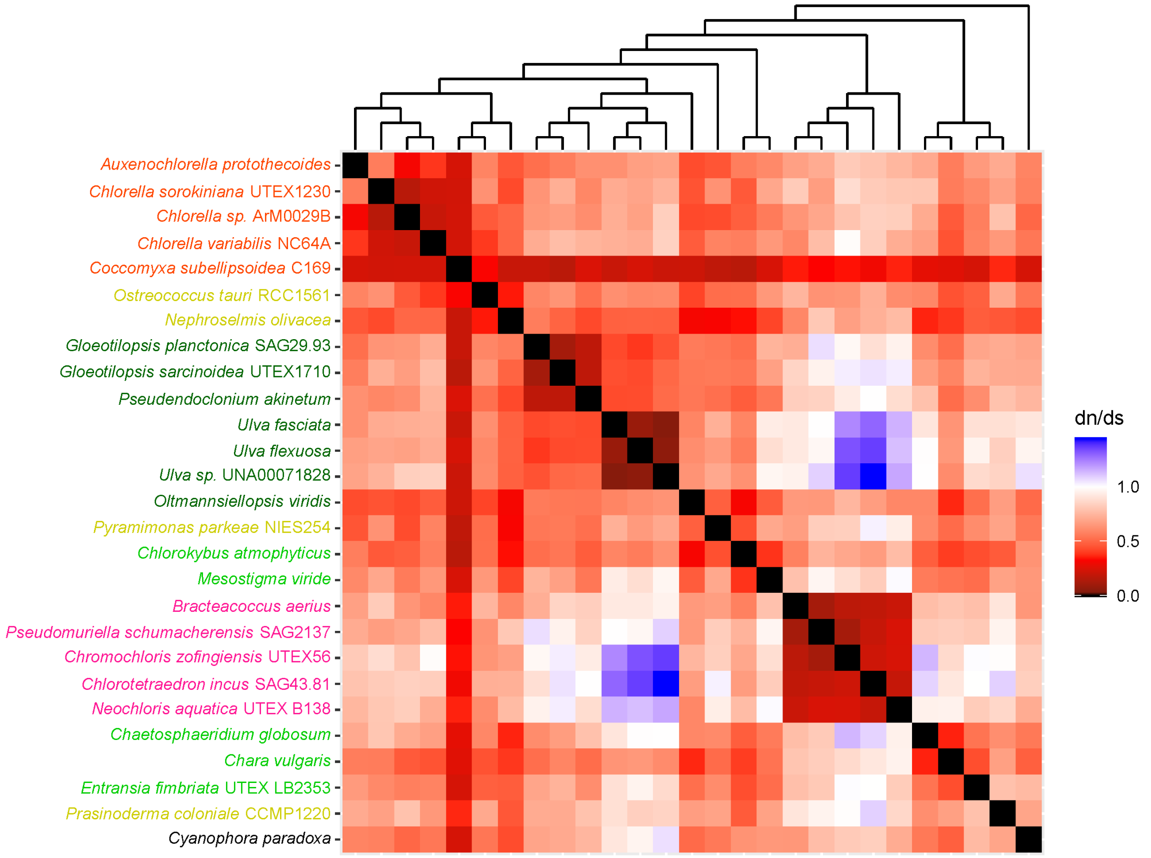

S4 Fig. AZ. Heatmap of pairwise dN/dS ratios calculated from alignment of plastid *rps*4 of algal taxa in Table 1 by MEGA7.0 using modified Nei-Gojobori method (Jukes-Cantor). The scale bar showed the color corresponded to dn/ds ratios. The cladogram above the heatmap corresponded to its single-gene phylogenetic tree.

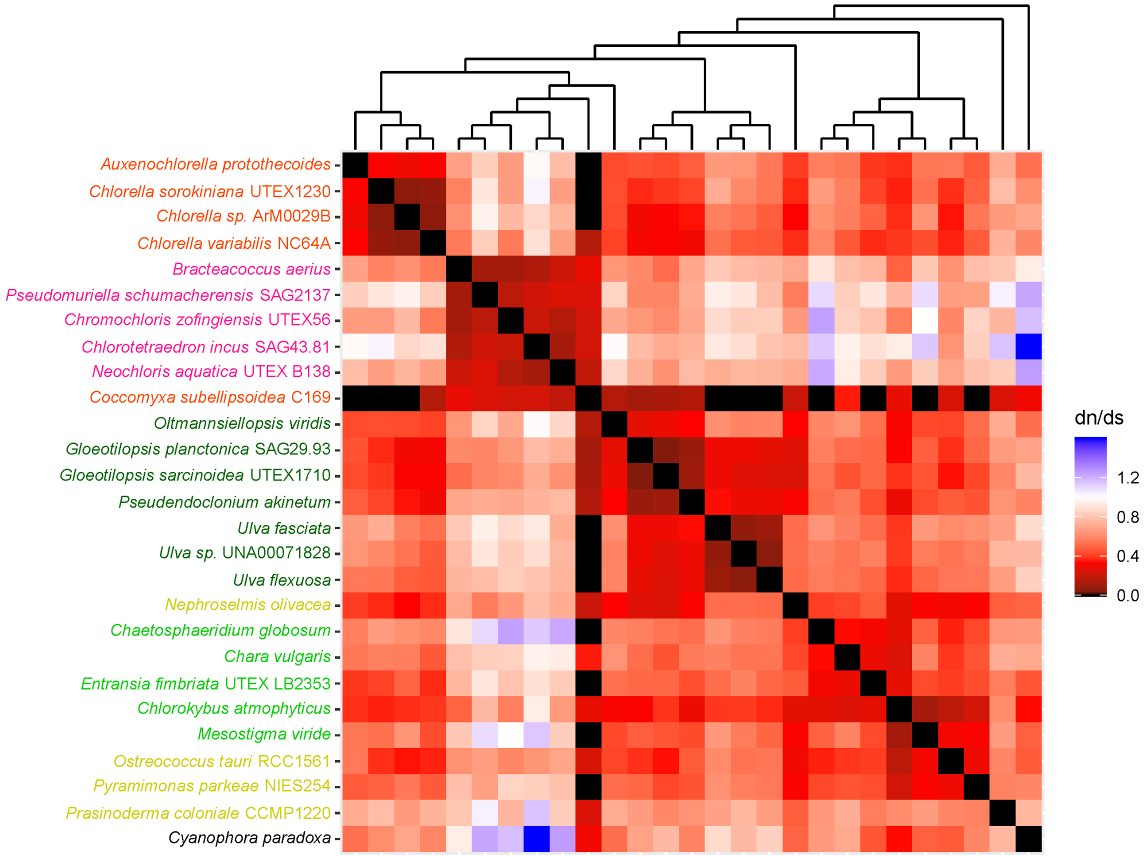

S4 Fig. BA. Heatmap of pairwise dN/dS ratios calculated from alignment of plastid *rps*7 of algal taxa in Table 1 by MEGA7.0 using modified Nei-Gojobori method (Jukes-Cantor). The scale bar showed the color corresponded to dn/ds ratios. The cladogram above the heatmap corresponded to its single-gene phylogenetic tree.

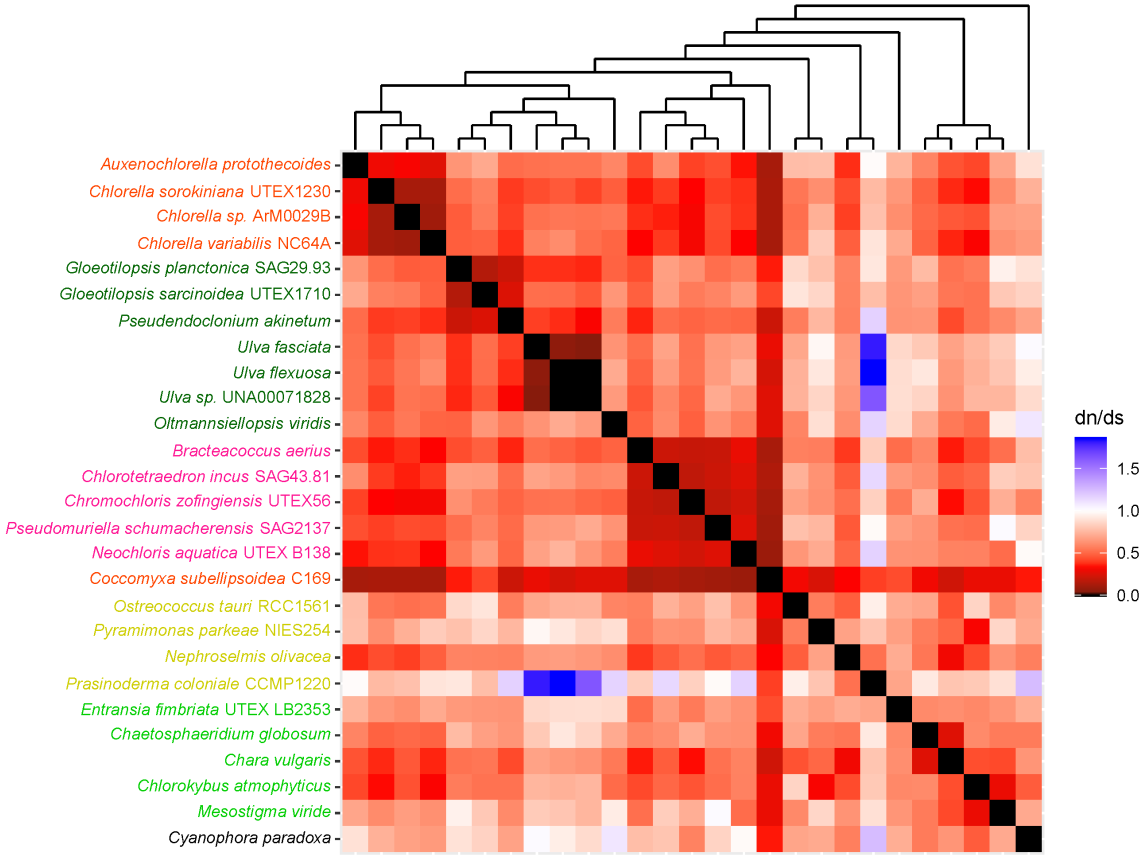

S4 Fig. BC. Heatmap of pairwise dN/dS ratios calculated from alignment of plastid *rps*8 of algal taxa in Table 1 by MEGA7.0 using modified Nei-Gojobori method (Jukes-Cantor). The scale bar showed the color corresponded to dn/ds ratios. The cladogram above the heatmap corresponded to its single-gene phylogenetic tree.

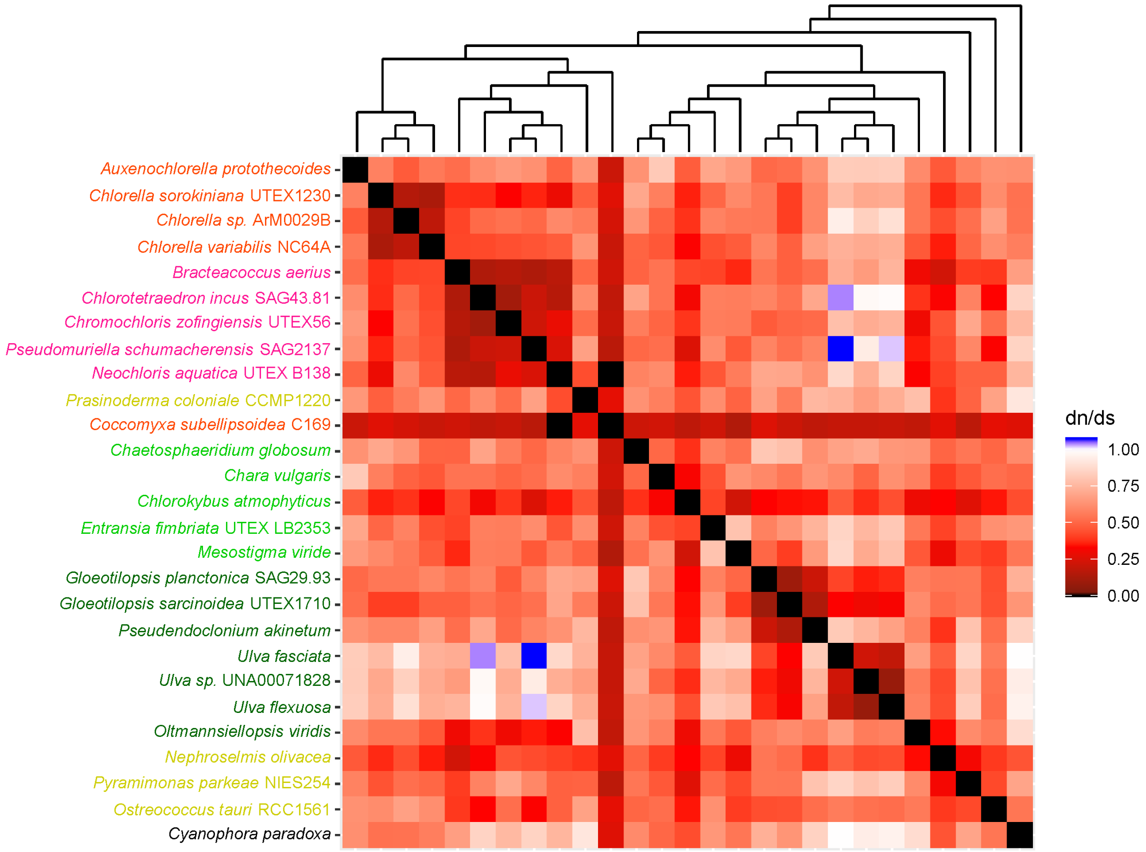

S4 Fig. BD. Heatmap of pairwise dN/dS ratios calculated from alignment of plastid *rps*14 of algal taxa in Table 1 by MEGA7.0 using modified Nei-Gojobori method (Jukes-Cantor). The scale bar showed the color corresponded to dn/ds ratios. The cladogram above the heatmap corresponded to its single-gene phylogenetic tree.

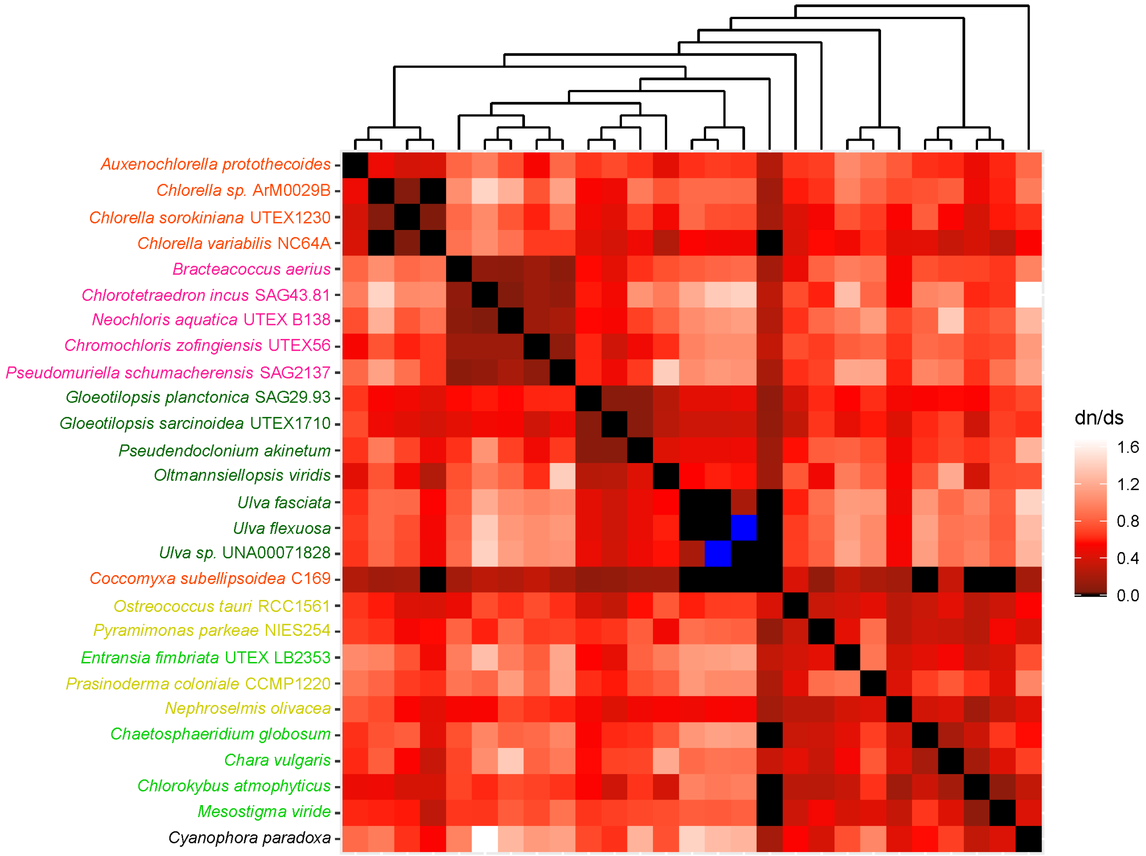

S4 Fig. BE. Heatmap of pairwise dN/dS ratios calculated from alignment of plastid *rps*18 of algal taxa in Table 1 by MEGA7.0 using modified Nei-Gojobori method (Jukes-Cantor). The scale bar showed the color corresponded to dn/ds ratios. The cladogram above the heatmap corresponded to its single-gene phylogenetic tree.

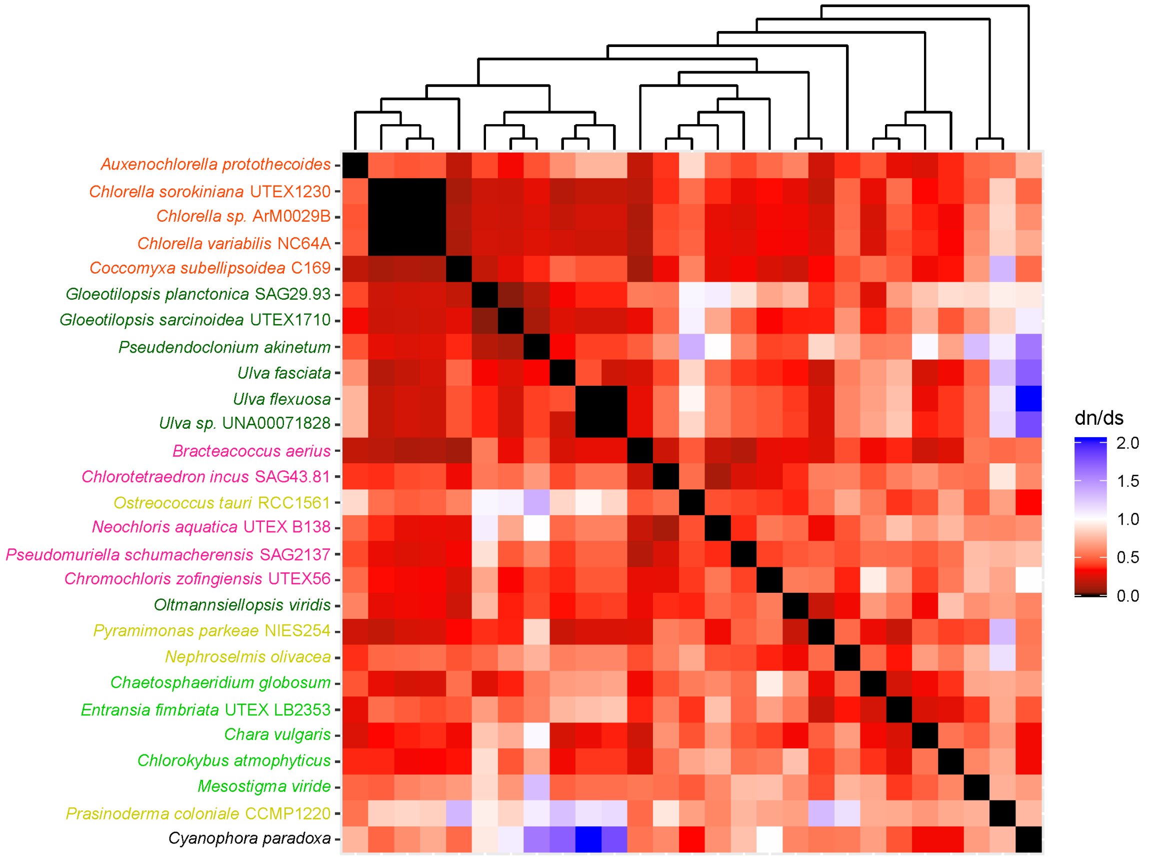

S4 Fig. BF. Heatmap of pairwise dN/dS ratios calculated from alignment of plastid *ycf*12 of algal taxa in Table 1 by MEGA7.0 using modified Nei-Gojobori method (Jukes-Cantor). The scale bar showed the color corresponded to dn/ds ratios. The cladogram above the heatmap corresponded to its single-gene phylogenetic tree.
